# A compromised developmental trajectory of the infant gut microbiome and metabolome in atopic eczema

**DOI:** 10.1080/19490976.2020.1801964

**Published:** 2020-10-06

**Authors:** Le Duc Huy Ta, James Chun Yip Chan, Gaik Chin Yap, Rikky W. Purbojati, Daniela I. Drautz-Moses, Yanqing Michelle Koh, Carina Jing Xuan Tay, Chiung-Hui Huang, Dorinda Yan Qin Kioh, Jia Yun Woon, Elizabeth Huiwen Tham, Evelyn Xiu Ling Loo, Lynette P.C. Shek, Neerja Karnani, Anne Goh, Hugo P.S. Van Bever, Oon Hoe Teoh, Yiong Huak Chan, Christophe Lay, Jan Knol, Fabian Yap, Kok Hian Tan, Yap-Seng Chong, Keith M. Godfrey, Staffan Kjelleberg, Stephan C. Schuster, Eric Chun Yong Chan, Bee Wah Lee

**Affiliations:** a Department of Paediatrics, Yong Loo Lin School of Medicine, National University of Singapore, Singapore, Singapore; b Skin Research Institute of Singapore, A*STAR, Singapore; c Innovations in Food & Chemical Safety Programme, A*STAR; d Singapore Centre For Environmental Life Sciences Engineering (SCELSE), Nanyang Technological University, Singapore; e Department of Pharmacy, Faculty of Science, National University of Singapore, Singapore; f Khoo Teck Puat-National University Children’s Medical Institute, National University Health System, Singapore; g Singapore Institute for Clinical Sciences (SICS), Agency for Science, Technology and Research (A*STAR), Singapore, Singapore; h Department of Paediatrics, KK Women’s and Children’s Hospital, Singapore; i Biostatistics Unit, Yong Loo Lin School of Medicine, National University of Singapore, Singapore, Singapore; j Danone Nutricia Research, Singapore; k Danone Nutricia Research, Utrecht, The Netherlands; l Laboratory of Microbiology, Wageningen University, Wageningen, The Netherlands; m Department of Obstetrics & Gynaecology, National University of Singapore, Singapore; n MRC Lifecourse Epidemiology Unit and NIHR Southampton Biomedical Research Centre, University of Southampton and University Hospital Southampton NHS Foundation Trust, Southampton, UK

**Keywords:** Early life, gut microbiome, gut metabolome, SCFA, atopic eczema, atopic dermatitis, allergen sensitization

## Abstract

Evidence is accumulating that the establishment of the gut microbiome in early life influences the development of atopic eczema. In this longitudinal study, we used integrated multi-omics analyses to infer functional mechanisms by which the microbiome modulates atopic eczema risk. We measured the functionality of the gut microbiome and metabolome of 63 infants between ages 3 weeks and 12 months with well-defined eczema cases and controls in a sub-cohort from the Growing Up in Singapore Toward healthy Outcomes (GUSTO) mother-offspring cohort. At 3 weeks, the microbiome and metabolome of allergen-sensitized atopic eczema infants were characterized by an enrichment of *Escherichia coli* and *Klebsiella pneumoniae*, associated with increased stool D-glucose concentration and increased gene expression of associated virulence factors. A delayed colonization by beneficial *Bacteroides fragilis* and subsequent delayed accumulation of butyrate and propionate producers after 3 months was also observed. Here, we describe an aberrant developmental trajectory of the gut microbiome and stool metabolome in allergen sensitized atopic eczema infants.

The infographic describes an impaired developmental trajectory of the gut microbiome and metabolome in allergen-sensitized atopic eczema (AE) infants and infer its contribution in modulating allergy risk in the Singaporean mother-offspring GUSTO cohort. The key microbial signature of AE is characterized by (1) an enrichment of *Escherichia coli* and *Klebsiella pneumoniae* which are associated with accumulation of pre-glycolysis intermediates (D-glucose) via the trehalose metabolic pathway, increased gene expression of associated virulence factors (invasin, adhesin, flagellin and lipopolysaccharides) by utilizing ATP from oxidative phosphorylation and delayed production of butyrate and propionate, (2) depletion of *Bacteroides fragilis* which resulted in lower expression of immunostimulatory bacterial cell envelope structure and folate (vitamin B9) biosynthesis pathway, and (3) accompanied depletion of bacterial groups with the ability to derive butyrate and propionate through direct or indirect pathways which collectively resulted in reduced glycolysis, butyrate and propionate biosynthesis.

## Graphical abstract

**Figure:**
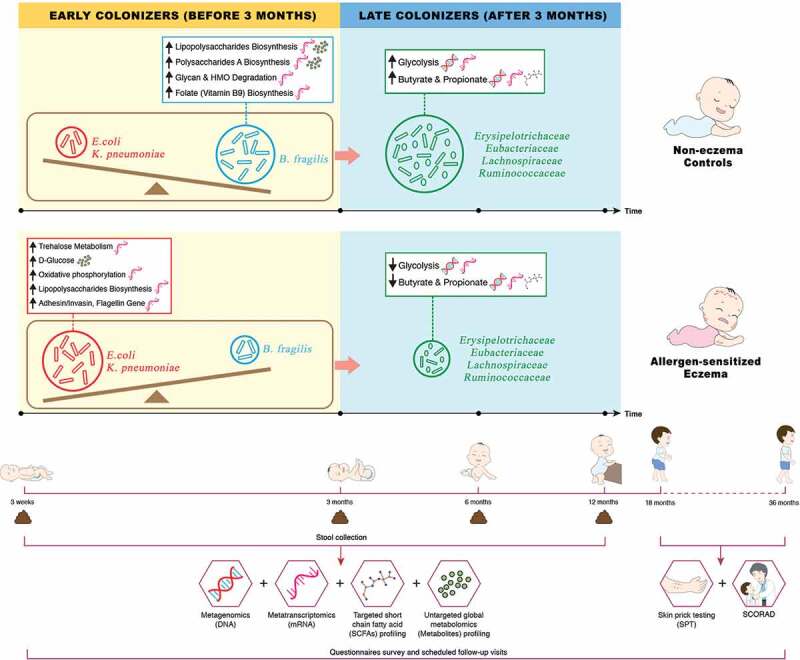

## Introduction

Atopic eczema, a chronic inflammatory skin disorder, is one of the most common childhood non-communicable diseases, affecting up to 20% of children and is one of the earliest manifestations of the atopic march.^1^ The pathogenesis of eczema has been attributed to skin barrier dysfunctions, immune dysregulation as well as environmental-host-microbial interactions.^2^

Environmental factors and modern lifestyle trends have been pinpointed to indirectly contribute to allergy risk through modulation of the gut microbiome (collective genomes and gene products of the microbiota within the human gut).^3,4,5,6^ Evidence is accumulating that the stool metabolome (global collection of small-molecule metabolites found within a stool sample) provides a functional readout of microbial activity and has direct influence on the systemic metabolome and the host through epigenetic modifications as a possible mechanism.^7^ Short-chain fatty acids (SCFAs), a source of energy for colonocytes, liver and muscle cells,^8^ are the major metabolic end products of anaerobic bacterial fermentation in the gut. SCFAs regulate immune function and modulate host metabolism through specific receptor-mediated regulatory pathways.

In support, several birth cohort studies from varied populations in Europe, Asia, and Canada have demonstrated that lower stool SCFAs levels or butyrate producers in infants influence the development of atopic diseases. Roduit et al. demonstrated in the European PASTURE cohort that infants with the highest levels of stool butyrate and propionate (≥95th percentile) at 1 year of age had significantly less atopic sensitization, diagnosed food allergy, allergic rhinitis and asthma between 3 and 6 years of age.^9^ In the mainly Asian PATCH cohort, infants with eczema by 18 months displayed a decreased acquisition of lactate-utilizing butyrate producers (*Eubacterium* and *Anaerostipes*) manifesting with increased stool lactate and decreased butyrate levels in the first 26 weeks of life.^10^ In a Korean case study, Song et al. found that stool samples from patients with eczema had decreased levels of butyrate and propionate, associated with reduced number of butyrate and propionate producer – *Faecalibacterium prausnitzii*.^11^ The Canadian CHILD birth cohort showed that lower stool acetate levels in infants was associated with childhood atopic wheeze.^12^ In a subsequent publication, this cohort also demonstrated a low butyrate production accompanied by reduced expression of microbial genes encoding key enzymes for butyrate biosynthesis in 3-month old infants with allergen sensitization.^13^ These reports suggested the lower production of SCFAs in allergic infants is partly due to the depletion of their corresponding SCFAs producers and this signature in allergic infants is common across different parts of the world.

Using multi-omics approach (metagenomics, metatranscriptomics and metabolomics), we leveraged on a clinically well-characterized mother-offspring cohort, to monitor and compare the gut microbiome and metabolome of infants with early onset eczema (up till 36 months of age) and controls in the first year of life. Here, we hypothesize that infants with atopic eczema and concomitant allergen sensitization present an altered gut microbiome and metabolome in the first 3 weeks of life that led to a subsequent depletion of butyrate and propionate producers. These early events were characterized by an enrichment of *Escherichia coli* and *Klebsiella pneumoniae* accompanied by increased gene expression of virulence factors (invasin, adhesin, flagellin and lipopolysaccharides). These observations were accompanied by a delayed colonization by beneficial *Bacteroides fragilis*, and subsequent depletion of butyrate and propionate producers at ages 6 and 12 months. For the first time, our findings illuminates comprehensive multi-omics insights by unraveling the putative microbial pathways underlying the susceptibility to atopic eczema in early life.

## Results

### Participant characteristics

The overall demographic, allergy risk factors and clinical characteristics of the 3 clinical groups (allergen sensitized atopic eczema, AE; non-allergen sensitized atopic eczema, NAE; and non-eczema controls) are summarized in Table 1. Only the parental history of eczema differed between groups. The mean age of onset of eczema symptoms in the AE group (3.7 months, range 1 to 18 months) was earlier than that of NAE group (6.4 months, range 1.5 to 18 months). AE participants had a higher mean SCORAD compared to NAE participants at 18 months (23.7 ± 14.0 vs 15.6 ± 5.0) and 36 months (30.1 ± 21.5 vs 17.2 ± 6.9) although this did not reach statistical significance. In terms of allergic comorbidities, food allergy was observed only in the AE participants (*p* = .024). The AE group also had more wheeze and rhinitis compared to controls (*p* < .05) (Table 2). Taking into account of missing samples at different timepoints, there were no significant differences in demographic and clinical confounders between groups for the remaining samples (data not shown).

**Table 1.** Demographic and clinical profiles of the three clinical groups.

	Control		All Eczema (Non Allergen Sensitized Atopic Eczema & Allergen Sensitized Atopic Eczema)		Non Allergen Sensitized Atopic Eczema (NAE)		Allergen Sensitized Atopic Eczema (AE)	
	N = 30		N = 33		N = 14		N = 19	
	n	%	n	%	n	%	n	%
DEMOGRAPHIC								
Male gender	12	(40.00)	18	(54.55)	6	(42.86)	12	(63.16)
Presence of sibling	8	(26.67)	14	(42.42)	8	(57.14)	6	(31.58)
Cesarean delivery	10	(33.33)	13	(39.39)	4	(28.57)	9	(47.37)
Pre-term gestation (<37 week)	0	(0.00)	3	(9.09)	1	(7.14)	2	(10.53)
Maternal antibiotics during pregnancy	8	(26.67)	5	(15.15)	2	(14.29)	3	(15.79)
Maternal antibiotics during labor	8	(27.59)	10	(30.30)	7	(50.00)	3	(15.79)
Postnatal antibiotics within first year	6	(20.00)	8	(24.24)	3	(21.43)	5	(26.32)
Ethnicity								
Chinese	13	(43.33)	20	(60.61)	8	(57.14)	12	(63.16)
Malay	9	(30.00)	10	(30.30)	5	(35.71)	5	(26.32)
Indian	8	(26.67)	3	(9.09)	1	(7.14)	2	(10.53)
Infant care attendance within first year	1	(5.88)	3	(9.09)	2	(22.22)	1	(7.14)
Feeding history in 1^st^ 6 months								
Exclusive breastfeeding	2	(6.67)	3	(9.09)	0	(0.00)	3	(15.79)
Predominantly breastfeeding	1	(3.33)	8	(24.24)	3	(21.43)	5	(26.32)
Partial breastfeeding	11	(36.67)	9	(27.27)	2	(14.29)	7	(36.84)
Exclusively formula	16	(53.33)	13	(29.29)	9	(64.29)	4	(21.05)
CLINICAL								
Maternal history								
Any of atopic diseases	4	(13.33)	13	(39.39)*	7	(50.00)*	6	(31.58)
Rhinitis	2	(6.67)	5	(15.15)	3	(21.43)	2	(10.53)
Eczema	1	(3.33)	9	(27.27)*	4	(28.57)*	5	(26.32)*
Asthma	4	(13.33)	5	(15.20)	2	(14.29)	3	(15.79)
Paternal History								
Any of atopic diseases	6	(20.00)	11	(33.33)	3	(21.43)	8	(42.11)
Rhinitis	1	(3.33)	6	(18.18)	3	(21.43)	3	(15.79)
Eczema	1	(3.33)	5	(15.15)	0	(0.00)	5	(26.32)*
Asthma	4	(13.33)	4	(12.12)	1	(7.14)	3	(15.79)

*Significant at *P* < .05 compared with the healthy group in univariate analysis.

**Table 2.** Eczema characteristics of the three clinical groups.

	Control		All Eczema (Non Allergen Sensitized Atopic Eczema & Allergen Sensitized Atopic Eczema)		Non Allergen Sensitized Atopic Eczema (NAE)		Allergen Sensitized Atopic Eczema (AE)	
	N = 30		N = 33		N = 14		N = 19	
	n	%	n	%	n	%	n	%
Age of onset of eczema								
Before 3rd month			12	(40.00)	3	(27.27)	9	(47.37)
Between 3rd and 6th month			11	(36.67)	3	(27.27)	8	(42.11)
Between 6th and 12th month			4	(13.33)	3	(27.27)	1	(5.26)
Between 12th and 18th month			3	(10.00)	2	(18.18)	1	(5.26)
Mean ± SD (Range)			4.7 ± 4.6 (1.0–18.0)		6.4 ± 5.6 (1.5–18.0)		3.7 ± 3.7 (1.0–18.0)	
SCORAD Index Score†								
At 18 Months – Mean ± SD (Range)			19.7 ± 11.0 (7.1–46.1)		15.6 ± 5.0 (10.7–20.3)		23.7 ± 14.0 (7.1–46.1)	
At 36 Months – Mean ± SD (Range)			25.0 ± 17.8 (7.1–69.4)		17.2 ± 6.9 (7.1–22.7)		30.1 ± 21.5 (12.8–69.4)	
Other allergic co-morbiditides								
Wheezing	0	(0.00)	6	(18.75)*	2	(14.29)	4	(22.22)*
Rhinitis	3	(10.00)	20	(60.61)*	9	(64.29)*	11	(57.89)*
Food allergy	0	(0.00)	6	(18.75)*	0	(0.00)	6	(33.33)*

†SCORing of Atopic Dermatitis severity assessments (SCORAD) were performed by trained physicians at 18 and 36 months for evaluation of eczema severity.

*Significant at *P* < .05 compared with the healthy group in univariate analysis.

In this study, we used a “reverse engineering” approach to deconvolute the multi-omics dataset by first delving into the metabolome between the clinical groups. Subsequently, the role of other dominant bacterial species at the early timepoints were then investigated to deduce the potential underlying mechanism involved in the pathogenenesis of atopic eczema. The aim was to investigate and unravel the putative microbiome and metabolome risk phenotype of atopic eczema.

### Depletion of SCFAs (acetate/propionate/butyrate), butyrate/propionate-producing bacterial families/species and related functional gene expression in AE group

**Figure 1.**
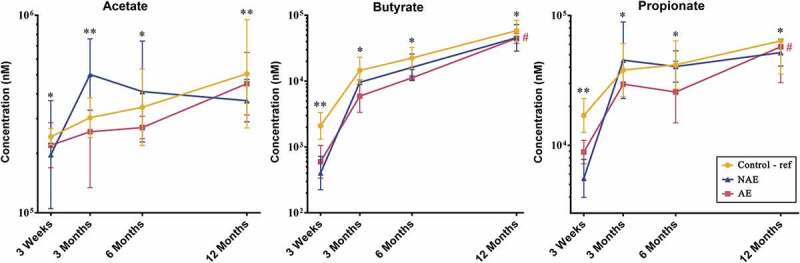
Longitudinal comparison of 3 major stool SCFAs by 3 clinical outcomes. Data are presented as geometric mean and geometric standard deviation range of absolute concentration (nM) in log-scale. Linear mixed-model and general linear model were used to assess difference of abundance between the eczema (AE/NAE) and control (reference) groups adjusting for confounders (gender, birth order, mode of delivery, breastfeeding till 6 months, antibiotics at labor and family of atopic history). List of SCFAs and the comparison of abundance between eczema (NAE or AE) and control (reference) at individual timepoints and longitudinal mean differences are shown in Supplementary Table 6a. *Significant difference at adj *P* < .05 between AE only and control group at specific time point. **Significant difference at adj *P* < .05 between both AE and NAE compared to control groups at specific time point. # (red) annotates significant longitudinal difference between AE and control at adj *P* < .05. Sample size at 3 weeks – Control (n = 13) vs NAE (n = 5) vs AE (n = 5); 3 months – Control (n = 16) vs NAE (n = 11) vs AE (n = 10); 6 months – Control (n = 27) vs NAE (n = 9) vs AE (n = 14) and 12 months – Control (n = 26) vs NAE (n = 8) vs AE (n = 18).

Of the nine varieties of stool SCFAs analyzed, acetate, propionate and butyrate were the most abundant SCFA among all clinical groups across all four timepoints. They accounted for about 98% of the total SCFAs analyzed (data not shown). Compared to controls, lower absolute concentrations of acetate, butyrate and propionate levels were observed in AE (adj *p* < .05), but not in NAE subjects at all time points. Taking into account of longitudinal mean difference, only the reduction in butyrate and propionate levels was significantly associated with AE compared to controls after adjustment for confounders (adj *p* < .05) (Figure 1). Longitudinal analysis of other SCFAs did not show significant differences between groups (Supplementary Figure 1).

**Figure 2.**
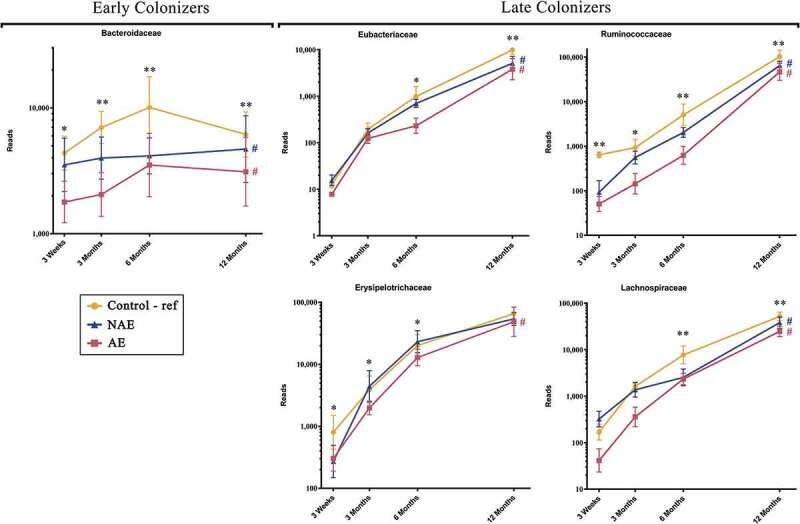
Longitudinal maturation of major butyrate-producing, propionate-producing families in the first year of life. Bacterial community were divided into 2 groups: “Early Colonizers” consisting of major bacterial family and their corresponding predominant species that are dominant from week 3 till month 6: *Bacteroidaceae* (*Bacteroides fragilis*) and “Late Colonizers” consisting of major bacterial families and their corresponding predominant species that are dominant after month 6: *Eubacteriaceae* (*Eubacterium ramulus, Eubacterium limosum, Eubacterium hallii), Erysipelotrichaceae* (*Erysipelatoclostridium ramosum), Lachnospiraceae* (*Anaerostipes caccae, Blautia wexlerae, Blautia producta, Lachnospiraceae bacterium, Tyzzerella nexilis*) and *Ruminococcaceae* (*Faecalibacterium prausnitzii, Ruminococcus gnavus, Ruminococcus sp. JC304*). Data are presented as geometric mean and geometric standard deviation range of reads in log-scale. Different y-axis scales between graph were used to highlight the difference of microbiota between groups. Linear mixed-model and general linear model were used to assess difference of abundance between the eczema (AE/NAE) and control (reference) groups adjusting for confounders (gender, birth order, mode of delivery, breastfeeding till 6 months, antibiotics at labor and family of atopic history). Comparison of key colonizers at individual timepoints and longitudinal mean differences between eczema (NAE or AE) and control (reference) were listed in Supplementary Table 1b. *Significant difference at adj *p* < .05 between AE only and control group at specific time point. **Significant difference at adj *p* < .05 between both AE and NAE compared to control groups at specific time point. # (blue) annotates significant longitudinal difference between NAE and control at adj *P* < .05. # (red) annotates significant longitudinal difference between AE and control at adj *P* < .05. Sample size at 3 weeks – Control (n = 13) vs NAE (n = 5) vs AE (n = 5); 3 months – Control (n = 16) vs NAE (n = 11) vs AE (n = 10); 6 months – Control (n = 27) vs NAE (n = 9) vs AE (n = 14) and 12 months – Control (n = 26) vs NAE (n = 8) vs AE (n = 18).

Based on Spearman correlation and functional gene carriage as described in methods, five major butyrate- and propionate-producing families and their 13 corresponding species were shortlisted and analyzed according to their abundance (read counts) (Figure 2). They included the following: (1) “Early colonizers” from week 3 till month 3 – *Bacteroidaceae* and (2) “Late colonizers” after month 3 – *Erysipelotrichaceae, Eubacteriaceae, Lachnospiraceae* and *Ruminococcaceae* (Supplementary Table 1a). Between ages 3 week and 12 months, normal succession of butyrate and propionate producers belonging to *Bacteroidaceae, Eubacteriaceae, Lachnospiraceae* and *Ruminococcaceae* families were disrupted in both AE and NAE compared to controls (adj *p* < .05) except for *Erysipelotrichaceae* of which only longitudinal difference was observed between AE and controls (adj *p* < .05) (Figure 2). The pairwise comparision between clinical groups for the 13 dominant bacterial species belonging to these 5 families were shown in Supplementary Table 1b. Of note, longitudinal difference between AE and controls for *Bacteroides fragilis, Anaerostipes caccae, Blautia producta, Blautia wexlerae, Lachnospiraceae bacterium, Tyzzerella nexilis, Eubacterium limosum, Eubacterium hallii, Eubacterium ramulus, Faecalibacterium prausnitzii, Ruminococcus gnavus, Ruminococcus sp. JC304* and *Erysipelatoclostridium ramosum* (adj <0.05) was observed. These 13 dominant species were collectively lower in abundance in AE, but not in NAE, compared to controls (adj *p* < .05) (Supplementary Figure 2).

**Figure 3.**
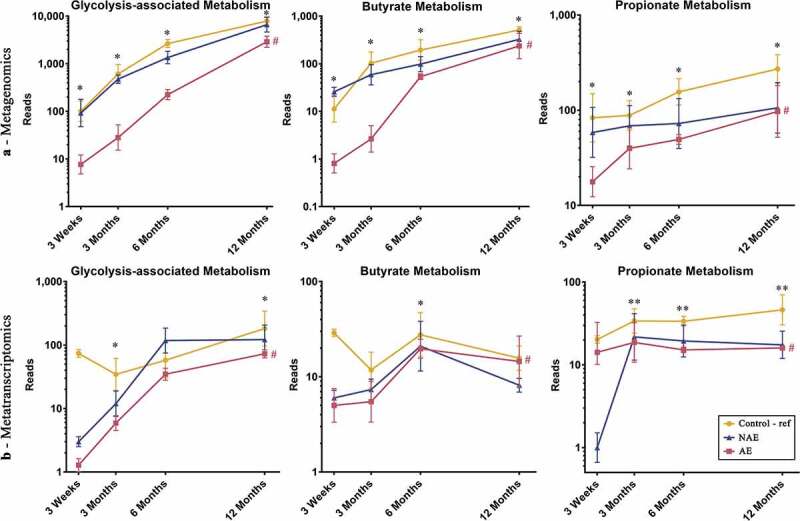
Longitudinal distribution of functional genes belonging to glycolysis, butyrate and propionate biosynthesis pathway of major butyrate-producing and propionate-producing bacterial species from (a) metagenomics and (b) metatranscriptomics. All relevant functional genes carried by shortlisted species were grouped into “Glycolysis-associated Metabolism”, “Butyrate Metabolism” and “Propionate metabolism” as described in methods (list of functional genes in Supplementary Table 5). The cumulative read numbers of all genes belonging to the same metabolism pathway were then pooled and plotted as a line plot. Data are presented as geometric mean and geometric standard deviation range of cumulative read number in log-scale. Different y-axis scales between graphs were used to highlight the difference of read counts between groups. Linear mixed-model and general linear model were used to assess difference of abundance between the eczema (AE/NAE) and control (reference) groups adjusting for confounders (gender, birth order, mode of delivery, breastfeeding till 6 months, antibiotics at labor and family of atopic history). Abundance data at individual timepoints are listed in Supplementary Table 4a and 4b. *Significant difference at *P* < .05 between AE only and control group at specific time point. **Significant difference at *P* < .05 between both AE and NAE compared to control groups at specific time point. # (red) annotates significant longitudinal difference between AE and control at adj *P* < .05. Sample size for (a) at 3 weeks – Control (n = 13) vs NAE (n = 5) vs AE (n = 5); 3 months – Control (n = 16) vs NAE (n = 11) vs AE (n = 10); 6 months – Control (n = 27) vs NAE (n = 9) vs AE (n = 14) and 12 months – Control (n = 26) vs NAE (n = 8) vs AE (n = 18). Sample size for (b) at 3 weeks – Control (n = 6) vs NAE (n = 2) vs AE (n = 2); 3 months – Control (n = 11) vs NAE (n = 3) vs AE (n = 7); 6 months – Control (n = 17) vs NAE (n = 6) vs AE (n = 11) and 12 months – Control (n = 13) vs NAE (n = 3) vs AE (n = 11).

**Figure 4.**
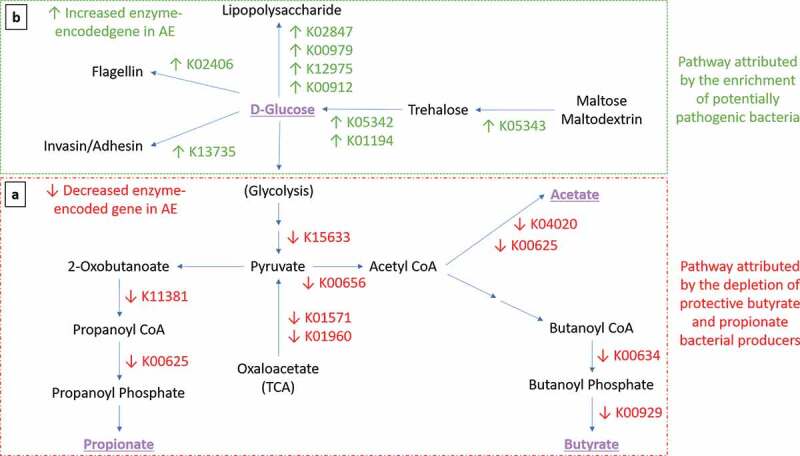
Expression of functional gene profile for carbohydrates metabolism in AE group. Arrows indicate significant differential expression of functional gene in AE compared to control group (*p* < .05). (a-Red Box) Perturbation of carbohydrate metabolism attributed by the depletion of butyrate- and propionate-producing bacteria. Several key carbohydrate metabolism genes were significantly reduced in AE (adj *p* < .05) from the glycolysis pathway (K15633 2,3-bisphosphoglycerate-independent phosphoglycerate mutase, K01571 oxaloacetate decarboxylase and K01960 pyruvate carboxylase subunit B); butyrate biosynthesis pathway (K00634 phosphate butyryltransferase, K00929 butyrate kinase); acetate biosynthesis pathway (K04020 phosphotransacetylase, K00625 phosphate acetyltransferase) and propionate biosynthesis pathway (K01734 methylglyoxal synthase, K00005 glycerol dehydrogenase, K01699 propanediol dehydratase, K13922 propionaldehyde dehydrogenase, K11381 2-oxoisovalerate dehydrogenase, K00625 phosphate acetyltransferase). (b-Green Box) Pathway attributed by the enrichment of potentially pathogenic bacteria. In contrast, several key genes were found to be enriched in AE compared to controls (adj *p* < .05) in the trehalose metabolism pathway (K05343 maltose alpha-D-glucosyltransferase/alpha-amylase, K05342 alpha,alpha-trehalose phosphorylase and K01194 alpha,alpha-trehalase); lipopolysaccharide (LPS) biosynthesis (K02847 O-antigen ligase, K00979 3-deoxy-manno-octulosonate cytidylyltransferase, K12975 KDO II ethanol-amine-phosphotransferase and K00912 tetra-acyl-disaccharide 4ʹ-kinase) and virulence components biosynthesis pathways (K13735 adhesin/invasin, K02406 flagellin). List of metatranscriptomics functional annotation that are significantly different between AE and controls is shown in Supplementary Table 5.

Furthermore, the functional gene expression patterns of all 13 bacterial species contributing to the glycolysis-associated, butyrate and propionate metabolic pathways were then analyzed (Supplementary Table 2). When compared to controls, a lower sum of the abundance read counts of gene carriage (metagenomics) and functional gene transcripts (metatranscriptomics) belonging to these metabolic pathways was observed in AE (adj *p* < .05), but not NAE (Figure 3 and Supplementary Table 3, 4a, 4b). Specific functional genes from 13 species that were differentially expressed (adj *p* < .05) between AE and controls were identified and the pathways of the key genes involved are illustrated in Figure 4a. Several key carbohydrate metabolism genes from the glycolysis-associated, butyrate, acetate and propionate metabolic pathways were significantly reduced in AE (adj *p* < .05) (Supplementary Table 5a).

### Accumulation of sugar metabolites from glycolysis-associated pathway and depletion of intermediates in butyrate and propionate biosynthesis pathway in AE infants

**Figure 5.**
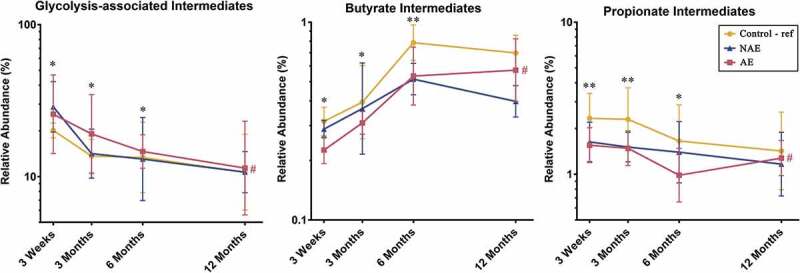
Distribution of intermediate metabolites belonging to the glycolysis-associated pathway, butyrate and propionate biosynthesis pathways (list of intermediate metabolites in Supplementary Table 10). All physiologically and metabolically relevant metabolites were grouped into “Glycolysis-associated intermediates”, “Butyrate intermediates” or “Propionate intermediates metabolites” based on the KEGG database. Data are presented as geometric mean and geometric standard deviation range of relative abundance (%) in log-scale. Linear mixed-model and general linear model were used to assess difference of abundance between the eczema (AE/NAE) and control (reference) groups adjusting for confounders (gender, birth order, mode of delivery, breastfeeding till 6 months, antibiotics at labor and family of atopic history). List of predominant metabolites and the comparsion of abundance between eczema (NAE or AE) and control (reference) at individual timepoints and longitudinal mean differences are shown in Supplementary Table 6b. *Significant difference at adj *P* < .05 between AE only and control group at specific time point. **Significant difference at adj *P* < .05 between both AE and NAE compared to control groups at specific time point. # (red) annotates significant longitudinal difference between AE and control at adj *P* < .05. Sample size at 3 weeks – Control (n = 13) vs NAE (n = 5) vs AE (n = 5); 3 months – Control (n = 16) vs NAE (n = 11) vs AE (n = 10); 6 months – Control (n = 27) vs NAE (n = 9) vs AE (n = 14) and 12 months – Control (n = 26) vs NAE (n = 8) vs AE (n = 18).

Data from untargeted stool metabolite profiling was analyzed to evaluate the intermediate metabolites which promote butyrate and propionate production and determine the specific metabolic pathways responsible for the differences in stool butyrate and propionate levels between AE and controls. The physiologically relevant global metabolites were grouped based on the KEGG database into “glycolysis-associated metabolites”, “butyrate intermediates” and “propionate intermediates” as described in methods. Longitudinal analysis showed that compared to controls, AE infants had significantly lower levels of intermediate stool metabolites in the butyrate biosynthesis pathway (predominantly hydroxybutyrate and dihydroxybutanoate) (adj *p* < .05); propionate biosynthesis pathway (predominantly propanoic acid, 2-oxo-3-(trimethylsilyl)-, trimethylsilyl ester) (adj *p* < .05) and a corresponding increase in accumulation of metabolites belonging to the pre-glycolysis pathway (predominantly D-glucose, D-galactose, lactose and lactate) (adj *p* < .05) in the first 6 months (Figure 5 and Supplementary Table 6b). These data suggest that the production of butyrate and propionate intermediate metabolites is impaired in AE infants. Consequently, intermediate metabolites in the pre-glycolysis pathway accumulated in AE, but not NAE subjects, when compared against controls.

### *Early deviation in the developmental trajectory of the gut microbiome of infants with atopic eczema –* Enterobacteriaceae*/*Bacteroidaceae *ratio*

We next explored if other microbial groups contributed to the microbial signature associated with AE. Unsupervised analysis of the microbiome and metabolome (SCFAs) datasets as a whole indicated that the maturation of the microbiome/metabolome was driven by age from 3 weeks toward 12 months (Supplementary Figure 3). The Shannon species diversity in all three groups increased over time, though there were no significant differences observed between clinical groups (Supplementary Figure 4). At the family level, we observed that members of *Bifidobacteriaceae, Enterobacteriaceae, Bacteroidaceae* and *Lachnospiraceae* were predominant across the clinical groups (Supplementary Figure 5).

**Figure 6.**
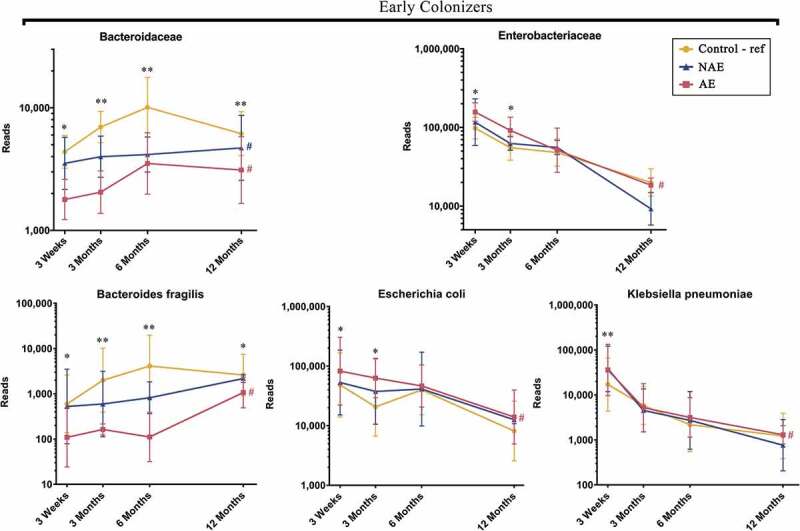
Longitudinal maturation of three early colonizers namely *Bacteroides fragilis* (*Bacteroidaceae* family), *Escherichia coli* (*Enterobacteriaceae* family) and *Klebsiella pneumoniae* (*Enterobacteriaceae* family). Data are presented as geometric mean and geometric standard deviation range of reads in log-scale. Different y-axis scales between graph were used to highlight the difference of microbiota between groups. Linear mixed-model and general linear model were used to assess difference of abundance between the eczema (AE/NAE) and control (reference) groups adjusting for confounders (gender, birth order, mode of delivery, breastfeeding till 6 months, antibiotics at labor and family of atopic history). Comparison of key colonizers at individual timepoints and longitudinal mean differences between eczema (NAE or AE) and control (reference) were listed in Supplementary Table 1b. *Significant difference at adj *p* < .05 between AE only and control group at specific time point. **Significant difference at adj *p* < .05 between both AE and NAE compared to control groups at specific time point. # (blue) annotates significant longitudinal difference between NAE and control at adj *P* < .05. # (red) annotates significant longitudinal difference between AE and control at adj *P* < .05. Sample size at 3 weeks – Control (n = 13) vs NAE (n = 5) vs AE (n = 5); 3 months – Control (n = 16) vs NAE (n = 11) vs AE (n = 10); 6 months – Control (n = 27) vs NAE (n = 9) vs AE (n = 14) and 12 months – Control (n = 26) vs NAE (n = 8) vs AE (n = 18).

Pairwise group comparisons indicated that there was a higher proportion of *Enterobacteriaceae* and a lower abundance of *Bacteroidaceae* in AE compared to controls at 3 weeks and 3 months (adj *p* < .05). *Lachnospiraceae* was only significantly higher in controls than AE at 6, 12 months (adj *p* < .05) (Supplementary Table 1a), whilst there was no statistically significant difference in *Bifidobacteriaceae* between groups (data not shown). We demonstrated in AE, an inverse relationship between *Bacteroidaceae* (*B. fragilis*) and *Enterobacteriaceae* (*E. coli* and *K. pneumoniae*) (Figure 6), manifesting as high ratios of *Enterobacteriaceae*/*Bacteroidaceae, E. coli*/*B. fragilis* and *K. pneumoniae/B. fragilis* that peaked at week 3 and gradually decreased almost to zero at month 12 (Supplementary Figure 6).

### *Enrichment of* Escherichia coli, Klebsiella pneumoniae *and increased carbohydrate metabolism and virulence gene expression in AE*

Compared to controls, AE presented an enrichment of *E. coli* and *K. pneumoniae* in the first 3 weeks of life (adj *p* < .05) (Figure 6). Of these, *E. coli* and *K. pneumoniae* predominated before 6 months of age. Compared to controls, pairwise comparison of carbohydrate gene profiles from *E. coli* and *K. pneumoniae* in AE revealed an increase in several key genes in the trehalose metabolic pathway, which yields from hydrolyzed trehalose D-glucose as substrates for glycolysis (Figure 4b and Supplementary Table 5b). These results are in line with the observed accumulation of pre-glycolysis intermediates such as D-glucose in AE subjects (Figure 5).

In addition, the expression of genes responsible for bacterial LPS, virulence components biosynthesis (adhesin/invasion and flagellin) and oxidative phosphorylation were also increased in AE (Supplementary Table 5b). Further correlation analysis revealed that the increase in D-glucose positively correlated with the increased expression of several virulence genes coding for adhesin/invasin (R = 0.761, *p* < .05), flagellin (R = 0.752, *p* < .05) and LPS biosynthesis (R = 0.805, *p* < .05) in both bacterial species.

### Bacteroides fragilis, *an early life colonizer depleted in AE infants*

In comparison to AE, control infants presented an enrichment of *B. fragilis* from first 3 weeks of life onwards (Figure 6). Transcriptomics-based functional gene analysis of *B. fragilis* revealed that the depletion of *B. fragilis* in AE was associated with lower gene read counts involving glycan degradation, lipopolysaccharides (LPS) biosynthesis, polysaccharide A (PSA) capsule biosynthesis, membrane transport and folate biosynthesis (adj *p* < .05) (Supplementary Table 5). Further correlation analysis between transcriptomic functional gene and metabolome showed a strong positive correlation between the abundance of *B. fragilis* and LPS fatty acid components namely pentadecanoic acid (R = 0.673, *p* < .05).

### Sensitivity Analysis

In lieu of the missing stool data in this cohort at various timepoints, linear mixed modeling was repeatedly performed under two extreme scenarios as described in methods for the two key SCFAs (butyrate and propionate) and three key bacterial species (*B. fragilis, E. coli* and *K. pneumoniae*). For the “best” scenarios, the longitudinal trend of all variables between AE and controls remained significant (adj *p* < .05). For the “worst” scenario, all variables were significant (adj *p* < .05) except for propionate concentration (adj *p* = .062).

## Discussion

This study leveraged an integrated, multi-omics approach to deconvolute the gut microbiome-metabolome cross-talk in infants with atopic eczema in the first year of life. The longitudinal microbial analysis uncovered a pattern of successive microbial colonization at a taxonomic and functional level in atopic eczema. Most of the differences observed in microbial composition, functional gene expression and metabolomes between clinical groups commenced as early as 3 weeks, which in most cases preceded the onset of eczema, suggesting that the early life gut microbiome is likely to influence the risk of developing AE. The abnormalities in the stool microbiome and metabolome were only significant in eczema infants with concomitant allergen sensitization by the age of 36 months (AE), while the data of the non-allergen sensitized atopic eczema group (NAE) was intermediate between AE and controls (Figures 1,2,3,5). We hypothesize that the larger the extent of microbiome dysbiosis, the greater its influence on allergen sensitization and the development of allergic co-morbidities (rhinitis and wheeze) (Table 1). At the study population level, the maturation of the microbiome and metabolome was driven by age indicating a synchronized process that likely have a role in gut maturation and immunity development.^14^ Conversely, a disruption of this process may have a negative impact.

### *Birth to age 3 months: Over-representation of* E. coli *and* K. pneumoniae *and delayed colonization by* B. fragilis *in AE*

In this study, we uncovered an impaired developmental trajectory of the gut microbiome/metabolome in infants with AE. The microbial deviation in AE infants was observed as early as 3 weeks of life and was characterized by a delayed colonization by *Bacteroidaceae family* (*B. fragilis*) with concomitant enrichment of members of the *Enterobacteriaceae* family (*E. coli* and *K. pneumoniae*) (Figure 6), resulting in a high ratio of *Enterobacteriaceae*/*Bacteroidaceae* in AE compared to controls (Supplementary Figure 6). In support, a high *Enterobacteriaceae*/*Bacteroidaceae* ratio has been reported in infants with food allergy.^15,16^ Contrary to other reports that have found reduced *Bifidobacteriaceae* in infants who develop eczema,^17–19^ our study did not find any association of this family at both structural and functional gene level.

We hypothesize that the early colonization by *E. coli* and *K. pneumoniae* in the first weeks of life would subsequently impact the successive establishment of other microbial species, leading to the delayed colonization by butyrate- and propionate-producing bacteria observed in AE. In support, high abundance of *E. coli* was linked to eczema in both longitudinal and cross-sectional studies,^20,21^ and early onset (mean age of 2 months) eczema with high total serum IgE levels.^22^ A study showed that adherent-invasive *E. coli* colonization alters the intestinal commensal microbiome acquisition in a way that increases its pro-inflammatory potential.^23^ At the functional level, we identified multi-omic signatures indicating that in AE infants the presence of *E. coli* and *K. pneumoniae* was associated with the expression of genes involved in the metabolism of trehalose and this was translated to an increased detection of D-glucose in stool samples (Figure 4b). It has been shown that the accumulation of trehalose from dietary maltodextrin/maltose is an exclusive response to stress conditions of *E. coli* and *K. pneumoniae* before undergoing hydrolysis to D-glucose as an alternative energy source.^24,25^ We also observed that the elevations of stool D-glucose was associated with the expression of genes encoding for virulence factors (LPS, adhesin, invasion and flagellin) suggesting that D-glucose could be diverted for the biosynthesis of those virulence factors by utilizing ATP from oxidative phosphorylation. Adhesin, invasin and flagellin have been shown to increase the ability of *E. coli* and *K. pneumoniae* to colonize mucin and epithelial surfaces,^26,27,28^ allowing them to compete with *B. fragilis* for colonization in niches which normally favor *B. fragilis* colonization.^29^ D-glucose is also a well-known key component of LPS core oligosaccharides in *E. coli* and *K. pneumoniae*.^30,31^ Accumulation of stool D-glucose has been reported previously, with stool samples profiled in 3-month old infants with eczema being higher compared to controls.^32^ LPS, found in the outer membrane of Gram-negative *Enterobacteriaceae*, is also known to contribute to atopic risk. Binding of LPS to CD14 and TLR4 receptors leads to activation of MyD88-dependent NF-κB and IRF3, culminating in production of pro-inflammatory cytokines.^33,34^

Conversely, in control infants, the presence of *B. fragilis* indicates its protective role against the development of atopic eczema. *B. fragilis* is transmitted vertically from the maternal microbiota, colonizes the infant gut as early as day three and has the ability to consume Human Milk Oligosaccharides (HMOs).^35,36,37,38^ Current evidence supports breastfeeding, and when not possible formula supplemented with prebiotics and/or probiotics as a nutritional solution may prevent the primary development of eczema by supporting a healthy gut microbiome.^39,40,41^ Studies on the use of prebiotics to specifically support the colonization and enrichment of beneficial *B. fragilis* are needed to demonstrate its potential therapeutic application for eczema prevention. Here, we postulate that the acquisition of *B. fragilis* during birth allows the modulation of a gut environment that would promote the establishment of butyrate and propionate producers. The reduced abundance of *Bacteroides* genus and *B. fragilis* within the first year of life in infants with AE are consistent with other reports.^18,42^ A low abundance of *B. fragilis* in early life has also been associated with Cesarean delivery, a known risk factor for eczema development.^43^ Cesarean delivery was, however, not significantly associated with eczema in our study as mode of delivery was similar in the cases and controls [Control: 10 cases (33.33%) vs Eczema: 13 cases (39.39%)] (Table 1). A study on the larger GUSTO cohort (1077 pregnant women) also supports this observation.^44^

Besides members of the *Enterobacteriaceae*, LPS is also a common feature of *B. fragilis*. In support, we observed a positive association between abundance of *B. fragilis*, LPS biosynthesis gene and LPS component (pentadecanoic acid). Several studies have shown differences in LPS immunogenicity between bacterial species, whereby LPS from *Bacteroides* species promote immune tolerance; while those from *E. coli* induce inflammation due to their structurally distinct LPS structures.^45,46^ Those data suggest that *Bacteroides* LPS could potentially inhibit the immunostimulatory activity of *E. coli* LPS. Hence, increased *E. coli* and *K. pneumoniae* LPS in early life may thus be pro-inflammatory and hence adversely influence the development of immune tolerance.

Additionally, increased expression of essential genes by *B. fragilis* involved in PSA biosynthesis was associated with controls. Studies reported that *B. fragilis* exert anti-inflammatory properties through its PSA which induces IL-10 and IFN-γ, restoring Th1/Th2 balance in the host.^47^ Furthermore, *B. fragilis* carries glycan degradation genes which were shown to be reduced in AE. These enzymes metabolize HMOs consumption due to their structural similarities with glycan.^48^ *B. fragilis* metabolizes HMOs to precursor substrates for butyrate and propionate production, which serve as nutrients for colonocytes and other gut epithelial cells.^49^ Moreover, other relevant functional genes of *B. fragilis* involved in membrane transport and folate biosynthesis were reduced in AE. Membrane transports, especially ATP-binding cassette (ABC) transporters have previously been shown to be immunogenic.^50^ Folate (vitamin B9) is known to be an important nutrient for DNA synthesis, replication, and repair as well as the maintenance of regulatory T cells.^51,52^ Thus, the presence of this early colonizer might be an important determinant for normal immune maturation in early life.

### Beyond age 3 months: Delayed colonization by butyrate and propionate producers and consequent reduction of SCFAs production

Between ages 3 and 12 months, the normal succession of butyrate and propionate producers belonging to *Erysipelotrichaceae, Eubacteriaceae, Lachnospiraceae* and especially *Ruminococcaceae* families were disrupted in AE compared to controls (Figure 2). Other studies substantiate our observations, with *Erysipelatoclostridium* higher in abundance in healthy compared with allergic infants.^53^ A previous report observed that eczema severity correlated inversely with the abundance of *Eubacteriaceae, Lachnospiraceae* and *Ruminococcaceae* in 6-month-old infants.^54^ High-risk infants who developed eczema by 18 months likewise demonstrated delayed acquisition by *Eubacterium* and *Anaerostipes* species and correspondingly reduced butyrate and propionate levels at 26 weeks of age, compared to healthy controls.^10^ *F. prausnitzii* (*Ruminococcaceae* family) is a major component of the healthy human microbiome and is depleted in atopic children.^55^ The Korean COCOA birth cohort study showed that the stool of six-month-old infants with eczema was depleted of *R. gnavus* (*Ruminococcaceae* family) and *L. bacterium* (*Lachnospiraceae* family).^56^

Consequently, the impact of the reduced abundance of these SFCA producers in AE participants was reflected in the accumulation of pre-glycolysis intermediates as well as lower expression of functional genes and transcripts involved in butyrate and propionate biosynthesis (Figures 1, 5). These strengthen the notion that the observed accumulation of stool intermediates derived from the pre-glycolysis pathway and, low butyrate and propionate levels are related to diminished glycolysis, butyrate and propionate biosynthesis capabilities by corresponding SCFAs producers. These finding corroborate with those of several recent cohort studies.^9,10,12,13^ High dietary fiber or live biotherapeutic intervention could regulate protective pathways in the gastrointestinal tract and promote oral tolerance through the modulation of the gut microbiome.^57^ Our findings indicated that the delayed production of SCFA starts to occur around the weaning period. A recent preclinical study related to the weaning reaction, a key period that allows the establishment of the SCFA producers. Failing to orchestrate this microbial programming event led to pathological inflammatory disorders.^58^

The potential limitation of this study is the small sample size at early timepoint (3 weeks) which preclude us from investigating the effect of other early-life confounding factors such as diet or lifestyle factors on these findings. To overcome the possibility of a type 1 error from small sample size, statistical power was increased by bootstrapping method whereas sensitivity test was performed under 2 extreme scenarios to assess the influence of missing samples. The statistical significance for key variables remained unchanged. However, caution should also be exercised in interpreting the findings as they are not necessarily representative of AE in other geographical settings and replication is required. Moreover, as with previous cohort studies,^9–12^ SCFAs were quantified based on per mg of wet stool due to the high volatility of the SCFAs. Differential stool consistency between samples may however be a potential confounding factor. It is proposed that stool consistency should be taken into account in the design of future studies. For example, the dry weight of an equal aliquot of each stool sample could be incorporated to standardize measurement. Despite the small cohort size, our study is the first integrated multi-omics study in the field of allergy including atopic eczema. This strategy elucidates the cross-talk between host-microbiota and their metabolites, and therefore provides comprehensive multi-omics insights into unraveling the microbial pathways underlying the pathogenesis of eczema in early life.

In summary, we described key early life gut microbiome and metabolomic signatures that are associated with AE in a Singaporean mother-offspring cohort. Taken together, our data depict a disrupted developmental trajectory of the gut microbiome that commences in the first weeks of life. We identified microbiome and metabolome risk phenotypes for AE that occurs in the first three months of life. A compromised microbiome in the first 100 days of life has been identified as a risk factor for allergy.^10,12,59^ Our findings provide key directions for future mechanistic studies and targets for intervention strategies to optimize the influential role of the maturing gut microbiome on the programming of the immune system for the prevention of eczema in early life.

## Methods

### Subjects recruitment and sample collection

GUSTO is an ongoing mother-offspring cohort study in Singapore which recruited 1237 pregnant women from the general population irrespective of atopy and followed their offspring prospectively. The primary objective of GUSTO is to identify and evaluate the role of risk factors and determinants influencing body composition and metabolism during early development, and their influence on infants’ health in later childhood. The study methodology has been described in detail previously.^60^ Standardized questionnaires, including the modified ISAAC questionnaire previously validated in other epidemiological studies for assessment of allergic outcomes,^61,62,63^ were administered by trained interviewers at birth, 3 weeks, 3, 6, 9, 12, 15, 18, 24 and 36 months. Information on demographics, family history of allergy, social and lifestyle factors, such as diet and breastfeeding, were collected. Outcomes were classified as absent when the answers for all visits up to 36 months were “no”. Family history of allergy was defined as positive if the mother, father or an older sibling ever had atopic eczema, asthma or allergic rhinitis. A physician’s diagnosis of eczema in the child was determined by a positive answer to the written question: “Has your child ever been diagnosed with eczema?”. SCORAD (SCORing of Atopic Dermatitis (AD) severity) assessments were performed by trained physicians at 18 and 36 months for evaluation of eczema severity.

Allergen sensitization was assessed through skin prick testing (SPT) to aeroallergens (house dust mites *Dermatophagoides pteronyssinus, Dermatophagoides farinae*, and *Blomia tropicalis*) and to food allergens (egg, peanut and cow’s milk) at the 18- and 36-month visits. These are the most common aeroallergens and food allergens in sensitized Singaporean children.^64^ All skin prick extracts were obtained from Greer Laboratories (Lenoir, NC, USA), except for *B. tropicalis*, which was obtained from our in-house laboratory. *B. tropicalis* extract was prepared as previously described.^65^ A wheal of at least 3 mm was defined as a positive SPT and a child was considered as SPT-positive (allergen-sensitized) if any one or more of the individual tests were positive with a positive reaction to histamine (positive control) and negative reaction to saline (negative control).

Other potential allergic co-morbidities (wheezing, rhinitis) were also assessed at 18 and 36 months. Rhinitis was defined as having symptoms of sneezing, runny and/or blocked nose that lasted for at least four weeks in single or multiple episodes (each episode lasting at least two weeks). This definition was based on the Allergic Rhinitis and its Impact on Asthma (ARIA) guidelines.^66^ Wheezing was defined as the presence of wheeze symptoms (noisy breathing with a high-pitched, whistling sound heard from the chest, not the mouth) and with the use of nebulizer. Food allergy was defined as a convincing history of an IgE mediated reaction to a food allergen and a positive SPT to the specified food allergen.

A sub-cohort of 63 participants who had clinical data from birth up to the 36-month time-point, and stool samples collected at 3 weeks, 3, 6 and 12 months, were analyzed in this study. Thirty-three participants with physician-diagnosed eczema were further classified into non-allergen sensitized atopic eczema (NAE) (n = 14) and allergen-sensitized atopic eczema (AE) (n = 19). Atopic eczema cases were selected with similar characteristics as 30 non-eczema controls by age, mode of delivery, breastfeeding pattern till 6 months, use of antibiotic at labor and postnatal antibiotics to minimize potential selection bias. (Supplementary Figure 7). Ethics approval was obtained from the Domain Specific Review Board of Singapore, National Healthcare Group and the Centralized Institutional Review Board of SingHealth (DSRB D/09/021 and CIRB 2009/280/D). Informed written consent was obtained from all subjects.

For metagenomics sequencing and metabolomics profiling, stool samples were collected by the parents using sterile feces containers and stored at −20°C at home. For metatranscriptomic sequencing, stool samples were additionally collected directly into containers with RNAlater Stabilization Solution (Thermo Fisher Scientific, Waltham, Massachusetts) and stored at 4°C at home. Both samples were then transported to the lab in cold chain within 20 hours of sample collection for processing. After processing, sample were stored at −80°C until further analysis. A total of 162 stool samples were collected from subjects of the three clinical groups over four timepoints which were all then subjected to SCFAs and untargetted metabolomic profiling and metagenomics sequencing. Excluding samples with low RNA yield and low read counts, 91 stool samples eventually underwent metatranscriptomic sequencing (technique summary in Supplementary Table 7).

### Profiling of Stool SCFAs using LC/MS/MS and Untargeted Stool Metabolite Profiling by GC/TOFMS

For targeted analysis of SCFAs, details of chemicals and reagents sources, sample and calibration standards preparation were performed as reported previously which was shown to able to detect a total of 9 SCFAs including 6 common SCFAs (acetate, propionate, butyrate, isobutyrate, isovalerate and caproate) and 3 uncommon SCFAs (valerate, 2-methylbutyrate and 4-methylvalerate).^67^ For global profiling of stool metabolites, samples were randomized then processed according to our in-house protocol. Briefly, lyophilized stool sample (80 mg) was ultrasonicated with 1 mL of ice-cold extraction solvent (methanol:water [8:2]) containing 1 µg/mL d_27_-myristic acid as an internal standard at 4°C in a bath ultrasonicator (Elma Transsonic 460, Germany) for 30 min, and vortex-mixed for 2 min. The samples were then centrifuged at 18,000 *g* for 20 min at 4°C, and 0.5 mL of the supernatant was extracted carefully followed by drying at 50°C under a gentle stream of nitrogen gas (Turbovap LV, Caliper Life Sciences, Hopkinton, MA, USA). 100 μL of toluene (kept anhydrous with sodium sulfate) was added to the dry residue and evaporated completely again at 50°C under nitrogen gas to remove traces of water. The dried metabolic extract was then oximated with 50 μL of MOX (20 mg/mL) at 60°C for 2 h. Following centrifugation, 100 μL of MSTFA with 1% TMCS was added and the mixture was incubated at 60°C for 45 min to form trimethylsilyl (TMS) derivatives. Finally, 100 μL of the TMS derivatives was transferred into a GC vial and subjected to gas chromatography/time-of-flight mass spectrometry (GC/TOFMS) analysis. 50 µL of each stool extract prepared during extraction of lyophilized stool were also pooled to prepare quality control (QC) samples.

Analysis was performed on a Pegasus GC/TOFMS (LecoCorp, St. Joseph, MI, USA) coupled to an Agilent 7890 gas chromatograph (Agilent Technologies, Santa Clara, California, USA). A DB-1 (30 m × 250 μm i.d.) fused silica capillary column (Agilent J&W Scientific, Folsom, CA), with 0.25 μm film thickness, was used with open split interface. Helium was employed as the carrier gas at a constant flow rate of 1.5 mL/min and the injector split ratio was adjusted to 1:10. An injection volume of 1 μL was used. The injector, transfer line and ion source temperatures were maintained at 220, 280 and 250°C, respectively. The oven temperature was programmed at 70°C for 0.2 min, increased at 9°C/min to 270°C where it was sustained for 5 min, and further increased at 40°C/min to 310°C where it was held for 11 min. The mass spectrometer was operated in the electron impact ionization mode at 70 eV. Data were acquired in full scan mode from *m/z* 40 to 600 with an acquisition rate of 20 spectra per second. To detect retention time shifts and facilitate Kovats retention index (RI) calculation, a standard alkane series mixture (C7-C30) was injected before every batch during sample analysis. This was accompanied by an injection of one pooled QC after every injection of 10 samples.

Gas chromatograms were subjected to baseline correction, smoothing, noise reduction, deconvolution, library matching and area calculation using ChromaTOF software (version 4.41, LecoCorp). Each peak was then manually checked for proper peak alignment and integration. Metabolites were assigned putative identities by comparing both the RI and the mass spectra with those available in the National Institute of Standards and Technology (NIST) library, Fiehn library and/or our in-house spectral library. The peak areas were subjected to quality control-based robust LOESS signal correction.^34^ Subsequently, the peak areas were normalized by the lyophilized weight of each stool sample. Thereafter, metabolites with coefficient of variation greater than 30% were removed from the dataset, and total area normalization was performed. List of all global metabolites identified are shown in Supplementary Table 8. All raw metabolomics data are available at Metabolomics Workbench data repository (http://www.metabolomicsworkbench.org),^68^ (Project Digital Object Identifier: 10.21228/M8N104; Project ID: PR000983; Study ID: ST001431, ST001432).

### Nucleic acid extraction and metagenomics sequencing

Approximately 100–150 mg of stool material from four serial stool samples (collected at week 3, months 3, 6 and 12) were used to extract genomic DNA with the ZR Fecal DNA MicroPrep Kit (Zymo Research, USA) according to the manufacturer’s instructions. For each stool sample, a sequencing library was first constructed using Illumina’s Truseq Nano DNA Library Preparation Kit (Illumina, San Diego, USA). The samples were sheared on a Covaris E220 to ~450 bp, following the manufacturer’s recommendation, and uniquely tagged with one of Illumina’s TruSeq HT DNA barcode combinations to enable sample pooling for sequencing.

The finished libraries were quantitated using Invitrogen’s Picogreen assay and the average library size was determined on Bioanalyzer 2100, using a DNA 7500 chip (Agilent). Library concentrations were then normalized to 4 nM and validated by qPCR on a ViiA-7 real-time thermocycler (Applied Biosystems), using qPCR primers recommended in Illumina’s qPCR protocol, and Illumina’s PhiX control library as standard. The libraries were then pooled at equimolar concentrations and sequenced on an Illumina HiSeq2500 sequencer in rapid mode at a read-length of 250 bp paired-end.

For metatranscriptomic sequencing, approximately 100–150 mg of stool material was used to extract total RNA with the Quick-RNA Fecal/Soil Microprep Kit (Zymo Research, USA) according to the manufacturer’s instructions and including the optional in-column DNase I digestion step to eliminate any traces of leftover DNA. For each stool sample, a sequencing library was first constructed using the Truseq Stranded mRNA Library Preparation Kit (Illumina, San Diego, USA) with the following modifications: The oligo-dT mRNA purification step was omitted and instead, 200ng of total RNA was directly added to the Elution2-Frag-Prime step. The PCR amplification step, which selectively enriches for library fragments that have adapters ligated at both ends, was performed according to the manufacturer’s recommendation but the number of amplification cycles was reduced to 12 instead of 15. Each library was uniquely tagged with one of Illumina’s TruSeq HT RNA barcode combinations to enable sample pooling for sequencing.

Finished libraries were quantitated using Promega’s QuantiFluor dsDNA assay and the average library size was determined on an Agilent Tapestation 4200. Library concentrations were then normalized to 4 nM and validated by qPCR on a QuantStudio-3 real-time PCR system (Applied Biosystems), using the Kapa library quantification kit for Illumina platforms (Kapa Biosystems). The libraries were then pooled at equimolar concentrations and sequenced on an Illumina HiSeq2500 sequencer in rapid mode at a read-length of 100bp paired-end.

### Bioinformatics

The metagenomics and metatranscriptomics Illumina reads were adapter-trimmed and quality-trimmed using cutadapt-1.8.1 with parameter of “-q 20 – trim-n – minimum-length 30 – match-read-wildcards”.^69^

The metagenomics dataset was mapped against the hg19 human reference genome with bowtie2 2.1.0 using “–very-sensitive-local” preset as its sensitivity parameter.^70^ Any reads that can’t be confidently mapped against the human genome, which were generated by “ – un-conc” switch, were separated and processed as the non-host reads. The non-host reads were then aligned against NCBI non-redundant protein database (downloaded on 3 January 2016) with Diamond version 0.8.5.^71^ Based on the resulting alignments, the microbial taxonomical classification was determined using the Lowest-Common-Ancestor (LCA) algorithm implemented in MEGAN6 version 6.4.19 (parameters: maxmatches = 25 minscore = 100 minsupport = 100).^72^ In addition, the functional classification was also determined with MEGAN6 against its internal KEGG database (gi2keggMarch2016.bin).

The metatranscriptomics dataset was mapped against GRCh38 reference genome using Hisat2 2.1.0 with default parameters.^73^ The unmapped reads, as generated with the “ – un-conc” option, were separated into putative ribosomal RNA and non-ribosomal RNA reads with sortMeRNA 2.1.^74^ The non-ribosomal reads were then assembled into transcript contigs with Trinity 2.6.5 and the samples’ abundance were estimated using the Salmon 0.9.1 package. To annotate the function of the transcripts, the contigs were aligned to NCBI non-redundant protein database (downloaded on 3 January 2016) with Diamond version 0.85.^71^ The microbial taxonomical classification was determined using the Lowest-Common-Ancestor (LCA) algorithm implemented in MEGAN6 version 6.4.19 (parameters: maxmatches = 25 minscore = 100 minsupport = 100). In addition, the functional classification was also determined with MEGAN6 against its internal KEGG database (gi2keggMarch2016.bin). Metagenomics abundance data was normalized against the smallest sample library size, while the metatranscriptomics abundance data was normalized using trimmed mean of M values (TMM) method implemented by Trinity.^75^ Both metagenomics and metatranscriptomics normalized taxonomical abundance and KEGG classification data was then cross-referenced to produce the table of microbial composition and their gene expression profile. The Shannon Diversity Index were calculated for each sample at genus level. All raw microbiome data are available at NCBI (https://www.ncbi.nlm.nih.gov) (Accession Number PRJNA642723).

### Statistical analysis

R Package “vegan” in the R version 3.4.1 within R studio version 1.0.136 was used to plot Principal Coordinates Analysis (PCoA) based on Bray-Curtis distance to visualize the separation of microbiome and metabolites profiles between groups.

All subsequent statistical analysis were carried out using IBM SPSS version 24.0 (IBM SPSS Statistics, Armonk, NY). Univariate analysis (Pearson chi-square test) was used to study demographic, lifestyle, and clinical factors between groups. Data (SCFA absolute concentrations, relative abundance of global metabolites, bacterial taxonomic metagenomics read counts and metagenomics/transcriptomics functional genes read counts) were subjected to log-transformation in order to normalize the data prior to subsequent analysis. The selection of confounding factors for subsequent analysis adjustment were based on two criteria: (1) known confounding factors that have been reported by other studies and (2) any other potential factors that show significant unequal distribution between groups by Chi-square test (*p* < .05). Exclusion of factors was based on the “backward exclusion” approach in linear mixed models analysis. Briefly, all relevant factors were first all included into the model for adjustment. Factors were then excluded if their *p*-value were > 0.9, indicating minimal or no effect on the model. Linear mixed model was used to evaluate the longitudinal mean difference of the normalized abundance data and assess the trend significance of the trajectories of eczema group (NAE/AE) compared to controls group (i.e., at the selected four timepoints of week 3, months 3, 6 and 12) among the three clinical groups, adjusted for seven potential confounders (i.e., gender, birth order, mode of delivery, breastfeeding till 6 months, antibiotics during labor, maternal and paternal atopic history). Three variance-covariance structures (compound symmetry, 1st order autoregressive and unstructured) were used for linear mixed model, and the selection of covariance structure was based on Akaike’s Information Criterion (AIC) and Schwarz’s Bayesian Criterion (BIC). General linear models were performed to compare the normalized abundance data between groups at each timepoint with Bonferroni correction for pairwise comparisons adjusting for baseline values and the 7 mentioned potential confounders. To increase statistical power in view of the relative small sample size, linear mixed model with bootstrapping (1000 iterations) was also performed to evaluate the trend significance of the trajectories. Spearman correlation was performed to assess the strength and direction (positive or negative) of the relationship between variables (SCFA absolute concentrations, relative abundance of global metabolites, bacterial taxonomic metagenomics read counts and metagenomics/transcriptomics functional genes read counts).

For identification of major butyrate- and propionate-producing bacterial families and species, the following criteria were used: (1) Demonstration of a positive Spearman correlation between the bacterial abundance in read count and absolute concentration of corresponding SCFAs (*p* < .05, Spearman correlation value ρ ≥ 0.65) and (2) Carriage of at least one genomic/transcriptomics functional gene from the butyrate and/or propionate metabolic pathway. (Supplementary Table 9A,9B).

All physiologically relevant global metabolites were grouped based on the KEGG database according to three main carbohydrate metabolic pathways: (1) “Glycolysis-associated intermediates” involving the upstream biosynthesis pathways of both butyrate and propionate which includes pre-glycolysis (starch and sucrose metabolism), glycolysis, para-glycolysis (citrate cycle – TCA, pentose phosphate metabolism) and post-glycolysis (pyruvate metabolism); (2) “Butyrate intermediates” and (3) “Propionate intermediates” (Supplementary Table 10).

All relevant functional genes carried by shortlisted species were grouped into “Glycolysis-associated Metabolism”, “Butyrate Metabolism” and “Propionate metabolism”. Based on KEGG database, “Glycolysis-associated Metabolism” includes functional gene from Glycolysis pathway (ko00010 Glycolysis/Gluconeogenesis), Pre-glycolysis pathway (ko00500 Starch and sucrose metabolism), Para-glycolysis pathway (ko00020 Citrate cycle – TCA cycle, ko00030 Pentose phosphate pathway) and Post-glycolysis pathway (ko00620 Pyruvate metabolism). “Butyrate Metabolism” includes gene from ko00650 Butanoate metabolism. “Propionate Metabolism” includes gene from ko00640 Propanoate metabolism (Supplementary Table 5).

Geometric means and standard deviation range (SD) were used to represent abundance data in log-scale. Abundance data were plotted in GraphPad Prism 7.0 (GraphPad Software, La Jolla, Calif). All statistical significance tests and confidence intervals (CIs) were 2-sided and set at a *P* value of less than 0.05. A sensitivity analysis involving two extreme “best” and “worst” cases analysis was conducted to test the stability of our model to account for missing data. In “best” scenarios, the missing value(s) were replaced with the highest values of data from other timepoints within that subject. Whereas in “worst” scenarios, the missing value(s) were replaced with the lowest value. The linear mixed model was then performed using the dataset generated under two extreme “best” and “worst” scenarios while adjusting for the same 7 confounding factors.

## Supplementary Material

Supplemental Material

## List of abbreviation

NAE: Non allergen-sensitized atopic eczema
AE: Allergen-sensitized atopic eczema
HMOs: Human milk oligosaccharides
GUSTO: Growing Up in Singapore Towards healthy Outcomes
SCORAD: SCORing of Atopic Dermatitis (AD) severity
SCFAs: Short-chain fatty acids
KEGG: Kyoto Encyclopedia of Genes and Genomes database
HMDB: Human Metabolome Database
SPT: Skin prick testing
LPS: Lipopolysaccharides
PSA: Polysaccharide A
