# Supplementary material for: A compromised developmental trajectory of the infant gut microbiome and metabolome in atopic eczema
Source: Gut Microbes. 2020 Oct 6;12(1):1801964. doi: 10.1080/19490976.2020.1801964 (PMC7553750; doi:10.1080/19490976.2020.1801964)
Supplement: Supplemental Material [file KGMI_A_1801964_SM8703.zip › Supplementary information/Supplementary Figures - Ta et al - Gut Microbes 170420.pdf]

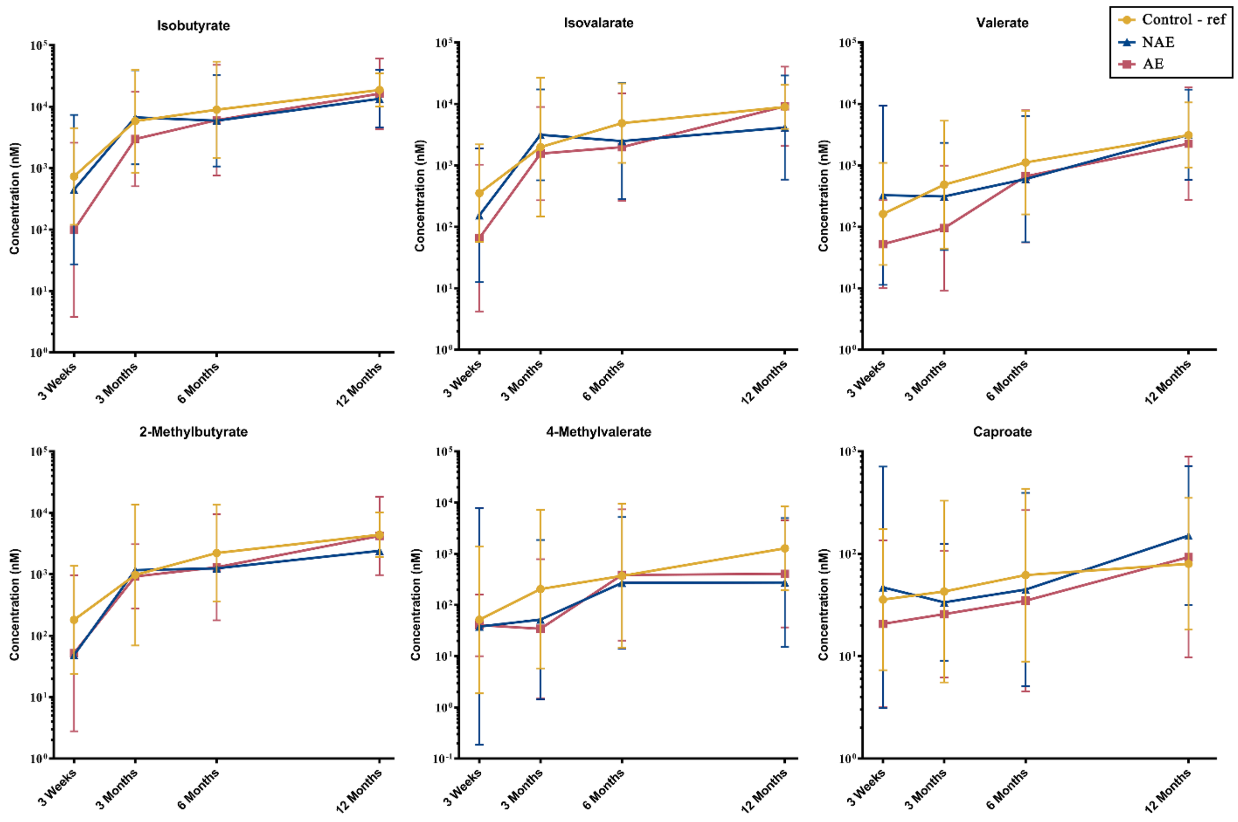

Supplementary Figure 1: Longitudinal comparison of 6 minor fecal short chain fatty acids by 3 clinical outcomes. Data are presented as the geometric mean and geometric standard deviation range of absolute concentration (nM) in log-scale. Different y-axis scale was used to highlight the difference of metabolites between groups. Linear mixed-model and general linear model were used to assess difference of abundance between the eczema (AE/NAE) and control (reference) groups adjusting for confounders (gender, birth order, mode of delivery, breastfeeding till 6 months, antibiotics at labour and family of atopic history). List of SCFAs and predominant metabolites and the comparison of abundance between eczema (NAE or AE) and control (reference) at individual timepoints are shown in **Supplementary Table 6a**. Sample size at 3 weeks - Control (n=13) vs NAE (n=5) vs AE (n=5); 3 months - Control (n=16) vs NAE (n=11) vs AE (n=10); 6 months - Control (n=27) vs NAE (n=9) vs AE (n=14) and 12 months - Control (n=26) vs NAE (n=8) vs AE (n=18).

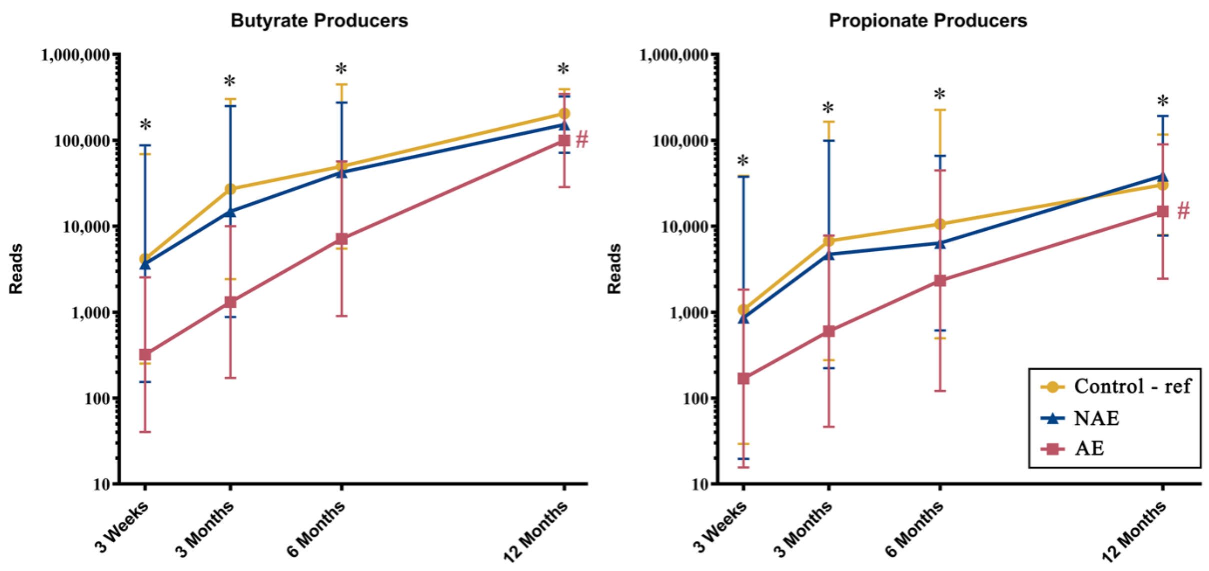

Supplementary Figure 2: Longitudinal maturation of butyrate and propionate producers at species level. "Butyrate producers" consists of *Bacteroides fragilis*, *Blautia producta*, *Blautia wexlerae*, *Eubacterium ramulus*, *Erysipelatoclostridium ramosum*, *Faecalibacterium prausnitzii*, *Lachnospiraceae bacterium*, *Tyzzerella nexilis and* and *Ruminococcus gnavus*. "Propionate producers" consists of *Anaerostipes caccae*, *Bacteroides fragilis*, *Blautia wexlerae*, *Eubacterium hallii*, *Eubacterium limosum* and *Ruminococcus sp. JC304*. Data are presented as the geometric mean and geometric standard deviation range of cumulative read counts in log-scale. Linear mixed-model and general linear model were used to assess difference of abundance between the eczema (AE/NAE) and control (reference) groups adjusting for confounders (gender, birth order, mode of delivery, breastfeeding till 6 months, antibiotics at labour and family of atopic history). Pairwise comparisons of abundance data at individual timepoints are listed in **Supplementary Table 1c**. *Significant difference at adj $P < 0.05$ between AE only and control group at specific time point. # (red) annotates significant longitudinal difference between AE and control at adj $P < 0.05$. Sample size at 3 weeks - Control (n=13) vs NAE (n=5) vs AE (n=5); 3 months - Control (n=16) vs NAE (n=11) vs AE (n=10); 6 months - Control (n=27) vs NAE (n=9) vs AE (n=14) and 12 months - Control (n=26) vs NAE (n=8) vs AE (n=18).

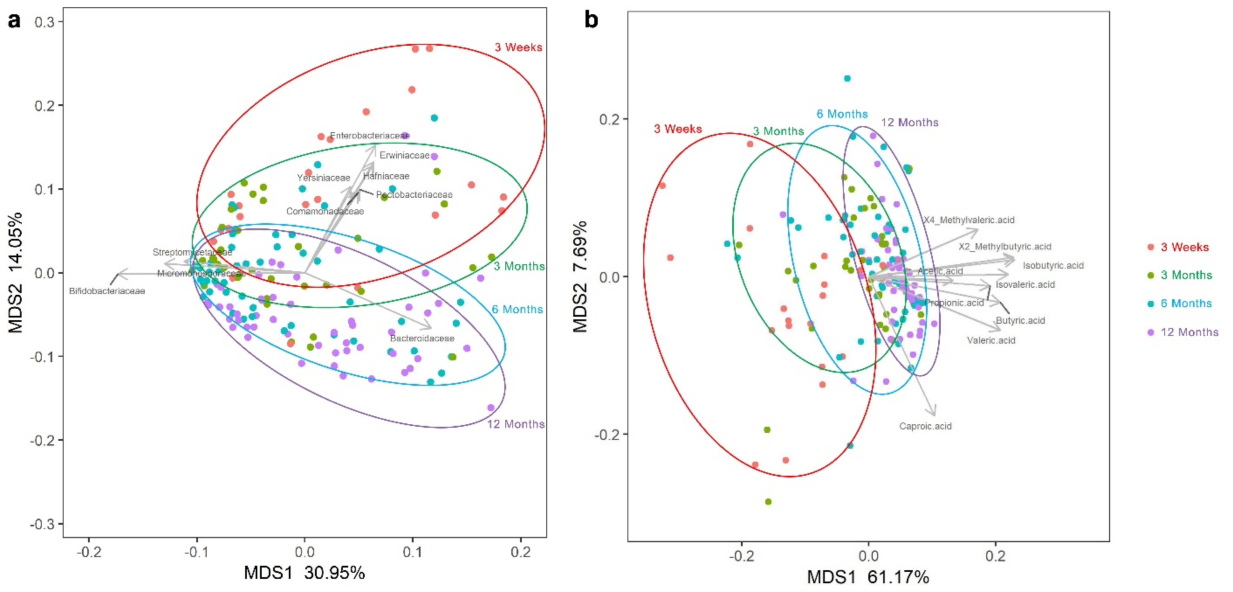

Supplementary Figure 3: Maturation of gut (A) microbiome and (B) metabolome depicted by PCoA based on Bray–Curtis dissimilarity between gut microbiome (family level) and metabolome (SCFAs) profiles of all 63 subjects at various time points. Arrow shows directions from the origin for which clusters have significant abundances for the bacterial or SCFA groups ($P < .001$, 999 permutations), and its length is proportional to the correlation between ordination and bacterial or SCFA groups.

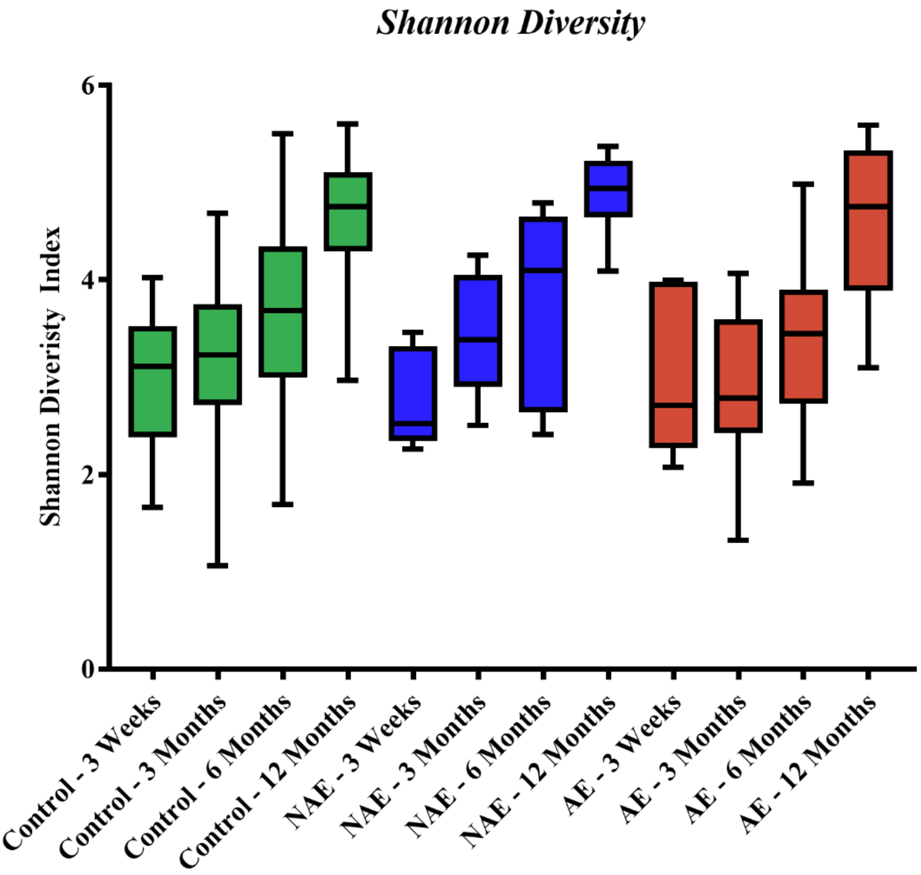

Supplementary Figure 4: Shannon diversity of the 3 clinical groups by time point. Boxplot 'boxes' indicate 25th percentile, median and 75th percentile of the data. Boxplot 'whiskers' indicate the minimum and maximum of the data.

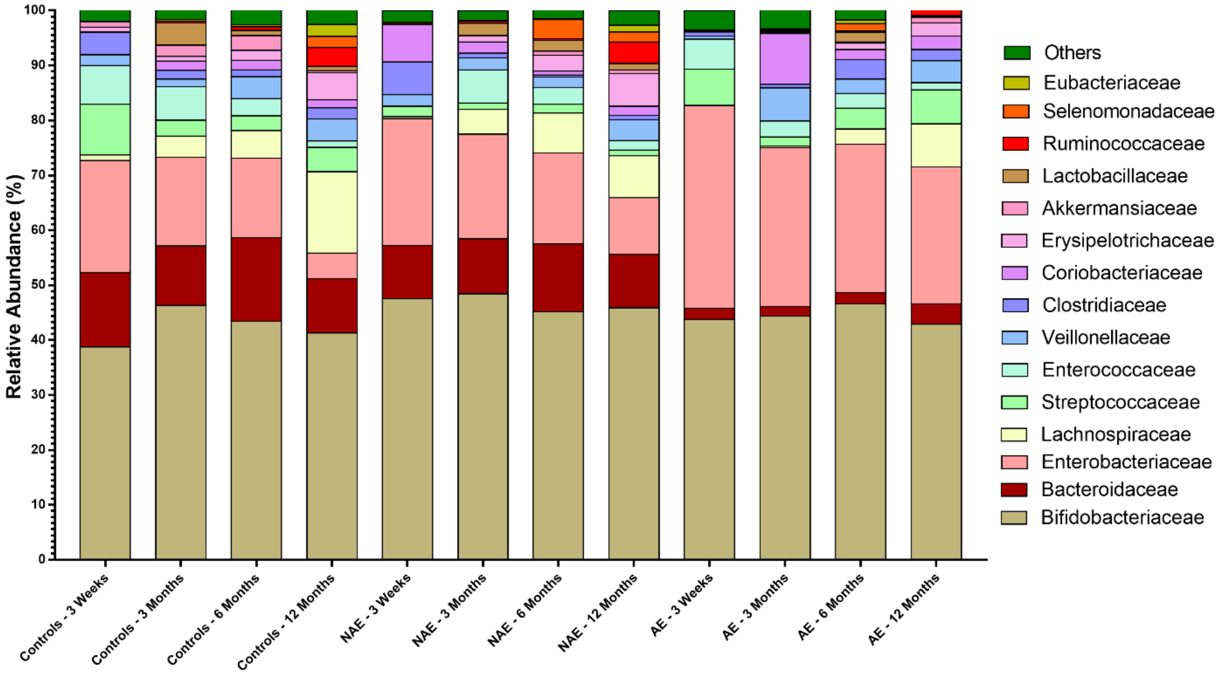

Supplementary Figure 5: Distribution of major families between groups over time. Data presented as mean of relative abundance (%)

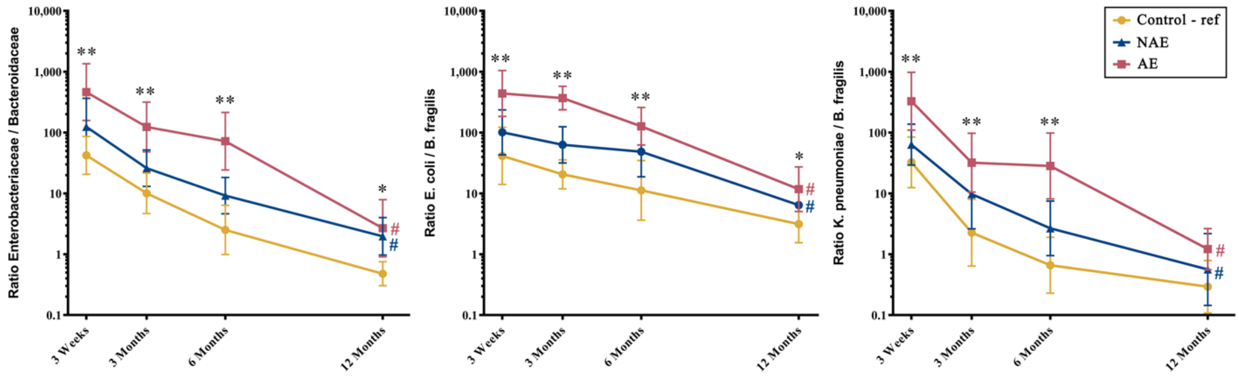

Supplementary Figure 6: Abundance ratio of *Enterobacteriaceae*/*Bacteroidaceae*, *E. coli/B. fragilis* and *K. pneumoniae*/*B. fragilis* in 3 clinical groups. Data are presented as abundance ratio of bacteria pairs and standard deviation range of reads ratio in log-scale. Linear mixed-model and general linear model were used to assess difference of abundance between the eczema (AE/NAE) and control (reference) groups adjusting for confounders (gender, birth order, mode of delivery, breastfeeding till 6 months, antibiotics at labour and family of atopic history). *Significant difference at adj P< 0.05 between AE only and control group at specific time point. **Significant difference at p < 0.05 between both AE and NAE compared to control groups at specific time point. # (blue) annotates significant longitudinal difference between NAE and control at adj P < 0.05. # (red) annotates significant longitudinal difference between AE and control at adj P < 0.05.Sample size at 3 weeks - Control (n=13) vs NAE (n=5) vs AE (n=5); 3 months - Control (n=16) vs NAE (n=11) vs AE (n=10); 6 months - Control (n=27) vs NAE (n=9) vs AE (n=14) and 12 months - Control (n=26) vs NAE (n=8) vs AE (n=18).

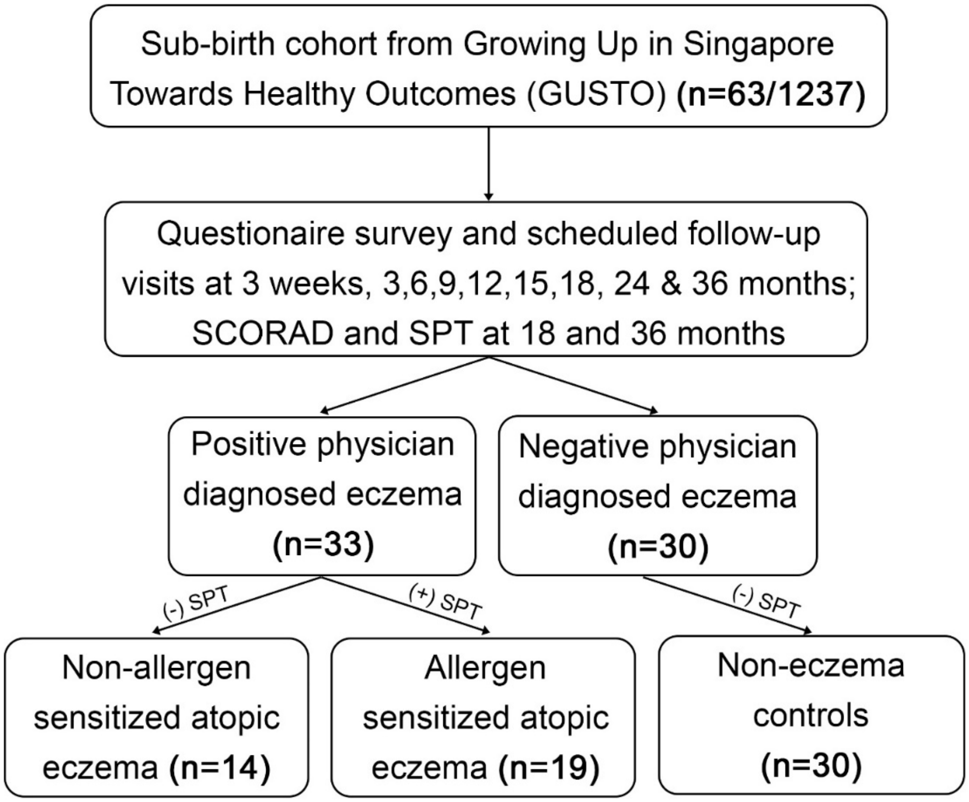

Supplementary Figure 7: A sub cohort of 63 subjects from the GUSTO cohort who completed clinical follow up from birth till 36 month old, and had stool samples collected at 3 weeks, 3, 6 and 12 months were analyzed in this study. Standardized questionnaires were administered at 3 monthly intervals from birth till 18 months, 24 and 36 months of age to capture demographic and clinical information including a physician's diagnosis of eczema. SCORAD for eczema severity assessments were performed by trained physicians at the 18 and 36 month time-point. Allergic sensitization was assessed through skin prick testing (SPT) to aeroallergens (house dust mites *Dermatophagoides pteronyssinus* and *Dermatophagoides farinae*, and *Blomia tropicalis*) and to food allergens (egg, peanut and cow's milk) which were performed at the 18 and 36 month visit. A wheal of at least 3 mm was defined as a positive SPT and a child was considered as SPT-positive (sensitized) if any one or more of the individual tests was positive with a positive reaction to histamine (positive control) and negative reaction to saline (negative control). Control cases were selected with similar characteristics as eczema cases by age (date of birth), mode of delivery, breastfeeding pattern till 6 months, use of antibiotic at labour and postnatal antibiotics to minimize potential selection bias.
