# Supplementary material for: A compromised developmental trajectory of the infant gut microbiome and metabolome in atopic eczema
Source: Gut Microbes. 2020 Oct 6;12(1):1801964. doi: 10.1080/19490976.2020.1801964 (PMC7553750; doi:10.1080/19490976.2020.1801964)
Supplement: Supplemental Material [file KGMI_A_1801964_SM8703.zip › Supplementary information/Supplementary Tables - Ta et al - Gut Microbes 010620.pdf]

Supplementary Table 1a: Longitudinal maturation of dominant bacteria families at metagenomics level

3 Weeks

	Phylum	Family	Control (n=13)			Non-Allergen Sensitized Atopic Eczema (NAE) (n=5)							Allergen Sensitized Atopic Eczema (AE) (n=5)						
			Geometric Mean (Reads)	Upper Range of Geometric SD	Lower Range of Geometric SD	Geometric Mean (Reads)	Upper Range of Geometric SD	Lower Range of Geometric SD	B Estimate[ψ]	95% CI Lower Bound[ψ]	95% CI Upper Bound[ψ]	Adj p value[ψ]	Geometric Mean (Reads)	Upper Range of Geometric SD	Lower Range of Geometric SD	B Estimate[ψ]	95% CI Lower Bound[ψ]	95% CI Upper Bound[ψ]	Adj p value[ψ]
**Early Colonizers**	*Bacteroidetes*	*Bacteroidaceae*	4,373.36	5,947.77	3,215.71	3,526.07	5,747.50	2,163.24	-3,791.17	-31,714.18	24,131.85	0.197	1,787.63	2,609.94	1,224.40	-6,204.70	-41,812.44	29,403.05	0.014*
	Proteobacteria	*Enterobacteriaceae*	97,891.35	134,111.20	71,453.54	116,554.82	230,778.50	58,866.07	19,365.90	-61,093.00	99,824.80	0.964	156,838.39	205,458.30	119,724.00	60,946.30	-89,353.47	211,246.06	0.043*
**Late Colonizers**	*Firmicutes*	*Lachnospiraceae*	168.19	248.93	113.64	324.00	476.28	220.41	-339.74	-2,496.39	1,816.91	0.475	41.39	73.40	23.34	-623.28	-6,321.51	5,074.94	0.288
	Firmicutes	*Eubacteriaceae*	12.62	14.76	10.78	15.57	20.24	11.97	-30.80	-440.08	378.47	0.874	7.82	8.75	6.98	-15.65	-547.70	516.41	0.951
	Firmicutes	*Ruminococcaceae*	639.85	742.22	551.59	92.40	168.17	50.77	-600.09	-1,419.04	218.87	0.031*	50.38	74.47	34.08	-1,137.07	-3,935.80	1,661.66	0.013*
	Firmicutes	*Erysipelotrichaceae*	801.82	1,495.86	429.79	270.82	493.29	148.68	-751.12	-1,824.24	322.01	0.413	303.71	486.02	189.79	-814.25	-2,810.17	1,181.67	0.029*

3 Months

	Phylum	Family	Control (n=16)			Non-Allergen Sensitized Atopic Eczema (NAE) (n=11)							Allergen Sensitized Atopic Eczema (AE) (n=10)						
			Geometric Mean (Reads)	Upper Range of Geometric SD	Lower Range of Geometric SD	Geometric Mean (Reads)	Upper Range of Geometric SD	Lower Range of Geometric SD	B Estimate[ψ]	95% CI Lower Bound[ψ]	95% CI Upper Bound[ψ]	Adj p value[ψ]	Geometric Mean (Reads)	Upper Range of Geometric SD	Lower Range of Geometric SD	B Estimate[ψ]	95% CI Lower Bound[ψ]	95% CI Upper Bound[ψ]	Adj p value[ψ]
**Early Colonizers**	*Bacteroidetes*	*Bacteroidaceae*	6,976.54	9,348.57	5,206.38	3,992.28	5,868.66	2,715.84	-3,806.31	-44,724.71	37,112.09	0.039*	2,050.83	3,055.74	1,376.40	-6,320.28	-33,309.31	20,668.74	0.027*
	Proteobacteria	*Enterobacteriaceae*	55,339.83	79,689.35	38,430.43	62,639.58	76,420.28	51,343.91	24,132.94	-33,010.00	81,275.88	0.691	91,699.01	134,797.50	62,380.28	48,586.32	-34,317.86	131,490.49	0.017*
**Late Colonizers**	*Firmicutes*	*Lachnospiraceae*	1,620.50	2,041.84	1,286.12	1,369.77	1,958.77	957.88	-1,570.68	-8,580.29	5,438.93	0.389	359.39	582.21	221.84	-2,012.91	-6,357.23	2,331.41	0.194
	Firmicutes	*Eubacteriaceae*	196.19	266.82	144.26	162.32	201.27	130.90	186.26	-2,000.19	2,372.72	0.975	124.16	158.93	97.00	661.61	-1,063.93	2,387.15	0.909
	Firmicutes	*Ruminococcaceae*	928.39	1,420.44	606.79	557.36	774.73	400.98	114.10	-1,528.93	1,757.14	0.208	143.45	243.86	84.38	-1,725.11	-2,663.03	-787.20	0.038*
	Firmicutes	*Erysipelotrichaceae*	3,887.79	6,472.26	2,335.34	4,433.23	7,944.82	2,473.76	990.14	-2,967.49	4,947.77	0.609	1,983.96	2,564.47	1,534.87	-1,541.76	-3,235.62	152.11	0.036*

6 Months

	Phylum	Family	Control (n=27)			Non-Allergen Sensitized Atopic Eczema (NAE) (n=9)							Allergen Sensitized Atopic Eczema (AE) (n=14)						
			Geometric Mean (Reads)	Upper Range of Geometric SD	Lower Range of Geometric SD	Geometric Mean (Reads)	Upper Range of Geometric SD	Lower Range of Geometric SD	B Estimate[ψ]	95% CI Lower Bound[ψ]	95% CI Upper Bound[ψ]	Adj p value[ψ]	Geometric Mean (Reads)	Upper Range of Geometric SD	Lower Range of Geometric SD	B Estimate[ψ]	95% CI Lower Bound[ψ]	95% CI Upper Bound[ψ]	Adj p value[ψ]
**Early Colonizers**	*Bacteroidetes*	*Bacteroidaceae*	10,093.70	17,663.97	5,767.83	4,160.52	5,783.13	2,993.18	-6,242.59	-34,909.22	22,424.05	0.028*	3,516.92	6,260.12	1,975.80	-8,221.19	-40,913.14	24,470.77	0.035*
	Proteobacteria	*Enterobacteriaceae*	47,973.56	71,480.61	32,197.02	55,653.71	68,454.07	45,246.92	9,271.03	-23,345.73	41,887.79	0.644	51,297.64	97,978.50	26,857.40	9,658.23	-39,349.74	58,666.21	0.119
**Late Colonizers**	*Firmicutes*	*Lachnospiraceae*	7,709.28	11,949.38	4,973.73	2,540.54	3,836.22	1,682.48	-5,806.31	-17,598.17	5,985.55	0.022*	2,334.95	3,082.14	1,768.91	-3,482.60	-15,168.68	8,203.48	0.045*
	Firmicutes	*Eubacteriaceae*	988.85	1,631.61	599.30	699.65	853.57	573.48	-1,224.26	-5,177.66	2,729.13	0.178	231.99	338.70	158.90	-1,542.06	-6,848.92	3,764.81	0.029*
	Firmicutes	*Ruminococcaceae*	5,036.54	8,813.94	2,878.02	2,036.00	2,606.08	1,590.62	-4,292.09	-12,358.43	3,774.25	0.043*	624.14	992.39	392.54	-6,056.44	-17,193.47	5,080.59	0.025*
	Firmicutes	*Erysipelotrichaceae*	20,089.66	30,426.62	13,264.52	23,150.28	34,881.20	15,364.59	1,339.21	-3,154.12	5,832.53	0.523	12,864.13	17,389.05	9,516.67	-6,096.44	-22,712.54	10,519.67	0.039*

12 Months

	Phylum	Family	Control (n=26)			Non-Allergen Sensitized Atopic Eczema (NAE) (n=8)							Allergen Sensitized Atopic Eczema (AE) (n=18)						
			Geometric Mean (Reads)	Upper Range of Geometric SD	Lower Range of Geometric SD	Geometric Mean (Reads)	Upper Range of Geometric SD	Lower Range of Geometric SD	B Estimate[ψ]	95% CI Lower Bound[ψ]	95% CI Upper Bound[ψ]	Adj p value[ψ]	Geometric Mean (Reads)	Upper Range of Geometric SD	Lower Range of Geometric SD	B Estimate[ψ]	95% CI Lower Bound[ψ]	95% CI Upper Bound[ψ]	Adj p value[ψ]
**Early Colonizers**	*Bacteroidetes*	*Bacteroidaceae*	6,149.94	9,286.41	4,072.81	4,712.16	8,670.38	2,560.96	-4,665.87	-52,275.21	42,943.48	0.032*	3,108.31	5,812.55	1,662.20	-5,454.06	-48,030.99	37,122.87	0.045*
	Proteobacteria	*Enterobacteriaceae*	19,969.76	29,754.94	13,402.52	9,242.90	14,881.07	5,740.93	-7,950.09	-53,679.00	37,778.83	0.438	18,451.12	22,694.88	15,000.91	2,796.47	-13,054.82	18,647.76	0.055
**Late Colonizers**	*Firmicutes*	*Lachnospiraceae*	51,906.45	63,844.93	42,200.36	38,118.82	50,316.84	28,877.89	-17,512.32	-83,277.26	48,252.62	0.032*	24,713.42	31,880.31	19,157.69	-26,988.66	-54,763.76	786.45	0.037*
	Firmicutes	*Eubacteriaceae*	9,905.34	12,282.62	7,988.18	5,113.73	7,210.36	3,626.76	-1,828.32	-7,217.90	3,561.26	0.047*	3,820.08	6,455.93	2,260.40	-3,499.85	-7,614.28	614.58	0.029*
	Firmicutes	*Ruminococcaceae*	102,761.18	142,838.00	73,928.90	64,657.24	80,174.98	52,142.94	-40,359.89	-98,726.21	18,006.43	0.026*	46,600.23	71,764.35	30,259.89	-61,959.44	-100,780.11	-23,138.76	0.047*
	Firmicutes	*Erysipelotrichaceae*	65,696.42	84,397.29	51,139.31	54,070.08	68,663.62	42,578.20	-7,395.55	-12,571.93	-2,219.17	0.914	48,732.65	83,783.32	28,345.39	-37,883.87	-53,461.29	-22,306.45	0.1

Trend Analysis

	Phylum	Family	Non-Allergen Sensitized Atopic Eczema (NAE)					Allergen Sensitized Atopic Eczema (AE)					
			Longitudinal Mean Difference (Reads) - B Estimate[φ]	95% CI Lower Bound[φ]	95% CI Upper Bound[φ]	Adj p value for the mean difference[φ]	Adj p value for the slope difference[φ]	Longitudinal Mean Difference (Reads) - B Estimate[φ]	95% CI Lower Bound[φ]	95% CI Upper Bound[φ]	Adj p value for the mean difference[φ]	Adj p value for the slope difference[φ]	Adj p value for time trend[φ]
**Early Colonizers**	*Bacteroidetes*	*Bacteroidaceae*	-38,224.76	-298,343.03	221,893.51	0.0362*	0.870	-56,806.33	-335,073.01	221,460.36	0.028*	0.008	0.115
	Proteobacteria	*Enterobacteriaceae*	24,133.97	-152,560.12	200,828.05	0.440	0.498	74,402.90	-32,881.18	181,686.98	0.016*	0.283	<0.001
**Late Colonizers**	*Firmicutes*	*Lachnospiraceae*	-14,215.68	-85,435.76	57,004.41	0.037*	0.632	-68,959.76	-156,175.13	18,255.61	0.018*	0.189	<0.001
	Firmicutes	*Eubacteriaceae*	-480.75	-1,382.60	421.11	0.036*	0.171	-1,846.71	-4,774.69	1,081.26	<0.001*	0.809	<0.001
	Firmicutes	*Ruminococcaceae*	-3,695.23	-8,332.16	941.71	0.035*	0.715	-8,001.35	-19,029.96	3,027.26	0.014*	0.170	0.001
	Firmicutes	*Erysipelotrichaceae*	-6,456.29	-16,225.03	3,312.45	0.159	0.837	-9,990.36	-31,792.84	11,812.12	0.035*	0.909	0.067

_ Data are presented as geometric mean and geometric standard deviation range for normalized bacterial read counts at family level & plotted in Figure 2a.

[ψ] General Linear models performed to compare the eczema group (NAE or AE) and controls (reference) at each time point with Bonferroni correction for pair wise comparisons adjusting for baseline values and the 7 mentioned potential confounders (i.e., gender, birth order, mode of delivery, breastfeeding till 6 months, antibiotics during labour, maternal and paternal atopic history).

[φ] Linear mixed model was used to evaluate normalized bacterial read counts longitudinally and assess the trend significance of the trajectories of eczema group (NAE/AE) compared to controls group (i.e., at the selected four timepoints of week 3, months 3, 6 and 12) among the three clinical groups, adjusted for seven potential confounders (i.e., gender, birth order, mode of delivery, breastfeeding till 6 months, antibiotics during labour, maternal and paternal atopic history).

*Significance at adj p <0.05 between eczema (AE/NAE) and control (reference).

Supplementary Table 1b : Longitudinal maturation of dominant bacteria species at metagenomics level

3 Weeks

	Phylum	Family	Species	Control (n=13)			Non-Allergen Sensitized Atopic Eczema (NAE) (n=5)							Allergen Sensitized Atopic Eczema (AE) (n=5)						
				Geometric Mean (Reads)	Upper Range of Geometric SD	Lower Range of Geometric SD	Geometric Mean (Reads)	Upper Range of Geometric SD	Lower Range of Geometric SD	B Estimate [ɸ]	95% CI Lower Bound [ɸ]	95% CI Upper Bound [ɸ]	Adj p value [ɸ]	Geometric Mean (Reads)	Upper Range of Geometric SD	Lower Range of Geometric SD	B Estimate [ɸ]	95% CI Lower Bound [ɸ]	95% CI Upper Bound [ɸ]	Adj p value [ɸ]
**Early Colonizers**	*Bacteroidetes*	*Bacteroidaceae*	*Bacteroides fragilis*	598.28	137.75	2,598.40	525.54	78.83	3,503.61	-484.17	-4,100.05	3,131.71	0.984	109.44	24.29	493.06	-1,241.38	-4,060.58	1,577.83	0.017*
	Proteobacteria	*Enterobacteriaceae*	*Escherichia coli*	48,135.43	13,973.04	165,820.78	53,050.81	15,156.66	185,686.64	23,005.85	-111,365.52	157,377.23	0.669	82,323.78	22,197.84	305,309.18	61,537.49	-161,545.30	284,620.27	0.028*
	Proteobacteria	*Enterobacteriaceae*	*Klebsiella pneumoniae*	17,066.51	4,375.69	66,564.51	37,982.48	11,830.71	121,942.75	19,512.28	-23,284.10	62,308.67	0.038*	35,952.05	9,677.44	133,563.27	21,821.07	-28,942.30	72,584.44	0.019*
**Late Colonizers**	*Firmicutes*	*Lachnospiraceae*	*Anaerostipes caccae*	6.90	1.31	36.39	4.33	0.84	22.46	-10.85	-61.52	39.82	0.995	5.28	1.26	30.25	28.36	-34.30	91.01	0.852
	Firmicutes	*Lachnospiraceae*	*Blautia producta*	10.76	0.89	129.90	11.50	1.94	68.29	-347.71	-1,322.69	627.27	0.822	2.67	1.48	4.82	125.04	-1,080.55	1,330.62	0.964
	Firmicutes	*Lachnospiraceae*	*Blautia wexlerae*	8.46	2.88	24.86	9.63	1.91	48.40	3.59	-16.49	23.67	0.979	8.80	4.66	16.59	7.75	-17.08	32.58	0.734
	Firmicutes	*Lachnospiraceae*	*Lachnospiraceae bacterium*	2.19	0.70	6.83	18.44	0.63	536.16	275.84	-766.41	1,318.10	0.641	1.19	0.84	1.68	797.45	-491.33	2,086.23	0.622
	Firmicutes	*Lachnospiraceae*	*Tyzzerella nexilis*	41.14	10.15	166.75	72.00	15.87	326.71	58.01	-110.34	226.37	0.387	9.46	3.20	27.99	48.02	-160.15	256.19	0.754
	Firmicutes	*Eubacteriaceae*	*Eubacterium limosum*	6.41	2.93	14.01	5.89	1.22	28.44	-26.23	-92.67	40.22	0.747	6.52	3.15	13.47	39.16	-43.00	121.33	0.483
	Firmicutes	*Eubacteriaceae*	*Eubacterium hallii*	2.57	0.93	7.11	6.51	0.75	56.38	16.98	-4.71	38.66	0.964	0.00	0.00	0.00	10.32	-16.50	37.13	0.626
	Firmicutes	*Eubacteriaceae*	*Eubacterium ramulus*	4.20	2.00	8.82	4.76	2.30	9.84	1.58	-4.85	8.02	0.883	1.73	0.92	3.27	-0.61	-8.57	7.35	0.677
	Firmicutes	*Ruminococcaceae*	*Faecalibacterium prausnitzii*	20.97	5.41	81.30	24.50	6.99	85.85	20.50	-31.97	72.98	0.170	8.36	1.84	38.11	20.78	-44.10	85.67	0.204
	Firmicutes	*Ruminococcaceae*	*Ruminococcus gnavus*	54.59	24.54	121.47	44.69	19.23	127.88	333.11	-886.42	1,552.64	0.510	29.51	19.67	44.29	222.90	-748.62	1,194.41	0.985
	Firmicutes	*Ruminococcaceae*	*Ruminococcus sp. JC304*	2.80	1.06	7.38	4.90	1.32	18.12	3.09	-5.06	11.23	0.782	2.83	1.52	5.25	6.32	-3.75	16.40	0.747
	Firmicutes	*Erysipelotrichaceae*	*Erysipelatoclostridium ramosum*	30.72	45.04	20.08	9.30	14.51	5.96	-117.77	-562.14	326.61	0.397	7.58	10.31	5.58	40.344	-105.849	186.54	0.978

3 Months

	Phylum	Family	Species	Control (n=16)			Non-Allergen Sensitized Atopic Eczema (NAE) (n=11)							Allergen Sensitized Atopic Eczema (AE) (n=10)						
				Geometric Mean (Reads)	Upper Range of Geometric SD	Lower Range of Geometric SD	Geometric Mean (Reads)	Upper Range of Geometric SD	Lower Range of Geometric SD	B Estimate [ɸ]	95% CI Lower Bound [ɸ]	95% CI Upper Bound [ɸ]	Adj p value [ɸ]	Geometric Mean (Reads)	Upper Range of Geometric SD	Lower Range of Geometric SD	B Estimate [ɸ]	95% CI Lower Bound [ɸ]	95% CI Upper Bound [ɸ]	Adj p value [ɸ]
**Early Colonizers**	*Bacteroidetes*	*Bacteroidaceae*	*Bacteroides fragilis*	1,999.85	393.44	10,165.26	596.16	112.57	3,157.15	-1,577.87	-10,049.89	6,894.15	0.035*	163.07	123.13	215.98	-1,318.70	-10,451.74	7,814.34	0.017*
	Proteobacteria	*Enterobacteriaceae*	*Escherichia coli*	20,708.32	6,672.62	64,267.75	37,518.33	10,586.62	132,962.71	17,027.86	-55,469.02	89,524.74	0.025	63,021.39	29,423.99	134,981.54	47,217.16	-25,778.05	120,212.37	0.036*
	Proteobacteria	*Enterobacteriaceae*	*Klebsiella pneumoniae*	5,806.31	2,199.06	15,330.76	4,521.24	1,505.32	13,579.57	1,519.96	-12,990.93	16,030.84	0.781	5,212.12	1,525.65	17,806.31	3,288.64	-9,079.02	15,656.30	0.785
**Late Colonizers**	*Firmicutes*	*Lachnospiraceae*	*Anaerostipes caccae*	18.18	1.54	215.18	29.49	1.69	514.48	35.07	-212.90	283.03	0.588	9.77	1.21	79.18	37.69	-764.24	839.62	0.547
	Firmicutes	*Lachnospiraceae*	*Blautia producta*	107.19	12.29	934.48	44.32	1.38	1,422.56	-793.82	-1,577.23	-10.41	0.523	12.31	3.10	48.89	-803.37	-3,155.30	1,548.57	0.117
	Firmicutes	*Lachnospiraceae*	*Blautia wexlerae*	63.34	3.06	1,311.97	23.39	2.57	212.95	-943.81	-1,785.38	-102.25	0.423	16.17	2.68	97.54	-803.37	-1,589.55	-17.18	0.125
	Firmicutes	*Lachnospiraceae*	*Lachnospiraceae bacterium*	16.86	1.09	261.61	21.67	0.75	622.66	230.77	-738.07	1,199.61	0.295	3.54	0.59	21.10	-702.90	-1,847.68	441.88	0.083
	Firmicutes	*Lachnospiraceae*	*Tyzzerella nexilis*	199.75	9.65	4,134.33	247.97	17.68	3,477.27	-428.79	-2,757.24	1,899.65	0.640	74.67	7.79	715.67	-713.01	-2,442.35	1,016.34	0.422
	Firmicutes	*Eubacteriaceae*	*Eubacterium limosum*	62.89	1.89	2,088.42	28.84	1.95	426.14	-183.34	-1,242.77	876.10	0.859	27.48	1.87	403.42	874.10	-1,584.89	3,333.10	0.474
	Firmicutes	*Eubacteriaceae*	*Eubacterium hallii*	7.81	1.09	55.97	24.70	2.58	236.33	43.25	-16.39	102.90	0.671	1.93	0.72	5.22	-35.52	-91.93	20.89	0.280
	Firmicutes	*Eubacteriaceae*	*Eubacterium ramulus*	57.72	2.61	1,277.45	50.54	1.81	1,414.56	-365.90	-1,997.67	1,265.88	0.538	6.32	0.80	49.67	-388.49	-1,768.80	991.82	0.155
	Firmicutes	*Ruminococcaceae*	*Faecalibacterium prausnitzii*	48.20	11.88	195.55	46.93	14.17	155.39	-29.80	-143.39	83.80	0.821	40.02	12.67	126.42	-57.42	-164.84	50.01	0.715
	Firmicutes	*Ruminococcaceae*	*Ruminococcus gnavus*	636.61	20.62	19,656.84	229.60	13.36	3,944.83	-694.55	-1,509.58	120.48	0.112	73.51	21.47	251.63	-984.52	-1,702.89	-266.14	0.026*
	Firmicutes	*Ruminococcaceae*	*Ruminococcus sp. JC304*	11.89	0.81	175.17	10.63	1.90	59.63	-1,023.77	-2,869.06	821.51	0.759	4.75	2.74	8.22	-124.40	-869.50	620.70	0.042*
	Firmicutes	*Erysipelotrichaceae*	*Erysipelatoclostridium ramosum*	45.17	75.88	26.89	25.17	41.04	15.44	171.48	-177.84	520.79	0.850	15.12	42.84	28.79	-195.41	-1,944.32	1,553.50	0.048*

6 Months

	Phylum	Family	Species	Control (n=27)			Non-Allergen Sensitized Atopic Eczema (NAE) (n=9)							Allergen Sensitized Atopic Eczema (AE) (n=14)						
				Geometric Mean (Reads)	Upper Range of Geometric SD	Lower Range of Geometric SD	Geometric Mean (Reads)	Upper Range of Geometric SD	Lower Range of Geometric SD	B Estimate [ɸ]	95% CI Lower Bound [ɸ]	95% CI Upper Bound [ɸ]	Adj p value [ɸ]	Geometric Mean (Reads)	Upper Range of Geometric SD	Lower Range of Geometric SD	B Estimate [ɸ]	95% CI Lower Bound [ɸ]	95% CI Upper Bound [ɸ]	Adj p value [ɸ]
**Early Colonizers**	*Bacteroidetes*	*Bacteroidaceae*	*Bacteroides fragilis*	4,123.53	863.10	19,700.59	818.73	364.52	1,838.92	-3,291.22	-15,037.12	8,454.69	0.049*	111.39	31.87	389.32	-19,502.78	-33,853.43	-5,152.13	0.047*
	Proteobacteria	*Enterobacteriaceae*	*Escherichia coli*	39,746.74	15,265.57	103,488.03	41,121.79	9,885.47	171,059.33	7,800.53	-38,412.82	54,013.88	0.944	46,396.83	20,494.61	105,035.70	6,390.92	-48,654.16	61,435.99	0.839
	Proteobacteria	*Enterobacteriaceae*	*Klebsiella pneumoniae*	2,184.03	548.50	8,696.40	2,720.83	620.66	11,927.47	3,176.33	-30,901.66	37,254.32	0.913	3,158.94	1,154.12	8,646.31	5,319.22	-13,270.33	23,908.77	0.760
**Late Colonizers**	*Firmicutes*	*Lachnospiraceae*	*Anaerostipes caccae*	152.87	13.25	1,763.69	102.30	2.17	4,815.44	186.18	-936.14	1,308.50	0.414	83.91	6.92	1,018.19	-169.51	-668.74	329.73	0.956
	Firmicutes	*Lachnospiraceae*	*Blautia producta*	279.22	29.92	2,605.91	152.72	8.35	2,793.36	-1,043.83	-3,067.83	980.18	0.183	165.66	13.54	2,026.83	-955.79	-6,606.96	4,695.37	0.809
	Firmicutes	*Lachnospiraceae*	*Blautia wexlerae*	215.29	16.10	2,878.56	62.76	3.58	1,098.97	-1,093.81	-8,720.60	6,532.98	0.371	29.34	4.86	176.97	-1,281.74	-2,035.97	-527.51	0.035*
	Firmicutes	*Lachnospiraceae*	*Lachnospiraceae bacterium*	150.00	10.49	2,145.81	74.60	1.29	4,311.74	117.32	-3,528.71	3,763.34	0.488	23.35	1.02	536.82	-944.06	-5,680.76	3,792.65	0.706
	Firmicutes	*Lachnospiraceae*	*Tyzzerella nexilis*	975.55	68.58	13,877.15	440.38	25.18	7,701.50	-1,130.56	-3,662.88	1,401.76	0.684	219.50	27.63	1,743.62	-1,475.88	-3,023.47	71.71	0.041*
	Firmicutes	*Eubacteriaceae*	*Eubacterium limosum*	632.51	11.89	33,644.76	46.53	3.92	551.82	-1,759.55	-3,542.99	23.89	0.007*	11.92	2.86	49.62	-1,168.46	-2,571.24	234.33	0.009*
	Firmicutes	*Eubacteriaceae*	*Eubacterium hallii*	12.20	1.50	99.25	15.29	1.66	140.51	-18.56	-203.17	166.04	0.913	6.95	1.89	25.50	-122.19	-277.06	32.69	0.706
	Firmicutes	*Eubacteriaceae*	*Eubacterium ramulus*	232.79	10.84	4,999.98	117.62	2.76	5,012.41	-406.52	-1,876.80	1,063.76	0.590	43.60	2.19	867.11	-1,304.20	-3,893.46	1,285.07	0.035*
	Firmicutes	*Ruminococcaceae*	*Faecalibacterium prausnitzii*	127.69	16.66	978.50	48.65	17.16	137.94	-430.09	-1,474.57	614.40	0.237	60.76	21.93	168.35	-720.92	-2,082.85	641.02	0.748
	Firmicutes	*Ruminococcaceae*	*Ruminococcus gnavus*	3,199.41	148.65	68,859.26	1,410.91	20.15	98,785.75	-1,669.04	-2,614.34	-723.73	0.611	378.92	17.64	8,141.69	-2,610.44	-4,607.98	-612.90	0.036*
	Firmicutes	*Ruminococcaceae*	*Ruminococcus sp. JC304*	30.80	2.82	336.74	30.67	2.44	384.77	-908.04	-3,861.07	2,044.98	0.983	4.24	1.47	12.20	-279.14	-756.56	198.28	0.023*
	Firmicutes	*Erysipelotrichaceae*	*Erysipelatoclostridium ramosum*	565.18	921.24	346.74	233.31	286.97	189.68	-536.41	-1,147.49	74.67	0.036*	279.16	443.87	175.57	-954.85	-1,961.22	51.53	0.031*

12 Months

	Phylum	Family	Species	Control (n=26)			Non-Allergen Sensitized Atopic Eczema (NAE) (n=8)							Allergen Sensitized Atopic Eczema (AE) (n=18)						
				Geometric Mean (Reads)	Upper Range of Geometric SD	Lower Range of Geometric SD	Geometric Mean (Reads)	Upper Range of Geometric SD	Lower Range of Geometric SD	B Estimate [ɸ]	95% CI Lower Bound [ɸ]	95% CI Upper Bound [ɸ]	Adj p value [ɸ]	Geometric Mean (Reads)	Upper Range of Geometric SD	Lower Range of Geometric SD	B Estimate [ɸ]	95% CI Lower Bound [ɸ]	95% CI Upper Bound [ɸ]	Adj p value [ɸ]
**Early Colonizers**	*Bacteroidetes*	*Bacteroidaceae*	*Bacteroides fragilis*	2,604.82	908.27	7,470.34	2,174.52	1,779.22	2,657.66	157.21	-16,470.42	16,784.84	0.740	1,070.19	489.69	2,338.83	-1,208.98	-13,536.71	11,118.76	0.033*
	Proteobacteria	*Enterobacteriaceae*	*Escherichia coli*	8,139.00	2,560.34	25,872.85	12,573.51	10,770.99	14,677.69	4,904.35	-33,194.65	43,003.34	0.368	13,976.20	4,926.10	39,652.87	5,870.73	-13,980.38	25,721.84	0.701
	Proteobacteria	*Enterobacteriaceae*	*Klebsiella pneumoniae*	1,222.47	381.50	3,917.20	763.08	205.23	2,837.24	2,125.20	-13,762.20	18,012.60	0.632	1,299.20	815.40	2,070.05	1,001.82	-4,455.92	6,459.57	0.537
**Late Colonizers**	*Firmicutes*	*Lachnospiraceae*	*Anaerostipes caccae*	1,236.17	369.53	4,135.26	981.99	290.91	3,314.79	915.07	-1,963.48	3,793.62	0.316	811.14	103.92	811.14	-948.27	-1,305.04	-591.50	0.037*
	Firmicutes	*Lachnospiraceae*	*Blautia producta*	2,327.38	467.77	11,579.77	1,791.01	334.41	9,592.12	-881.83	-4,549.01	2,785.36	0.774	1,522.10	199.70	1,522.10	-1,952.04	-3,845.26	-58.82	0.047*
	Firmicutes	*Lachnospiraceae*	*Blautia wexlerae*	4,753.94	650.64	34,735.11	3,797.76	265.14	54,396.93	-1,275.57	-3,590.83	1,039.69	0.749	955.73	88.39	955.73	-2,814.05	-4,841.61	-786.48	0.026*
	Firmicutes	*Lachnospiraceae*	*Lachnospiraceae bacterium*	1,917.17	471.40	7,797.06	1,187.17	306.38	4,600.03	400.52	-3,414.56	4,215.59	0.746	764.12	156.86	764.12	-1,821.77	-3,156.56	-486.97	0.017*
	Firmicutes	*Lachnospiraceae*	*Tyzzerella nexilis*	8,956.57	1,324.36	60,572.75	4,485.29	675.85	29,766.67	-5,723.34	-14,334.07	2,887.40	0.036*	6,350.52	1,567.62	6,350.52	-6,377.14	-13,773.44	1,019.15	0.038*
	Firmicutes	*Eubacteriaceae*	*Eubacterium limosum*	597.69	30.99	11,527.73	302.79	18.61	4,926.36	-1,010.61	-3,036.20	1,014.98	0.360	58.77	5.51	58.77	-1,626.13	-3,439.04	186.78	0.042*
	Firmicutes	*Eubacteriaceae*	*Eubacterium hallii*	237.80	22.18	2,548.98	77.24	9.97	598.54	-580.90	-1,781.36	619.57	0.557	69.22	12.84	69.22	-1,380.00	-3,396.47	636.48	0.01*
	Firmicutes	*Eubacteriaceae*	*Eubacterium ramulus*	2,769.19	318.02	24,112.56	1,678.94	234.96	11,996.84	-1,254.94	-4,040.20	1,530.32	0.917	1,620.75	253.26	1,620.75	-1,679.99	-4,905.41	1,545.44	0.028*
	Firmicutes	*Ruminococcaceae*	*Faecalibacterium prausnitzii*	2,360.94	141.95	39,268.59	487.01	46.53	5,097.33	-2,382.09	-4,478.98	-285.21	0.04*	867.30	57.44	867.30	-2,584.13	-4,610.93	-557.33	0.035*
	Firmicutes	*Ruminococcaceae*	*Ruminococcus gnavus*	52,187.50	11,601.63	234,754.51	41,474.78	14,122.32	121,804.13	-19,602.90	-30,165.25	-9,040.55	0.764	29,804.44	5,398.96	29,804.44	-28,314.08	-35,949.82	-20,678.34	0.039*
	Firmicutes	*Ruminococcaceae*	*Ruminococcus sp. JC304*	752.23	90.94	6,221.94	545.32	28.50	10,435.44	-795.19	-1,682.36	91.98	0.780	245.18	28.50	545.32	-1,213.21	-2,283.80	-142.63	0.011*
	Firmicutes	*Erysipelotrichaceae*	*Erysipelatoclostridium ramosum*	1,822.16	3,243.44	1,023.69	1,620.22	2,770.58	947.50	-1,245.75	-3,571.40	1,079.90	0.395	1,082.68	1,364.18	859.27	-1,212.40	-5,479.15	3,054.35	0.284

Trend Analysis

	Phylum	Family	Species	Non-Allergen Sensitized Atopic Eczema (NAE)					Allergen Sensitized Atopic Eczema (AE)					
				Longitudinal Mean Difference (Reads) - B Estimate [φ]	95% CI Lower Bound [φ]	95% CI Upper Bound [φ]	Adj p value for the mean difference [φ]	Adj p value for the slope difference	Longitudinal Mean Difference (Reads) - B Estimate [φ]	95% CI Lower Bound [φ]	95% CI Upper Bound [φ]	Adj p value for the mean difference [φ]	Adj p value for the slope difference	Adj p value for time trend [φ]
**Early Colonizers**	*Bacteroidetes*	*Bacteroidaceae*	*Bacteroides fragilis*	-11,976.60	-57,081.17	33,127.97	0.069	0.932	-21,039.95	-58,323.88	16,243.98	0.014*	0.485	0.748
	Proteobacteria	*Enterobacteriaceae*	*Escherichia coli*	13,536.22	-26,070.10	53,142.53	0.359	0.587	25,576.47	-7,961.03	59,113.97	0.014*	0.283	0.005
	Proteobacteria	*Enterobacteriaceae*	*Klebsiella pneumoniae*	2,188.54	-20,529.30	24,906.38	0.188	0.446	10,625.46	-9,023.54	30,274.47	0.026*	0.876	0.034
**Late Colonizers**	*Firmicutes*	*Lachnospiraceae*	*Anaerostipes caccae*	-79.85	-840.32	680.62	0.340	0.086	-517.08	-1,270.87	236.71	0.043*	0.727	0.002
	Firmicutes	*Lachnospiraceae*	*Blautia producta*	-115.97	-961.46	729.51	0.787	0.548	-647.37	-1,684.27	389.52	0.021*	0.117	<0.001
	Firmicutes	*Lachnospiraceae*	*Blautia wexlerae*	-58.49	-1,342.17	1,225.20	0.929	0.410	-1,011.06	-2,622.48	600.36	0.0189*	0.179	0.004
	Firmicutes	*Lachnospiraceae*	*Lachnospiraceae bacterium*	-359.14	-1,386.74	668.45	0.001*	0.532	-1,863.14	-3,940.20	213.92	0.033*	0.128	<0.001
	Firmicutes	*Lachnospiraceae*	*Tyzzerella nexilis*	-720.52	-4,580.86	3,139.83	0.021*	0.363	-977.14	-2,800.95	846.68	0.038*	0.046	<0.001
	Firmicutes	*Eubacteriaceae*	*Eubacterium limosum*	-415.35	-1,124.34	293.64	0.251	0.615	-2,374.05	-3,805.04	-943.07	<0.001*	0.403	0.032
	Firmicutes	*Eubacteriaceae*	*Eubacterium hallii*	-580.26	-1,213.59	53.07	0.012*	0.893	-950.95	-2,270.12	368.21	0.028*	0.014	0.003
	Firmicutes	*Eubacteriaceae*	*Eubacterium ramulus*	-402.49	-1,770.13	965.15	0.032*	0.673	-758.72	-2,444.06	926.62	0.038*	0.256	<0.001
	Firmicutes	*Ruminococcaceae*	*Faecalibacterium prausnitzii*	-9.55	-1,128.53	1,109.42	0.760	0.826	-125.97	-2,588.47	2,336.52	0.014*	0.931	0.786
	Firmicutes	*Ruminococcaceae*	*Ruminococcus gnavus*	-3,477.46	-7,702.24	747.33	0.014*	0.354	-6,436.67	-17,452.13	4,578.79	0.015*	0.223	<0.001
	Firmicutes	*Ruminococcaceae*	*Ruminococcus sp. JC304*	-240.39	-1,020.82	540.03	0.384	0.395	-1,556.79	-5,102.94	1,989.36	0.0259*	0.092	0.001
	Firmicutes	*Erysipelotrichaceae*	*Erysipelatoclostridium ramosum*	-576.80	-1,684.71	531.10	0.361	0.053	-1,177.17	-9,427.88	7,073.54	0.027*	0.718	0.048

_ Data are presented as geometric mean and geometric standard deviation range for normalized bacterial read counts at species level & plotted in Figure 2b.

[ɸ] General Linear models performed to compare the eczema group (NAE or AE) and controls (reference) at each time point with Bonferroni correction for pair wise comparisons adjusting for baseline values and the 7 mentioned potential confounders (i.e., gender, birth order, mode of delivery, breastfeeding till 6 months, antibiotics during labour, maternal and paternal atopic history).

[φ] Linear mixed model was used to evaluate normalized bacterial read counts longitudinally and assess the trend significance of the trajectories of eczema group (NAE/AE) compared to controls group (i.e., at the selected four timepoints of week 3, months 3, 6 and 12) among the three clinical groups, adjusted for seven potential confounders (i.e., gender, birth order, mode of delivery, breastfeeding till 6 months, antibiotics during labour, maternal and paternal atopic history).

*Significance at adj p <0.05 between eczema (AE/NAE) and control (reference).

Table 1c: Longitudinal maturation of dominant butyrate & propionate producers at metagenomics level

3 Weeks

	Species	Control (n=13)			Non-Allergen Sensitized Atopic Eczema (NAE) (n=5)							Allergen Sensitized Atopic Eczema (AE) (n=5)						
		Geometric Mean (Reads)	Lower Range of Geometric SD	Upper Range of Geometric SD	Geometric Mean (Reads)	Lower Range of Geometric SD	Upper Range of Geometric SD	B Estimate[ψ]	95% CI Lower Bound[ψ]	95% CI Upper Bound[ψ]	Adj p value[ψ]	Geometric Mean (Reads)	Lower Range of Geometric SD	Upper Range of Geometric SD	B Estimate[ψ]	95% CI Lower Bound[ψ]	95% CI Upper Bound[ψ]	Adj p value[ψ]
**Butyrate Producers**	*Bacteroides fragilis, Blautia producta, Blautia wexlerae, Eubacterium ramulus, Erysipelatoclostridium ramosum, Faecalibacterium prausnitzii, Lachnospiraceae bacterium, Tyzzerella nexilis, Ruminococcus gnavus*	4,166.35	69,016.05	251.51	3,668.25	87,310.98	154.12	-1,157.01	-2,237.81	623.80	0.774	320.25	2,544.10	40.31	-5,279.71	-9,846.86	1,287.45	0.036*
**Propionate Producers**	*Anaerostipes caccae, Bacteroides fragilis, Blautia wexlerae, Eubacterium hallii, Eubacterium limosum, Ruminococcus sp. JC304*	1,064.70	38,601.53	29.37	860.52	37,742.99	19.62	-497.59	-1,109.08	523.89	0.699	169.02	1,831.66	15.60	-1,449.47	-2,963.23	264.30	0.037*

3 Months

	Species	Control (n=16)			Non-Allergen Sensitized Atopic Eczema (NAE) (n=11)							Allergen Sensitized Atopic Eczema (AE) (n=10)						
		Geometric Mean (Reads)	Lower Range of Geometric SD	Upper Range of Geometric SD	Geometric Mean (Reads)	Lower Range of Geometric SD	Upper Range of Geometric SD	B Estimate[ψ]	95% CI Lower Bound[ψ]	95% CI Upper Bound[ψ]	Adj p value[ψ]	Geometric Mean (Reads)	Lower Range of Geometric SD	Upper Range of Geometric SD	B Estimate[ψ]	95% CI Lower Bound[ψ]	95% CI Upper Bound[ψ]	Adj p value[ψ]
**Butyrate Producers**	*Bacteroides fragilis, Blautia producta, Blautia wexlerae, Eubacterium ramulus, Erysipelatoclostridium ramosum, Faecalibacterium prausnitzii, Lachnospiraceae bacterium, Tyzzerella nexilis, Ruminococcus gnavus*	27,076.14	302,537.49	2,423.23	14,848.45	250,486.85	880.19	-16,625.54	-30,272.23	17,021.14	0.487	1,311.59	10,003.65	171.96	-27,956.10	-39,460.58	13,548.38	0.039*
**Propionate Producers**	*Anaerostipes caccae, Bacteroides fragilis, Blautia wexlerae, Eubacterium hallii, Eubacterium limosum, Ruminococcus sp. JC304*	6,739.54	164,063.10	276.85	4,696.62	99,007.47	222.79	-2,220.47	-131,198.64	9,657.70	0.195	601.10	7,793.78	46.36	-5,949.13	-13,667.31	7,769.06	0.036*

6 Months

	Species	Control (n=27)			Non-Allergen Sensitized Atopic Eczema (NAE) (n=9)							Allergen Sensitized Atopic Eczema (AE) (n=14)						
		Geometric Mean (Reads)	Lower Range of Geometric SD	Upper Range of Geometric SD	Geometric Mean (Reads)	Lower Range of Geometric SD	Upper Range of Geometric SD	B Estimate[ψ]	95% CI Lower Bound[ψ]	95% CI Upper Bound[ψ]	Adj p value[ψ]	Geometric Mean (Reads)	Lower Range of Geometric SD	Upper Range of Geometric SD	B Estimate[ψ]	95% CI Lower Bound[ψ]	95% CI Upper Bound[ψ]	Adj p value[ψ]
**Butyrate Producers**	*Bacteroides fragilis, Blautia producta, Blautia wexlerae, Eubacterium ramulus, Erysipelatoclostridium ramosum, Faecalibacterium prausnitzii, Lachnospiraceae bacterium, Tyzzerella nexilis, Ruminococcus gnavus*	49,593.77	447,688.73	5,493.87	42,360.60	275,032.05	6,524.41	-1,073.25	-9,698.20	7,751.70	0.635	7,141.87	56,743.63	898.89	-36,192.95	-52,294.36	12,908.46	0.027*
**Propionate Producers**	*Anaerostipes caccae, Bacteroides fragilis, Blautia wexlerae, Eubacterium hallii, Eubacterium limosum, Ruminococcus sp. JC304*	10,603.52	225,859.80	497.81	6,370.07	66,086.74	614.01	-5,885.00	-12,461.29	7,691.29	0.196	2,327.89	44,721.03	121.18	-8,110.90	-21,919.25	2,697.45	0.035*

12 Months

	Species	Control (n=26)			Non-Allergen Sensitized Atopic Eczema (NAE) (n=8)							Allergen Sensitized Atopic Eczema (AE) (n=18)						
		Geometric Mean (Reads)	Lower Range of Geometric SD	Upper Range of Geometric SD	Geometric Mean (Reads)	Lower Range of Geometric SD	Upper Range of Geometric SD	B Estimate[ψ]	95% CI Lower Bound[ψ]	95% CI Upper Bound[ψ]	Adj p value[ψ]	Geometric Mean (Reads)	Lower Range of Geometric SD	Upper Range of Geometric SD	B Estimate[ψ]	95% CI Lower Bound[ψ]	95% CI Upper Bound[ψ]	Adj p value[ψ]
**Butyrate Producers**	*Bacteroides fragilis, Blautia producta, Blautia wexlerae, Eubacterium ramulus, Erysipelatoclostridium ramosum, Faecalibacterium prausnitzii, Lachnospiraceae bacterium, Tyzzerella nexilis, Ruminococcus gnavus*	205,203.98	394,449.71	106,752.95	152,327.32	324,105.61	71,592.75	-38,466.05	-146,176.25	69,244.15	0.475	99,629.66	346,839.78	28,618.60	-81,196.31	-165,511.14	3,118.53	0.009*
**Propionate Producers**	*Anaerostipes caccae, Bacteroides fragilis, Blautia wexlerae, Eubacterium hallii, Eubacterium limosum, Ruminococcus sp. JC304*	30,357.49	116,326.37	7,922.34	38,623.02	191,846.07	7,775.70	1,051.53	-12,885.62	14,788.69	0.431	14,852.77	89,788.78	2,456.93	-29,421.83	-77,044.76	18,201.09	0.021*

Trend Analysis

	Species	Non-Allergen Sensitized Atopic Eczema (NAE)					Allergen Sensitized Atopic Eczema (AE)					
		Longitudinal Mean Difference (Reads) - B Estimate[φ]	95% CI Lower Bound[φ]	95% CI Upper Bound[φ]	Adj p value for the mean difference[φ]	Adj p value for slope difference[φ]	Longitudinal Mean Difference (Reads) - B Estimate[φ]	95% CI Lower Bound[φ]	95% CI Upper Bound[φ]	Adj p value for the mean difference[φ]	Adj p value for slope difference[φ]	Adj p value for time trend[φ]
**Butyrate Producers**	*Bacteroides fragilis, Blautia producta, Blautia wexlerae, Eubacterium ramulus, Erysipelatoclostridium ramosum, Faecalibacterium prausnitzii, Lachnospiraceae bacterium, Tyzzerella nexilis, Ruminococcus gnavus*	-38,342.80	-129,074.95	52,389.36	0.034*	0.613	-94,746.72	94,747.95	-22,051.51	0.012*	0.522	0.001
**Propionate Producers**	*Anaerostipes caccae, Bacteroides fragilis, Blautia wexlerae, Eubacterium hallii, Eubacterium limosum, Ruminococcus sp. JC304*	-24,312.50	-127,202.08	78,577.07	0.342*	0.031	-49,030.06	49,030.12	12,470.81	0.016*	0.102	0.178

_ Data are presented as geometric mean and geometric standard deviation range for normalized bacterial read counts at species level of butyrate and propionate producers & plotted in Supplementary Figure 2.

ψ General Linear models performed to compare the eczema group (NAE or AE) and controls (reference) at each time point with Bonferroni correction for pair wise comparisons adjusting for baseline values and the 7 mentioned potential confounders (i.e., gender, birth order, mode of delivery, breastfeeding till 6 months, antibiotics during labour, maternal and paternal atopic history).

φ Linear mixed model was used to evaluate normalized bacterial read counts longitudinally and assess the trend significance of the trajectories of eczema group (NAE/AE) compared to controls group (i.e., at the selected four timepoints of week 3, months 3, 6 and 12) among the three clinical groups, adjusted for seven potential confounders (i.e., gender, birth order, mode of delivery, breastfeeding till 6 months, antibiotics during labour, maternal and paternal atopic history).

*Significance at adj p <0.05 between eczema (AE/NAE) and control (reference).

Supplementary Table 2: Butyrate and propionate functional annotation derived from metagenomics and metatranscriptomics classification belonging to 13 shortlisted bacterial species.

<table>
<tr><th>Phylum</th><th>Family</th><th>Species</th><th>Metabolic Pathway</th><th>Butyrate & Propionate Functional Genes (Metatranscriptomics Classification based on KEGG database)</th></tr>
<tr><td>Firmicutes</td><td>Lachnospiraceae</td><td>Anaerostipes caccae</td><td>Glycolysis-associated Metabolism</td><td>KEGG | ko00030 Pentose phosphate pathway | K08094 6-phospho-3-hexuloisomerase [EC:5.3.1.27]</td></tr>
<tr><td></td><td></td><td></td><td>Propionate Metabolism</td><td>KEGG | ko00640 Propanoate metabolism | K01034 acetate CoA/acetoacetate CoA-transferase alpha subunit [EC:2.8.3.8 2.8.3.9]</td></tr>
<tr><td>Bacteroidetes</td><td>Bacteroidaceae</td><td>Bacteroides fragilis</td><td>Propionate Metabolism</td><td>KEGG | ko00640 Propanoate metabolism | K11381 2-oxoisovalerate dehydrogenase E1 component [EC:1.2.4.4]</td></tr>
<tr><td></td><td></td><td></td><td></td><td>KEGG | ko00640 Propanoate metabolism | K08325 NADP-dependent alcohol dehydrogenase [EC:1.1.-.-]</td></tr>
<tr><td></td><td></td><td></td><td>Butyrate Metabolism</td><td>KEGG | ko00650 Butanoate metabolism | K00929 butyrate kinase [EC:2.7.2.7]</td></tr>
<tr><td></td><td></td><td></td><td></td><td>KEGG | ko00650 Butanoate metabolism | K00634 phosphate butyryltransferase [EC:2.3.1.19]</td></tr>
<tr><td>Firmicutes</td><td>Lachnospiraceae</td><td>Blautia producta</td><td>Glycolysis-associated Metabolism</td><td>KEGG | ko00010 Glycolysis / Gluconeogenesis | K00016 L-lactate dehydrogenase [EC:1.1.1.27]</td></tr>
<tr><td></td><td></td><td></td><td></td><td>KEGG | ko00010 Glycolysis / Gluconeogenesis | K00845 glucokinase [EC:2.7.1.2]</td></tr>
<tr><td></td><td></td><td></td><td></td><td>KEGG | ko00010 Glycolysis / Gluconeogenesis | K00873 pyruvate kinase [EC:2.7.1.40]</td></tr>
<tr><td></td><td></td><td></td><td>Butyrate Metabolism</td><td>KEGG | ko00650 Butanoate metabolism | K00929 butyrate kinase [EC:2.7.2.7]</td></tr>
<tr><td>Firmicutes</td><td>Lachnospiraceae</td><td>Blautia wexlerae</td><td>Glycolysis-associated Metabolism</td><td>KEGG | ko00010 Glycolysis / Gluconeogenesis | K00850 6-phosphofructokinase 1 [EC:2.7.1.11]</td></tr>
<tr><td></td><td></td><td></td><td></td><td>KEGG | ko00030 Pentose phosphate pathway | K07404 6-phosphogluconolactonase [EC:3.1.1.31]</td></tr>
<tr><td></td><td></td><td></td><td></td><td>KEGG | ko00620 Pyruvate metabolism | K00625 phosphate acetyltransferase [EC:2.3.1.8]</td></tr>
<tr><td></td><td></td><td></td><td>Propionate Metabolism</td><td>KEGG | ko00640 Propanoate metabolism | K13922 propionaldehyde dehydrogenase [EC:1.2.1.87]</td></tr>
<tr><td></td><td></td><td></td><td>Butyrate Metabolism</td><td>KEGG | ko00650 Butanoate metabolism | K18120 4-hydroxybutyrate dehydrogenase [EC:1.1.1.61]</td></tr>
<tr><td>Firmicutes</td><td>Eubacteriaceae</td><td>Eubacterium hallii</td><td>Propionate Metabolism</td><td>KEGG | ko00640 Propanoate metabolism | K01699 propanediol dehydratase large subunit [EC:4.2.1.28]</td></tr>
<tr><td>Firmicutes</td><td>Eubacteriaceae</td><td>Eubacterium limosum</td><td>Propionate Metabolism</td><td>KEGG | ko00640 Propanoate metabolism | K00005 glycerol dehydrogenase [EC:1.1.1.6]</td></tr>
<tr><td></td><td></td><td></td><td></td><td>KEGG | ko00640 Propanoate metabolism | K13921 1-propanol dehydrogenase</td></tr>
<tr><td></td><td></td><td></td><td></td><td>KEGG | ko00640 Propanoate metabolism | K13922 propionaldehyde dehydrogenase [EC:1.2.1.87]</td></tr>
<tr><td>Firmicutes</td><td>Eubacteriaceae</td><td>Eubacterium ramulus</td><td>Butyrate Metabolism</td><td>KEGG | ko00650 Butanoate metabolism | K00248 butyryl-CoA dehydrogenase [EC:1.3.8.1]</td></tr>
<tr><td></td><td></td><td></td><td></td><td>KEGG | ko00650 Butanoate metabolism | K01652 acetolactate synthase I/II/III large subunit [EC:2.2.1.6]</td></tr>
<tr><td>Firmicutes</td><td>Erysipelotrichaceae</td><td>Erysipelatoclostridium ramosum</td><td>Butyrate Metabolism</td><td>KEGG | ko00650 Butanoate metabolism | K01652 acetolactate synthase I/II/III large subunit [EC:2.2.1.6]</td></tr>
<tr><td>Firmicutes</td><td>Ruminococcaceae</td><td>Faecalibacterium prausnitzii</td><td>Butyrate Metabolism</td><td>KEGG | ko00650 Butanoate metabolism | K01653 acetolactate synthase I/III small subunit [EC:2.2.1.6]</td></tr>
<tr><td>Firmicutes</td><td>Lachnospiraceae</td><td>Lachnospiraceae bacterium</td><td>Glycolysis-associated Metabolism</td><td>KEGG | ko00010 Glycolysis / Gluconeogenesis | K00850 6-phosphofructokinase 1 [EC:2.7.1.11]</td></tr>
<tr><td></td><td></td><td></td><td></td><td>KEGG | ko00030 Pentose phosphate pathway | K00615 transketolase [EC:2.2.1.1]</td></tr>
<tr><td></td><td></td><td></td><td>Butyrate Metabolism</td><td>KEGG | ko00650 Butanoate metabolism | K00074 3-hydroxybutyryl-CoA dehydrogenase [EC:1.1.1.157]</td></tr>
<tr><td></td><td></td><td></td><td></td><td>KEGG | ko00650 Butanoate metabolism | K00100 butanol dehydrogenase [EC:1.1.1.-]</td></tr>
<tr><td></td><td></td><td></td><td></td><td>KEGG | ko00650 Butanoate metabolism | K00248 butyryl-CoA dehydrogenase [EC:1.3.8.1]</td></tr>
<tr><td>Firmicutes</td><td>Lachnospiraceae</td><td>Tyzzerella nexilis</td><td>Glycolysis-associated Metabolism</td><td>KEGG | ko00010 Glycolysis / Gluconeogenesis | K00845 glucokinase [EC:2.7.1.2]</td></tr>
<tr><td></td><td></td><td></td><td></td><td>KEGG | ko00020 Citrate cycle (TCA cycle) | K01678 fumarate hydratase subunit beta [EC:4.2.1.2]</td></tr>
<tr><td></td><td></td><td></td><td></td><td>KEGG | ko00500 Starch and sucrose metabolism | K00700 1,4-alpha-glucan branching enzyme [EC:2.4.1.18]</td></tr>
<tr><td></td><td></td><td></td><td></td><td>KEGG | ko00500 Starch and sucrose metabolism | K00975 glucose-1-phosphate adenylyltransferase [EC:2.7.7.27]</td></tr>
<tr><td></td><td></td><td></td><td></td><td>KEGG | ko00500 Starch and sucrose metabolism | K02438 glycogen debranching enzyme [EC:3.2.1.196]</td></tr>
<tr><td></td><td></td><td></td><td>Butyrate Metabolism</td><td>KEGG | ko00620 Pyruvate metabolism | K01961 acetyl-CoA carboxylase, biotin carboxylase subunit [EC:6.4.1.2 6.3.4.14]</td></tr>
<tr><td>Firmicutes</td><td>Ruminococcaceae</td><td>Ruminococcus gnavus</td><td>Glycolysis-associated Metabolism</td><td>KEGG | ko00010 Glycolysis / Gluconeogenesis | K01223 6-phospho-beta-glucosidase [EC:3.2.1.86]</td></tr>
<tr><td></td><td></td><td></td><td></td><td>KEGG | ko00010 Glycolysis / Gluconeogenesis | K01624 fructose-bisphosphate aldolase, class II [EC:4.1.2.13]</td></tr>
<tr><td></td><td></td><td></td><td></td><td>KEGG | ko00010 Glycolysis / Gluconeogenesis | K03737 pyruvate-ferredoxin/flavodoxin oxidoreductase [EC:1.2.7.1 1.2.7.-]</td></tr>
<tr><td></td><td></td><td></td><td></td><td>KEGG | ko00020 Citrate cycle (TCA cycle) | K01677 fumarate hydratase subunit alpha [EC:4.2.1.2]</td></tr>
<tr><td></td><td></td><td></td><td></td><td>KEGG | ko00030 Pentose phosphate pathway | K00852 ribokinase [EC:2.7.1.15]</td></tr>
<tr><td></td><td></td><td></td><td></td><td>KEGG | ko00500 Starch and sucrose metabolism | K02759 PTS system, cellobiose-specific IIA component [EC:2.7.1.196 2.7.1.205]</td></tr>
<tr><td></td><td></td><td></td><td></td><td>KEGG | ko00500 Starch and sucrose metabolism | K02810 PTS system, sucrose-specific IIC component</td></tr>
<tr><td></td><td></td><td></td><td></td><td>KEGG | ko00500 Starch and sucrose metabolism | K05349 beta-glucosidase [EC:3.2.1.21]</td></tr>
<tr><td></td><td></td><td></td><td></td><td>KEGG | ko00620 Pyruvate metabolism | K01512 acylphosphatase [EC:3.6.1.7]</td></tr>
<tr><td></td><td></td><td></td><td></td><td>KEGG | ko00620 Pyruvate metabolism | K01649 2-isopropylmalate synthase [EC:2.3.3.13]</td></tr>
<tr><td></td><td></td><td></td><td></td><td>KEGG | ko00620 Pyruvate metabolism | K01961 acetyl-CoA carboxylase, biotin carboxylase subunit [EC:6.4.1.2 6.3.4.14]</td></tr>
<tr><td></td><td></td><td></td><td>Butyrate Metabolism</td><td>KEGG | ko00650 Butanoate metabolism | K00074 3-hydroxybutyryl-CoA dehydrogenase [EC:1.1.1.157]</td></tr>
<tr><td></td><td></td><td></td><td></td><td>KEGG | ko00650 Butanoate metabolism | K00100 butanol dehydrogenase [EC:1.1.1.-]</td></tr>
<tr><td>Firmicutes</td><td>Ruminococcaceae</td><td>Ruminococcus sp. JC304</td><td>Propionate Metabolism</td><td>KEGG | ko00640 Propanoate metabolism | K01734 methylglyoxal synthase [EC:4.2.3.3]</td></tr>
</table>

<table>
<tr><th>Phylum</th><th>Family</th><th>Species</th><th>Metabolic Pathway</th><th>Butyrate & Propionate Functional Genes (Metagenomics Classification based on KEGG database)</th></tr>
<tr><td>Firmicutes</td><td>Lachnospiraceae</td><td>Anaerostipes caccae</td><td>Glycolysis-associated Metabolism</td><td>KEGG | ko00010 Glycolysis / Gluconeogenesis | K00128 aldehyde dehydrogenase (NAD+) [EC:1.2.1.3] | "</td></tr>
<tr><td></td><td></td><td></td><td></td><td>KEGG | ko00010 Glycolysis / Gluconeogenesis | K00134 glyceraldehyde 3-phosphate dehydrogenase [EC:1.2.1.12] | "</td></tr>
<tr><td></td><td></td><td></td><td></td><td>KEGG | ko00010 Glycolysis / Gluconeogenesis | K00161 pyruvate dehydrogenase E1 component alpha subunit [EC:1.2.4.1] | "</td></tr>
<tr><td></td><td></td><td></td><td></td><td>KEGG | ko00010 Glycolysis / Gluconeogenesis | K00162 pyruvate dehydrogenase E1 component beta subunit [EC:1.2.4.1] | "</td></tr>
<tr><td></td><td></td><td></td><td></td><td>KEGG | ko00010 Glycolysis / Gluconeogenesis | K00382 dihydrolipoamide dehydrogenase [EC:1.8.1.4] | "</td></tr>
<tr><td></td><td></td><td></td><td></td><td>KEGG | ko00010 Glycolysis / Gluconeogenesis | K00627 pyruvate dehydrogenase E2 component (dihydrolipoamide acetyltransferase) [EC:2.3.1.12] | "</td></tr>
<tr><td></td><td></td><td></td><td></td><td>KEGG | ko00010 Glycolysis / Gluconeogenesis | K00850 6-phosphofructokinase 1 [EC:2.7.1.11] | "</td></tr>
<tr><td></td><td></td><td></td><td></td><td>KEGG | ko00010 Glycolysis / Gluconeogenesis | K01223 6-phospho-beta-glucosidase [EC:3.2.1.86] | "</td></tr>
<tr><td></td><td></td><td></td><td></td><td>KEGG | ko00010 Glycolysis / Gluconeogenesis | K01624 fructose-bisphosphate aldolase, class II [EC:4.1.2.13] | "</td></tr>
<tr><td></td><td></td><td></td><td></td><td>KEGG | ko00010 Glycolysis / Gluconeogenesis | K01689 enolase [EC:4.2.1.11] | "</td></tr>
<tr><td></td><td></td><td></td><td></td><td>KEGG | ko00010 Glycolysis / Gluconeogenesis | K01803 triosephosphate isomerase (TIM) [EC:5.3.1.1] | "</td></tr>
<tr><td></td><td></td><td></td><td></td><td>KEGG | ko00010 Glycolysis / Gluconeogenesis | K01834 2,3-bisphosphoglycerate-dependent phosphoglycerate mutase [EC:5.4.2.11] | "</td></tr>
<tr><td></td><td></td><td></td><td></td><td>KEGG | ko00010 Glycolysis / Gluconeogenesis | K01835 phosphoglucomutase [EC:5.4.2.2] | "</td></tr>
<tr><td></td><td></td><td></td><td></td><td>KEGG | ko00010 Glycolysis / Gluconeogenesis | K04041 fructose-1,6-bisphosphatase III [EC:3.1.3.11] | "</td></tr>
<tr><td></td><td></td><td></td><td></td><td>KEGG | ko00010 Glycolysis / Gluconeogenesis | K15633 2,3-bisphosphoglycerate-independent phosphoglycerate mutase [EC:5.4.2.12] | "</td></tr>
<tr><td></td><td></td><td></td><td></td><td>KEGG | ko00010 Glycolysis / Gluconeogenesis | K16370 6-phosphofructokinase 2 [EC:2.7.1.11] | "</td></tr>
<tr><td></td><td></td><td></td><td></td><td>KEGG | ko00020 Citrate cycle (TCA cycle) | K01647 citrate synthase [EC:2.3.3.1] | "</td></tr>
<tr><td></td><td></td><td></td><td></td><td>KEGG | ko00020 Citrate cycle (TCA cycle) | K01677 fumarate hydratase subunit alpha [EC:4.2.1.2] | "</td></tr>
<tr><td></td><td></td><td></td><td></td><td>KEGG | ko00020 Citrate cycle (TCA cycle) | K01678 fumarate hydratase subunit beta [EC:4.2.1.2] | "</td></tr>
<tr><td></td><td></td><td></td><td></td><td>KEGG | ko00020 Citrate cycle (TCA cycle) | K01681 aconitate hydratase [EC:4.2.1.3] | "</td></tr>
<tr><td></td><td></td><td></td><td></td><td>KEGG | ko00030 Pentose phosphate pathway | K00615 transketolase [EC:2.2.1.1] | "</td></tr>
<tr><td></td><td></td><td></td><td></td><td>KEGG | ko00030 Pentose phosphate pathway | K00616 transaldolase [EC:2.2.1.2] | "</td></tr>
<tr><td></td><td></td><td></td><td></td><td>KEGG | ko00030 Pentose phosphate pathway | K00852 ribokinase [EC:2.7.1.15] | "</td></tr>
<tr><td></td><td></td><td></td><td></td><td>KEGG | ko00030 Pentose phosphate pathway | K00874 2-dehydro-3-deoxygluconokinase [EC:2.7.1.45] | "</td></tr>
<tr><td></td><td></td><td></td><td></td><td>KEGG | ko00030 Pentose phosphate pathway | K01619 deoxyribose-phosphate aldolase [EC:4.1.2.4] | "</td></tr>
<tr><td></td><td></td><td></td><td></td><td>KEGG | ko00030 Pentose phosphate pathway | K01625 2-dehydro-3-deoxyphosphogluconate aldolase / (4S)-4-hydroxy-2-oxoglutarate aldolase [EC:4.1.2.14 4.1.3.42] | "</td></tr>
<tr><td></td><td></td><td></td><td></td><td>KEGG | ko00030 Pentose phosphate pathway | K01783 ribulose-phosphate 3-epimerase [EC:5.1.3.1] | "</td></tr>
<tr><td></td><td></td><td></td><td></td><td>KEGG | ko00030 Pentose phosphate pathway | K01839 phosphopentomutase [EC:5.4.2.7] | "</td></tr>
<tr><td></td><td></td><td></td><td></td><td>KEGG | ko00030 Pentose phosphate pathway | K08093 3-hexulose-6-phosphate synthase [EC:4.1.2.43] | "</td></tr>
<tr><td></td><td></td><td></td><td></td><td>KEGG | ko00030 Pentose phosphate pathway | K08094 6-phospho-3-hexuloisomerase [EC:5.3.1.27] | "</td></tr>
<tr><td></td><td></td><td></td><td></td><td>KEGG | ko00030 Pentose phosphate pathway | K17466 PTS system, D-glucosaminate-specific IIC component | "</td></tr>
<tr><td></td><td></td><td></td><td></td><td>KEGG | ko00030 Pentose phosphate pathway | K17467 PTS system, D-glucosaminate-specific IID component | "</td></tr>
<tr><td></td><td></td><td></td><td></td><td>KEGG | ko00500 Starch and sucrose metabolism | K00688 glycogen phosphorylase [EC:2.4.1.1] | "</td></tr>
<tr><td></td><td></td><td></td><td></td><td>KEGG | ko00500 Starch and sucrose metabolism | K00705 4-alpha-glucanotransferase [EC:2.4.1.25] | "</td></tr>
<tr><td></td><td></td><td></td><td></td><td>KEGG | ko00500 Starch and sucrose metabolism | K01232 maltose-6'-phosphate glucosidase [EC:3.2.1.122] | "</td></tr>
<tr><td></td><td></td><td></td><td></td><td>KEGG | ko00500 Starch and sucrose metabolism | K02438 glycogen debranching enzyme [EC:3.2.1.196] | "</td></tr>
<tr><td></td><td></td><td></td><td></td><td>KEGG | ko00500 Starch and sucrose metabolism | K02750 PTS system, alpha-glucoside-specific IIC component | "</td></tr>
<tr><td></td><td></td><td></td><td></td><td>KEGG | ko00500 Starch and sucrose metabolism | K02761 PTS system, cellobiose-specific IIC component | "</td></tr>
<tr><td></td><td></td><td></td><td></td><td>KEGG | ko00500 Starch and sucrose metabolism | K02819 PTS system, trehalose-specific IIC component | "</td></tr>
<tr><td></td><td></td><td></td><td></td><td>KEGG | ko00620 Pyruvate metabolism | K00027 malate dehydrogenase (oxaloacetate-decarboxylating) [EC:1.1.1.38] | "</td></tr>
<tr><td></td><td></td><td></td><td></td><td>KEGG | ko00620 Pyruvate metabolism | K00625 phosphate acetyltransferase [EC:2.3.1.8] | "</td></tr>
<tr><td></td><td></td><td></td><td></td><td>KEGG | ko00620 Pyruvate metabolism | K00656 formate C-acetyltransferase [EC:2.3.1.54] | "</td></tr>
<tr><td></td><td></td><td></td><td></td><td>KEGG | ko00620 Pyruvate metabolism | K00925 acetate kinase [EC:2.7.2.1] | "</td></tr>
<tr><td></td><td></td><td></td><td></td><td>KEGG | ko00620 Pyruvate metabolism | K01026 propionate CoA-transferase [EC:2.8.3.1] | "</td></tr>
<tr><td></td><td></td><td></td><td>Propionate Metabolism</td><td>KEGG | ko00640 Propanoate metabolism | K01034 acetate CoA/acetoacetate CoA-transferase alpha subunit [EC:2.8.3.8 2.8.3.9] | "</td></tr>
<tr><td></td><td></td><td></td><td></td><td>KEGG | ko00640 Propanoate metabolism | K01035 acetate CoA/acetoacetate CoA-transferase beta subunit [EC:2.8.3.8 2.8.3.9] | "</td></tr>
<tr><td></td><td></td><td></td><td></td><td>KEGG | ko00640 Propanoate metabolism | K11381 2-oxoisovalerate dehydrogenase E1 component [EC:1.2.4.4] | "</td></tr>
<tr><td></td><td></td><td></td><td>Butyrate Metabolism</td><td>KEGG | ko00650 Butanoate metabolism | K00100 butanol dehydrogenase [EC:1.1.1.-] | "</td></tr>
<tr><td></td><td></td><td></td><td></td><td>KEGG | ko00650 Butanoate metabolism | K00209 enoyl-[acyl-carrier protein] reductase / trans-2-enoyl-CoA reductase (NAD+) [EC:1.3.1.9 1.3.1.44] | "</td></tr>
<tr><td></td><td></td><td></td><td></td><td>KEGG | ko00650 Butanoate metabolism | K01652 acetolactate synthase I/II/III large subunit [EC:2.2.1.6] | "</td></tr>
<tr><td></td><td></td><td></td><td></td><td>KEGG | ko00650 Butanoate metabolism | K01653 acetolactate synthase I/III small subunit [EC:2.2.1.6] | "</td></tr>
<tr><td></td><td></td><td></td><td></td><td>KEGG | ko00650 Butanoate metabolism | K01715 enoyl-CoA hydratase [EC:4.2.1.17] | "</td></tr>
<tr><td></td><td></td><td></td><td></td><td>KEGG | ko00650 Butanoate metabolism | K14534 4-hydroxybutyryl-CoA dehydratase / vinylacetyl-CoA-Delta-isomerase [EC:4.2.1.120 5.3.3.3] | "</td></tr>
<tr><td></td><td></td><td></td><td></td><td>KEGG | ko00650 Butanoate metabolism | K18120 4-hydroxybutyrate dehydrogenase [EC:1.1.1.61] | "</td></tr>
<tr><td></td><td></td><td></td><td></td><td>KEGG | ko00650 Butanoate metabolism | K18122 4-hydroxybutyrate CoA-transferase [EC:2.8.3.-] | "</td></tr>
<tr><td></td><td></td><td></td><td></td><td>KEGG | ko00010 Glycolysis / Gluconeogenesis | K00001 alcohol dehydrogenase [EC:1.1.1.1] | "</td></tr>
<tr><td></td><td></td><td></td><td></td><td>KEGG | ko00010 Glycolysis / Gluconeogenesis | K00016 L-lactate dehydrogenase [EC:1.1.1.27] | "</td></tr>
<tr><td></td><td></td><td></td><td></td><td>KEGG | ko00010 Glycolysis / Gluconeogenesis | K00128 aldehyde dehydrogenase (NAD+) [EC:1.2.1.3] | "</td></tr>
<tr><td></td><td></td><td></td><td></td><td>KEGG | ko00010 Glycolysis / Gluconeogenesis | K00134 glyceraldehyde 3-phosphate dehydrogenase [EC:1.2.1.12] | "</td></tr>
<tr><td></td><td></td><td></td><td></td><td>KEGG | ko00010 Glycolysis / Gluconeogenesis | K00138 aldehyde dehydrogenase [EC:1.2.1.-] | "</td></tr>
<tr><td></td><td></td><td></td><td></td><td>KEGG | ko00010 Glycolysis / Gluconeogenesis | K00163 pyruvate dehydrogenase E1 component [EC:1.2.4.1] | "</td></tr>
<tr><td></td><td></td><td></td><td></td><td>KEGG | ko00010 Glycolysis / Gluconeogenesis | K00174 2-oxoglutarate/2-oxoacid ferredoxin oxidoreductase subunit alpha [EC:1.2.7.3 1.2.7.11] | "</td></tr>
<tr><td></td><td></td><td></td><td></td><td>KEGG | ko00010 Glycolysis / Gluconeogenesis | K00175 2-oxoglutarate/2-oxoacid ferredoxin oxidoreductase subunit beta [EC:1.2.7.3 1.2.7.11] | "</td></tr>
<tr><td></td><td></td><td></td><td></td><td>KEGG | ko00010 Glycolysis / Gluconeogenesis | K00382 dihydrolipoamide dehydrogenase [EC:1.8.1.4] | "</td></tr>
<tr><td></td><td></td><td></td><td></td><td>KEGG | ko00010 Glycolysis / Gluconeogenesis | K00627 pyruvate dehydrogenase E2 component (dihydrolipoamide acetyltransferase) [EC:2.3.1.12] | "</td></tr>
<tr><td></td><td></td><td></td><td></td><td>KEGG | ko00010 Glycolysis / Gluconeogenesis | K00844 hexokinase [EC:2.7.1.1] | "</td></tr>
<tr><td></td><td></td><td></td><td></td><td>KEGG | ko00010 Glycolysis / Gluconeogenesis | K00845 glucokinase [EC:2.7.1.2] | "</td></tr>
<tr><td></td><td></td><td></td><td></td><td>KEGG | ko00010 Glycolysis / Gluconeogenesis | K00850 6-phosphofructokinase 1 [EC:2.7.1.11] | "</td></tr>
<tr><td></td><td></td><td></td><td></td><td>KEGG | ko00010 Glycolysis / Gluconeogenesis | K00873 pyruvate kinase [EC:2.7.1.40] | "</td></tr>
<tr><td></td><td></td><td></td><td></td><td>KEGG | ko00010 Glycolysis / Gluconeogenesis | K00886 polyphosphate glucokinase [EC:2.7.1.63] | "</td></tr>
<tr><td></td><td></td><td></td><td></td><td>KEGG | ko00010 Glycolysis / Gluconeogenesis | K00895 diphosphate-dependent phosphofructokinase [EC:2.7.1.90] | "</td></tr>
<tr><td></td><td></td><td></td><td></td><td>KEGG | ko00010 Glycolysis / Gluconeogenesis | K00927 phosphoglycerate kinase [EC:2.7.2.3] | "</td></tr>
<tr><td></td><td></td><td></td><td></td><td>KEGG | ko00010 Glycolysis / Gluconeogenesis | K01222 6-phospho-beta-glucosidase [EC:3.2.1.86] | "</td></tr>
<tr><td></td><td></td><td></td><td></td><td>KEGG | ko00010 Glycolysis / Gluconeogenesis | K01223 6-phospho-beta-glucosidase [EC:3.2.1.86] | "</td></tr>
<tr><td></td><td></td><td></td><td></td><td>KEGG | ko00010 Glycolysis / Gluconeogenesis | K01610 phosphoenolpyruvate carboxykinase (ATP) [EC:4.1.1.49] | "</td></tr>
<tr><td></td><td></td><td></td><td></td><td>KEGG | ko00010 Glycolysis / Gluconeogenesis | K01624 fructose-bisphosphate aldolase, class II [EC:4.1.2.13] | "</td></tr>
<tr><td></td><td></td><td></td><td></td><td>KEGG | ko00010 Glycolysis / Gluconeogenesis | K01689 enolase [EC:4.2.1.11] | "</td></tr>
<tr><td></td><td></td><td></td><td></td><td>KEGG | ko00010 Glycolysis / Gluconeogenesis | K01785 aldose 1-epimerase [EC:5.1.3.3] | "</td></tr>
<tr><td></td><td></td><td></td><td></td><td>KEGG | ko00010 Glycolysis / Gluconeogenesis | K01803 triosephosphate isomerase (TIM) [EC:5.3.1.1] | "</td></tr>
<tr><td></td><td></td><td></td><td></td><td>KEGG | ko00010 Glycolysis / Gluconeogenesis | K01810 glucose-6-phosphate isomerase [EC:5.3.1.9] | "</td></tr>
<tr><td></td><td></td><td></td><td></td><td>KEGG | ko00010 Glycolysis / Gluconeogenesis | K01834 2,3-bisphosphoglycerate-dependent phosphoglycerate mutase [EC:5.4.2.11] | "</td></tr>
<tr><td></td><td></td><td></td><td></td><td>KEGG | ko00010 Glycolysis / Gluconeogenesis | K01835 phosphoglucomutase [EC:5.4.2.2] | "</td></tr>
<tr><td></td><td></td><td></td><td></td><td>KEGG | ko00010 Glycolysis / Gluconeogenesis | K01895 acetyl-CoA synthetase [EC:6.2.1.1] | "</td></tr>
<tr><td></td><td></td><td></td><td></td><td>KEGG | ko00010 Glycolysis / Gluconeogenesis | K03737 pyruvate-ferredoxin/flavodoxin oxidoreductase [EC:1.2.7.1 1.2.7.-] | "</td></tr>
<tr><td></td><td></td><td></td><td></td><td>KEGG | ko00010 Glycolysis / Gluconeogenesis | K04041 fructose-1,6-bisphosphatase III [EC:3.1.3.11] | "</td></tr>
<tr><td></td><td></td><td></td><td></td><td>KEGG | ko00010 Glycolysis / Gluconeogenesis | K04072 acetaldehyde dehydrogenase / alcohol dehydrogenase [EC:1.2.1.10 1.1.1.1] | "</td></tr>
<tr><td></td><td></td><td></td><td></td><td>KEGG | ko00010 Glycolysis / Gluconeogenesis | K11645 fructose-bisphosphate aldolase, class I [EC:4.1.2.13] | "</td></tr>
<tr><td></td><td></td><td></td><td></td><td>KEGG | ko00010 Glycolysis / Gluconeogenesis | K15633 2,3-bisphosphoglycerate-independent phosphoglycerate mutase [EC:5.4.2.12] | "</td></tr>
<tr><td></td><td></td><td></td><td></td><td>KEGG | ko00010 Glycolysis / Gluconeogenesis | K15634 probable phosphoglycerate mutase [EC:5.4.2.12] | "</td></tr>
<tr><td></td><td></td><td></td><td></td><td>KEGG | ko00010 Glycolysis / Gluconeogenesis | K15635 2,3-bisphosphoglycerate-independent phosphoglycerate mutase [EC:5.4.2.12] | "</td></tr>
<tr><td></td><td></td><td></td><td></td><td>KEGG | ko00020 Citrate cycle (TCA cycle) | K00024 malate dehydrogenase [EC:1.1.1.37] | "</td></tr>
<tr><td></td><td></td><td></td><td></td><td>KEGG | ko00020 Citrate cycle (TCA cycle) | K00031 isocitrate dehydrogenase [EC:1.1.1.42] | "</td></tr>
<tr><td></td><td></td><td></td><td></td><td>KEGG | ko00020 Citrate cycle (TCA cycle) | K00116 malate dehydrogenase (quinone) [EC:1.1.5.4] | "</td></tr>
<tr><td></td><td></td><td></td><td></td><td>KEGG | ko00020 Citrate cycle (TCA cycle) | K00176 2-oxoglutarate ferredoxin oxidoreductase subunit delta [EC:1.2.7.3] | "</td></tr>
<tr><td></td><td></td><td></td><td></td><td>KEGG | ko00020 Citrate cycle (TCA cycle) | K00177 2-oxoglutarate ferredoxin oxidoreductase subunit gamma [EC:1.2.7.3] | "</td></tr>
<tr><td></td><td></td><td></td><td></td><td>KEGG | ko00020 Citrate cycle (TCA cycle) | K00239 succinate dehydrogenase / fumarate reductase, flavoprotein subunit [EC:1.3.5.1 1.3.5.4] | "</td></tr>
<tr><td></td><td></td><td></td><td></td><td>KEGG | ko00020 Citrate cycle (TCA cycle) | K00240 succinate dehydrogenase / fumarate reductase, iron-sulfur subunit [EC:1.3.5.1 1.3.5.4] | "</td></tr>
<tr><td></td><td></td><td></td><td></td><td>KEGG | ko00020 Citrate cycle (TCA cycle) | K00241 succinate dehydrogenase / fumarate reductase, cytochrome b subunit | "</td></tr>
<tr><td></td><td></td><td></td><td></td><td>KEGG | ko00020 Citrate cycle (TCA cycle) | K00658 2-oxoglutarate dehydrogenase E2 component (dihydrolipoamide succinyltransferase) [EC:2.3.1.61] | "</td></tr>
<tr><td></td><td></td><td></td><td></td><td>KEGG | ko00020 Citrate cycle (TCA cycle) | K01647 citrate synthase [EC:2.3.3.1] | "</td></tr>
<tr><td></td><td></td><td></td><td></td><td>KEGG | ko00020 Citrate cycle (TCA cycle) | K01676 fumarate hydratase, class I [EC:4.2.1.2] | "</td></tr>
<tr><td></td><td></td><td></td><td></td><td>KEGG | ko00020 Citrate cycle (TCA cycle) | K01679 fumarate hydratase, class II [EC:4.2.1.2] | "</td></tr>
<tr><td></td><td></td><td></td><td></td><td>KEGG | ko00020 Citrate cycle (TCA cycle) | K01681 aconitate hydratase [EC:4.2.1.3] | "</td></tr>
<tr><td></td><td></td><td></td><td></td><td>KEGG | ko00020 Citrate cycle (TCA cycle) | K01682 aconitate hydratase 2 / 2-methylisocitrate dehydratase [EC:4.2.1.3 4.2.1.99] | "</td></tr>
<tr><td></td><td></td><td></td><td></td><td>KEGG | ko00020 Citrate cycle (TCA cycle) | K01902 succinyl-CoA synthetase alpha subunit [EC:6.2.1.5] | "</td></tr>
<tr><td></td><td></td><td></td><td></td><td>KEGG | ko00020 Citrate cycle (TCA cycle) | K01903 succinyl-CoA synthetase beta subunit [EC:6.2.1.5] | "</td></tr>
<tr><td></td><td></td><td></td><td></td><td>KEGG | ko00020 Citrate cycle (TCA cycle) | K01960 pyruvate carboxylase subunit B [EC:6.4.1.1] | "</td></tr>
<tr><td></td><td></td><td></td><td></td><td>KEGG | ko00030 Pentose phosphate pathway | K00033 6-phosphogluconate dehydrogenase [EC:1.1.1.44 1.1.1.343] | "</td></tr>
<tr><td></td><td></td><td></td><td></td><td>KEGG | ko00030 Pentose phosphate pathway | K00036 glucose-6-phosphate 1-dehydrogenase [EC:1.1.1.49 1.1.1.363] | "</td></tr>
</table>

Bacteroidetes	*Bacteroidaceae*	*Bacteroides fragilis*	Glycolysis-associated Metabolism	KEGG | ko00030 Pentose phosphate pathway | K00117 quinoprotein glucose dehydrogenase [EC:1.1.5.2] | "
				KEGG | ko00030 Pentose phosphate pathway | K00615 transketolase [EC:2.2.1.1] | "
				KEGG | ko00030 Pentose phosphate pathway | K00616 transaldolase [EC:2.2.1.2] | "
				KEGG | ko00030 Pentose phosphate pathway | K00851 gluconokinase [EC:2.7.1.12] | "
				KEGG | ko00030 Pentose phosphate pathway | K00852 ribokinase [EC:2.7.1.15] | "
				KEGG | ko00030 Pentose phosphate pathway | K00874 2-dehydro-3-deoxygluconokinase [EC:2.7.1.45] | "
				KEGG | ko00030 Pentose phosphate pathway | K00948 ribose-phosphate pyrophosphokinase [EC:2.7.6.1] | "
				KEGG | ko00030 Pentose phosphate pathway | K01057 6-phosphogluconolactonase [EC:3.1.1.31] | "
				KEGG | ko00030 Pentose phosphate pathway | K01619 deoxyribose-phosphate aldolase [EC:4.1.2.4] | "
				KEGG | ko00030 Pentose phosphate pathway | K01621 xylulose-5-phosphate/fructose-6-phosphate phosphoketolase [EC:4.1.2.9 4.1.2.22] | "
				KEGG | ko00030 Pentose phosphate pathway | K01625 2-dehydro-3-deoxyphosphogluconate aldolase / (4S)-4-hydroxy-2-oxoglutarate aldolase [EC:4.1.2.14 4.1.3.42] | "
				KEGG | ko00030 Pentose phosphate pathway | K01783 ribulose-phosphate 3-epimerase [EC:5.1.3.1] | "
				KEGG | ko00030 Pentose phosphate pathway | K01807 ribose 5-phosphate isomerase A [EC:5.3.1.6] | "
				KEGG | ko00030 Pentose phosphate pathway | K01808 ribose 5-phosphate isomerase B [EC:5.3.1.6] | "
				KEGG | ko00030 Pentose phosphate pathway | K01839 phosphopentomutase [EC:5.4.2.7] | "
				KEGG | ko00500 Starch and sucrose metabolism | K00688 glycogen phosphorylase [EC:2.4.1.1] | "
				KEGG | ko00500 Starch and sucrose metabolism | K00690 sucrose phosphorylase [EC:2.4.1.7] | "
				KEGG | ko00500 Starch and sucrose metabolism | K00691 maltose phosphorylase [EC:2.4.1.8] | "
				KEGG | ko00500 Starch and sucrose metabolism | K00697 trehalose 6-phosphate synthase [EC:2.4.1.15] | "
				KEGG | ko00500 Starch and sucrose metabolism | K00700 1,4-alpha-glucan branching enzyme [EC:2.4.1.18] | "
				KEGG | ko00500 Starch and sucrose metabolism | K00703 starch synthase [EC:2.4.1.21] | "
				KEGG | ko00500 Starch and sucrose metabolism | K00705 4-alpha-glucanotransferase [EC:2.4.1.25] | "
				KEGG | ko00500 Starch and sucrose metabolism | K00975 glucose-1-phosphate adenylyltransferase [EC:2.7.7.27] | "
				KEGG | ko00500 Starch and sucrose metabolism | K00978 glucose-1-phosphate cytidylyltransferase [EC:2.7.7.33] | "
				KEGG | ko00500 Starch and sucrose metabolism | K01176 alpha-amylase [EC:3.2.1.1] | "
				KEGG | ko00500 Starch and sucrose metabolism | K01179 endoglucanase [EC:3.2.1.4] | "
				KEGG | ko00500 Starch and sucrose metabolism | K01194 alpha,alpha-trehalase [EC:3.2.1.28] | "
				KEGG | ko00500 Starch and sucrose metabolism | K01210 glucan 1,3-beta-glucosidase [EC:3.2.1.58] | "
				KEGG | ko00500 Starch and sucrose metabolism | K01232 maltose-6'-phosphate glucosidase [EC:3.2.1.122] | "
				KEGG | ko00500 Starch and sucrose metabolism | K02438 glycogen debranching enzyme [EC:3.2.1.196] | "
				KEGG | ko00500 Starch and sucrose metabolism | K02750 PTS system, alpha-glucoside-specific IIC component | "
				KEGG | ko00500 Starch and sucrose metabolism | K02759 PTS system, cellobiose-specific IIA component [EC:2.7.1.196 2.7.1.205] | "
				KEGG | ko00500 Starch and sucrose metabolism | K02761 PTS system, cellobiose-specific IIC component | "
				KEGG | ko00500 Starch and sucrose metabolism | K02819 PTS system, trehalose-specific IIC component | "
				KEGG | ko00500 Starch and sucrose metabolism | K05343 maltose alpha-D-glucosyltransferase / alpha-amylase [EC:5.4.99.16 3.2.1.1] | "
				KEGG | ko00500 Starch and sucrose metabolism | K05349 beta-glucosidase [EC:3.2.1.21] | "
				KEGG | ko00500 Starch and sucrose metabolism | K05350 beta-glucosidase [EC:3.2.1.21] | "
				KEGG | ko00500 Starch and sucrose metabolism | K07405 alpha-amylase [EC:3.2.1.1] | "
				KEGG | ko00500 Starch and sucrose metabolism | K16147 starch synthase (maltosyl-transferring) [EC:2.4.99.16] | "
				KEGG | ko00500 Starch and sucrose metabolism | K16148 alpha-maltose-1-phosphate synthase [EC:2.4.1.342] | "
				KEGG | ko00620 Pyruvate metabolism | K00027 malate dehydrogenase (oxaloacetate-decarboxylating) [EC:1.1.1.38] | "
				KEGG | ko00620 Pyruvate metabolism | K00029 malate dehydrogenase (oxaloacetate-decarboxylating)(NADP+) [EC:1.1.1.40] | "
				KEGG | ko00620 Pyruvate metabolism | K00156 pyruvate dehydrogenase (quinone) [EC:1.2.5.1] | "
				KEGG | ko00620 Pyruvate metabolism | K00625 phosphate acetyltransferase [EC:2.3.1.8] | "
				KEGG | ko00620 Pyruvate metabolism | K00656 formate C-acetyltransferase [EC:2.3.1.54] | "
				KEGG | ko00620 Pyruvate metabolism | K00925 acetate kinase [EC:2.7.2.1] | "
				KEGG | ko00620 Pyruvate metabolism | K01006 pyruvate, orthophosphate dikinase [EC:2.7.9.1] | "
				KEGG | ko00620 Pyruvate metabolism | K01007 pyruvate, water dikinase [EC:2.7.9.2] | "
				KEGG | ko00620 Pyruvate metabolism | K01069 hydroxyacylglutathione hydrolase [EC:3.1.2.6] | "
				KEGG | ko00620 Pyruvate metabolism | K01572 oxaloacetate decarboxylase, beta subunit [EC:4.1.1.3] | "
				KEGG | ko00620 Pyruvate metabolism | K01573 oxaloacetate decarboxylase, gamma subunit [EC:4.1.1.3] | "
				KEGG | ko00620 Pyruvate metabolism | K01595 phosphoenolpyruvate carboxylase [EC:4.1.1.31] | "
				KEGG | ko00620 Pyruvate metabolism | K01638 malate synthase [EC:2.3.3.9] | "
				KEGG | ko00620 Pyruvate metabolism | K01649 2-isopropylmalate synthase [EC:2.3.3.13] | "
				KEGG | ko00620 Pyruvate metabolism | K01759 lactoylglutathione lyase [EC:4.4.1.5] | "
				KEGG | ko00620 Pyruvate metabolism | K01961 acetyl-CoA carboxylase, biotin carboxylase subunit [EC:6.4.1.2 6.3.4.14] | "
				KEGG | ko00620 Pyruvate metabolism | K03778 D-lactate dehydrogenase [EC:1.1.1.28] | "
				KEGG | ko00620 Pyruvate metabolism | K04073 acetaldehyde dehydrogenase [EC:1.2.1.10] | "
				KEGG | ko00620 Pyruvate metabolism | K11263 acetyl-CoA/propionyl-CoA carboxylase, biotin carboxylase, biotin carboxyl carrier protein [EC:6.4.1.2 6.4.1.3 6.3.4.14] | "
				KEGG | ko00620 Pyruvate metabolism | K12972 glyoxylate/hydroxypyruvatereductase [EC:1.1.1.79 1.1.1.81] | "
				KEGG | ko00620 Pyruvate metabolism | K13788 phosphate acetyltransferase [EC:2.3.1.8] | "
			Propionate Metabolism	KEGG | ko00640 Propanoate metabolism | K01734 methylglyoxal synthase [EC:4.2.3.3] | "
				KEGG | ko00640 Propanoate metabolism | K01782 3-hydroxyacyl-CoA dehydrogenase / enoyl-CoA hydratase / 3-hydroxybutyryl-CoA epimerase [EC:1.1.1.35 4.2.1.17 5.1.2.3] | "
				KEGG | ko00640 Propanoate metabolism | K01825 3-hydroxyacyl-CoA dehydrogenase / enoyl-CoA hydratase / 3-hydroxybutyryl-CoA epimerase / enoyl-CoA isomerase [EC:1.1.1.35 4.2.1.17 5.1.2.3 5.3.3.8] | "
				KEGG | ko00640 Propanoate metabolism | K08325 NADP-dependent alcohol dehydrogenase [EC:1.1.-.-] | "
				KEGG | ko00640 Propanoate metabolism | K11381 2-oxoisovalerate dehydrogenase E1 component [EC:1.2.4.4] | "
				KEGG | ko00640 Propanoate metabolism | K18369 alcohol dehydrogenase [EC:1.1.1.-] | "
			Butyrate Metabolism	KEGG | ko00650 Butanoate metabolism | K00135 succinate-semialdehyde dehydrogenase / glutarate-semialdehyde dehydrogenase [EC:1.2.1.16 1.2.1.79 1.2.1.20] | "
				KEGG | ko00650 Butanoate metabolism | K00634 phosphate butyryltransferase [EC:2.3.1.19] | "
				KEGG | ko00650 Butanoate metabolism | K00929 butyrate kinase [EC:2.7.2.7] | "
				KEGG | ko00650 Butanoate metabolism | K01575 acetolactate decarboxylase [EC:4.1.1.5] | "
				KEGG | ko00650 Butanoate metabolism | K01580 glutamate decarboxylase [EC:4.1.1.15] | "
				KEGG | ko00650 Butanoate metabolism | K01652 acetolactate synthase I/II/III large subunit [EC:2.2.1.6] | "
				KEGG | ko00650 Butanoate metabolism | K01653 acetolactate synthase I/III small subunit [EC:2.2.1.6] | "
				KEGG | ko00650 Butanoate metabolism | K03366 meso-butanediol dehydrogenase / (S,S)-butanediol dehydrogenase / diacetyl reductase [EC:1.1.1.- 1.1.1.76 1.1.1.304] | "
Firmicutes	*Lachnospiraceae*	*Blautia producta*	Glycolysis-associated Metabolism	KEGG | ko00010 Glycolysis / Gluconeogenesis | K00001 alcohol dehydrogenase [EC:1.1.1.1] | "
				KEGG | ko00010 Glycolysis / Gluconeogenesis | K00016 L-lactate dehydrogenase [EC:1.1.1.27] | "
				KEGG | ko00010 Glycolysis / Gluconeogenesis | K00128 aldehyde dehydrogenase (NAD+) [EC:1.2.1.3] | "
				KEGG | ko00010 Glycolysis / Gluconeogenesis | K00129 aldehyde dehydrogenase (NAD(P)+) [EC:1.2.1.5] | "
				KEGG | ko00010 Glycolysis / Gluconeogenesis | K00134 glyceraldehyde 3-phosphate dehydrogenase [EC:1.2.1.12] | "
				KEGG | ko00010 Glycolysis / Gluconeogenesis | K00169 pyruvate ferredoxin oxidoreductase alpha subunit [EC:1.2.7.1] | "
				KEGG | ko00010 Glycolysis / Gluconeogenesis | K00172 pyruvate ferredoxin oxidoreductase gamma subunit [EC:1.2.7.1] | "
				KEGG | ko00010 Glycolysis / Gluconeogenesis | K00174 2-oxoglutarate/2-oxoacid ferredoxin oxidoreductase subunit alpha [EC:1.2.7.3 1.2.7.11] | "
				KEGG | ko00010 Glycolysis / Gluconeogenesis | K00175 2-oxoglutarate/2-oxoacid ferredoxin oxidoreductase subunit beta [EC:1.2.7.3 1.2.7.11] | "
				KEGG | ko00010 Glycolysis / Gluconeogenesis | K00845 glucokinase [EC:2.7.1.2] | "
				KEGG | ko00010 Glycolysis / Gluconeogenesis | K00850 6-phosphofructokinase 1 [EC:2.7.1.11] | "
				KEGG | ko00010 Glycolysis / Gluconeogenesis | K00873 pyruvate kinase [EC:2.7.1.40] | "
				KEGG | ko00010 Glycolysis / Gluconeogenesis | K00927 phosphoglycerate kinase [EC:2.7.2.3] | "
				KEGG | ko00010 Glycolysis / Gluconeogenesis | K01222 6-phospho-beta-glucosidase [EC:3.2.1.86] | "
				KEGG | ko00010 Glycolysis / Gluconeogenesis | K01223 6-phospho-beta-glucosidase [EC:3.2.1.86] | "
				KEGG | ko00010 Glycolysis / Gluconeogenesis | K01623 fructose-bisphosphate aldolase, class I [EC:4.1.2.13] | "
				KEGG | ko00010 Glycolysis / Gluconeogenesis | K01624 fructose-bisphosphate aldolase, class II [EC:4.1.2.13] | "
				KEGG | ko00010 Glycolysis / Gluconeogenesis | K01689 enolase [EC:4.2.1.11] | "
				KEGG | ko00010 Glycolysis / Gluconeogenesis | K01803 triosephosphate isomerase (TIM) [EC:5.3.1.1] | "
				KEGG | ko00010 Glycolysis / Gluconeogenesis | K01810 glucose-6-phosphate isomerase [EC:5.3.1.9] | "
				KEGG | ko00010 Glycolysis / Gluconeogenesis | K01835 phosphoglucomutase [EC:5.4.2.2] | "
				KEGG | ko00010 Glycolysis / Gluconeogenesis | K02779 PTS system, glucose-specific IIC component | "
				KEGG | ko00010 Glycolysis / Gluconeogenesis | K03737 pyruvate-ferredoxin/flavodoxin oxidoreductase [EC:1.2.7.1 1.2.7.-] | "
				KEGG | ko00010 Glycolysis / Gluconeogenesis | K04041 fructose-1,6-bisphosphatase III [EC:3.1.3.11] | "
				KEGG | ko00010 Glycolysis / Gluconeogenesis | K04072 acetaldehyde dehydrogenase / alcohol dehydrogenase [EC:1.2.1.10 1.1.1.1] | "
				KEGG | ko00010 Glycolysis / Gluconeogenesis | K15633 2,3-bisphosphoglycerate-independent phosphoglycerate mutase [EC:5.4.2.12] | "
				KEGG | ko00020 Citrate cycle (TCA cycle) | K00031 isocitrate dehydrogenase [EC:1.1.1.42] | "
				KEGG | ko00020 Citrate cycle (TCA cycle) | K00176 2-oxoglutarate ferredoxin oxidoreductase subunit delta [EC:1.2.7.3] | "
				KEGG | ko00020 Citrate cycle (TCA cycle) | K00177 2-oxoglutarate ferredoxin oxidoreductase subunit gamma [EC:1.2.7.3] | "
				KEGG | ko00020 Citrate cycle (TCA cycle) | K00244 fumarate reductase flavoprotein subunit [EC:1.3.5.4] | "
				KEGG | ko00020 Citrate cycle (TCA cycle) | K01647 citrate synthase [EC:2.3.3.1] | "
				KEGG | ko00020 Citrate cycle (TCA cycle) | K01677 fumarate hydratase subunit alpha [EC:4.2.1.2] | "
				KEGG | ko00020 Citrate cycle (TCA cycle) | K01678 fumarate hydratase subunit beta [EC:4.2.1.2] | "
				KEGG | ko00020 Citrate cycle (TCA cycle) | K01681 aconitate hydratase [EC:4.2.1.3] | "
				KEGG | ko00020 Citrate cycle (TCA cycle) | K01903 succinyl-CoA synthetase beta subunit [EC:6.2.1.5] | "
				KEGG | ko00020 Citrate cycle (TCA cycle) | K01958 pyruvate carboxylase [EC:6.4.1.1] | "
				KEGG | ko00030 Pentose phosphate pathway | K00033 6-phosphogluconate dehydrogenase [EC:1.1.1.44 1.1.1.343] | "
				KEGG | ko00030 Pentose phosphate pathway | K00615 transketolase [EC:2.2.1.1] | "
				KEGG | ko00030 Pentose phosphate pathway | K00616 transaldolase [EC:2.2.1.2] | "
				KEGG | ko00030 Pentose phosphate pathway | K00852 ribokinase [EC:2.7.1.15] | "
				KEGG | ko00030 Pentose phosphate pathway | K00874 2-dehydro-3-deoxygluconokinase [EC:2.7.1.45] | "
				KEGG | ko00030 Pentose phosphate pathway | K00948 ribose-phosphate pyrophosphokinase [EC:2.7.6.1] | "
				KEGG | ko00030 Pentose phosphate pathway | K01619 deoxyribose-phosphate aldolase [EC:4.1.2.4] | "
				KEGG | ko00030 Pentose phosphate pathway | K01621 xylulose-5-phosphate/fructose-6-phosphate phosphoketolase [EC:4.1.2.9 4.1.2.22] | "
				KEGG | ko00030 Pentose phosphate pathway | K01625 2-dehydro-3-deoxyphosphogluconate aldolase / (4S)-4-hydroxy-2-oxoglutarate aldolase [EC:4.1.2.14 4.1.3.42] | "
				KEGG | ko00030 Pentose phosphate pathway | K01783 ribulose-phosphate 3-epimerase [EC:5.1.3.1] | "
				KEGG | ko00030 Pentose phosphate pathway | K01807 ribose 5-phosphate isomerase A [EC:5.3.1.6] | "
				KEGG | ko00030 Pentose phosphate pathway | K01808 ribose 5-phosphate isomerase B [EC:5.3.1.6] | "
				KEGG | ko00030 Pentose phosphate pathway | K01839 phosphopentomutase [EC:5.4.2.7] | "
				KEGG | ko00030 Pentose phosphate pathway | K07404 6-phosphogluconolactonase [EC:3.1.1.31] | "
				KEGG | ko00500 Starch and sucrose metabolism | K00688 glycogen phosphorylase [EC:2.4.1.1] | "
				KEGG | ko00500 Starch and sucrose metabolism | K00690 sucrose phosphorylase [EC:2.4.1.7] | "
				KEGG | ko00500 Starch and sucrose metabolism | K00700 1,4-alpha-glucan branching enzyme [EC:2.4.1.18] | "
				KEGG | ko00500 Starch and sucrose metabolism | K00703 starch synthase [EC:2.4.1.21] | "
				KEGG | ko00500 Starch and sucrose metabolism | K00705 4-alpha-glucanotransferase [EC:2.4.1.25] | "
				KEGG | ko00500 Starch and sucrose metabolism | K00975 glucose-1-phosphate adenylyltransferase [EC:2.7.7.27] | "
				KEGG | ko00500 Starch and sucrose metabolism | K01176 alpha-amylase [EC:3.2.1.1] | "
				KEGG | ko00500 Starch and sucrose metabolism | K01188 beta-glucosidase [EC:3.2.1.21] | "
				KEGG | ko00500 Starch and sucrose metabolism | K01232 maltose-6'-phosphate glucosidase [EC:3.2.1.122] | "
				KEGG | ko00500 Starch and sucrose metabolism | K01838 beta-phosphoglucomutase [EC:5.4.2.6] | "
				KEGG | ko00500 Starch and sucrose metabolism | K02438 glycogen debranching enzyme [EC:3.2.1.196] | "
				KEGG | ko00500 Starch and sucrose metabolism | K02750 PTS system, alpha-glucoside-specific IIC component | "
				KEGG | ko00500 Starch and sucrose metabolism | K02810 PTS system, sucrose-specific IIC component | "
				KEGG | ko00500 Starch and sucrose metabolism | K05341 amylosucrase [EC:2.4.1.4] | "
				KEGG | ko00500 Starch and sucrose metabolism | K05342 alpha,alpha-trehalose phosphorylase [EC:2.4.1.64] | "
				KEGG | ko00500 Starch and sucrose metabolism | K05349 beta-glucosidase [EC:3.2.1.21] | "
				KEGG | ko00500 Starch and sucrose metabolism | K05350 beta-glucosidase [EC:3.2.1.21] | "
				KEGG | ko00620 Pyruvate metabolism | K00027 malate dehydrogenase (oxaloacetate-decarboxylating) [EC:1.1.1.38] | "
				KEGG | ko00620 Pyruvate metabolism | K00156 pyruvate dehydrogenase (quinone) [EC:1.2.5.1] | "
				KEGG | ko00620 Pyruvate metabolism | K00656 formate C-acetyltransferase [EC:2.3.1.54] | "
				KEGG | ko00620 Pyruvate metabolism | K00925 acetate kinase [EC:2.7.2.1] | "
				KEGG | ko00620 Pyruvate metabolism | K01006 pyruvate, orthophosphate dikinase [EC:2.7.9.1] | "
				KEGG | ko00620 Pyruvate metabolism | K01007 pyruvate, water dikinase [EC:2.7.9.2] | "
				KEGG | ko00620 Pyruvate metabolism | K01571 oxaloacetate decarboxylase, alpha subunit [EC:4.1.1.3] | "
				KEGG | ko00620 Pyruvate metabolism | K01572 oxaloacetate decarboxylase, beta subunit [EC:4.1.1.3] | "
				KEGG | ko00620 Pyruvate metabolism | K01759 lactoylglutathione lyase [EC:4.4.1.5] | "
				KEGG | ko00620 Pyruvate metabolism | K01961 acetyl-CoA carboxylase, biotin carboxylase subunit [EC:6.4.1.2 6.3.4.14] | "
				KEGG | ko00620 Pyruvate metabolism | K01962 acetyl-CoA carboxylase carboxyl transferase subunit alpha [EC:6.4.1.2] | "
				KEGG | ko00620 Pyruvate metabolism | K01963 acetyl-CoA carboxylase carboxyl transferase subunit beta [EC:6.4.1.2] | "
				KEGG | ko00620 Pyruvate metabolism | K03778 D-lactate dehydrogenase [EC:1.1.1.28] | "
			Propionate Metabolism	KEGG | ko00640 Propanoate metabolism | K00005 glycerol dehydrogenase [EC:1.1.1.6] | "
				KEGG | ko00640 Propanoate metabolism | K00140 malonate-semialdehyde dehydrogenase (acetylating) / methylmalonate-semialdehyde dehydrogenase [EC:1.2.1.18 1.2.1.27] | "
				KEGG | ko00640 Propanoate metabolism | K01734 methylglyoxal synthase [EC:4.2.3.3] | "
				KEGG | ko00640 Propanoate metabolism | K18369 alcohol dehydrogenase [EC:1.1.1.-] | "
				KEGG | ko00640 Propanoate metabolism | K18471 methylglyoxal reductase [EC:1.1.1.-] | "
			Butyrate Metabolism	KEGG | ko00650 Butanoate metabolism | K00074 3-hydroxybutyryl-CoA dehydrogenase [EC:1.1.1.157] | "
				KEGG | ko00650 Butanoate metabolism | K00209 enoyl-[acyl-carrier protein] reductase / trans-2-enoyl-CoA reductase (NAD+) [EC:1.3.1.9 1.3.1.44] | "
				KEGG | ko00650 Butanoate metabolism | K00929 butyrate kinase [EC:2.7.2.7] | "
				KEGG | ko00650 Butanoate metabolism | K01580 glutamate decarboxylase [EC:4.1.1.15] | "
				KEGG | ko00650 Butanoate metabolism | K01652 acetolactate synthase I/II/III large subunit [EC:2.2.1.6] | "
				KEGG | ko00650 Butanoate metabolism | K01653 acetolactate synthase I/III small subunit [EC:2.2.1.6] | "

Firmicutes	*Lachnospiraceae*	*Blautia wexlerae*	Glycolysis-associated Metabolism	KEGG | ko00010 Glycolysis / Gluconeogenesis | K00128 aldehyde dehydrogenase (NAD+) [EC:1.2.1.3] | "
				KEGG | ko00010 Glycolysis / Gluconeogenesis | K00134 glyceraldehyde 3-phosphate dehydrogenase [EC:1.2.1.12] | "
				KEGG | ko00010 Glycolysis / Gluconeogenesis | K00162 pyruvate dehydrogenase E1 component beta subunit [EC:1.2.4.1] | "
				KEGG | ko00010 Glycolysis / Gluconeogenesis | K00850 6-phosphofructokinase 1 [EC:2.7.1.11] | "
				KEGG | ko00010 Glycolysis / Gluconeogenesis | K00873 pyruvate kinase [EC:2.7.1.40] | "
				KEGG | ko00010 Glycolysis / Gluconeogenesis | K00927 phosphoglycerate kinase [EC:2.7.2.3] | "
				KEGG | ko00010 Glycolysis / Gluconeogenesis | K01222 6-phospho-beta-glucosidase [EC:3.2.1.86] | "
				KEGG | ko00010 Glycolysis / Gluconeogenesis | K01223 6-phospho-beta-glucosidase [EC:3.2.1.86] | "
				KEGG | ko00010 Glycolysis / Gluconeogenesis | K01610 phosphoenolpyruvate carboxykinase (ATP) [EC:4.1.1.49] | "
				KEGG | ko00010 Glycolysis / Gluconeogenesis | K01624 fructose-bisphosphate aldolase, class II [EC:4.1.2.13] | "
				KEGG | ko00010 Glycolysis / Gluconeogenesis | K01689 enolase [EC:4.2.1.11] | "
				KEGG | ko00010 Glycolysis / Gluconeogenesis | K01785 aldose 1-epimerase [EC:5.1.3.3] | "
				KEGG | ko00010 Glycolysis / Gluconeogenesis | K01803 triosephosphate isomerase (TIM) [EC:5.3.1.1] | "
				KEGG | ko00010 Glycolysis / Gluconeogenesis | K01810 glucose-6-phosphate isomerase [EC:5.3.1.9] | "
				KEGG | ko00010 Glycolysis / Gluconeogenesis | K01834 2,3-bisphosphoglycerate-dependent phosphoglycerate mutase [EC:5.4.2.11] | "
				KEGG | ko00010 Glycolysis / Gluconeogenesis | K01835 phosphoglucomutase [EC:5.4.2.2] | "
				KEGG | ko00010 Glycolysis / Gluconeogenesis | K01895 acetyl-CoA synthetase [EC:6.2.1.1] | "
				KEGG | ko00010 Glycolysis / Gluconeogenesis | K03737 pyruvate-ferredoxin/flavodoxin oxidoreductase [EC:1.2.7.1 1.2.7.-] | "
				KEGG | ko00010 Glycolysis / Gluconeogenesis | K04041 fructose-1,6-bisphosphatase III [EC:3.1.3.11] | "
				KEGG | ko00010 Glycolysis / Gluconeogenesis | K04072 acetaldehyde dehydrogenase / alcohol dehydrogenase [EC:1.2.1.10 1.1.1.1] | "
				KEGG | ko00010 Glycolysis / Gluconeogenesis | K13954 alcohol dehydrogenase [EC:1.1.1.1] | "
				KEGG | ko00010 Glycolysis / Gluconeogenesis | K15634 probable phosphoglycerate mutase [EC:5.4.2.12] | "
				KEGG | ko00020 Citrate cycle (TCA cycle) | K00031 isocitrate dehydrogenase [EC:1.1.1.42] | "
				KEGG | ko00020 Citrate cycle (TCA cycle) | K01647 citrate synthase [EC:2.3.3.1] | "
				KEGG | ko00020 Citrate cycle (TCA cycle) | K01677 fumarate hydratase subunit alpha [EC:4.2.1.2] | "
				KEGG | ko00020 Citrate cycle (TCA cycle) | K01681 aconitate hydratase [EC:4.2.1.3] | "
				KEGG | ko00030 Pentose phosphate pathway | K00036 glucose-6-phosphate 1-dehydrogenase [EC:1.1.1.49 1.1.1.363] | "
				KEGG | ko00030 Pentose phosphate pathway | K00615 transketolase [EC:2.2.1.1] | "
				KEGG | ko00030 Pentose phosphate pathway | K00948 ribose-phosphate pyrophosphokinase [EC:2.7.6.1] | "
				KEGG | ko00030 Pentose phosphate pathway | K01621 xylulose-5-phosphate/fructose-6-phosphate phosphoketolase [EC:4.1.2.9 4.1.2.22] | "
				KEGG | ko00030 Pentose phosphate pathway | K01625 2-dehydro-3-deoxyphosphogluconate aldolase / (4S)-4-hydroxy-2-oxoglutarate aldolase [EC:4.1.2.14 4.1.3.42] | "
				KEGG | ko00030 Pentose phosphate pathway | K01783 ribulose-phosphate 3-epimerase [EC:5.1.3.1] | "
				KEGG | ko00030 Pentose phosphate pathway | K01808 ribose 5-phosphate isomerase B [EC:5.3.1.6] | "
				KEGG | ko00030 Pentose phosphate pathway | K07404 6-phosphogluconolactonase [EC:3.1.1.31] | "
				KEGG | ko00500 Starch and sucrose metabolism | K00688 glycogen phosphorylase [EC:2.4.1.1] | "
				KEGG | ko00500 Starch and sucrose metabolism | K00690 sucrose phosphorylase [EC:2.4.1.7] | "
				KEGG | ko00500 Starch and sucrose metabolism | K00700 1,4-alpha-glucan branching enzyme [EC:2.4.1.18] | "
				KEGG | ko00500 Starch and sucrose metabolism | K00702 cellobiose phosphorylase [EC:2.4.1.20] | "
				KEGG | ko00500 Starch and sucrose metabolism | K00703 starch synthase [EC:2.4.1.21] | "
				KEGG | ko00500 Starch and sucrose metabolism | K00705 4-alpha-glucanotransferase [EC:2.4.1.25] | "
				KEGG | ko00500 Starch and sucrose metabolism | K00975 glucose-1-phosphate adenylyltransferase [EC:2.7.7.27] | "
				KEGG | ko00500 Starch and sucrose metabolism | K00978 glucose-1-phosphate cytidylyltransferase [EC:2.7.7.33] | "
				KEGG | ko00500 Starch and sucrose metabolism | K01176 alpha-amylase [EC:3.2.1.1] | "
				KEGG | ko00500 Starch and sucrose metabolism | K02438 glycogen debranching enzyme [EC:3.2.1.196] | "
				KEGG | ko00500 Starch and sucrose metabolism | K02759 PTS system, cellobiose-specific IIA component [EC:2.7.1.196 2.7.1.205] | "
				KEGG | ko00500 Starch and sucrose metabolism | K02760 PTS system, cellobiose-specific IIB component [EC:2.7.1.196 2.7.1.205] | "
				KEGG | ko00500 Starch and sucrose metabolism | K02761 PTS system, cellobiose-specific IIC component | "
				KEGG | ko00500 Starch and sucrose metabolism | K05341 amylosucrase [EC:2.4.1.4] | "
				KEGG | ko00500 Starch and sucrose metabolism | K05343 maltose alpha-D-glucosyltransferase / alpha-amylase [EC:5.4.99.16 3.2.1.1] | "
				KEGG | ko00500 Starch and sucrose metabolism | K05349 beta-glucosidase [EC:3.2.1.21] | "
				KEGG | ko00620 Pyruvate metabolism | K00625 phosphate acetyltransferase [EC:2.3.1.8] | "
				KEGG | ko00620 Pyruvate metabolism | K00656 formate C-acetyltransferase [EC:2.3.1.54] | "
				KEGG | ko00620 Pyruvate metabolism | K00925 acetate kinase [EC:2.7.2.1] | "
				KEGG | ko00620 Pyruvate metabolism | K01006 pyruvate, orthophosphate dikinase [EC:2.7.9.1] | "
				KEGG | ko00620 Pyruvate metabolism | K01571 oxaloacetate decarboxylase, alpha subunit [EC:4.1.1.3] | "
				KEGG | ko00620 Pyruvate metabolism | K01572 oxaloacetate decarboxylase, beta subunit [EC:4.1.1.3] | "
				KEGG | ko00620 Pyruvate metabolism | K01595 phosphoenolpyruvate carboxylase [EC:4.1.1.31] | "
				KEGG | ko00620 Pyruvate metabolism | K01649 2-isopropylmalate synthase [EC:2.3.3.13] | "
				KEGG | ko00620 Pyruvate metabolism | K01961 acetyl-CoA carboxylase, biotin carboxylase subunit [EC:6.4.1.2 6.3.4.14] | "
				KEGG | ko00620 Pyruvate metabolism | K01962 acetyl-CoA carboxylase carboxyl transferase subunit alpha [EC:6.4.1.2] | "
				KEGG | ko00620 Pyruvate metabolism | K01963 acetyl-CoA carboxylase carboxyl transferase subunit beta [EC:6.4.1.2] | "
				KEGG | ko00620 Pyruvate metabolism | K02160 acetyl-CoA carboxylase biotin carboxyl carrier protein | "
				KEGG | ko00620 Pyruvate metabolism | K03778 D-lactate dehydrogenase [EC:1.1.1.28] | "
				KEGG | ko00620 Pyruvate metabolism | K11263 acetyl-CoA/propionyl-CoA carboxylase, biotin carboxylase, biotin carboxyl carrier protein [EC:6.4.1.2 6.4.1.3 6.3.4.14] | "
				KEGG | ko00620 Pyruvate metabolism | K13788 phosphate acetyltransferase [EC:2.3.1.8] | "
			Propionate Metabolism	KEGG | ko00640 Propanoate metabolism | K07250 4-aminobutyrate aminotransferase / (S)-3-amino-2-methylpropionate transaminase [EC:2.6.1.19 2.6.1.22] | "
				KEGG | ko00640 Propanoate metabolism | K13922 propionaldehyde dehydrogenase [EC:1.2.1.87] | "
			Butyrate Metabolism	KEGG | ko00650 Butanoate metabolism | K00209 enoyl-[acyl-carrier protein] reductase / trans-2-enoyl-CoA reductase (NAD+) [EC:1.3.1.9 1.3.1.44] | "
				KEGG | ko00650 Butanoate metabolism | K01652 acetolactate synthase I/II/III large subunit [EC:2.2.1.6] | "
				KEGG | ko00650 Butanoate metabolism | K01653 acetolactate synthase I/III small subunit [EC:2.2.1.6] | "
				KEGG | ko00650 Butanoate metabolism | K18120 4-hydroxybutyrate dehydrogenase [EC:1.1.1.61] | "
Firmicutes	*Eubacteriaceae*	*Eubacterium hallii*	Glycolysis-associated Metabolism	KEGG | ko00010 Glycolysis / Gluconeogenesis | K00128 aldehyde dehydrogenase (NAD+) [EC:1.2.1.3] | "
				KEGG | ko00010 Glycolysis / Gluconeogenesis | K00134 glyceraldehyde 3-phosphate dehydrogenase [EC:1.2.1.12] | "
				KEGG | ko00010 Glycolysis / Gluconeogenesis | K00850 6-phosphofructokinase 1 [EC:2.7.1.11] | "
				KEGG | ko00010 Glycolysis / Gluconeogenesis | K01834 2,3-bisphosphoglycerate-dependent phosphoglycerate mutase [EC:5.4.2.11] | "
				KEGG | ko00010 Glycolysis / Gluconeogenesis | K01895 acetyl-CoA synthetase [EC:6.2.1.1] | "
				KEGG | ko00020 Citrate cycle (TCA cycle) | K00240 succinate dehydrogenase / fumarate reductase, iron-sulfur subunit [EC:1.3.5.1 1.3.5.4] | "
				KEGG | ko00020 Citrate cycle (TCA cycle) | K01958 pyruvate carboxylase [EC:6.4.1.1] | "
				KEGG | ko00030 Pentose phosphate pathway | K00615 transketolase [EC:2.2.1.1] | "
				KEGG | ko00030 Pentose phosphate pathway | K01690 phosphogluconate dehydratase [EC:4.2.1.12] | "
				KEGG | ko00030 Pentose phosphate pathway | K01807 ribose 5-phosphate isomerase A [EC:5.3.1.6] | "
				KEGG | ko00500 Starch and sucrose metabolism | K00975 glucose-1-phosphate adenylyltransferase [EC:2.7.7.27] | "
				KEGG | ko00500 Starch and sucrose metabolism | K02761 PTS system, cellobiose-specific IIC component | "
				KEGG | ko00500 Starch and sucrose metabolism | K05349 beta-glucosidase [EC:3.2.1.21] | "
				KEGG | ko00620 Pyruvate metabolism | K01595 phosphoenolpyruvate carboxylase [EC:4.1.1.31] | "
				KEGG | ko00620 Pyruvate metabolism | K03778 D-lactate dehydrogenase [EC:1.1.1.28] | "
			Propionate Metabolism	KEGG | ko00640 Propanoate metabolism | K00005 glycerol dehydrogenase [EC:1.1.1.6] | "
			Butyrate Metabolism	KEGG | ko00650 Butanoate metabolism | K00248 butyryl-CoA dehydrogenase [EC:1.3.8.1] | "
				KEGG | ko00650 Butanoate metabolism | K01652 acetolactate synthase I/II/III large subunit [EC:2.2.1.6] | "
				KEGG | ko00650 Butanoate metabolism | K01653 acetolactate synthase I/III small subunit [EC:2.2.1.6] | "
				KEGG | ko00650 Butanoate metabolism | K03366 meso-butanediol dehydrogenase / (S,S)-butanediol dehydrogenase / diacetyl reductase [EC:1.1.1.- 1.1.1.76 1.1.1.304] | "
		Eubacterium limosum	Glycolysis-associated Metabolism	KEGG | ko00010 Glycolysis / Gluconeogenesis | K00169 pyruvate ferredoxin oxidoreductase alpha subunit [EC:1.2.7.1] | "
				KEGG | ko00010 Glycolysis / Gluconeogenesis | K00850 6-phosphofructokinase 1 [EC:2.7.1.11] | "
				KEGG | ko00010 Glycolysis / Gluconeogenesis | K01222 6-phospho-beta-glucosidase [EC:3.2.1.86] | "
				KEGG | ko00010 Glycolysis / Gluconeogenesis | K01223 6-phospho-beta-glucosidase [EC:3.2.1.86] | "
				KEGG | ko00010 Glycolysis / Gluconeogenesis | K15634 probable phosphoglycerate mutase [EC:5.4.2.12] | "
				KEGG | ko00030 Pentose phosphate pathway | K00615 transketolase [EC:2.2.1.1] | "
				KEGG | ko00030 Pentose phosphate pathway | K00616 transaldolase [EC:2.2.1.2] | "
				KEGG | ko00030 Pentose phosphate pathway | K01625 2-dehydro-3-deoxyphosphogluconate aldolase / (4S)-4-hydroxy-2-oxoglutarate aldolase [EC:4.1.2.14 4.1.3.42] | "
				KEGG | ko00030 Pentose phosphate pathway | K01808 ribose 5-phosphate isomerase B [EC:5.3.1.6] | "
				KEGG | ko00500 Starch and sucrose metabolism | K00705 4-alpha-glucanotransferase [EC:2.4.1.25] | "
				KEGG | ko00500 Starch and sucrose metabolism | K01226 trehalose-6-phosphate hydrolase [EC:3.2.1.93] | "
				KEGG | ko00500 Starch and sucrose metabolism | K02760 PTS system, cellobiose-specific IIB component [EC:2.7.1.196 2.7.1.205] | "
				KEGG | ko00500 Starch and sucrose metabolism | K02810 PTS system, sucrose-specific IIC component | "
				KEGG | ko00500 Starch and sucrose metabolism | K05343 maltose alpha-D-glucosyltransferase / alpha-amylase [EC:5.4.99.16 3.2.1.1] | "
				KEGG | ko00500 Starch and sucrose metabolism | K05350 beta-glucosidase [EC:3.2.1.21] | "
				KEGG | ko00620 Pyruvate metabolism | K00656 formate C-acetyltransferase [EC:2.3.1.54] | "
				KEGG | ko00620 Pyruvate metabolism | K03778 D-lactate dehydrogenase [EC:1.1.1.28] | "
			Propionate Metabolism	KEGG | ko00640 Propanoate metabolism | K01734 methylglyoxal synthase [EC:4.2.3.3] | "
Firmicutes	*Eubacteriaceae*	*Erysipelatoclostridium ramosum*	Glycolysis-associated Metabolism	KEGG | ko00010 Glycolysis / Gluconeogenesis | K00134 glyceraldehyde 3-phosphate dehydrogenase [EC:1.2.1.12] | "
				KEGG | ko00010 Glycolysis / Gluconeogenesis | K00169 pyruvate ferredoxin oxidoreductase alpha subunit [EC:1.2.7.1] | "
				KEGG | ko00010 Glycolysis / Gluconeogenesis | K00172 pyruvate ferredoxin oxidoreductase gamma subunit [EC:1.2.7.1] | "
				KEGG | ko00010 Glycolysis / Gluconeogenesis | K00850 6-phosphofructokinase 1 [EC:2.7.1.11] | "
				KEGG | ko00010 Glycolysis / Gluconeogenesis | K00873 pyruvate kinase [EC:2.7.1.40] | "
				KEGG | ko00010 Glycolysis / Gluconeogenesis | K01222 6-phospho-beta-glucosidase [EC:3.2.1.86] | "
				KEGG | ko00010 Glycolysis / Gluconeogenesis | K01223 6-phospho-beta-glucosidase [EC:3.2.1.86] | "
				KEGG | ko00010 Glycolysis / Gluconeogenesis | K02791 PTS system, maltose/glucose-specific IIC component | "
				KEGG | ko00010 Glycolysis / Gluconeogenesis | K15634 probable phosphoglycerate mutase [EC:5.4.2.12] | "
				KEGG | ko00030 Pentose phosphate pathway | K00036 glucose-6-phosphate 1-dehydrogenase [EC:1.1.1.49 1.1.1.363] | "
				KEGG | ko00030 Pentose phosphate pathway | K00615 transketolase [EC:2.2.1.1] | "
				KEGG | ko00030 Pentose phosphate pathway | K00616 transaldolase [EC:2.2.1.2] | "
				KEGG | ko00030 Pentose phosphate pathway | K01619 deoxyribose-phosphate aldolase [EC:4.1.2.4] | "
				KEGG | ko00030 Pentose phosphate pathway | K01625 2-dehydro-3-deoxyphosphogluconate aldolase / (4S)-4-hydroxy-2-oxoglutarate aldolase [EC:4.1.2.14 4.1.3.42] | "
				KEGG | ko00030 Pentose phosphate pathway | K01808 ribose 5-phosphate isomerase B [EC:5.3.1.6] | "
				KEGG | ko00030 Pentose phosphate pathway | K01839 phosphopentomutase [EC:5.4.2.7] | "
				KEGG | ko00500 Starch and sucrose metabolism | K00705 4-alpha-glucanotransferase [EC:2.4.1.25] | "
				KEGG | ko00500 Starch and sucrose metabolism | K01226 trehalose-6-phosphate hydrolase [EC:3.2.1.93] | "
				KEGG | ko00500 Starch and sucrose metabolism | K01232 maltose-6'-phosphate glucosidase [EC:3.2.1.122] | "
				KEGG | ko00500 Starch and sucrose metabolism | K02750 PTS system, alpha-glucoside-specific IIC component | "
				KEGG | ko00500 Starch and sucrose metabolism | K02760 PTS system, cellobiose-specific IIB component [EC:2.7.1.196 2.7.1.205] | "
				KEGG | ko00500 Starch and sucrose metabolism | K02761 PTS system, cellobiose-specific IIC component | "
				KEGG | ko00500 Starch and sucrose metabolism | K02810 PTS system, sucrose-specific IIC component | "
				KEGG | ko00500 Starch and sucrose metabolism | K02819 PTS system, trehalose-specific IIC component | "
				KEGG | ko00500 Starch and sucrose metabolism | K05343 maltose alpha-D-glucosyltransferase / alpha-amylase [EC:5.4.99.16 3.2.1.1] | "
				KEGG | ko00500 Starch and sucrose metabolism | K05349 beta-glucosidase [EC:3.2.1.21] | "
				KEGG | ko00500 Starch and sucrose metabolism | K05350 beta-glucosidase [EC:3.2.1.21] | "
				KEGG | ko00620 Pyruvate metabolism | K00656 formate C-acetyltransferase [EC:2.3.1.54] | "
				KEGG | ko00620 Pyruvate metabolism | K03778 D-lactate dehydrogenase [EC:1.1.1.28] | "
			Propionate Metabolism	KEGG | ko00640 Propanoate metabolism | K01734 methylglyoxal synthase [EC:4.2.3.3] | "
Firmicutes	*Eubacteriaceae*	*Eubacterium ramulus*	Glycolysis-associated Metabolism	KEGG | ko00010 Glycolysis / Gluconeogenesis | K00128 aldehyde dehydrogenase (NAD+) [EC:1.2.1.3] | "
				KEGG | ko00010 Glycolysis / Gluconeogenesis | K00134 glyceraldehyde 3-phosphate dehydrogenase [EC:1.2.1.12] | "
				KEGG | ko00010 Glycolysis / Gluconeogenesis | K00138 aldehyde dehydrogenase [EC:1.2.1.-] | "
				KEGG | ko00010 Glycolysis / Gluconeogenesis | K00850 6-phosphofructokinase 1 [EC:2.7.1.11] | "
				KEGG | ko00010 Glycolysis / Gluconeogenesis | K01792 glucose-6-phosphate 1-epimerase [EC:5.1.3.15] | "
				KEGG | ko00010 Glycolysis / Gluconeogenesis | K01834 2,3-bisphosphoglycerate-dependent phosphoglycerate mutase [EC:5.4.2.11] | "
				KEGG | ko00010 Glycolysis / Gluconeogenesis | K01835 phosphoglucomutase [EC:5.4.2.2] | "
				KEGG | ko00010 Glycolysis / Gluconeogenesis | K01895 acetyl-CoA synthetase [EC:6.2.1.1] | "
				KEGG | ko00020 Citrate cycle (TCA cycle) | K00240 succinate dehydrogenase / fumarate reductase, iron-sulfur subunit [EC:1.3.5.1 1.3.5.4] | "
				KEGG | ko00020 Citrate cycle (TCA cycle) | K01647 citrate synthase [EC:2.3.3.1] | "
				KEGG | ko00020 Citrate cycle (TCA cycle) | K01958 pyruvate carboxylase [EC:6.4.1.1] | "
				KEGG | ko00030 Pentose phosphate pathway | K00615 transketolase [EC:2.2.1.1] | "
				KEGG | ko00030 Pentose phosphate pathway | K00948 ribose-phosphate pyrophosphokinase [EC:2.7.6.1] | "
				KEGG | ko00030 Pentose phosphate pathway | K01690 phosphogluconate dehydratase [EC:4.2.1.12] | "
				KEGG | ko00030 Pentose phosphate pathway | K01807 ribose 5-phosphate isomerase A [EC:5.3.1.6] | "
				KEGG | ko00500 Starch and sucrose metabolism | K00688 glycogen phosphorylase [EC:2.4.1.1] | "
				KEGG | ko00500 Starch and sucrose metabolism | K00975 glucose-1-phosphate adenylyltransferase [EC:2.7.7.27] | "
				KEGG | ko00500 Starch and sucrose metabolism | K02438 glycogen debranching enzyme [EC:3.2.1.196] | "
				KEGG | ko00500 Starch and sucrose metabolism | K02761 PTS system, cellobiose-specific IIC component | "
				KEGG | ko00500 Starch and sucrose metabolism | K05349 beta-glucosidase [EC:3.2.1.21] | "
				KEGG | ko00500 Starch and sucrose metabolism | K16147 starch synthase (maltosyl-transferring) [EC:2.4.99.16] | "
				KEGG | ko00620 Pyruvate metabolism | K00656 formate C-acetyltransferase [EC:2.3.1.54] | "
				KEGG | ko00620 Pyruvate metabolism | K01595 phosphoenolpyruvate carboxylase [EC:4.1.1.31] | "
				KEGG | ko00620 Pyruvate metabolism | K01649 2-isopropylmalate synthase [EC:2.3.3.13] | "
				KEGG | ko00620 Pyruvate metabolism | K03778 D-lactate dehydrogenase [EC:1.1.1.28] | "
			Propionate Metabolism	KEGG | ko00640 Propanoate metabolism | K00005 glycerol dehydrogenase [EC:1.1.1.6] | "
			Butyrate Metabolism	KEGG | ko00650 Butanoate metabolism | K00248 butyryl-CoA dehydrogenase [EC:1.3.8.1] | "
				KEGG | ko00650 Butanoate metabolism | K01652 acetolactate synthase I/II/III large subunit [EC:2.2.1.6] | "
				KEGG | ko00650 Butanoate metabolism | K01653 acetolactate synthase I/III small subunit [EC:2.2.1.6] | "
				KEGG | ko00650 Butanoate metabolism | K03366 meso-butanediol dehydrogenase / (S,S)-butanediol dehydrogenase / diacetyl reductase [EC:1.1.1.- 1.1.1.76 1.1.1.304] | "
				KEGG | ko00010 Glycolysis / Gluconeogenesis | K00016 L-lactate dehydrogenase [EC:1.1.1.27] | "
				KEGG | ko00010 Glycolysis / Gluconeogenesis | K00128 aldehyde dehydrogenase (NAD+) [EC:1.2.1.3] | "
				KEGG | ko00010 Glycolysis / Gluconeogenesis | K00134 glyceraldehyde 3-phosphate dehydrogenase [EC:1.2.1.12] | "
				KEGG | ko00010 Glycolysis / Gluconeogenesis | K00845 glucokinase [EC:2.7.1.2] | "
				KEGG | ko00010 Glycolysis / Gluconeogenesis | K00850 6-phosphofructokinase 1 [EC:2.7.1.11] | "

Firmicutes	*Ruminococcaceae*	*Faecalibacterium prausnitzii*	Glycolysis-associated Metabolism	KEGG | ko00010 Glycolysis / Gluconeogenesis | K00873 pyruvate kinase [EC:2.7.1.40] | "
				KEGG | ko00010 Glycolysis / Gluconeogenesis | K00927 phosphoglycerate kinase [EC:2.7.2.3] | "
				KEGG | ko00010 Glycolysis / Gluconeogenesis | K01223 6-phospho-beta-glucosidase [EC:3.2.1.86] | "
				KEGG | ko00010 Glycolysis / Gluconeogenesis | K01610 phosphoenolpyruvate carboxykinase (ATP) [EC:4.1.1.49] | "
				KEGG | ko00010 Glycolysis / Gluconeogenesis | K01624 fructose-bisphosphate aldolase, class II [EC:4.1.2.13] | "
				KEGG | ko00010 Glycolysis / Gluconeogenesis | K01689 enolase [EC:4.2.1.11] | "
				KEGG | ko00010 Glycolysis / Gluconeogenesis | K01785 aldose 1-epimerase [EC:5.1.3.3] | "
				KEGG | ko00010 Glycolysis / Gluconeogenesis | K01803 triosephosphate isomerase (TIM) [EC:5.3.1.1] | "
				KEGG | ko00010 Glycolysis / Gluconeogenesis | K01810 glucose-6-phosphate isomerase [EC:5.3.1.9] | "
				KEGG | ko00010 Glycolysis / Gluconeogenesis | K01835 phosphoglucomutase [EC:5.4.2.2] | "
				KEGG | ko00010 Glycolysis / Gluconeogenesis | K02777 PTS system, sugar-specific IIA component [EC:2.7.1.-] | "
				KEGG | ko00010 Glycolysis / Gluconeogenesis | K02791 PTS system, maltose/glucose-specific IIC component | "
				KEGG | ko00010 Glycolysis / Gluconeogenesis | K03737 pyruvate-ferredoxin/flavodoxin oxidoreductase [EC:1.2.7.1 1.2.7.-] | "
				KEGG | ko00010 Glycolysis / Gluconeogenesis | K04041 fructose-1,6-bisphosphatase III [EC:3.1.3.11] | "
				KEGG | ko00010 Glycolysis / Gluconeogenesis | K04072 acetaldehyde dehydrogenase / alcohol dehydrogenase [EC:1.2.1.10 1.1.1.1] | "
				KEGG | ko00010 Glycolysis / Gluconeogenesis | K13954 alcohol dehydrogenase [EC:1.1.1.1] | "
				KEGG | ko00010 Glycolysis / Gluconeogenesis | K15633 2,3-bisphosphoglycerate-independent phosphoglycerate mutase [EC:5.4.2.12] | "
				KEGG | ko00010 Glycolysis / Gluconeogenesis | K15634 probable phosphoglycerate mutase [EC:5.4.2.12] | "
				KEGG | ko00020 Citrate cycle (TCA cycle) | K00031 isocitrate dehydrogenase [EC:1.1.1.42] | "
				KEGG | ko00020 Citrate cycle (TCA cycle) | K00239 succinate dehydrogenase / fumarate reductase, flavoprotein subunit [EC:1.3.5.1 1.3.5.4] | "
				KEGG | ko00020 Citrate cycle (TCA cycle) | K00240 succinate dehydrogenase / fumarate reductase, iron-sulfur subunit [EC:1.3.5.1 1.3.5.4] | "
				KEGG | ko00020 Citrate cycle (TCA cycle) | K00244 fumarate reductase flavoprotein subunit [EC:1.3.5.4] | "
				KEGG | ko00020 Citrate cycle (TCA cycle) | K01647 citrate synthase [EC:2.3.3.1] | "
				KEGG | ko00020 Citrate cycle (TCA cycle) | K01677 fumarate hydratase subunit alpha [EC:4.2.1.2] | "
				KEGG | ko00020 Citrate cycle (TCA cycle) | K01678 fumarate hydratase subunit beta [EC:4.2.1.2] | "
				KEGG | ko00020 Citrate cycle (TCA cycle) | K01681 aconitate hydratase [EC:4.2.1.3] | "
				KEGG | ko00030 Pentose phosphate pathway | K00033 6-phosphogluconate dehydrogenase [EC:1.1.1.44 1.1.1.343] | "
				KEGG | ko00030 Pentose phosphate pathway | K00036 glucose-6-phosphate 1-dehydrogenase [EC:1.1.1.49 1.1.1.363] | "
				KEGG | ko00030 Pentose phosphate pathway | K00615 transketolase [EC:2.2.1.1] | "
				KEGG | ko00030 Pentose phosphate pathway | K00948 ribose-phosphate pyrophosphokinase [EC:2.7.6.1] | "
				KEGG | ko00030 Pentose phosphate pathway | K01619 deoxyribose-phosphate aldolase [EC:4.1.2.4] | "
				KEGG | ko00030 Pentose phosphate pathway | K01625 2-dehydro-3-deoxyphosphogluconate aldolase / (4S)-4-hydroxy-2-oxoglutarate aldolase [EC:4.1.2.14 4.1.3.42] | "
				KEGG | ko00030 Pentose phosphate pathway | K01783 ribulose-phosphate 3-epimerase [EC:5.1.3.1] | "
				KEGG | ko00030 Pentose phosphate pathway | K01808 ribose 5-phosphate isomerase B [EC:5.3.1.6] | "
				KEGG | ko00030 Pentose phosphate pathway | K01839 phosphopentomutase [EC:5.4.2.7] | "
				KEGG | ko00500 Starch and sucrose metabolism | K00688 glycogen phosphorylase [EC:2.4.1.1] | "
				KEGG | ko00500 Starch and sucrose metabolism | K00690 sucrose phosphorylase [EC:2.4.1.7] | "
				KEGG | ko00500 Starch and sucrose metabolism | K00700 1,4-alpha-glucan branching enzyme [EC:2.4.1.18] | "
				KEGG | ko00500 Starch and sucrose metabolism | K00702 cellobiose phosphorylase [EC:2.4.1.20] | "
				KEGG | ko00500 Starch and sucrose metabolism | K00703 starch synthase [EC:2.4.1.21] | "
				KEGG | ko00500 Starch and sucrose metabolism | K00705 4-alpha-glucanotransferase [EC:2.4.1.25] | "
				KEGG | ko00500 Starch and sucrose metabolism | K00975 glucose-1-phosphate adenylyltransferase [EC:2.7.7.27] | "
				KEGG | ko00500 Starch and sucrose metabolism | K01176 alpha-amylase [EC:3.2.1.1] | "
				KEGG | ko00500 Starch and sucrose metabolism | K01188 beta-glucosidase [EC:3.2.1.21] | "
				KEGG | ko00500 Starch and sucrose metabolism | K01226 trehalose-6-phosphate hydrolase [EC:3.2.1.93] | "
				KEGG | ko00500 Starch and sucrose metabolism | K01232 maltose-6'-phosphate glucosidase [EC:3.2.1.122] | "
				KEGG | ko00500 Starch and sucrose metabolism | K02438 glycogen debranching enzyme [EC:3.2.1.196] | "
				KEGG | ko00500 Starch and sucrose metabolism | K02761 PTS system, cellobiose-specific IIC component | "
				KEGG | ko00500 Starch and sucrose metabolism | K02819 PTS system, trehalose-specific IIC component | "
				KEGG | ko00500 Starch and sucrose metabolism | K05341 amylosucrase [EC:2.4.1.4] | "
				KEGG | ko00500 Starch and sucrose metabolism | K05343 maltose alpha-D-glucosyltransferase / alpha-amylase [EC:5.4.99.16 3.2.1.1] | "
				KEGG | ko00500 Starch and sucrose metabolism | K05349 beta-glucosidase [EC:3.2.1.21] | "
				KEGG | ko00500 Starch and sucrose metabolism | K05350 beta-glucosidase [EC:3.2.1.21] | "
				KEGG | ko00500 Starch and sucrose metabolism | K16147 starch synthase (maltosyl-transferring) [EC:2.4.99.16] | "
				KEGG | ko00620 Pyruvate metabolism | K00027 malate dehydrogenase (oxaloacetate-decarboxylating) [EC:1.1.1.38] | "
				KEGG | ko00620 Pyruvate metabolism | K00626 acetyl-CoA C-acetyltransferase [EC:2.3.1.9] | "
				KEGG | ko00620 Pyruvate metabolism | K00656 formate C-acetyltransferase [EC:2.3.1.54] | "
				KEGG | ko00620 Pyruvate metabolism | K00925 acetate kinase [EC:2.7.2.1] | "
				KEGG | ko00620 Pyruvate metabolism | K01006 pyruvate, orthophosphate dikinase [EC:2.7.9.1] | "
				KEGG | ko00620 Pyruvate metabolism | K01571 oxaloacetate decarboxylase, alpha subunit [EC:4.1.1.3] | "
				KEGG | ko00620 Pyruvate metabolism | K01572 oxaloacetate decarboxylase, beta subunit [EC:4.1.1.3] | "
				KEGG | ko00620 Pyruvate metabolism | K01595 phosphoenolpyruvate carboxylase [EC:4.1.1.31] | "
				KEGG | ko00620 Pyruvate metabolism | K01638 malate synthase [EC:2.3.3.9] | "
				KEGG | ko00620 Pyruvate metabolism | K01649 2-isopropylmalate synthase [EC:2.3.3.13] | "
				KEGG | ko00620 Pyruvate metabolism | K01759 lactoylglutathione lyase [EC:4.4.1.5] | "
				KEGG | ko00620 Pyruvate metabolism | K01962 acetyl-CoA carboxylase carboxyl transferase subunit alpha [EC:6.4.1.2] | "
				KEGG | ko00620 Pyruvate metabolism | K01963 acetyl-CoA carboxylase carboxyl transferase subunit beta [EC:6.4.1.2] | "
				KEGG | ko00620 Pyruvate metabolism | K03778 D-lactate dehydrogenase [EC:1.1.1.28] | "
				KEGG | ko00620 Pyruvate metabolism | K11263 acetyl-CoA/propionyl-CoA carboxylase, biotin carboxylase, biotin carboxyl carrier protein [EC:6.4.1.2 6.4.1.3 6.3.4.14] | "
				KEGG | ko00620 Pyruvate metabolism | K13788 phosphate acetyltransferase [EC:2.3.1.8] | "
				KEGG | ko00620 Pyruvate metabolism | K15024 putative phosphotransacetylase [EC:2.3.1.8] | "
			Propionate Metabolism	KEGG | ko00640 Propanoate metabolism | K01734 methylglyoxal synthase [EC:4.2.3.3] | "
				KEGG | ko00640 Propanoate metabolism | K13922 propionaldehyde dehydrogenase [EC:1.2.1.87] | "
				KEGG | ko00640 Propanoate metabolism | K18369 alcohol dehydrogenase [EC:1.1.1.-] | "
				KEGG | ko00640 Propanoate metabolism | K18471 methylglyoxal reductase [EC:1.1.1.-] | "
				KEGG | ko00640 Propanoate metabolism | K19697 propionate kinase [EC:2.7.2.15] | "
			Butyrate Metabolism	KEGG | ko00650 Butanoate metabolism | K00074 3-hydroxybutyryl-CoA dehydrogenase [EC:1.1.1.157] | "
				KEGG | ko00650 Butanoate metabolism | K00248 butyryl-CoA dehydrogenase [EC:1.3.8.1] | "
				KEGG | ko00650 Butanoate metabolism | K01640 hydroxymethylglutaryl-CoA lyase [EC:4.1.3.4] | "
				KEGG | ko00650 Butanoate metabolism | K01652 acetolactate synthase I/II/III large subunit [EC:2.2.1.6] | "
				KEGG | ko00650 Butanoate metabolism | K01653 acetolactate synthase I/III small subunit [EC:2.2.1.6] | "
Firmicutes	*Lachnospiraceae*	*Lachnospiraceae bacterium*	Glycolysis-associated Metabolism	KEGG | ko00010 Glycolysis / Gluconeogenesis | K00016 L-lactate dehydrogenase [EC:1.1.1.27] | "
				KEGG | ko00010 Glycolysis / Gluconeogenesis | K00128 aldehyde dehydrogenase (NAD+) [EC:1.2.1.3] | "
				KEGG | ko00010 Glycolysis / Gluconeogenesis | K00131 glyceraldehyde-3-phosphate dehydrogenase (NADP+) [EC:1.2.1.9] | "
				KEGG | ko00010 Glycolysis / Gluconeogenesis | K00134 glyceraldehyde 3-phosphate dehydrogenase [EC:1.2.1.12] | "
				KEGG | ko00010 Glycolysis / Gluconeogenesis | K00161 pyruvate dehydrogenase E1 component alpha subunit [EC:1.2.4.1] | "
				KEGG | ko00010 Glycolysis / Gluconeogenesis | K00163 pyruvate dehydrogenase E1 component [EC:1.2.4.1] | "
				KEGG | ko00010 Glycolysis / Gluconeogenesis | K00174 2-oxoglutarate/2-oxoacid ferredoxin oxidoreductase subunit alpha [EC:1.2.7.3 1.2.7.11] | "
				KEGG | ko00010 Glycolysis / Gluconeogenesis | K00175 2-oxoglutarate/2-oxoacid ferredoxin oxidoreductase subunit beta [EC:1.2.7.3 1.2.7.11] | "
				KEGG | ko00010 Glycolysis / Gluconeogenesis | K00382 dihydrolipoamide dehydrogenase [EC:1.8.1.4] | "
				KEGG | ko00010 Glycolysis / Gluconeogenesis | K00845 glucokinase [EC:2.7.1.2] | "
				KEGG | ko00010 Glycolysis / Gluconeogenesis | K00850 6-phosphofructokinase 1 [EC:2.7.1.11] | "
				KEGG | ko00010 Glycolysis / Gluconeogenesis | K00873 pyruvate kinase [EC:2.7.1.40] | "
				KEGG | ko00010 Glycolysis / Gluconeogenesis | K01222 6-phospho-beta-glucosidase [EC:3.2.1.86] | "
				KEGG | ko00010 Glycolysis / Gluconeogenesis | K01223 6-phospho-beta-glucosidase [EC:3.2.1.86] | "
				KEGG | ko00010 Glycolysis / Gluconeogenesis | K01610 phosphoenolpyruvate carboxykinase (ATP) [EC:4.1.1.49] | "
				KEGG | ko00010 Glycolysis / Gluconeogenesis | K01624 fructose-bisphosphate aldolase, class II [EC:4.1.2.13] | "
				KEGG | ko00010 Glycolysis / Gluconeogenesis | K01689 enolase [EC:4.2.1.11] | "
				KEGG | ko00010 Glycolysis / Gluconeogenesis | K01785 aldose 1-epimerase [EC:5.1.3.3] | "
				KEGG | ko00010 Glycolysis / Gluconeogenesis | K01803 triosephosphate isomerase (TIM) [EC:5.3.1.1] | "
				KEGG | ko00010 Glycolysis / Gluconeogenesis | K01810 glucose-6-phosphate isomerase [EC:5.3.1.9] | "
				KEGG | ko00010 Glycolysis / Gluconeogenesis | K01834 2,3-bisphosphoglycerate-dependent phosphoglycerate mutase [EC:5.4.2.11] | "
				KEGG | ko00010 Glycolysis / Gluconeogenesis | K01835 phosphoglucomutase [EC:5.4.2.2] | "
				KEGG | ko00010 Glycolysis / Gluconeogenesis | K02777 PTS system, sugar-specific IIA component [EC:2.7.1.-] | "
				KEGG | ko00010 Glycolysis / Gluconeogenesis | K02779 PTS system, glucose-specific IIC component | "
				KEGG | ko00010 Glycolysis / Gluconeogenesis | K03737 pyruvate-ferredoxin/flavodoxin oxidoreductase [EC:1.2.7.1 1.2.7.-] | "
				KEGG | ko00010 Glycolysis / Gluconeogenesis | K04041 fructose-1,6-bisphosphatase III [EC:3.1.3.11] | "
				KEGG | ko00010 Glycolysis / Gluconeogenesis | K04072 acetaldehyde dehydrogenase / alcohol dehydrogenase [EC:1.2.1.10 1.1.1.1] | "
				KEGG | ko00010 Glycolysis / Gluconeogenesis | K15633 2,3-bisphosphoglycerate-independent phosphoglycerate mutase [EC:5.4.2.12] | "
				KEGG | ko00010 Glycolysis / Gluconeogenesis | K15634 probable phosphoglycerate mutase [EC:5.4.2.12] | "
				KEGG | ko00010 Glycolysis / Gluconeogenesis | K16370 6-phosphofructokinase 2 [EC:2.7.1.11] | "
				KEGG | ko00020 Citrate cycle (TCA cycle) | K00031 isocitrate dehydrogenase [EC:1.1.1.42] | "
				KEGG | ko00020 Citrate cycle (TCA cycle) | K00239 succinate dehydrogenase / fumarate reductase, flavoprotein subunit [EC:1.3.5.1 1.3.5.4] | "
				KEGG | ko00020 Citrate cycle (TCA cycle) | K01647 citrate synthase [EC:2.3.3.1] | "
				KEGG | ko00020 Citrate cycle (TCA cycle) | K01677 fumarate hydratase subunit alpha [EC:4.2.1.2] | "
				KEGG | ko00020 Citrate cycle (TCA cycle) | K01678 fumarate hydratase subunit beta [EC:4.2.1.2] | "
				KEGG | ko00020 Citrate cycle (TCA cycle) | K01681 aconitate hydratase [EC:4.2.1.3] | "
				KEGG | ko00020 Citrate cycle (TCA cycle) | K01958 pyruvate carboxylase [EC:6.4.1.1] | "
				KEGG | ko00030 Pentose phosphate pathway | K00033 6-phosphogluconate dehydrogenase [EC:1.1.1.44 1.1.1.343] | "
				KEGG | ko00030 Pentose phosphate pathway | K00036 glucose-6-phosphate 1-dehydrogenase [EC:1.1.1.49 1.1.1.363] | "
				KEGG | ko00030 Pentose phosphate pathway | K00615 transketolase [EC:2.2.1.1] | "
				KEGG | ko00030 Pentose phosphate pathway | K00616 transaldolase [EC:2.2.1.2] | "
				KEGG | ko00030 Pentose phosphate pathway | K00852 ribokinase [EC:2.7.1.15] | "
				KEGG | ko00030 Pentose phosphate pathway | K00874 2-dehydro-3-deoxygluconokinase [EC:2.7.1.45] | "
				KEGG | ko00030 Pentose phosphate pathway | K00948 ribose-phosphate pyrophosphokinase [EC:2.7.6.1] | "
				KEGG | ko00030 Pentose phosphate pathway | K01619 deoxyribose-phosphate aldolase [EC:4.1.2.4] | "
				KEGG | ko00030 Pentose phosphate pathway | K01621 xylulose-5-phosphate/fructose-6-phosphate phosphoketolase [EC:4.1.2.9 4.1.2.22] | "
				KEGG | ko00030 Pentose phosphate pathway | K01625 2-dehydro-3-deoxyphosphogluconate aldolase / (4S)-4-hydroxy-2-oxoglutarate aldolase [EC:4.1.2.14 4.1.3.42] | "
				KEGG | ko00030 Pentose phosphate pathway | K01783 ribulose-phosphate 3-epimerase [EC:5.1.3.1] | "
				KEGG | ko00030 Pentose phosphate pathway | K01808 ribose 5-phosphate isomerase B [EC:5.3.1.6] | "
				KEGG | ko00030 Pentose phosphate pathway | K01839 phosphopentomutase [EC:5.4.2.7] | "
				KEGG | ko00030 Pentose phosphate pathway | K08093 3-hexulose-6-phosphate synthase [EC:4.1.2.43] | "
				KEGG | ko00030 Pentose phosphate pathway | K08094 6-phospho-3-hexuloisomerase [EC:5.3.1.27] | "
				KEGG | ko00030 Pentose phosphate pathway | K17467 PTS system, D-glucosaminate-specific IID component | "
				KEGG | ko00500 Starch and sucrose metabolism | K00688 glycogen phosphorylase [EC:2.4.1.1] | "
				KEGG | ko00500 Starch and sucrose metabolism | K00690 sucrose phosphorylase [EC:2.4.1.7] | "
				KEGG | ko00500 Starch and sucrose metabolism | K00692 levansucrase [EC:2.4.1.10] | "
				KEGG | ko00500 Starch and sucrose metabolism | K00697 trehalose 6-phosphate synthase [EC:2.4.1.15] | "
				KEGG | ko00500 Starch and sucrose metabolism | K00700 1,4-alpha-glucan branching enzyme [EC:2.4.1.18] | "
				KEGG | ko00500 Starch and sucrose metabolism | K00702 cellobiose phosphorylase [EC:2.4.1.20] | "
				KEGG | ko00500 Starch and sucrose metabolism | K00703 starch synthase [EC:2.4.1.21] | "
				KEGG | ko00500 Starch and sucrose metabolism | K00705 4-alpha-glucanotransferase [EC:2.4.1.25] | "
				KEGG | ko00500 Starch and sucrose metabolism | K00975 glucose-1-phosphate adenylyltransferase [EC:2.7.7.27] | "
				KEGG | ko00500 Starch and sucrose metabolism | K00978 glucose-1-phosphate cytidylyltransferase [EC:2.7.7.33] | "
				KEGG | ko00500 Starch and sucrose metabolism | K01176 alpha-amylase [EC:3.2.1.1] | "
				KEGG | ko00500 Starch and sucrose metabolism | K01226 trehalose-6-phosphate hydrolase [EC:3.2.1.93] | "
				KEGG | ko00500 Starch and sucrose metabolism | K01232 maltose-6'-phosphate glucosidase [EC:3.2.1.122] | "
				KEGG | ko00500 Starch and sucrose metabolism | K01838 beta-phosphoglucomutase [EC:5.4.2.6] | "
				KEGG | ko00500 Starch and sucrose metabolism | K02438 glycogen debranching enzyme [EC:3.2.1.196] | "
				KEGG | ko00500 Starch and sucrose metabolism | K02750 PTS system, alpha-glucoside-specific IIC component | "
				KEGG | ko00500 Starch and sucrose metabolism | K02761 PTS system, cellobiose-specific IIC component | "
				KEGG | ko00500 Starch and sucrose metabolism | K02810 PTS system, sucrose-specific IIC component | "
				KEGG | ko00500 Starch and sucrose metabolism | K02819 PTS system, trehalose-specific IIC component | "
				KEGG | ko00500 Starch and sucrose metabolism | K05341 amylosucrase [EC:2.4.1.4] | "
				KEGG | ko00500 Starch and sucrose metabolism | K05342 alpha,alpha-trehalose phosphorylase [EC:2.4.1.64] | "
				KEGG | ko00500 Starch and sucrose metabolism | K05343 maltose alpha-D-glucosyltransferase / alpha-amylase [EC:5.4.99.16 3.2.1.1] | "
				KEGG | ko00500 Starch and sucrose metabolism | K05349 beta-glucosidase [EC:3.2.1.21] | "
				KEGG | ko00500 Starch and sucrose metabolism | K05350 beta-glucosidase [EC:3.2.1.21] | "
				KEGG | ko00500 Starch and sucrose metabolism | K16147 starch synthase (maltosyl-transferring) [EC:2.4.99.16] | "
				KEGG | ko00620 Pyruvate metabolism | K00027 malate dehydrogenase (oxaloacetate-decarboxylating) [EC:1.1.1.38] | "
				KEGG | ko00620 Pyruvate metabolism | K00156 pyruvate dehydrogenase (quinone) [EC:1.2.5.1] | "
				KEGG | ko00620 Pyruvate metabolism | K00625 phosphate acetyltransferase [EC:2.3.1.8] | "
				KEGG | ko00620 Pyruvate metabolism | K00626 acetyl-CoA C-acetyltransferase [EC:2.3.1.9] | "
				KEGG | ko00620 Pyruvate metabolism | K00656 formate C-acetyltransferase [EC:2.3.1.54] | "
				KEGG | ko00620 Pyruvate metabolism | K00925 acetate kinase [EC:2.7.2.1] | "
				KEGG | ko00620 Pyruvate metabolism | K01006 pyruvate, orthophosphate dikinase [EC:2.7.9.1] | "
				KEGG | ko00620 Pyruvate metabolism | K01007 pyruvate, water dikinase [EC:2.7.9.2] | "
				KEGG | ko00620 Pyruvate metabolism | K01069 hydroxyacylglutathione hydrolase [EC:3.1.2.6] | "
				KEGG | ko00620 Pyruvate metabolism | K01571 oxaloacetate decarboxylase, alpha subunit [EC:4.1.1.3] | "
				KEGG | ko00620 Pyruvate metabolism | K01572 oxaloacetate decarboxylase, beta subunit [EC:4.1.1.3] | "
				KEGG | ko00620 Pyruvate metabolism | K01595 phosphoenolpyruvate carboxylase [EC:4.1.1.31] | "
				KEGG | ko00620 Pyruvate metabolism | K01649 2-isopropylmalate synthase [EC:2.3.3.13] | "
				KEGG | ko00620 Pyruvate metabolism | K01759 lactoylglutathione lyase [EC:4.4.1.5] | "
				KEGG | ko00620 Pyruvate metabolism | K01961 acetyl-CoA carboxylase, biotin carboxylase subunit [EC:6.4.1.2 6.3.4.14] | "

<table>
<tr><th></th><th></th><th></th><th></th><th></th></tr>
<tr><td></td><td></td><td></td><td></td><td>KEGG | ko00620 Pyruvate metabolism | K01962 acetyl-CoA carboxylase carboxyl transferase subunit alpha [EC:6.4.1.2] | "</td></tr>
<tr><td></td><td></td><td></td><td></td><td>KEGG | ko00620 Pyruvate metabolism | K01963 acetyl-CoA carboxylase carboxyl transferase subunit beta [EC:6.4.1.2] | "</td></tr>
<tr><td></td><td></td><td></td><td></td><td>KEGG | ko00620 Pyruvate metabolism | K02160 acetyl-CoA carboxylase biotin carboxyl carrier protein | "</td></tr>
<tr><td></td><td></td><td></td><td></td><td>KEGG | ko00620 Pyruvate metabolism | K03778 D-lactate dehydrogenase [EC:1.1.1.28] | "</td></tr>
<tr><td></td><td></td><td></td><td>Propionate Metabolism</td><td>KEGG | ko00640 Propanoate metabolism | K00005 glycerol dehydrogenase [EC:1.1.1.6] | "</td></tr>
<tr><td></td><td></td><td></td><td></td><td>KEGG | ko00640 Propanoate metabolism | K01034 acetate CoA/acetoacetate CoA-transferase alpha subunit [EC:2.8.3.8 2.8.3.9] | "</td></tr>
<tr><td></td><td></td><td></td><td></td><td>KEGG | ko00640 Propanoate metabolism | K01035 acetate CoA/acetoacetate CoA-transferase beta subunit [EC:2.8.3.8 2.8.3.9] | "</td></tr>
<tr><td></td><td></td><td></td><td></td><td>KEGG | ko00640 Propanoate metabolism | K01734 methylglyoxal synthase [EC:4.2.3.3] | "</td></tr>
<tr><td></td><td></td><td></td><td></td><td>KEGG | ko00640 Propanoate metabolism | K01825 3-hydroxyacyl-CoA dehydrogenase / enoyl-CoA hydratase / 3-hydroxybutyryl-CoA epimerase / enoyl-CoA isomerase [EC:1.1.1.35 4.2.1.17 5.1.2.3 5.3.3.8] | "</td></tr>
<tr><td></td><td></td><td></td><td></td><td>KEGG | ko00640 Propanoate metabolism | K08325 NADP-dependent alcohol dehydrogenase [EC:1.1.-.-] | "</td></tr>
<tr><td></td><td></td><td></td><td></td><td>KEGG | ko00640 Propanoate metabolism | K13919 propanediol dehydratase medium subunit [EC:4.2.1.28] | "</td></tr>
<tr><td></td><td></td><td></td><td></td><td>KEGG | ko00640 Propanoate metabolism | K13922 propionaldehyde dehydrogenase [EC:1.2.1.87] | "</td></tr>
<tr><td></td><td></td><td></td><td></td><td>KEGG | ko00640 Propanoate metabolism | K18369 alcohol dehydrogenase [EC:1.1.1.-] | "</td></tr>
<tr><td></td><td></td><td></td><td>Butyrate Metabolism</td><td>KEGG | ko00650 Butanoate metabolism | K00004 (R,R)-butanediol dehydrogenase / meso-butanediol dehydrogenase / diacetyl reductase [EC:1.1.1.4 1.1.1.- 1.1.1.303] | "</td></tr>
<tr><td></td><td></td><td></td><td></td><td>KEGG | ko00650 Butanoate metabolism | K00074 3-hydroxybutyryl-CoA dehydrogenase [EC:1.1.1.157] | "</td></tr>
<tr><td></td><td></td><td></td><td></td><td>KEGG | ko00650 Butanoate metabolism | K00100 butanol dehydrogenase [EC:1.1.1.-] | "</td></tr>
<tr><td></td><td></td><td></td><td></td><td>KEGG | ko00650 Butanoate metabolism | K00209 enoyl-[acyl-carrier protein] reductase / trans-2-enoyl-CoA reductase (NAD+) [EC:1.3.1.9 1.3.1.44] | "</td></tr>
<tr><td></td><td></td><td></td><td></td><td>KEGG | ko00650 Butanoate metabolism | K00248 butyryl-CoA dehydrogenase [EC:1.3.8.1] | "</td></tr>
<tr><td></td><td></td><td></td><td></td><td>KEGG | ko00650 Butanoate metabolism | K01652 acetolactate synthase I/II/III large subunit [EC:2.2.1.6] | "</td></tr>
<tr><td></td><td></td><td></td><td></td><td>KEGG | ko00650 Butanoate metabolism | K01653 acetolactate synthase I/III small subunit [EC:2.2.1.6] | "</td></tr>
<tr><td></td><td></td><td></td><td></td><td>KEGG | ko00650 Butanoate metabolism | K14534 4-hydroxybutyryl-CoA dehydratase / vinylacetyl-CoA-Delta-isomerase [EC:4.2.1.120 5.3.3.3] | "</td></tr>
<tr><td></td><td></td><td></td><td></td><td>KEGG | ko00650 Butanoate metabolism | K18119 succinate-semialdehyde dehydrogenase [EC:1.2.1.76] | "</td></tr>
<tr><td></td><td></td><td></td><td></td><td>KEGG | ko00650 Butanoate metabolism | K18122 4-hydroxybutyrate CoA-transferase [EC:2.8.3.-] | "</td></tr>
<tr><td>Firmicutes</td><td>Ruminococcaceae</td><td>Ruminococcus gnavus</td><td>Glycolysis-associated Metabolism</td><td>KEGG | ko00010 Glycolysis / Gluconeogenesis | K00016 L-lactate dehydrogenase [EC:1.1.1.27] | "</td></tr>
<tr><td></td><td></td><td></td><td></td><td>KEGG | ko00010 Glycolysis / Gluconeogenesis | K00121 S-(hydroxymethyl)glutathione dehydrogenase / alcohol dehydrogenase [EC:1.1.1.284 1.1.1.1] | "</td></tr>
<tr><td></td><td></td><td></td><td></td><td>KEGG | ko00010 Glycolysis / Gluconeogenesis | K00128 aldehyde dehydrogenase (NAD+) [EC:1.2.1.3] | "</td></tr>
<tr><td></td><td></td><td></td><td></td><td>KEGG | ko00010 Glycolysis / Gluconeogenesis | K00134 glyceraldehyde 3-phosphate dehydrogenase [EC:1.2.1.12] | "</td></tr>
<tr><td></td><td></td><td></td><td></td><td>KEGG | ko00010 Glycolysis / Gluconeogenesis | K00162 pyruvate dehydrogenase E1 component beta subunit [EC:1.2.4.1] | "</td></tr>
<tr><td></td><td></td><td></td><td></td><td>KEGG | ko00010 Glycolysis / Gluconeogenesis | K00382 dihydrolipoamide dehydrogenase [EC:1.8.1.4] | "</td></tr>
<tr><td></td><td></td><td></td><td></td><td>KEGG | ko00010 Glycolysis / Gluconeogenesis | K00845 glucokinase [EC:2.7.1.2] | "</td></tr>
<tr><td></td><td></td><td></td><td></td><td>KEGG | ko00010 Glycolysis / Gluconeogenesis | K00850 6-phosphofructokinase 1 [EC:2.7.1.11] | "</td></tr>
<tr><td></td><td></td><td></td><td></td><td>KEGG | ko00010 Glycolysis / Gluconeogenesis | K00873 pyruvate kinase [EC:2.7.1.40] | "</td></tr>
<tr><td></td><td></td><td></td><td></td><td>KEGG | ko00010 Glycolysis / Gluconeogenesis | K00927 phosphoglycerate kinase [EC:2.7.2.3] | "</td></tr>
<tr><td></td><td></td><td></td><td></td><td>KEGG | ko00010 Glycolysis / Gluconeogenesis | K01223 6-phospho-beta-glucosidase [EC:3.2.1.86] | "</td></tr>
<tr><td></td><td></td><td></td><td></td><td>KEGG | ko00010 Glycolysis / Gluconeogenesis | K01610 phosphoenolpyruvate carboxykinase (ATP) [EC:4.1.1.49] | "</td></tr>
<tr><td></td><td></td><td></td><td></td><td>KEGG | ko00010 Glycolysis / Gluconeogenesis | K01624 fructose-bisphosphate aldolase, class II [EC:4.1.2.13] | "</td></tr>
<tr><td></td><td></td><td></td><td></td><td>KEGG | ko00010 Glycolysis / Gluconeogenesis | K01689 enolase [EC:4.2.1.11] | "</td></tr>
<tr><td></td><td></td><td></td><td></td><td>KEGG | ko00010 Glycolysis / Gluconeogenesis | K01785 aldose 1-epimerase [EC:5.1.3.3] | "</td></tr>
<tr><td></td><td></td><td></td><td></td><td>KEGG | ko00010 Glycolysis / Gluconeogenesis | K01803 triosephosphate isomerase (TIM) [EC:5.3.1.1] | "</td></tr>
<tr><td></td><td></td><td></td><td></td><td>KEGG | ko00010 Glycolysis / Gluconeogenesis | K01810 glucose-6-phosphate isomerase [EC:5.3.1.9] | "</td></tr>
<tr><td></td><td></td><td></td><td></td><td>KEGG | ko00010 Glycolysis / Gluconeogenesis | K01834 2,3-bisphosphoglycerate-dependent phosphoglycerate mutase [EC:5.4.2.11] | "</td></tr>
<tr><td></td><td></td><td></td><td></td><td>KEGG | ko00010 Glycolysis / Gluconeogenesis | K01835 phosphoglucomutase [EC:5.4.2.2] | "</td></tr>
<tr><td></td><td></td><td></td><td></td><td>KEGG | ko00010 Glycolysis / Gluconeogenesis | K02777 PTS system, sugar-specific IIA component [EC:2.7.1.-] | "</td></tr>
<tr><td></td><td></td><td></td><td></td><td>KEGG | ko00010 Glycolysis / Gluconeogenesis | K02779 PTS system, glucose-specific IIC component | "</td></tr>
<tr><td></td><td></td><td></td><td></td><td>KEGG | ko00010 Glycolysis / Gluconeogenesis | K02791 PTS system, maltose/glucose-specific IIC component | "</td></tr>
<tr><td></td><td></td><td></td><td></td><td>KEGG | ko00010 Glycolysis / Gluconeogenesis | K03737 pyruvate-ferredoxin/flavodoxin oxidoreductase [EC:1.2.7.1 1.2.7.-] | "</td></tr>
<tr><td></td><td></td><td></td><td></td><td>KEGG | ko00010 Glycolysis / Gluconeogenesis | K04022 alcohol dehydrogenase | "</td></tr>
<tr><td></td><td></td><td></td><td></td><td>KEGG | ko00010 Glycolysis / Gluconeogenesis | K04041 fructose-1,6-bisphosphatase III [EC:3.1.3.11] | "</td></tr>
<tr><td></td><td></td><td></td><td></td><td>KEGG | ko00010 Glycolysis / Gluconeogenesis | K04072 acetaldehyde dehydrogenase / alcohol dehydrogenase [EC:1.2.1.10 1.1.1.1] | "</td></tr>
<tr><td></td><td></td><td></td><td></td><td>KEGG | ko00010 Glycolysis / Gluconeogenesis | K11645 fructose-bisphosphate aldolase, class I [EC:4.1.2.13] | "</td></tr>
<tr><td></td><td></td><td></td><td></td><td>KEGG | ko00010 Glycolysis / Gluconeogenesis | K13953 alcohol dehydrogenase, propanol-preferring [EC:1.1.1.1] | "</td></tr>
<tr><td></td><td></td><td></td><td></td><td>KEGG | ko00010 Glycolysis / Gluconeogenesis | K15633 2,3-bisphosphoglycerate-independent phosphoglycerate mutase [EC:5.4.2.12] | "</td></tr>
<tr><td></td><td></td><td></td><td></td><td>KEGG | ko00010 Glycolysis / Gluconeogenesis | K15634 probable phosphoglycerate mutase [EC:5.4.2.12] | "</td></tr>
<tr><td></td><td></td><td></td><td></td><td>KEGG | ko00020 Citrate cycle (TCA cycle) | K00024 malate dehydrogenase [EC:1.1.1.37] | "</td></tr>
<tr><td></td><td></td><td></td><td></td><td>KEGG | ko00020 Citrate cycle (TCA cycle) | K00031 isocitrate dehydrogenase [EC:1.1.1.42] | "</td></tr>
<tr><td></td><td></td><td></td><td></td><td>KEGG | ko00020 Citrate cycle (TCA cycle) | K00116 malate dehydrogenase (quinone) [EC:1.1.5.4] | "</td></tr>
<tr><td></td><td></td><td></td><td></td><td>KEGG | ko00020 Citrate cycle (TCA cycle) | K00239 succinate dehydrogenase / fumarate reductase, flavoprotein subunit [EC:1.3.5.1 1.3.5.4] | "</td></tr>
<tr><td></td><td></td><td></td><td></td><td>KEGG | ko00020 Citrate cycle (TCA cycle) | K00240 succinate dehydrogenase / fumarate reductase, iron-sulfur subunit [EC:1.3.5.1 1.3.5.4] | "</td></tr>
<tr><td></td><td></td><td></td><td></td><td>KEGG | ko00020 Citrate cycle (TCA cycle) | K00658 2-oxoglutarate dehydrogenase E2 component (dihydrolipoamide succinyltransferase) [EC:2.3.1.61] | "</td></tr>
<tr><td></td><td></td><td></td><td></td><td>KEGG | ko00020 Citrate cycle (TCA cycle) | K01647 citrate synthase [EC:2.3.3.1] | "</td></tr>
<tr><td></td><td></td><td></td><td></td><td>KEGG | ko00020 Citrate cycle (TCA cycle) | K01676 fumarate hydratase, class I [EC:4.2.1.2] | "</td></tr>
<tr><td></td><td></td><td></td><td></td><td>KEGG | ko00020 Citrate cycle (TCA cycle) | K01677 fumarate hydratase subunit alpha [EC:4.2.1.2] | "</td></tr>
<tr><td></td><td></td><td></td><td></td><td>KEGG | ko00020 Citrate cycle (TCA cycle) | K01678 fumarate hydratase subunit beta [EC:4.2.1.2] | "</td></tr>
<tr><td></td><td></td><td></td><td></td><td>KEGG | ko00020 Citrate cycle (TCA cycle) | K01681 aconitate hydratase [EC:4.2.1.3] | "</td></tr>
<tr><td></td><td></td><td></td><td></td><td>KEGG | ko00020 Citrate cycle (TCA cycle) | K01903 succinyl-CoA synthetase beta subunit [EC:6.2.1.5] | "</td></tr>
<tr><td></td><td></td><td></td><td></td><td>KEGG | ko00020 Citrate cycle (TCA cycle) | K01960 pyruvate carboxylase subunit B [EC:6.4.1.1] | "</td></tr>
<tr><td></td><td></td><td></td><td></td><td>KEGG | ko00030 Pentose phosphate pathway | K00033 6-phosphogluconate dehydrogenase [EC:1.1.1.44 1.1.1.343] | "</td></tr>
<tr><td></td><td></td><td></td><td></td><td>KEGG | ko00030 Pentose phosphate pathway | K00036 glucose-6-phosphate 1-dehydrogenase [EC:1.1.1.49 1.1.1.363] | "</td></tr>
<tr><td></td><td></td><td></td><td></td><td>KEGG | ko00030 Pentose phosphate pathway | K00615 transketolase [EC:2.2.1.1] | "</td></tr>
<tr><td></td><td></td><td></td><td></td><td>KEGG | ko00030 Pentose phosphate pathway | K00616 transaldolase [EC:2.2.1.2] | "</td></tr>
<tr><td></td><td></td><td></td><td></td><td>KEGG | ko00030 Pentose phosphate pathway | K00851 gluconokinase [EC:2.7.1.12] | "</td></tr>
<tr><td></td><td></td><td></td><td></td><td>KEGG | ko00030 Pentose phosphate pathway | K00852 ribokinase [EC:2.7.1.15] | "</td></tr>
<tr><td></td><td></td><td></td><td></td><td>KEGG | ko00030 Pentose phosphate pathway | K00874 2-dehydro-3-deoxygluconokinase [EC:2.7.1.45] | "</td></tr>
<tr><td></td><td></td><td></td><td></td><td>KEGG | ko00030 Pentose phosphate pathway | K00948 ribose-phosphate pyrophosphokinase [EC:2.7.6.1] | "</td></tr>
<tr><td></td><td></td><td></td><td></td><td>KEGG | ko00030 Pentose phosphate pathway | K01621 xylulose-5-phosphate/fructose-6-phosphate phosphoketolase [EC:4.1.2.9 4.1.2.22] | "</td></tr>
<tr><td></td><td></td><td></td><td></td><td>KEGG | ko00030 Pentose phosphate pathway | K01625 2-dehydro-3-deoxyphosphogluconate aldolase / (4S)-4-hydroxy-2-oxoglutarate aldolase [EC:4.1.2.14 4.1.3.42] | "</td></tr>
<tr><td></td><td></td><td></td><td></td><td>KEGG | ko00030 Pentose phosphate pathway | K01690 phosphogluconate dehydratase [EC:4.2.1.12] | "</td></tr>
<tr><td></td><td></td><td></td><td></td><td>KEGG | ko00030 Pentose phosphate pathway | K01783 ribulose-phosphate 3-epimerase [EC:5.1.3.1] | "</td></tr>
<tr><td></td><td></td><td></td><td></td><td>KEGG | ko00030 Pentose phosphate pathway | K01808 ribose 5-phosphate isomerase B [EC:5.3.1.6] | "</td></tr>
<tr><td></td><td></td><td></td><td></td><td>KEGG | ko00030 Pentose phosphate pathway | K01839 phosphopentomutase [EC:5.4.2.7] | "</td></tr>
<tr><td></td><td></td><td></td><td></td><td>KEGG | ko00030 Pentose phosphate pathway | K08094 6-phospho-3-hexuloisomerase [EC:5.3.1.27] | "</td></tr>
<tr><td></td><td></td><td></td><td></td><td>KEGG | ko00500 Starch and sucrose metabolism | K00688 glycogen phosphorylase [EC:2.4.1.1] | "</td></tr>
<tr><td></td><td></td><td></td><td></td><td>KEGG | ko00500 Starch and sucrose metabolism | K00690 sucrose phosphorylase [EC:2.4.1.7] | "</td></tr>
<tr><td></td><td></td><td></td><td></td><td>KEGG | ko00500 Starch and sucrose metabolism | K00694 cellulose synthase (UDP-forming) [EC:2.4.1.12] | "</td></tr>
<tr><td></td><td></td><td></td><td></td><td>KEGG | ko00500 Starch and sucrose metabolism | K00700 1,4-alpha-glucan branching enzyme [EC:2.4.1.18] | "</td></tr>
<tr><td></td><td></td><td></td><td></td><td>KEGG | ko00500 Starch and sucrose metabolism | K00702 cellobiose phosphorylase [EC:2.4.1.20] | "</td></tr>
<tr><td></td><td></td><td></td><td></td><td>KEGG | ko00500 Starch and sucrose metabolism | K00703 starch synthase [EC:2.4.1.21] | "</td></tr>
<tr><td></td><td></td><td></td><td></td><td>KEGG | ko00500 Starch and sucrose metabolism | K00705 4-alpha-glucanotransferase [EC:2.4.1.25] | "</td></tr>
<tr><td></td><td></td><td></td><td></td><td>KEGG | ko00500 Starch and sucrose metabolism | K00975 glucose-1-phosphate adenylyltransferase [EC:2.7.7.27] | "</td></tr>
<tr><td></td><td></td><td></td><td></td><td>KEGG | ko00500 Starch and sucrose metabolism | K01179 endoglucanase [EC:3.2.1.4] | "</td></tr>
<tr><td></td><td></td><td></td><td></td><td>KEGG | ko00500 Starch and sucrose metabolism | K01232 maltose-6'-phosphate glucosidase [EC:3.2.1.122] | "</td></tr>
<tr><td></td><td></td><td></td><td></td><td>KEGG | ko00500 Starch and sucrose metabolism | K02438 glycogen debranching enzyme [EC:3.2.1.196] | "</td></tr>
<tr><td></td><td></td><td></td><td></td><td>KEGG | ko00500 Starch and sucrose metabolism | K02759 PTS system, cellobiose-specific IIA component [EC:2.7.1.196 2.7.1.205] | "</td></tr>
<tr><td></td><td></td><td></td><td></td><td>KEGG | ko00500 Starch and sucrose metabolism | K02760 PTS system, cellobiose-specific IIB component [EC:2.7.1.196 2.7.1.205] | "</td></tr>
<tr><td></td><td></td><td></td><td></td><td>KEGG | ko00500 Starch and sucrose metabolism | K02761 PTS system, cellobiose-specific IIC component | "</td></tr>
<tr><td></td><td></td><td></td><td></td><td>KEGG | ko00500 Starch and sucrose metabolism | K02810 PTS system, sucrose-specific IIC component | "</td></tr>
<tr><td></td><td></td><td></td><td></td><td>KEGG | ko00500 Starch and sucrose metabolism | K05341 amylosucrase [EC:2.4.1.4] | "</td></tr>
<tr><td></td><td></td><td></td><td></td><td>KEGG | ko00500 Starch and sucrose metabolism | K05349 beta-glucosidase [EC:3.2.1.21] | "</td></tr>
<tr><td></td><td></td><td></td><td></td><td>KEGG | ko00500 Starch and sucrose metabolism | K05350 beta-glucosidase [EC:3.2.1.21] | "</td></tr>
<tr><td></td><td></td><td></td><td></td><td>KEGG | ko00500 Starch and sucrose metabolism | K16147 starch synthase (maltosyl-transferring) [EC:2.4.99.16] | "</td></tr>
<tr><td></td><td></td><td></td><td></td><td>KEGG | ko00500 Starch and sucrose metabolism | K16148 alpha-maltose-1-phosphate synthase [EC:2.4.1.342] | "</td></tr>
<tr><td></td><td></td><td></td><td></td><td>KEGG | ko00620 Pyruvate metabolism | K00027 malate dehydrogenase (oxaloacetate-decarboxylating) [EC:1.1.1.38] | "</td></tr>
<tr><td></td><td></td><td></td><td></td><td>KEGG | ko00620 Pyruvate metabolism | K00625 phosphate acetyltransferase [EC:2.3.1.8] | "</td></tr>
<tr><td></td><td></td><td></td><td></td><td>KEGG | ko00620 Pyruvate metabolism | K00626 acetyl-CoA C-acetyltransferase [EC:2.3.1.9] | "</td></tr>
<tr><td></td><td></td><td></td><td></td><td>KEGG | ko00620 Pyruvate metabolism | K00656 formate C-acetyltransferase [EC:2.3.1.54] | "</td></tr>
<tr><td></td><td></td><td></td><td></td><td>KEGG | ko00620 Pyruvate metabolism | K00925 acetate kinase [EC:2.7.2.1] | "</td></tr>
<tr><td></td><td></td><td></td><td></td><td>KEGG | ko00620 Pyruvate metabolism | K01006 pyruvate, orthophosphate dikinase [EC:2.7.9.1] | "</td></tr>
<tr><td></td><td></td><td></td><td></td><td>KEGG | ko00620 Pyruvate metabolism | K01007 pyruvate, water dikinase [EC:2.7.9.2] | "</td></tr>
<tr><td></td><td></td><td></td><td></td><td>KEGG | ko00620 Pyruvate metabolism | K01512 acylphosphatase [EC:3.6.1.7] | "</td></tr>
<tr><td></td><td></td><td></td><td></td><td>KEGG | ko00620 Pyruvate metabolism | K01571 oxaloacetate decarboxylase, alpha subunit [EC:4.1.1.3] | "</td></tr>
<tr><td></td><td></td><td></td><td></td><td>KEGG | ko00620 Pyruvate metabolism | K01572 oxaloacetate decarboxylase, beta subunit [EC:4.1.1.3] | "</td></tr>
<tr><td></td><td></td><td></td><td></td><td>KEGG | ko00620 Pyruvate metabolism | K01595 phosphoenolpyruvate carboxylase [EC:4.1.1.31] | "</td></tr>
<tr><td></td><td></td><td></td><td></td><td>KEGG | ko00620 Pyruvate metabolism | K01638 malate synthase [EC:2.3.3.9] | "</td></tr>
<tr><td></td><td></td><td></td><td></td><td>KEGG | ko00620 Pyruvate metabolism | K01649 2-isopropylmalate synthase [EC:2.3.3.13] | "</td></tr>
<tr><td></td><td></td><td></td><td></td><td>KEGG | ko00620 Pyruvate metabolism | K01759 lactoylglutathione lyase [EC:4.4.1.5] | "</td></tr>
<tr><td></td><td></td><td></td><td></td><td>KEGG | ko00620 Pyruvate metabolism | K01961 acetyl-CoA carboxylase, biotin carboxylase subunit [EC:6.4.1.2 6.3.4.14] | "</td></tr>
<tr><td></td><td></td><td></td><td></td><td>KEGG | ko00620 Pyruvate metabolism | K01962 acetyl-CoA carboxylase carboxyl transferase subunit alpha [EC:6.4.1.2] | "</td></tr>
<tr><td></td><td></td><td></td><td></td><td>KEGG | ko00620 Pyruvate metabolism | K01963 acetyl-CoA carboxylase carboxyl transferase subunit beta [EC:6.4.1.2] | "</td></tr>
<tr><td></td><td></td><td></td><td></td><td>KEGG | ko00620 Pyruvate metabolism | K02160 acetyl-CoA carboxylase biotin carboxyl carrier protein | "</td></tr>
<tr><td></td><td></td><td></td><td></td><td>KEGG | ko00620 Pyruvate metabolism | K03778 D-lactate dehydrogenase [EC:1.1.1.28] | "</td></tr>
<tr><td></td><td></td><td></td><td></td><td>KEGG | ko00620 Pyruvate metabolism | K11263 acetyl-CoA/propionyl-CoA carboxylase, biotin carboxylase, biotin carboxyl carrier protein [EC:6.4.1.2 6.4.1.3 6.3.4.14] | "</td></tr>
<tr><td></td><td></td><td></td><td>Propionate Metabolism</td><td>KEGG | ko00640 Propanoate metabolism | K00005 glycerol dehydrogenase [EC:1.1.1.6] | "</td></tr>
<tr><td></td><td></td><td></td><td></td><td>KEGG | ko00640 Propanoate metabolism | K00823 4-aminobutyrate aminotransferase [EC:2.6.1.19] | "</td></tr>
<tr><td></td><td></td><td></td><td></td><td>KEGG | ko00640 Propanoate metabolism | K01699 propanediol dehydratase large subunit [EC:4.2.1.28] | "</td></tr>
<tr><td></td><td></td><td></td><td></td><td>KEGG | ko00640 Propanoate metabolism | K01720 2-methylcitrate dehydratase [EC:4.2.1.79] | "</td></tr>
<tr><td></td><td></td><td></td><td></td><td>KEGG | ko00640 Propanoate metabolism | K01734 methylglyoxal synthase [EC:4.2.3.3] | "</td></tr>
<tr><td></td><td></td><td></td><td></td><td>KEGG | ko00640 Propanoate metabolism | K01825 3-hydroxyacyl-CoA dehydrogenase / enoyl-CoA hydratase / 3-hydroxybutyryl-CoA epimerase / enoyl-CoA isomerase [EC:1.1.1.35 4.2.1.17 5.1.2.3 5.3.3.8] | "</td></tr>
<tr><td></td><td></td><td></td><td></td><td>KEGG | ko00640 Propanoate metabolism | K01908 propionyl-CoA synthetase [EC:6.2.1.17] | "</td></tr>
<tr><td></td><td></td><td></td><td></td><td>KEGG | ko00640 Propanoate metabolism | K08325 NADP-dependent alcohol dehydrogenase [EC:1.1.-.-] | "</td></tr>
<tr><td></td><td></td><td></td><td></td><td>KEGG | ko00640 Propanoate metabolism | K13919 propanediol dehydratase medium subunit [EC:4.2.1.28] | "</td></tr>
<tr><td></td><td></td><td></td><td></td><td>KEGG | ko00640 Propanoate metabolism | K13922 propionaldehyde dehydrogenase [EC:1.2.1.87] | "</td></tr>
<tr><td></td><td></td><td></td><td></td><td>KEGG | ko00640 Propanoate metabolism | K18471 methylglyoxal reductase [EC:1.1.1.-] | "</td></tr>
<tr><td></td><td></td><td></td><td>Butyrate Metabolism</td><td>KEGG | ko00650 Butanoate metabolism | K00004 (R,R)-butanediol dehydrogenase / meso-butanediol dehydrogenase / diacetyl reductase [EC:1.1.1.4 1.1.1.- 1.1.1.303] | "</td></tr>
<tr><td></td><td></td><td></td><td></td><td>KEGG | ko00650 Butanoate metabolism | K00074 3-hydroxybutyryl-CoA dehydrogenase [EC:1.1.1.157] | "</td></tr>
<tr><td></td><td></td><td></td><td></td><td>KEGG | ko00650 Butanoate metabolism | K00100 butanol dehydrogenase [EC:1.1.1.-] | "</td></tr>
<tr><td></td><td></td><td></td><td></td><td>KEGG | ko00650 Butanoate metabolism | K00135 succinate-semialdehyde dehydrogenase / glutarate-semialdehyde dehydrogenase [EC:1.2.1.16 1.2.1.79 1.2.1.20] | "</td></tr>
<tr><td></td><td></td><td></td><td></td><td>KEGG | ko00650 Butanoate metabolism | K01652 acetolactate synthase I/II/III large subunit [EC:2.2.1.6] | "</td></tr>
<tr><td></td><td></td><td></td><td></td><td>KEGG | ko00650 Butanoate metabolism | K01653 acetolactate synthase I/III small subunit [EC:2.2.1.6] | "</td></tr>
<tr><td></td><td></td><td></td><td></td><td>KEGG | ko00650 Butanoate metabolism | K03366 meso-butanediol dehydrogenase / (S,S)-butanediol dehydrogenase / diacetyl reductase [EC:1.1.1.- 1.1.1.76 1.1.1.304] | "</td></tr>
<tr><td>Firmicutes</td><td>Ruminococcaceae</td><td>Ruminococcus sp. JC304</td><td>Glycolysis-associated Metabolism</td><td>KEGG | ko00010 Glycolysis / Gluconeogenesis | K00128 aldehyde dehydrogenase (NAD+) [EC:1.2.1.3] | "</td></tr>
<tr><td></td><td></td><td></td><td></td><td>KEGG | ko00010 Glycolysis / Gluconeogenesis | K00134 glyceraldehyde 3-phosphate dehydrogenase [EC:1.2.1.12] | "</td></tr>
<tr><td></td><td></td><td></td><td></td><td>KEGG | ko00010 Glycolysis / Gluconeogenesis | K00382 dihydrolipoamide dehydrogenase [EC:1.8.1.4] | "</td></tr>
<tr><td></td><td></td><td></td><td></td><td>KEGG | ko00010 Glycolysis / Gluconeogenesis | K00845 glucokinase [EC:2.7.1.2] | "</td></tr>
<tr><td></td><td></td><td></td><td></td><td>KEGG | ko00010 Glycolysis / Gluconeogenesis | K00850 6-phosphofructokinase 1 [EC:2.7.1.11] | "</td></tr>
<tr><td></td><td></td><td></td><td></td><td>KEGG | ko00010 Glycolysis / Gluconeogenesis | K00895 diphosphate-dependent phosphofructokinase [EC:2.7.1.90] | "</td></tr>
<tr><td></td><td></td><td></td><td></td><td>KEGG | ko00010 Glycolysis / Gluconeogenesis | K00927 phosphoglycerate kinase [EC:2.7.2.3] | "</td></tr>
<tr><td></td><td></td><td></td><td></td><td>KEGG | ko00010 Glycolysis / Gluconeogenesis | K01223 6-phospho-beta-glucosidase [EC:3.2.1.86] | "</td></tr>
<tr><td></td><td></td><td></td><td></td><td>KEGG | ko00010 Glycolysis / Gluconeogenesis | K01610 phosphoenolpyruvate carboxykinase (ATP) [EC:4.1.1.49] | "</td></tr>
<tr><td></td><td></td><td></td><td></td><td>KEGG | ko00010 Glycolysis / Gluconeogenesis | K01624 fructose-bisphosphate aldolase, class II [EC:4.1.2.13] | "</td></tr>
<tr><td></td><td></td><td></td><td></td><td>KEGG | ko00010 Glycolysis / Gluconeogenesis | K01689 enolase [EC:4.2.1.11] | "</td></tr>
<tr><td></td><td></td><td></td><td></td><td>KEGG | ko00010 Glycolysis / Gluconeogenesis | K01785 aldose 1-epimerase [EC:5.1.3.3] | "</td></tr>
<tr><td></td><td></td><td></td><td></td><td>KEGG | ko00010 Glycolysis / Gluconeogenesis | K01810 glucose-6-phosphate isomerase [EC:5.3.1.9] | "</td></tr>
<tr><td></td><td></td><td></td><td></td><td>KEGG | ko00010 Glycolysis / Gluconeogenesis | K01834 2,3-bisphosphoglycerate-dependent phosphoglycerate mutase [EC:5.4.2.11] | "</td></tr>
<tr><td></td><td></td><td></td><td></td><td>KEGG | ko00010 Glycolysis / Gluconeogenesis | K01835 phosphoglucomutase [EC:5.4.2.2] | "</td></tr>
<tr><td></td><td></td><td></td><td></td><td>KEGG | ko00010 Glycolysis / Gluconeogenesis | K01895 acetyl-CoA synthetase [EC:6.2.1.1] | "</td></tr>
<tr><td></td><td></td><td></td><td></td><td>KEGG | ko00010 Glycolysis / Gluconeogenesis | K03737 pyruvate-ferredoxin/flavodoxin oxidoreductase [EC:1.2.7.1 1.2.7.-] | "</td></tr>
<tr><td></td><td></td><td></td><td></td><td>KEGG | ko00010 Glycolysis / Gluconeogenesis | K04041 fructose-1,6-bisphosphatase III [EC:3.1.3.11] | "</td></tr>
<tr><td></td><td></td><td></td><td></td><td>KEGG | ko00010 Glycolysis / Gluconeogenesis | K13954 alcohol dehydrogenase [EC:1.1.1.1] | "</td></tr>
<tr><td></td><td></td><td></td><td></td><td>KEGG | ko00010 Glycolysis / Gluconeogenesis | K15633 2,3-bisphosphoglycerate-independent phosphoglycerate mutase [EC:5.4.2.12] | "</td></tr>
<tr><td></td><td></td><td></td><td></td><td>KEGG | ko00010 Glycolysis / Gluconeogenesis | K15634 probable phosphoglycerate mutase [EC:5.4.2.12] | "</td></tr>
<tr><td></td><td></td><td></td><td></td><td>KEGG | ko00020 Citrate cycle (TCA cycle) | K00031 isocitrate dehydrogenase [EC:1.1.1.42] | "</td></tr>
<tr><td></td><td></td><td></td><td></td><td>KEGG | ko00020 Citrate cycle (TCA cycle) | K01647 citrate synthase [EC:2.3.3.1] | "</td></tr>
<tr><td></td><td></td><td></td><td></td><td>KEGG | ko00020 Citrate cycle (TCA cycle) | K01681 aconitate hydratase [EC:4.2.1.3] | "</td></tr>
<tr><td></td><td></td><td></td><td></td><td>KEGG | ko00030 Pentose phosphate pathway | K00615 transketolase [EC:2.2.1.1] | "</td></tr>
<tr><td></td><td></td><td></td><td></td><td>KEGG | ko00030 Pentose phosphate pathway | K00616 transaldolase [EC:2.2.1.2] | "</td></tr>
<tr><td></td><td></td><td></td><td></td><td>KEGG | ko00030 Pentose phosphate pathway | K00948 ribose-phosphate pyrophosphokinase [EC:2.7.6.1] | "</td></tr>
<tr><td></td><td></td><td></td><td></td><td>KEGG | ko00030 Pentose phosphate pathway | K01621 xylulose-5-phosphate/fructose-6-phosphate phosphoketolase [EC:4.1.2.9 4.1.2.22] | "</td></tr>
<tr><td></td><td></td><td></td><td></td><td>KEGG | ko00030 Pentose phosphate pathway | K01783 ribulose-phosphate 3-epimerase [EC:5.1.3.1] | "</td></tr>
<tr><td></td><td></td><td></td><td></td><td>KEGG | ko00030 Pentose phosphate pathway | K01808 ribose 5-phosphate isomerase B [EC:5.3.1.6] | "</td></tr>
<tr><td></td><td></td><td></td><td></td><td>KEGG | ko00030 Pentose phosphate pathway | K01839 phosphopentomutase [EC:5.4.2.7] | "</td></tr>
<tr><td></td><td></td><td></td><td></td><td>KEGG | ko00030 Pentose phosphate pathway | K07404 6-phosphogluconolactonase [EC:3.1.1.31] | "</td></tr>
<tr><td></td><td></td><td></td><td></td><td>KEGG | ko00500 Starch and sucrose metabolism | K00688 glycogen phosphorylase [EC:2.4.1.1] | "</td></tr>
<tr><td></td><td></td><td></td><td></td><td>KEGG | ko00500 Starch and sucrose metabolism | K00700 1,4-alpha-glucan branching enzyme [EC:2.4.1.18] | "</td></tr>
<tr><td></td><td></td><td></td><td></td><td>KEGG | ko00500 Starch and sucrose metabolism | K00702 cellobiose phosphorylase [EC:2.4.1.20] | "</td></tr>
</table>

<table>
<tr><td rowspan="20"></td><td rowspan="20"></td><td rowspan="20"></td><td rowspan="15"></td><td>KEGG | ko00500 Starch and sucrose metabolism | K00703 starch synthase [EC:2.4.1.21] | "</td></tr>
<tr><td>KEGG | ko00500 Starch and sucrose metabolism | K00705 4-alpha-glucanotransferase [EC:2.4.1.25] | "</td></tr>
<tr><td>KEGG | ko00500 Starch and sucrose metabolism | K00975 glucose-1-phosphate adenylyltransferase [EC:2.7.7.27] | "</td></tr>
<tr><td>KEGG | ko00500 Starch and sucrose metabolism | K02438 glycogen debranching enzyme [EC:3.2.1.196] | "</td></tr>
<tr><td>KEGG | ko00500 Starch and sucrose metabolism | K05341 amylosucrase [EC:2.4.1.4] | "</td></tr>
<tr><td>KEGG | ko00500 Starch and sucrose metabolism | K05349 beta-glucosidase [EC:3.2.1.21] | "</td></tr>
<tr><td>KEGG | ko00620 Pyruvate metabolism | K00625 phosphate acetyltransferase [EC:2.3.1.8] | "</td></tr>
<tr><td>KEGG | ko00620 Pyruvate metabolism | K00656 formate C-acetyltransferase [EC:2.3.1.54] | "</td></tr>
<tr><td>KEGG | ko00620 Pyruvate metabolism | K00925 acetate kinase [EC:2.7.2.1] | "</td></tr>
<tr><td>KEGG | ko00620 Pyruvate metabolism | K01571 oxaloacetate decarboxylase, alpha subunit [EC:4.1.1.3] | "</td></tr>
<tr><td>KEGG | ko00620 Pyruvate metabolism | K01572 oxaloacetate decarboxylase, beta subunit [EC:4.1.1.3] | "</td></tr>
<tr><td>KEGG | ko00620 Pyruvate metabolism | K01759 lactoylglutathione lyase [EC:4.4.1.5] | "</td></tr>
<tr><td>KEGG | ko00620 Pyruvate metabolism | K01961 acetyl-CoA carboxylase, biotin carboxylase subunit [EC:6.4.1.2 6.3.4.14] | "</td></tr>
<tr><td>KEGG | ko00620 Pyruvate metabolism | K01963 acetyl-CoA carboxylase carboxyl transferase subunit beta [EC:6.4.1.2] | "</td></tr>
<tr><td>KEGG | ko00620 Pyruvate metabolism | K03778 D-lactate dehydrogenase [EC:1.1.1.28] | "</td></tr>
<tr><td rowspan="2">Propionate Metabolism</td><td>KEGG | ko00640 Propanoate metabolism | K01734 methylglyoxal synthase [EC:4.2.3.3] | "</td></tr>
<tr><td>KEGG | ko00640 Propanoate metabolism | K13922 propionaldehyde dehydrogenase [EC:1.2.1.87] | "</td></tr>
<tr><td rowspan="3">Butyrate Metabolism</td><td>KEGG | ko00650 Butanoate metabolism | K00209 enoyl-[acyl-carrier protein] reductase / trans-2-enoyl-CoA reductase (NAD+) [EC:1.3.1.9 1.3.1.44] | "</td></tr>
<tr><td>KEGG | ko00650 Butanoate metabolism | K01652 acetolactate synthase I/II/III large subunit [EC:2.2.1.6] | "</td></tr>
<tr><td>KEGG | ko00650 Butanoate metabolism | K01653 acetolactate synthase I/III small subunit [EC:2.2.1.6] | "</td></tr>
<tr><td rowspan="71">Firmicutes</td><td rowspan="71">Lachnospiraceae</td><td rowspan="71">Tyzzerella nexilis</td><td rowspan="69">Glycolysis-associated Metabolism</td><td>KEGG | ko00010 Glycolysis / Gluconeogenesis | K00016 L-lactate dehydrogenase [EC:1.1.1.27] | "</td></tr>
<tr><td>KEGG | ko00010 Glycolysis / Gluconeogenesis | K00128 aldehyde dehydrogenase (NAD+) [EC:1.2.1.3] | "</td></tr>
<tr><td>KEGG | ko00010 Glycolysis / Gluconeogenesis | K00131 glyceraldehyde-3-phosphate dehydrogenase (NADP+) [EC:1.2.1.9] | "</td></tr>
<tr><td>KEGG | ko00010 Glycolysis / Gluconeogenesis | K00134 glyceraldehyde 3-phosphate dehydrogenase [EC:1.2.1.12] | "</td></tr>
<tr><td>KEGG | ko00010 Glycolysis / Gluconeogenesis | K00382 dihydrolipoamide dehydrogenase [EC:1.8.1.4] | "</td></tr>
<tr><td>KEGG | ko00010 Glycolysis / Gluconeogenesis | K00845 glucokinase [EC:2.7.1.2] | "</td></tr>
<tr><td>KEGG | ko00010 Glycolysis / Gluconeogenesis | K00850 6-phosphofructokinase 1 [EC:2.7.1.11] | "</td></tr>
<tr><td>KEGG | ko00010 Glycolysis / Gluconeogenesis | K00873 pyruvate kinase [EC:2.7.1.40] | "</td></tr>
<tr><td>KEGG | ko00010 Glycolysis / Gluconeogenesis | K00927 phosphoglycerate kinase [EC:2.7.2.3] | "</td></tr>
<tr><td>KEGG | ko00010 Glycolysis / Gluconeogenesis | K01223 6-phospho-beta-glucosidase [EC:3.2.1.86] | "</td></tr>
<tr><td>KEGG | ko00010 Glycolysis / Gluconeogenesis | K01610 phosphoenolpyruvate carboxykinase (ATP) [EC:4.1.1.49] | "</td></tr>
<tr><td>KEGG | ko00010 Glycolysis / Gluconeogenesis | K01624 fructose-bisphosphate aldolase, class II [EC:4.1.2.13] | "</td></tr>
<tr><td>KEGG | ko00010 Glycolysis / Gluconeogenesis | K01689 enolase [EC:4.2.1.11] | "</td></tr>
<tr><td>KEGG | ko00010 Glycolysis / Gluconeogenesis | K01785 aldose 1-epimerase [EC:5.1.3.3] | "</td></tr>
<tr><td>KEGG | ko00010 Glycolysis / Gluconeogenesis | K01803 triosephosphate isomerase (TIM) [EC:5.3.1.1] | "</td></tr>
<tr><td>KEGG | ko00010 Glycolysis / Gluconeogenesis | K01810 glucose-6-phosphate isomerase [EC:5.3.1.9] | "</td></tr>
<tr><td>KEGG | ko00010 Glycolysis / Gluconeogenesis | K01835 phosphoglucomutase [EC:5.4.2.2] | "</td></tr>
<tr><td>KEGG | ko00010 Glycolysis / Gluconeogenesis | K02777 PTS system, sugar-specific IIA component [EC:2.7.1.-] | "</td></tr>
<tr><td>KEGG | ko00010 Glycolysis / Gluconeogenesis | K03737 pyruvate-ferredoxin/flavodoxin oxidoreductase [EC:1.2.7.1 1.2.7.-] | "</td></tr>
<tr><td>KEGG | ko00010 Glycolysis / Gluconeogenesis | K04041 fructose-1,6-bisphosphatase III [EC:3.1.3.11] | "</td></tr>
<tr><td>KEGG | ko00010 Glycolysis / Gluconeogenesis | K04072 acetaldehyde dehydrogenase / alcohol dehydrogenase [EC:1.2.1.10 1.1.1.1] | "</td></tr>
<tr><td>KEGG | ko00010 Glycolysis / Gluconeogenesis | K13954 alcohol dehydrogenase [EC:1.1.1.1] | "</td></tr>
<tr><td>KEGG | ko00010 Glycolysis / Gluconeogenesis | K15633 2,3-bisphosphoglycerate-independent phosphoglycerate mutase [EC:5.4.2.12] | "</td></tr>
<tr><td>KEGG | ko00010 Glycolysis / Gluconeogenesis | K15634 probable phosphoglycerate mutase [EC:5.4.2.12] | "</td></tr>
<tr><td>KEGG | ko00020 Citrate cycle (TCA cycle) | K00031 isocitrate dehydrogenase [EC:1.1.1.42] | "</td></tr>
<tr><td>KEGG | ko00020 Citrate cycle (TCA cycle) | K00244 fumarate reductase flavoprotein subunit [EC:1.3.5.4] | "</td></tr>
<tr><td>KEGG | ko00020 Citrate cycle (TCA cycle) | K01647 citrate synthase [EC:2.3.3.1] | "</td></tr>
<tr><td>KEGG | ko00020 Citrate cycle (TCA cycle) | K01677 fumarate hydratase subunit alpha [EC:4.2.1.2] | "</td></tr>
<tr><td>KEGG | ko00020 Citrate cycle (TCA cycle) | K01678 fumarate hydratase subunit beta [EC:4.2.1.2] | "</td></tr>
<tr><td>KEGG | ko00020 Citrate cycle (TCA cycle) | K01681 aconitate hydratase [EC:4.2.1.3] | "</td></tr>
<tr><td>KEGG | ko00030 Pentose phosphate pathway | K00033 6-phosphogluconate dehydrogenase [EC:1.1.1.44 1.1.1.343] | "</td></tr>
<tr><td>KEGG | ko00030 Pentose phosphate pathway | K00036 glucose-6-phosphate 1-dehydrogenase [EC:1.1.1.49 1.1.1.363] | "</td></tr>
<tr><td>KEGG | ko00030 Pentose phosphate pathway | K00615 transketolase [EC:2.2.1.1] | "</td></tr>
<tr><td>KEGG | ko00030 Pentose phosphate pathway | K00616 transaldolase [EC:2.2.1.2] | "</td></tr>
<tr><td>KEGG | ko00030 Pentose phosphate pathway | K00948 ribose-phosphate pyrophosphokinase [EC:2.7.6.1] | "</td></tr>
<tr><td>KEGG | ko00030 Pentose phosphate pathway | K01621 xylulose-5-phosphate/fructose-6-phosphate phosphoketolase [EC:4.1.2.9 4.1.2.22] | "</td></tr>
<tr><td>KEGG | ko00030 Pentose phosphate pathway | K01808 ribose 5-phosphate isomerase B [EC:5.3.1.6] | "</td></tr>
<tr><td>KEGG | ko00030 Pentose phosphate pathway | K01839 phosphopentomutase [EC:5.4.2.7] | "</td></tr>
<tr><td>KEGG | ko00500 Starch and sucrose metabolism | K00688 glycogen phosphorylase [EC:2.4.1.1] | "</td></tr>
<tr><td>KEGG | ko00500 Starch and sucrose metabolism | K00700 1,4-alpha-glucan branching enzyme [EC:2.4.1.18] | "</td></tr>
<tr><td>KEGG | ko00500 Starch and sucrose metabolism | K00702 cellobiose phosphorylase [EC:2.4.1.20] | "</td></tr>
<tr><td>KEGG | ko00500 Starch and sucrose metabolism | K00703 starch synthase [EC:2.4.1.21] | "</td></tr>
<tr><td>KEGG | ko00500 Starch and sucrose metabolism | K00705 4-alpha-glucanotransferase [EC:2.4.1.25] | "</td></tr>
<tr><td>KEGG | ko00500 Starch and sucrose metabolism | K00975 glucose-1-phosphate adenylyltransferase [EC:2.7.7.27] | "</td></tr>
<tr><td>KEGG | ko00500 Starch and sucrose metabolism | K01210 glucan 1,3-beta-glucosidase [EC:3.2.1.58] | "</td></tr>
<tr><td>KEGG | ko00500 Starch and sucrose metabolism | K02438 glycogen debranching enzyme [EC:3.2.1.196] | "</td></tr>
<tr><td>KEGG | ko00500 Starch and sucrose metabolism | K02750 PTS system, alpha-glucoside-specific IIC component | "</td></tr>
<tr><td>KEGG | ko00500 Starch and sucrose metabolism | K02761 PTS system, cellobiose-specific IIC component | "</td></tr>
<tr><td>KEGG | ko00500 Starch and sucrose metabolism | K02810 PTS system, sucrose-specific IIC component | "</td></tr>
<tr><td>KEGG | ko00500 Starch and sucrose metabolism | K05343 maltose alpha-D-glucosyltransferase / alpha-amylase [EC:5.4.99.16 3.2.1.1] | "</td></tr>
<tr><td>KEGG | ko00500 Starch and sucrose metabolism | K05349 beta-glucosidase [EC:3.2.1.21] | "</td></tr>
<tr><td>KEGG | ko00500 Starch and sucrose metabolism | K05350 beta-glucosidase [EC:3.2.1.21] | "</td></tr>
<tr><td>KEGG | ko00500 Starch and sucrose metabolism | K16148 alpha-maltose-1-phosphate synthase [EC:2.4.1.342] | "</td></tr>
<tr><td>KEGG | ko00620 Pyruvate metabolism | K00027 malate dehydrogenase (oxaloacetate-decarboxylating) [EC:1.1.1.38] | "</td></tr>
<tr><td>KEGG | ko00620 Pyruvate metabolism | K00625 phosphate acetyltransferase [EC:2.3.1.8] | "</td></tr>
<tr><td>KEGG | ko00620 Pyruvate metabolism | K00656 formate C-acetyltransferase [EC:2.3.1.54] | "</td></tr>
<tr><td>KEGG | ko00620 Pyruvate metabolism | K00925 acetate kinase [EC:2.7.2.1] | "</td></tr>
<tr><td>KEGG | ko00620 Pyruvate metabolism | K01006 pyruvate, orthophosphate dikinase [EC:2.7.9.1] | "</td></tr>
<tr><td>KEGG | ko00620 Pyruvate metabolism | K01026 propionate CoA-transferase [EC:2.8.3.1] | "</td></tr>
<tr><td>KEGG | ko00620 Pyruvate metabolism | K01571 oxaloacetate decarboxylase, alpha subunit [EC:4.1.1.3] | "</td></tr>
<tr><td>KEGG | ko00620 Pyruvate metabolism | K01572 oxaloacetate decarboxylase, beta subunit [EC:4.1.1.3] | "</td></tr>
<tr><td>KEGG | ko00620 Pyruvate metabolism | K01595 phosphoenolpyruvate carboxylase [EC:4.1.1.31] | "</td></tr>
<tr><td>KEGG | ko00620 Pyruvate metabolism | K01759 lactoylglutathione lyase [EC:4.4.1.5] | "</td></tr>
<tr><td>KEGG | ko00620 Pyruvate metabolism | K01961 acetyl-CoA carboxylase, biotin carboxylase subunit [EC:6.4.1.2 6.3.4.14] | "</td></tr>
<tr><td>KEGG | ko00620 Pyruvate metabolism | K01962 acetyl-CoA carboxylase carboxyl transferase subunit alpha [EC:6.4.1.2] | "</td></tr>
<tr><td>KEGG | ko00620 Pyruvate metabolism | K01963 acetyl-CoA carboxylase carboxyl transferase subunit beta [EC:6.4.1.2] | "</td></tr>
<tr><td>KEGG | ko00620 Pyruvate metabolism | K02160 acetyl-CoA carboxylase biotin carboxyl carrier protein | "</td></tr>
<tr><td>KEGG | ko00620 Pyruvate metabolism | K11263 acetyl-CoA/propionyl-CoA carboxylase, biotin carboxylase, biotin carboxyl carrier protein [EC:6.4.1.2 6.4.1.3 6.3.4.14] | "</td></tr>
<tr><td>KEGG | ko00620 Pyruvate metabolism | K13788 phosphate acetyltransferase [EC:2.3.1.8] | "</td></tr>
<tr><td rowspan="2">Butyrate Metabolism</td><td>KEGG | ko00650 Butanoate metabolism | K01652 acetolactate synthase I/II/III large subunit [EC:2.2.1.6] | "</td></tr>
<tr><td>KEGG | ko00650 Butanoate metabolism | K01653 acetolactate synthase I/III small subunit [EC:2.2.1.6] | "</td></tr>
</table>

Supplementary Table 3: All carbohydrate functional annotation derived from metagenomics and metatranscriptomics classification

METAGENOMICS	
**Metabolic Pathway**	**Functional Genes involved in Butyrate and Propionate metabolism based on KEGG database**
Glycolysis-associated Metabolism	KEGG | ko00010 Glycolysis / Gluconeogenesis | K00001 alcohol dehydrogenase [EC:1.1.1.1]
Glycolysis-associated Metabolism	KEGG | ko00010 Glycolysis / Gluconeogenesis | K00002 alcohol dehydrogenase (NADP+) [EC:1.1.1.2]
Glycolysis-associated Metabolism	KEGG | ko00010 Glycolysis / Gluconeogenesis | K00016 L-lactate dehydrogenase [EC:1.1.1.27]
Glycolysis-associated Metabolism	KEGG | ko00010 Glycolysis / Gluconeogenesis | K00114 alcohol dehydrogenase (cytochrome c) [EC:1.1.2.8]
Glycolysis-associated Metabolism	KEGG | ko00010 Glycolysis / Gluconeogenesis | K00121 S-(hydroxymethyl)glutathione dehydrogenase / alcohol dehydrogenase [EC:1.1.1.284 1.1.1.1]
Glycolysis-associated Metabolism	KEGG | ko00010 Glycolysis / Gluconeogenesis | K00128 aldehyde dehydrogenase (NAD+) [EC:1.2.1.3]
Glycolysis-associated Metabolism	KEGG | ko00010 Glycolysis / Gluconeogenesis | K00129 aldehyde dehydrogenase (NAD(P)+) [EC:1.2.1.5]
Glycolysis-associated Metabolism	KEGG | ko00010 Glycolysis / Gluconeogenesis | K00131 glyceraldehyde-3-phosphate dehydrogenase (NADP+) [EC:1.2.1.9]
Glycolysis-associated Metabolism	KEGG | ko00010 Glycolysis / Gluconeogenesis | K00134 glyceraldehyde 3-phosphate dehydrogenase [EC:1.2.1.12]
Glycolysis-associated Metabolism	KEGG | ko00010 Glycolysis / Gluconeogenesis | K00138 aldehyde dehydrogenase [EC:1.2.1.-]
Glycolysis-associated Metabolism	KEGG | ko00010 Glycolysis / Gluconeogenesis | K00149 aldehyde dehydrogenase family 9 member A1 [EC:1.2.1.47 1.2.1.3]
Glycolysis-associated Metabolism	KEGG | ko00010 Glycolysis / Gluconeogenesis | K00150 glyceraldehyde-3-phosphate dehydrogenase (NAD(P)) [EC:1.2.1.59]
Glycolysis-associated Metabolism	KEGG | ko00010 Glycolysis / Gluconeogenesis | K00161 pyruvate dehydrogenase E1 component alpha subunit [EC:1.2.4.1]
Glycolysis-associated Metabolism	KEGG | ko00010 Glycolysis / Gluconeogenesis | K00162 pyruvate dehydrogenase E1 component beta subunit [EC:1.2.4.1]
Glycolysis-associated Metabolism	KEGG | ko00010 Glycolysis / Gluconeogenesis | K00163 pyruvate dehydrogenase E1 component [EC:1.2.4.1]
Glycolysis-associated Metabolism	KEGG | ko00010 Glycolysis / Gluconeogenesis | K00169 pyruvate ferredoxin oxidoreductase alpha subunit [EC:1.2.7.1]
Glycolysis-associated Metabolism	KEGG | ko00010 Glycolysis / Gluconeogenesis | K00170 pyruvate ferredoxin oxidoreductase beta subunit [EC:1.2.7.1]
Glycolysis-associated Metabolism	KEGG | ko00010 Glycolysis / Gluconeogenesis | K00171 pyruvate ferredoxin oxidoreductase delta subunit [EC:1.2.7.1]
Glycolysis-associated Metabolism	KEGG | ko00010 Glycolysis / Gluconeogenesis | K00172 pyruvate ferredoxin oxidoreductase gamma subunit [EC:1.2.7.1]
Glycolysis-associated Metabolism	KEGG | ko00010 Glycolysis / Gluconeogenesis | K00174 2-oxoglutarate/2-oxoacid ferredoxin oxidoreductase subunit alpha [EC:1.2.7.3 1.2.7.11]
Glycolysis-associated Metabolism	KEGG | ko00010 Glycolysis / Gluconeogenesis | K00175 2-oxoglutarate/2-oxoacid ferredoxin oxidoreductase subunit beta [EC:1.2.7.3 1.2.7.11]
Glycolysis-associated Metabolism	KEGG | ko00010 Glycolysis / Gluconeogenesis | K00382 dihydrolipoamide dehydrogenase [EC:1.8.1.4]
Glycolysis-associated Metabolism	KEGG | ko00010 Glycolysis / Gluconeogenesis | K00627 pyruvate dehydrogenase E2 component (dihydrolipoamide acetyltransferase) [EC:2.3.1.12]
Glycolysis-associated Metabolism	KEGG | ko00010 Glycolysis / Gluconeogenesis | K00844 hexokinase [EC:2.7.1.1]
Glycolysis-associated Metabolism	KEGG | ko00010 Glycolysis / Gluconeogenesis | K00845 glucokinase [EC:2.7.1.2]
Glycolysis-associated Metabolism	KEGG | ko00010 Glycolysis / Gluconeogenesis | K00850 6-phosphofructokinase 1 [EC:2.7.1.11]
Glycolysis-associated Metabolism	KEGG | ko00010 Glycolysis / Gluconeogenesis | K00873 pyruvate kinase [EC:2.7.1.40]
Glycolysis-associated Metabolism	KEGG | ko00010 Glycolysis / Gluconeogenesis | K00886 polyphosphate glucokinase [EC:2.7.1.63]
Glycolysis-associated Metabolism	KEGG | ko00010 Glycolysis / Gluconeogenesis | K00895 diphosphate-dependent phosphofructokinase [EC:2.7.1.90]
Glycolysis-associated Metabolism	KEGG | ko00010 Glycolysis / Gluconeogenesis | K00927 phosphoglycerate kinase [EC:2.7.2.3]
Glycolysis-associated Metabolism	KEGG | ko00010 Glycolysis / Gluconeogenesis | K01085 glucose-1-phosphatase [EC:3.1.3.10]
Glycolysis-associated Metabolism	KEGG | ko00010 Glycolysis / Gluconeogenesis | K01086 fructose-1,6-bisphosphatase I / sedoheptulose-1,7-bisphosphatase [EC:3.1.3.11 3.1.3.37]
Glycolysis-associated Metabolism	KEGG | ko00010 Glycolysis / Gluconeogenesis | K01222 6-phospho-beta-glucosidase [EC:3.2.1.86]
Glycolysis-associated Metabolism	KEGG | ko00010 Glycolysis / Gluconeogenesis | K01223 6-phospho-beta-glucosidase [EC:3.2.1.86]
Glycolysis-associated Metabolism	KEGG | ko00010 Glycolysis / Gluconeogenesis | K01568 pyruvate decarboxylase [EC:4.1.1.1]
Glycolysis-associated Metabolism	KEGG | ko00010 Glycolysis / Gluconeogenesis | K01596 phosphoenolpyruvate carboxykinase (GTP) [EC:4.1.1.32]
Glycolysis-associated Metabolism	KEGG | ko00010 Glycolysis / Gluconeogenesis | K01610 phosphoenolpyruvate carboxykinase (ATP) [EC:4.1.1.49]
Glycolysis-associated Metabolism	KEGG | ko00010 Glycolysis / Gluconeogenesis | K01623 fructose-bisphosphate aldolase, class I [EC:4.1.2.13]
Glycolysis-associated Metabolism	KEGG | ko00010 Glycolysis / Gluconeogenesis | K01624 fructose-bisphosphate aldolase, class II [EC:4.1.2.13]
Glycolysis-associated Metabolism	KEGG | ko00010 Glycolysis / Gluconeogenesis | K01689 enolase [EC:4.2.1.11]
Glycolysis-associated Metabolism	KEGG | ko00010 Glycolysis / Gluconeogenesis | K01785 aldose 1-epimerase [EC:5.1.3.3]
Glycolysis-associated Metabolism	KEGG | ko00010 Glycolysis / Gluconeogenesis | K01792 glucose-6-phosphate 1-epimerase [EC:5.1.3.15]
Glycolysis-associated Metabolism	KEGG | ko00010 Glycolysis / Gluconeogenesis | K01803 triosephosphate isomerase (TIM) [EC:5.3.1.1]
Glycolysis-associated Metabolism	KEGG | ko00010 Glycolysis / Gluconeogenesis | K01810 glucose-6-phosphate isomerase [EC:5.3.1.9]
Glycolysis-associated Metabolism	KEGG | ko00010 Glycolysis / Gluconeogenesis | K01834 2,3-bisphosphoglycerate-dependent phosphoglycerate mutase [EC:5.4.2.11]
Glycolysis-associated Metabolism	KEGG | ko00010 Glycolysis / Gluconeogenesis | K01835 phosphoglucomutase [EC:5.4.2.2]
Glycolysis-associated Metabolism	KEGG | ko00010 Glycolysis / Gluconeogenesis | K01895 acetyl-CoA synthetase [EC:6.2.1.1]
Glycolysis-associated Metabolism	KEGG | ko00010 Glycolysis / Gluconeogenesis | K01905 acetate---CoA ligase (ADP-forming) subunit alpha [EC:6.2.1.13]
Glycolysis-associated Metabolism	KEGG | ko00010 Glycolysis / Gluconeogenesis | K02446 fructose-1,6-bisphosphatase II [EC:3.1.3.11]
Glycolysis-associated Metabolism	KEGG | ko00010 Glycolysis / Gluconeogenesis | K02753 PTS system, beta-glucoside (arbutin/salicin/cellobiose)-specific IIC component
Glycolysis-associated Metabolism	KEGG | ko00010 Glycolysis / Gluconeogenesis | K02777 PTS system, sugar-specific IIA component [EC:2.7.1.-]
Glycolysis-associated Metabolism	KEGG | ko00010 Glycolysis / Gluconeogenesis | K02778 PTS system, glucose-specific IIB component [EC:2.7.1.199]
Glycolysis-associated Metabolism	KEGG | ko00010 Glycolysis / Gluconeogenesis | K02779 PTS system, glucose-specific IIC component
Glycolysis-associated Metabolism	KEGG | ko00010 Glycolysis / Gluconeogenesis | K02790 PTS system, maltose/glucose-specific IIB component [EC:2.7.1.199 2.7.1.208]
Glycolysis-associated Metabolism	KEGG | ko00010 Glycolysis / Gluconeogenesis | K02791 PTS system, maltose/glucose-specific IIC component
Glycolysis-associated Metabolism	KEGG | ko00010 Glycolysis / Gluconeogenesis | K03103 multiple inositol-polyphosphate phosphatase / 2,3-bisphosphoglycerate 3-phosphatase [EC:3.1.3.62 3.1.3.80]
Glycolysis-associated Metabolism	KEGG | ko00010 Glycolysis / Gluconeogenesis | K03737 pyruvate-ferredoxin/flavodoxin oxidoreductase [EC:1.2.7.1 1.2.7.-]
Glycolysis-associated Metabolism	KEGG | ko00010 Glycolysis / Gluconeogenesis | K03841 fructose-1,6-bisphosphatase I [EC:3.1.3.11]
Glycolysis-associated Metabolism	KEGG | ko00010 Glycolysis / Gluconeogenesis | K04022 alcohol dehydrogenase
Glycolysis-associated Metabolism	KEGG | ko00010 Glycolysis / Gluconeogenesis | K04041 fructose-1,6-bisphosphatase III [EC:3.1.3.11]
Glycolysis-associated Metabolism	KEGG | ko00010 Glycolysis / Gluconeogenesis | K04072 acetaldehyde dehydrogenase / alcohol dehydrogenase [EC:1.2.1.10 1.1.1.1]
Glycolysis-associated Metabolism	KEGG | ko00010 Glycolysis / Gluconeogenesis | K06859 glucose-6-phosphate isomerase, archaeal [EC:5.3.1.9]
Glycolysis-associated Metabolism	KEGG | ko00010 Glycolysis / Gluconeogenesis | K10705 glyceraldehyde-3-phosphate dehydrogenase, spermatogenic [EC:1.2.1.12]
Glycolysis-associated Metabolism	KEGG | ko00010 Glycolysis / Gluconeogenesis | K11532 fructose-1,6-bisphosphatase II / sedoheptulose-1,7-bisphosphatase [EC:3.1.3.11 3.1.3.37]
Glycolysis-associated Metabolism	KEGG | ko00010 Glycolysis / Gluconeogenesis | K11645 fructose-bisphosphate aldolase, class I [EC:4.1.2.13]
Glycolysis-associated Metabolism	KEGG | ko00010 Glycolysis / Gluconeogenesis | K13951 alcohol dehydrogenase 1/7 [EC:1.1.1.1]
Glycolysis-associated Metabolism	KEGG | ko00010 Glycolysis / Gluconeogenesis | K13953 alcohol dehydrogenase, propanol-preferring [EC:1.1.1.1]
Glycolysis-associated Metabolism	KEGG | ko00010 Glycolysis / Gluconeogenesis | K13954 alcohol dehydrogenase [EC:1.1.1.1]
Glycolysis-associated Metabolism	KEGG | ko00010 Glycolysis / Gluconeogenesis | K14085 aldehyde dehydrogenase family 7 member A1 [EC:1.2.1.31 1.2.1.8 1.2.1.3]
Glycolysis-associated Metabolism	KEGG | ko00010 Glycolysis / Gluconeogenesis | K15633 2,3-bisphosphoglycerate-independent phosphoglycerate mutase [EC:5.4.2.12]
Glycolysis-associated Metabolism	KEGG | ko00010 Glycolysis / Gluconeogenesis | K15634 probable phosphoglycerate mutase [EC:5.4.2.12]
Glycolysis-associated Metabolism	KEGG | ko00010 Glycolysis / Gluconeogenesis | K15635 2,3-bisphosphoglycerate-independent phosphoglycerate mutase [EC:5.4.2.12]
Glycolysis-associated Metabolism	KEGG | ko00010 Glycolysis / Gluconeogenesis | K15778 phosphomannomutase / phosphoglucomutase [EC:5.4.2.8 5.4.2.2]
Glycolysis-associated Metabolism	KEGG | ko00010 Glycolysis / Gluconeogenesis | K15779 phosphoglucomutase / phosphopentomutase [EC:5.4.2.2 5.4.2.7]
Glycolysis-associated Metabolism	KEGG | ko00010 Glycolysis / Gluconeogenesis | K15916 glucose/mannose-6-phosphate isomerase [EC:5.3.1.9 5.3.1.8]
Glycolysis-associated Metabolism	KEGG | ko00010 Glycolysis / Gluconeogenesis | K16306 fructose-bisphosphate aldolase / 2-amino-3,7-dideoxy-D-threo-hept-6-ulosonate synthase [EC:4.1.2.13 2.2.1.10]
Glycolysis-associated Metabolism	KEGG | ko00010 Glycolysis / Gluconeogenesis | K16370 6-phosphofructokinase 2 [EC:2.7.1.11]
Glycolysis-associated Metabolism	KEGG | ko00020 Citrate cycle (TCA cycle) | K00024 malate dehydrogenase [EC:1.1.1.37]
Glycolysis-associated Metabolism	KEGG | ko00020 Citrate cycle (TCA cycle) | K00025 malate dehydrogenase [EC:1.1.1.37]
Glycolysis-associated Metabolism	KEGG | ko00020 Citrate cycle (TCA cycle) | K00026 malate dehydrogenase [EC:1.1.1.37]
Glycolysis-associated Metabolism	KEGG | ko00020 Citrate cycle (TCA cycle) | K00030 isocitrate dehydrogenase (NAD+) [EC:1.1.1.41]
Glycolysis-associated Metabolism	KEGG | ko00020 Citrate cycle (TCA cycle) | K00031 isocitrate dehydrogenase [EC:1.1.1.42]
Glycolysis-associated Metabolism	KEGG | ko00020 Citrate cycle (TCA cycle) | K00116 malate dehydrogenase (quinone) [EC:1.1.5.4]
Glycolysis-associated Metabolism	KEGG | ko00020 Citrate cycle (TCA cycle) | K00164 2-oxoglutarate dehydrogenase E1 component [EC:1.2.4.2]
Glycolysis-associated Metabolism	KEGG | ko00020 Citrate cycle (TCA cycle) | K00176 2-oxoglutarate ferredoxin oxidoreductase subunit delta [EC:1.2.7.3]
Glycolysis-associated Metabolism	KEGG | ko00020 Citrate cycle (TCA cycle) | K00177 2-oxoglutarate ferredoxin oxidoreductase subunit gamma [EC:1.2.7.3]
Glycolysis-associated Metabolism	KEGG | ko00020 Citrate cycle (TCA cycle) | K00234 succinate dehydrogenase (ubiquinone) flavoprotein subunit [EC:1.3.5.1]
Glycolysis-associated Metabolism	KEGG | ko00020 Citrate cycle (TCA cycle) | K00236 succinate dehydrogenase (ubiquinone) cytochrome b560 subunit
Glycolysis-associated Metabolism	KEGG | ko00020 Citrate cycle (TCA cycle) | K00237 succinate dehydrogenase (ubiquinone) membrane anchor subunit
Glycolysis-associated Metabolism	KEGG | ko00020 Citrate cycle (TCA cycle) | K00239 succinate dehydrogenase / fumarate reductase, flavoprotein subunit [EC:1.3.5.1 1.3.5.4]
Glycolysis-associated Metabolism	KEGG | ko00020 Citrate cycle (TCA cycle) | K00240 succinate dehydrogenase / fumarate reductase, iron-sulfur subunit [EC:1.3.5.1 1.3.5.4]
Glycolysis-associated Metabolism	KEGG | ko00020 Citrate cycle (TCA cycle) | K00241 succinate dehydrogenase / fumarate reductase, cytochrome b subunit
Glycolysis-associated Metabolism	KEGG | ko00020 Citrate cycle (TCA cycle) | K00242 succinate dehydrogenase / fumarate reductase, membrane anchor subunit
Glycolysis-associated Metabolism	KEGG | ko00020 Citrate cycle (TCA cycle) | K00244 fumarate reductase flavoprotein subunit [EC:1.3.5.4]
Glycolysis-associated Metabolism	KEGG | ko00020 Citrate cycle (TCA cycle) | K00245 fumarate reductase iron-sulfur subunit [EC:1.3.5.4]
Glycolysis-associated Metabolism	KEGG | ko00020 Citrate cycle (TCA cycle) | K00246 fumarate reductase subunit C
Glycolysis-associated Metabolism	KEGG | ko00020 Citrate cycle (TCA cycle) | K00247 fumarate reductase subunit D
Glycolysis-associated Metabolism	KEGG | ko00020 Citrate cycle (TCA cycle) | K00658 2-oxoglutarate dehydrogenase E2 component (dihydrolipoamide succinyltransferase) [EC:2.3.1.61]
Glycolysis-associated Metabolism	KEGG | ko00020 Citrate cycle (TCA cycle) | K01647 citrate synthase [EC:2.3.3.1]
Glycolysis-associated Metabolism	KEGG | ko00020 Citrate cycle (TCA cycle) | K01648 ATP citrate (pro-S)-lyase [EC:2.3.3.8]

Glycolysis-associated Metabolism	KEGG | ko00020 Citrate cycle (TCA cycle) | K01676 fumarate hydratase, class I [EC:4.2.1.2]
Glycolysis-associated Metabolism	KEGG | ko00020 Citrate cycle (TCA cycle) | K01677 fumarate hydratase subunit alpha [EC:4.2.1.2]
Glycolysis-associated Metabolism	KEGG | ko00020 Citrate cycle (TCA cycle) | K01678 fumarate hydratase subunit beta [EC:4.2.1.2]
Glycolysis-associated Metabolism	KEGG | ko00020 Citrate cycle (TCA cycle) | K01679 fumarate hydratase, class II [EC:4.2.1.2]
Glycolysis-associated Metabolism	KEGG | ko00020 Citrate cycle (TCA cycle) | K01681 aconitate hydratase [EC:4.2.1.3]
Glycolysis-associated Metabolism	KEGG | ko00020 Citrate cycle (TCA cycle) | K01682 aconitate hydratase 2 / 2-methylisocitrate dehydratase [EC:4.2.1.3 4.2.1.99]
Glycolysis-associated Metabolism	KEGG | ko00020 Citrate cycle (TCA cycle) | K01899 succinyl-CoA synthetase alpha subunit [EC:6.2.1.4 6.2.1.5]
Glycolysis-associated Metabolism	KEGG | ko00020 Citrate cycle (TCA cycle) | K01900 succinyl-CoA synthetase beta subunit [EC:6.2.1.4 6.2.1.5]
Glycolysis-associated Metabolism	KEGG | ko00020 Citrate cycle (TCA cycle) | K01902 succinyl-CoA synthetase alpha subunit [EC:6.2.1.5]
Glycolysis-associated Metabolism	KEGG | ko00020 Citrate cycle (TCA cycle) | K01903 succinyl-CoA synthetase beta subunit [EC:6.2.1.5]
Glycolysis-associated Metabolism	KEGG | ko00020 Citrate cycle (TCA cycle) | K01958 pyruvate carboxylase [EC:6.4.1.1]
Glycolysis-associated Metabolism	KEGG | ko00020 Citrate cycle (TCA cycle) | K01959 pyruvate carboxylase subunit A [EC:6.4.1.1]
Glycolysis-associated Metabolism	KEGG | ko00020 Citrate cycle (TCA cycle) | K01960 pyruvate carboxylase subunit B [EC:6.4.1.1]
Glycolysis-associated Metabolism	KEGG | ko00020 Citrate cycle (TCA cycle) | K18118 succinyl-CoA:acetate CoA-transferase [EC:2.8.3.18]
Glycolysis-associated Metabolism	KEGG | ko00030 Pentose phosphate pathway | K00033 6-phosphogluconate dehydrogenase [EC:1.1.1.44 1.1.1.343]
Glycolysis-associated Metabolism	KEGG | ko00030 Pentose phosphate pathway | K00034 glucose 1-dehydrogenase [EC:1.1.1.47]
Glycolysis-associated Metabolism	KEGG | ko00030 Pentose phosphate pathway | K00036 glucose-6-phosphate 1-dehydrogenase [EC:1.1.1.49 1.1.1.363]
Glycolysis-associated Metabolism	KEGG | ko00030 Pentose phosphate pathway | K00090 glyoxylate/hydroxypyruvate/2-ketogluconate reductase [EC:1.1.1.79 1.1.1.81 1.1.1.215]
Glycolysis-associated Metabolism	KEGG | ko00030 Pentose phosphate pathway | K00117 quinoprotein glucose dehydrogenase [EC:1.1.5.2]
Glycolysis-associated Metabolism	KEGG | ko00030 Pentose phosphate pathway | K00615 transketolase [EC:2.2.1.1]
Glycolysis-associated Metabolism	KEGG | ko00030 Pentose phosphate pathway | K00616 transaldolase [EC:2.2.1.2]
Glycolysis-associated Metabolism	KEGG | ko00030 Pentose phosphate pathway | K00851 gluconokinase [EC:2.7.1.12]
Glycolysis-associated Metabolism	KEGG | ko00030 Pentose phosphate pathway | K00852 ribokinase [EC:2.7.1.15]
Glycolysis-associated Metabolism	KEGG | ko00030 Pentose phosphate pathway | K00874 2-dehydro-3-deoxygluconokinase [EC:2.7.1.45]
Glycolysis-associated Metabolism	KEGG | ko00030 Pentose phosphate pathway | K00948 ribose-phosphate pyrophosphokinase [EC:2.7.6.1]
Glycolysis-associated Metabolism	KEGG | ko00030 Pentose phosphate pathway | K01053 gluconolactonase [EC:3.1.1.17]
Glycolysis-associated Metabolism	KEGG | ko00030 Pentose phosphate pathway | K01057 6-phosphogluconolactonase [EC:3.1.1.31]
Glycolysis-associated Metabolism	KEGG | ko00030 Pentose phosphate pathway | K01619 deoxyribose-phosphate aldolase [EC:4.1.2.4]
Glycolysis-associated Metabolism	KEGG | ko00030 Pentose phosphate pathway | K01621 xylulose-5-phosphate/fructose-6-phosphate phosphoketolase [EC:4.1.2.9 4.1.2.22]
Glycolysis-associated Metabolism	KEGG | ko00030 Pentose phosphate pathway | K01625 2-dehydro-3-deoxyphosphogluconate aldolase / (4S)-4-hydroxy-2-oxoglutarate aldolase [EC:4.1.2.14 4.1.3.42]
Glycolysis-associated Metabolism	KEGG | ko00030 Pentose phosphate pathway | K01690 phosphogluconate dehydratase [EC:4.2.1.12]
Glycolysis-associated Metabolism	KEGG | ko00030 Pentose phosphate pathway | K01783 ribulose-phosphate 3-epimerase [EC:5.1.3.1]
Glycolysis-associated Metabolism	KEGG | ko00030 Pentose phosphate pathway | K01807 ribose 5-phosphate isomerase A [EC:5.3.1.6]
Glycolysis-associated Metabolism	KEGG | ko00030 Pentose phosphate pathway | K01808 ribose 5-phosphate isomerase B [EC:5.3.1.6]
Glycolysis-associated Metabolism	KEGG | ko00030 Pentose phosphate pathway | K01839 phosphopentomutase [EC:5.4.2.7]
Glycolysis-associated Metabolism	KEGG | ko00030 Pentose phosphate pathway | K03738 aldehyde:ferredoxin oxidoreductase [EC:1.2.7.5]
Glycolysis-associated Metabolism	KEGG | ko00030 Pentose phosphate pathway | K05774 ribose 1,5-bisphosphokinase [EC:2.7.4.23]
Glycolysis-associated Metabolism	KEGG | ko00030 Pentose phosphate pathway | K06151 gluconate 2-dehydrogenase alpha chain [EC:1.1.99.3]
Glycolysis-associated Metabolism	KEGG | ko00030 Pentose phosphate pathway | K06152 gluconate 2-dehydrogenase gamma chain [EC:1.1.99.3]
Glycolysis-associated Metabolism	KEGG | ko00030 Pentose phosphate pathway | K07404 6-phosphogluconolactonase [EC:3.1.1.31]
Glycolysis-associated Metabolism	KEGG | ko00030 Pentose phosphate pathway | K08093 3-hexulose-6-phosphate synthase [EC:4.1.2.43]
Glycolysis-associated Metabolism	KEGG | ko00030 Pentose phosphate pathway | K08094 6-phospho-3-hexuloisomerase [EC:5.3.1.27]
Glycolysis-associated Metabolism	KEGG | ko00030 Pentose phosphate pathway | K11441 dehydrogluconokinase [EC:2.7.1.13]
Glycolysis-associated Metabolism	KEGG | ko00030 Pentose phosphate pathway | K11529 glycerate 2-kinase [EC:2.7.1.165]
Glycolysis-associated Metabolism	KEGG | ko00030 Pentose phosphate pathway | K13937 hexose-6-phosphate dehydrogenase [EC:1.1.1.47 3.1.1.31]
Glycolysis-associated Metabolism	KEGG | ko00030 Pentose phosphate pathway | K17463 2-dehydro-3-deoxy-phosphogluconate aldolase [EC:4.1.2.14]
Glycolysis-associated Metabolism	KEGG | ko00030 Pentose phosphate pathway | K17464 PTS system, D-glucosaminate-specific IIA component [EC:2.7.1.203]
Glycolysis-associated Metabolism	KEGG | ko00030 Pentose phosphate pathway | K17465 PTS system, D-glucosaminate-specific IIB component [EC:2.7.1.203]
Glycolysis-associated Metabolism	KEGG | ko00030 Pentose phosphate pathway | K17466 PTS system, D-glucosaminate-specific IIC component
Glycolysis-associated Metabolism	KEGG | ko00030 Pentose phosphate pathway | K17467 PTS system, D-glucosaminate-specific IID component
Glycolysis-associated Metabolism	KEGG | ko00030 Pentose phosphate pathway | K17468 D-glucosaminate-6-phosphate ammonia-lyase [EC:4.3.1.29]
Glycolysis-associated Metabolism	KEGG | ko00500 Starch and sucrose metabolism | K00688 glycogen phosphorylase [EC:2.4.1.1]
Glycolysis-associated Metabolism	KEGG | ko00500 Starch and sucrose metabolism | K00689 dextransucrase [EC:2.4.1.5]
Glycolysis-associated Metabolism	KEGG | ko00500 Starch and sucrose metabolism | K00690 sucrose phosphorylase [EC:2.4.1.7]
Glycolysis-associated Metabolism	KEGG | ko00500 Starch and sucrose metabolism | K00691 maltose phosphorylase [EC:2.4.1.8]
Glycolysis-associated Metabolism	KEGG | ko00500 Starch and sucrose metabolism | K00692 levansucrase [EC:2.4.1.10]
Glycolysis-associated Metabolism	KEGG | ko00500 Starch and sucrose metabolism | K00693 glycogen synthase [EC:2.4.1.11]
Glycolysis-associated Metabolism	KEGG | ko00500 Starch and sucrose metabolism | K00694 cellulose synthase (UDP-forming) [EC:2.4.1.12]
Glycolysis-associated Metabolism	KEGG | ko00500 Starch and sucrose metabolism | K00695 sucrose synthase [EC:2.4.1.13]
Glycolysis-associated Metabolism	KEGG | ko00500 Starch and sucrose metabolism | K00696 sucrose-phosphate synthase [EC:2.4.1.14]
Glycolysis-associated Metabolism	KEGG | ko00500 Starch and sucrose metabolism | K00697 trehalose 6-phosphate synthase [EC:2.4.1.15]
Glycolysis-associated Metabolism	KEGG | ko00500 Starch and sucrose metabolism | K00700 1,4-alpha-glucan branching enzyme [EC:2.4.1.18]
Glycolysis-associated Metabolism	KEGG | ko00500 Starch and sucrose metabolism | K00702 cellobiose phosphorylase [EC:2.4.1.20]
Glycolysis-associated Metabolism	KEGG | ko00500 Starch and sucrose metabolism | K00703 starch synthase [EC:2.4.1.21]
Glycolysis-associated Metabolism	KEGG | ko00500 Starch and sucrose metabolism | K00705 4-alpha-glucanotransferase [EC:2.4.1.25]
Glycolysis-associated Metabolism	KEGG | ko00500 Starch and sucrose metabolism | K00706 1,3-beta-glucan synthase [EC:2.4.1.34]
Glycolysis-associated Metabolism	KEGG | ko00500 Starch and sucrose metabolism | K00975 glucose-1-phosphate adenylyltransferase [EC:2.7.7.27]
Glycolysis-associated Metabolism	KEGG | ko00500 Starch and sucrose metabolism | K00978 glucose-1-phosphate cytidylyltransferase [EC:2.7.7.33]
Glycolysis-associated Metabolism	KEGG | ko00500 Starch and sucrose metabolism | K01087 trehalose 6-phosphate phosphatase [EC:3.1.3.12]
Glycolysis-associated Metabolism	KEGG | ko00500 Starch and sucrose metabolism | K01176 alpha-amylase [EC:3.2.1.1]
Glycolysis-associated Metabolism	KEGG | ko00500 Starch and sucrose metabolism | K01177 beta-amylase [EC:3.2.1.2]
Glycolysis-associated Metabolism	KEGG | ko00500 Starch and sucrose metabolism | K01178 glucoamylase [EC:3.2.1.3]
Glycolysis-associated Metabolism	KEGG | ko00500 Starch and sucrose metabolism | K01179 endoglucanase [EC:3.2.1.4]
Glycolysis-associated Metabolism	KEGG | ko00500 Starch and sucrose metabolism | K01188 beta-glucosidase [EC:3.2.1.21]
Glycolysis-associated Metabolism	KEGG | ko00500 Starch and sucrose metabolism | K01194 alpha,alpha-trehalase [EC:3.2.1.28]
Glycolysis-associated Metabolism	KEGG | ko00500 Starch and sucrose metabolism | K01196 glycogen debranching enzyme [EC:2.4.1.25 3.2.1.33]
Glycolysis-associated Metabolism	KEGG | ko00500 Starch and sucrose metabolism | K01208 cyclomaltodextrinase / maltogenic alpha-amylase / neopullulanase [EC:3.2.1.54 3.2.1.133 3.2.1.135]
Glycolysis-associated Metabolism	KEGG | ko00500 Starch and sucrose metabolism | K01210 glucan 1,3-beta-glucosidase [EC:3.2.1.58]
Glycolysis-associated Metabolism	KEGG | ko00500 Starch and sucrose metabolism | K01212 levanase [EC:3.2.1.65]
Glycolysis-associated Metabolism	KEGG | ko00500 Starch and sucrose metabolism | K01226 trehalose-6-phosphate hydrolase [EC:3.2.1.93]
Glycolysis-associated Metabolism	KEGG | ko00500 Starch and sucrose metabolism | K01232 maltose-6'-phosphate glucosidase [EC:3.2.1.122]
Glycolysis-associated Metabolism	KEGG | ko00500 Starch and sucrose metabolism | K01236 maltooligosyltrehalose trehalohydrolase [EC:3.2.1.141]
Glycolysis-associated Metabolism	KEGG | ko00500 Starch and sucrose metabolism | K01513 ectonucleotide pyrophosphatase/phosphodiesterase 1 [HSA:5167]
Glycolysis-associated Metabolism	KEGG | ko00500 Starch and sucrose metabolism | K01838 beta-phosphoglucomutase [EC:5.4.2.6]
Glycolysis-associated Metabolism	KEGG | ko00500 Starch and sucrose metabolism | K02438 glycogen debranching enzyme [EC:3.2.1.196]
Glycolysis-associated Metabolism	KEGG | ko00500 Starch and sucrose metabolism | K02750 PTS system, alpha-glucoside-specific IIC component
Glycolysis-associated Metabolism	KEGG | ko00500 Starch and sucrose metabolism | K02759 PTS system, cellobiose-specific IIA component [EC:2.7.1.196 2.7.1.205]
Glycolysis-associated Metabolism	KEGG | ko00500 Starch and sucrose metabolism | K02760 PTS system, cellobiose-specific IIB component [EC:2.7.1.196 2.7.1.205]
Glycolysis-associated Metabolism	KEGG | ko00500 Starch and sucrose metabolism | K02761 PTS system, cellobiose-specific IIC component
Glycolysis-associated Metabolism	KEGG | ko00500 Starch and sucrose metabolism | K02809 PTS system, sucrose-specific IIB component [EC:2.7.1.211]
Glycolysis-associated Metabolism	KEGG | ko00500 Starch and sucrose metabolism | K02810 PTS system, sucrose-specific IIC component
Glycolysis-associated Metabolism	KEGG | ko00500 Starch and sucrose metabolism | K02817 PTS system, trehalose-specific IIA component [EC:2.7.1.201]
Glycolysis-associated Metabolism	KEGG | ko00500 Starch and sucrose metabolism | K02819 PTS system, trehalose-specific IIC component
Glycolysis-associated Metabolism	KEGG | ko00500 Starch and sucrose metabolism | K05341 amylosucrase [EC:2.4.1.4]
Glycolysis-associated Metabolism	KEGG | ko00500 Starch and sucrose metabolism | K05342 alpha,alpha-trehalose phosphorylase [EC:2.4.1.64]
Glycolysis-associated Metabolism	KEGG | ko00500 Starch and sucrose metabolism | K05343 maltose alpha-D-glucosyltransferase / alpha-amylase [EC:5.4.99.16 3.2.1.1]
Glycolysis-associated Metabolism	KEGG | ko00500 Starch and sucrose metabolism | K05349 beta-glucosidase [EC:3.2.1.21]
Glycolysis-associated Metabolism	KEGG | ko00500 Starch and sucrose metabolism | K05350 beta-glucosidase [EC:3.2.1.21]
Glycolysis-associated Metabolism	KEGG | ko00500 Starch and sucrose metabolism | K06044 (1->4)-alpha-D-glucan 1-alpha-D-glucosylmutase [EC:5.4.99.15]
Glycolysis-associated Metabolism	KEGG | ko00500 Starch and sucrose metabolism | K06896 maltose 6'-phosphate phosphatase [EC:3.1.3.90]
Glycolysis-associated Metabolism	KEGG | ko00500 Starch and sucrose metabolism | K07405 alpha-amylase [EC:3.2.1.1]
Glycolysis-associated Metabolism	KEGG | ko00500 Starch and sucrose metabolism | K16055 trehalose 6-phosphate synthase/phosphatase [EC:2.4.1.15 3.1.3.12]
Glycolysis-associated Metabolism	KEGG | ko00500 Starch and sucrose metabolism | K16146 maltokinase [EC:2.7.1.175]
Glycolysis-associated Metabolism	KEGG | ko00500 Starch and sucrose metabolism | K16147 starch synthase (maltosyl-transferring) [EC:2.4.99.16]
Glycolysis-associated Metabolism	KEGG | ko00500 Starch and sucrose metabolism | K16148 alpha-maltose-1-phosphate synthase [EC:2.4.1.342]

Glycolysis-associated Metabolism	KEGG | ko00500 Starch and sucrose metabolism | K16149 1,4-alpha-glucan branching enzyme [EC:2.4.1.18]
Glycolysis-associated Metabolism	KEGG | ko00500 Starch and sucrose metabolism | K18775 levanbiose-producing levanase [EC:3.2.1.64]
Glycolysis-associated Metabolism	KEGG | ko00500 Starch and sucrose metabolism | K19893 glucan endo-1,3-beta-glucosidase 5/6 [EC:3.2.1.39]
Glycolysis-associated Metabolism	KEGG | ko00620 Pyruvate metabolism | K00027 malate dehydrogenase (oxaloacetate-decarboxylating) [EC:1.1.1.38]
Glycolysis-associated Metabolism	KEGG | ko00620 Pyruvate metabolism | K00028 malate dehydrogenase (decarboxylating) [EC:1.1.1.39]
Glycolysis-associated Metabolism	KEGG | ko00620 Pyruvate metabolism | K00029 malate dehydrogenase (oxaloacetate-decarboxylating)(NADP+) [EC:1.1.1.40]
Glycolysis-associated Metabolism	KEGG | ko00620 Pyruvate metabolism | K00049 glyoxylate/hydroxypyruvate reductase [EC:1.1.1.79 1.1.1.81]
Glycolysis-associated Metabolism	KEGG | ko00620 Pyruvate metabolism | K00101 L-lactate dehydrogenase (cytochrome) [EC:1.1.2.3]
Glycolysis-associated Metabolism	KEGG | ko00620 Pyruvate metabolism | K00102 D-lactate dehydrogenase (cytochrome) [EC:1.1.2.4]
Glycolysis-associated Metabolism	KEGG | ko00620 Pyruvate metabolism | K00132 acetaldehyde dehydrogenase (acetylating) [EC:1.2.1.10]
Glycolysis-associated Metabolism	KEGG | ko00620 Pyruvate metabolism | K00156 pyruvate dehydrogenase (quinone) [EC:1.2.5.1]
Glycolysis-associated Metabolism	KEGG | ko00620 Pyruvate metabolism | K00158 pyruvate oxidase [EC:1.2.3.3]
Glycolysis-associated Metabolism	KEGG | ko00620 Pyruvate metabolism | K00625 phosphate acetyltransferase [EC:2.3.1.8]
Glycolysis-associated Metabolism	KEGG | ko00620 Pyruvate metabolism | K00626 acetyl-CoA C-acetyltransferase [EC:2.3.1.9]
Glycolysis-associated Metabolism	KEGG | ko00620 Pyruvate metabolism | K00656 formate C-acetyltransferase [EC:2.3.1.54]
Glycolysis-associated Metabolism	KEGG | ko00620 Pyruvate metabolism | K00925 acetate kinase [EC:2.7.2.1]
Glycolysis-associated Metabolism	KEGG | ko00620 Pyruvate metabolism | K01003 oxaloacetate decarboxylase [EC:4.1.1.3]
Glycolysis-associated Metabolism	KEGG | ko00620 Pyruvate metabolism | K01006 pyruvate, orthophosphate dikinase [EC:2.7.9.1]
Glycolysis-associated Metabolism	KEGG | ko00620 Pyruvate metabolism | K01007 pyruvate, water dikinase [EC:2.7.9.2]
Glycolysis-associated Metabolism	KEGG | ko00620 Pyruvate metabolism | K01026 propionate CoA-transferase [EC:2.8.3.1]
Glycolysis-associated Metabolism	KEGG | ko00620 Pyruvate metabolism | K01067 acetyl-CoA hydrolase [EC:3.1.2.1]
Glycolysis-associated Metabolism	KEGG | ko00620 Pyruvate metabolism | K01069 hydroxyacylglutathione hydrolase [EC:3.1.2.6]
Glycolysis-associated Metabolism	KEGG | ko00620 Pyruvate metabolism | K01512 acylphosphatase [EC:3.6.1.7]
Glycolysis-associated Metabolism	KEGG | ko00620 Pyruvate metabolism | K01571 oxaloacetate decarboxylase, alpha subunit [EC:4.1.1.3]
Glycolysis-associated Metabolism	KEGG | ko00620 Pyruvate metabolism | K01572 oxaloacetate decarboxylase, beta subunit [EC:4.1.1.3]
Glycolysis-associated Metabolism	KEGG | ko00620 Pyruvate metabolism | K01573 oxaloacetate decarboxylase, gamma subunit [EC:4.1.1.3]
Glycolysis-associated Metabolism	KEGG | ko00620 Pyruvate metabolism | K01595 phosphoenolpyruvate carboxylase [EC:4.1.1.31]
Glycolysis-associated Metabolism	KEGG | ko00620 Pyruvate metabolism | K01638 malate synthase [EC:2.3.3.9]
Glycolysis-associated Metabolism	KEGG | ko00620 Pyruvate metabolism | K01649 2-isopropylmalate synthase [EC:2.3.3.13]
Glycolysis-associated Metabolism	KEGG | ko00620 Pyruvate metabolism | K01655 homocitrate synthase [EC:2.3.3.14]
Glycolysis-associated Metabolism	KEGG | ko00620 Pyruvate metabolism | K01759 lactoylglutathione lyase [EC:4.4.1.5]
Glycolysis-associated Metabolism	KEGG | ko00620 Pyruvate metabolism | K01946 acetyl-CoA carboxylase / biotin carboxylase 2 [EC:6.4.1.2 6.3.4.14]
Glycolysis-associated Metabolism	KEGG | ko00620 Pyruvate metabolism | K01961 acetyl-CoA carboxylase, biotin carboxylase subunit [EC:6.4.1.2 6.3.4.14]
Glycolysis-associated Metabolism	KEGG | ko00620 Pyruvate metabolism | K01962 acetyl-CoA carboxylase carboxyl transferase subunit alpha [EC:6.4.1.2]
Glycolysis-associated Metabolism	KEGG | ko00620 Pyruvate metabolism | K01963 acetyl-CoA carboxylase carboxyl transferase subunit beta [EC:6.4.1.2]
Glycolysis-associated Metabolism	KEGG | ko00620 Pyruvate metabolism | K02160 acetyl-CoA carboxylase biotin carboxyl carrier protein
Glycolysis-associated Metabolism	KEGG | ko00620 Pyruvate metabolism | K02594 homocitrate synthase NifV [EC:2.3.3.14]
Glycolysis-associated Metabolism	KEGG | ko00620 Pyruvate metabolism | K03777 D-lactate dehydrogenase (quinone) [EC:1.1.5.12]
Glycolysis-associated Metabolism	KEGG | ko00620 Pyruvate metabolism | K03778 D-lactate dehydrogenase [EC:1.1.1.28]
Glycolysis-associated Metabolism	KEGG | ko00620 Pyruvate metabolism | K04020 phosphotransacetylase
Glycolysis-associated Metabolism	KEGG | ko00620 Pyruvate metabolism | K04021 aldehyde dehydrogenase
Glycolysis-associated Metabolism	KEGG | ko00620 Pyruvate metabolism | K04073 acetaldehyde dehydrogenase [EC:1.2.1.10]
Glycolysis-associated Metabolism	KEGG | ko00620 Pyruvate metabolism | K05523 D-lactate dehydratase / protein deglycase [EC:4.2.1.130 3.5.1.124]
Glycolysis-associated Metabolism	KEGG | ko00620 Pyruvate metabolism | K07248 lactaldehyde dehydrogenase / glycolaldehyde dehydrogenase [EC:1.2.1.22 1.2.1.21]
Glycolysis-associated Metabolism	KEGG | ko00620 Pyruvate metabolism | K11262 acetyl-CoA carboxylase / biotin carboxylase 1 [EC:6.4.1.2 6.3.4.14]
Glycolysis-associated Metabolism	KEGG | ko00620 Pyruvate metabolism | K11263 acetyl-CoA/propionyl-CoA carboxylase, biotin carboxylase, biotin carboxyl carrier protein [EC:6.4.1.2 6.4.1.3 6.3.4.14]
Glycolysis-associated Metabolism	KEGG | ko00620 Pyruvate metabolism | K12972 glyoxylate/hydroxypyruvatereductase [EC:1.1.1.79 1.1.1.81]
Glycolysis-associated Metabolism	KEGG | ko00620 Pyruvate metabolism | K13788 phosphate acetyltransferase [EC:2.3.1.8]
Glycolysis-associated Metabolism	KEGG | ko00620 Pyruvate metabolism | K15024 putative phosphotransacetylase [EC:2.3.1.8]
Glycolysis-associated Metabolism	KEGG | ko00620 Pyruvate metabolism | K17741 NADPH-dependent methylglyoxal reductase [EC:1.1.1.283]
Propionate Metabolism	KEGG | ko00640 Propanoate metabolism | K00005 glycerol dehydrogenase [EC:1.1.1.6]
Propionate Metabolism	KEGG | ko00640 Propanoate metabolism | K00086 1,3-propanediol dehydrogenase [EC:1.1.1.202]
Propionate Metabolism	KEGG | ko00640 Propanoate metabolism | K00140 malonate-semialdehyde dehydrogenase (acetylating) / methylmalonate-semialdehyde dehydrogenase [EC:1.2.1.18 1.2.1.27]
Propionate Metabolism	KEGG | ko00640 Propanoate metabolism | K00166 2-oxoisovalerate dehydrogenase E1 component alpha subunit [EC:1.2.4.4]
Propionate Metabolism	KEGG | ko00640 Propanoate metabolism | K00167 2-oxoisovalerate dehydrogenase E1 component beta subunit [EC:1.2.4.4]
Propionate Metabolism	KEGG | ko00640 Propanoate metabolism | K00249 acyl-CoA dehydrogenase [EC:1.3.8.7]
Propionate Metabolism	KEGG | ko00640 Propanoate metabolism | K00822 beta-alanine--pyruvate transaminase [EC:2.6.1.18]
Propionate Metabolism	KEGG | ko00640 Propanoate metabolism | K00823 4-aminobutyrate aminotransferase [EC:2.6.1.19]
Propionate Metabolism	KEGG | ko00640 Propanoate metabolism | K00932 propionate kinase [EC:2.7.2.15]
Propionate Metabolism	KEGG | ko00640 Propanoate metabolism | K01034 acetate CoA/acetoacetate CoA-transferase alpha subunit [EC:2.8.3.8 2.8.3.9]
Propionate Metabolism	KEGG | ko00640 Propanoate metabolism | K01035 acetate CoA/acetoacetate CoA-transferase beta subunit [EC:2.8.3.8 2.8.3.9]
Propionate Metabolism	KEGG | ko00640 Propanoate metabolism | K01574 acetoacetate decarboxylase [EC:4.1.1.4]
Propionate Metabolism	KEGG | ko00640 Propanoate metabolism | K01578 malonyl-CoA decarboxylase [EC:4.1.1.9]
Propionate Metabolism	KEGG | ko00640 Propanoate metabolism | K01659 2-methylcitrate synthase [EC:2.3.3.5]
Propionate Metabolism	KEGG | ko00640 Propanoate metabolism | K01692 enoyl-CoA hydratase [EC:4.2.1.17]
Propionate Metabolism	KEGG | ko00640 Propanoate metabolism | K01699 propanediol dehydratase large subunit [EC:4.2.1.28]
Propionate Metabolism	KEGG | ko00640 Propanoate metabolism | K01720 2-methylcitrate dehydratase [EC:4.2.1.79]
Propionate Metabolism	KEGG | ko00640 Propanoate metabolism | K01734 methylglyoxal synthase [EC:4.2.3.3]
Propionate Metabolism	KEGG | ko00640 Propanoate metabolism | K01782 3-hydroxyacyl-CoA dehydrogenase / enoyl-CoA hydratase / 3-hydroxybutyryl-CoA epimerase [EC:1.1.1.35 4.2.1.17 5.1.2.3]
Propionate Metabolism	KEGG | ko00640 Propanoate metabolism | K01825 3-hydroxyacyl-CoA dehydrogenase / enoyl-CoA hydratase / 3-hydroxybutyryl-CoA epimerase / enoyl-CoA isomerase [EC:1.1.1.35 4.2.1.17 5.1.2.3 5.3.3.8]
Propionate Metabolism	KEGG | ko00640 Propanoate metabolism | K01908 propionyl-CoA synthetase [EC:6.2.1.17]
Propionate Metabolism	KEGG | ko00640 Propanoate metabolism | K03416 methylmalonyl-CoA carboxyltransferase 5S subunit [EC:2.1.3.1]
Propionate Metabolism	KEGG | ko00640 Propanoate metabolism | K03417 methylisocitrate lyase [EC:4.1.3.30]
Propionate Metabolism	KEGG | ko00640 Propanoate metabolism | K05605 3-hydroxyisobutyryl-CoA hydrolase [EC:3.1.2.4]
Propionate Metabolism	KEGG | ko00640 Propanoate metabolism | K07250 4-aminobutyrate aminotransferase / (S)-3-amino-2-methylpropionate transaminase [EC:2.6.1.19 2.6.1.22]
Propionate Metabolism	KEGG | ko00640 Propanoate metabolism | K07511 enoyl-CoA hydratase [EC:4.2.1.17]
Propionate Metabolism	KEGG | ko00640 Propanoate metabolism | K07515 enoyl-CoA hydratase / long-chain 3-hydroxyacyl-CoA dehydrogenase [EC:4.2.1.17 1.1.1.211]
Propionate Metabolism	KEGG | ko00640 Propanoate metabolism | K08325 NADP-dependent alcohol dehydrogenase [EC:1.1.-.-]
Propionate Metabolism	KEGG | ko00640 Propanoate metabolism | K09699 2-oxoisovalerate dehydrogenase E2 component (dihydrolipoyl transacylase) [EC:2.3.1.168]
Propionate Metabolism	KEGG | ko00640 Propanoate metabolism | K11264 methylmalonyl-CoA decarboxylase [EC:4.1.1.41]
Propionate Metabolism	KEGG | ko00640 Propanoate metabolism | K11381 2-oxoisovalerate dehydrogenase E1 component [EC:1.2.4.4]
Propionate Metabolism	KEGG | ko00640 Propanoate metabolism | K13524 4-aminobutyrate aminotransferase / (S)-3-amino-2-methylpropionate transaminase [EC:2.6.1.19 2.6.1.22]
Propionate Metabolism	KEGG | ko00640 Propanoate metabolism | K13919 propanediol dehydratase medium subunit [EC:4.2.1.28]
Propionate Metabolism	KEGG | ko00640 Propanoate metabolism | K13920 propanediol dehydratase small subunit [EC:4.2.1.28]
Propionate Metabolism	KEGG | ko00640 Propanoate metabolism | K13921 1-propanol dehydrogenase
Propionate Metabolism	KEGG | ko00640 Propanoate metabolism | K13922 propionaldehyde dehydrogenase [EC:1.2.1.87]
Propionate Metabolism	KEGG | ko00640 Propanoate metabolism | K13923 phosphate propanoyltransferase [EC:2.3.1.222]
Propionate Metabolism	KEGG | ko00640 Propanoate metabolism | K17489 methylmalonyl-CoA carboxyltransferase 12S subunit [EC:2.1.3.1]
Propionate Metabolism	KEGG | ko00640 Propanoate metabolism | K17490 methylmalonyl-CoA carboxyltransferase 1.3S subunit [EC:2.1.3.1]
Propionate Metabolism	KEGG | ko00640 Propanoate metabolism | K18369 alcohol dehydrogenase [EC:1.1.1.-]
Propionate Metabolism	KEGG | ko00640 Propanoate metabolism | K18372 methyl acetate hydrolase [EC:3.1.1.-]
Propionate Metabolism	KEGG | ko00640 Propanoate metabolism | K18382 NAD+-dependent secondary alcohol dehydrogenase Adh1 [EC:1.1.1.-]
Propionate Metabolism	KEGG | ko00640 Propanoate metabolism | K18471 methylglyoxal reductase [EC:1.1.1.-]
Propionate Metabolism	KEGG | ko00640 Propanoate metabolism | K19697 propionate kinase [EC:2.7.2.15]
Propionate Metabolism	KEGG | ko00640 Propanoate metabolism | K19709 acetate CoA-transferase [EC:2.8.3.8]
Propionate Metabolism	KEGG | ko00640 Propanoate metabolism | K19745 acrylyl-CoA reductase (NADPH) [EC:1.3.1.-]
Butyrate Metabolism	KEGG | ko00650 Butanoate metabolism | K00004 (R,R)-butanediol dehydrogenase / meso-butanediol dehydrogenase / diacetyl reductase [EC:1.1.1.4 1.1.1.- 1.1.1.303]
Butyrate Metabolism	KEGG | ko00650 Butanoate metabolism | K00019 3-hydroxybutyrate dehydrogenase [EC:1.1.1.30]
Butyrate Metabolism	KEGG | ko00650 Butanoate metabolism | K00074 3-hydroxybutyryl-CoA dehydrogenase [EC:1.1.1.157]
Butyrate Metabolism	KEGG | ko00650 Butanoate metabolism | K00100 butanol dehydrogenase [EC:1.1.1.-]
Butyrate Metabolism	KEGG | ko00650 Butanoate metabolism | K00109 2-hydroxyglutarate dehydrogenase [EC:1.1.99.2]
Butyrate Metabolism	KEGG | ko00650 Butanoate metabolism | K00135 succinate-semialdehyde dehydrogenase / glutarate-semialdehyde dehydrogenase [EC:1.2.1.16 1.2.1.79 1.2.1.20]
Butyrate Metabolism	KEGG | ko00650 Butanoate metabolism | K00209 enoyl-[acyl-carrier protein] reductase / trans-2-enoyl-CoA reductase (NAD+) [EC:1.3.1.9 1.3.1.44]
Butyrate Metabolism	KEGG | ko00650 Butanoate metabolism | K00248 butyryl-CoA dehydrogenase [EC:1.3.8.1]
Butyrate Metabolism	KEGG | ko00650 Butanoate metabolism | K00634 phosphate butyryltransferase [EC:2.3.1.19]

Butyrate Metabolism	KEGG | ko00650 Butanoate metabolism | K00929 butyrate kinase [EC:2.7.2.7]
Butyrate Metabolism	KEGG | ko00650 Butanoate metabolism | K01027 3-oxoacid CoA-transferase [EC:2.8.3.5]
Butyrate Metabolism	KEGG | ko00650 Butanoate metabolism | K01028 3-oxoacid CoA-transferase subunit A [EC:2.8.3.5]
Butyrate Metabolism	KEGG | ko00650 Butanoate metabolism | K01029 3-oxoacid CoA-transferase subunit B [EC:2.8.3.5]
Butyrate Metabolism	KEGG | ko00650 Butanoate metabolism | K01039 glutaconate CoA-transferase, subunit A [EC:2.8.3.12]
Butyrate Metabolism	KEGG | ko00650 Butanoate metabolism | K01040 glutaconate CoA-transferase, subunit B [EC:2.8.3.12]
Butyrate Metabolism	KEGG | ko00650 Butanoate metabolism | K01575 acetolactate decarboxylase [EC:4.1.1.5]
Butyrate Metabolism	KEGG | ko00650 Butanoate metabolism | K01580 glutamate decarboxylase [EC:4.1.1.15]
Butyrate Metabolism	KEGG | ko00650 Butanoate metabolism | K01615 glutaconyl-CoA decarboxylase [EC:4.1.1.70]
Butyrate Metabolism	KEGG | ko00650 Butanoate metabolism | K01640 hydroxymethylglutaryl-CoA lyase [EC:4.1.3.4]
Butyrate Metabolism	KEGG | ko00650 Butanoate metabolism | K01641 hydroxymethylglutaryl-CoA synthase [EC:2.3.3.10]
Butyrate Metabolism	KEGG | ko00650 Butanoate metabolism | K01652 acetolactate synthase I/II/III large subunit [EC:2.2.1.6]
Butyrate Metabolism	KEGG | ko00650 Butanoate metabolism | K01653 acetolactate synthase I/III small subunit [EC:2.2.1.6]
Butyrate Metabolism	KEGG | ko00650 Butanoate metabolism | K01715 enoyl-CoA hydratase [EC:4.2.1.17]
Butyrate Metabolism	KEGG | ko00650 Butanoate metabolism | K01799 maleate isomerase [EC:5.2.1.1]
Butyrate Metabolism	KEGG | ko00650 Butanoate metabolism | K01896 medium-chain acyl-CoA synthetase [EC:6.2.1.2]
Butyrate Metabolism	KEGG | ko00650 Butanoate metabolism | K01907 acetoacetyl-CoA synthetase [EC:6.2.1.16]
Butyrate Metabolism	KEGG | ko00650 Butanoate metabolism | K03366 meso-butanediol dehydrogenase / (S,S)-butanediol dehydrogenase / diacetyl reductase [EC:1.1.1.- 1.1.1.76 1.1.1.304]
Butyrate Metabolism	KEGG | ko00650 Butanoate metabolism | K03821 polyhydroxyalkanoate synthase [EC:2.3.1.-]
Butyrate Metabolism	KEGG | ko00650 Butanoate metabolism | K05973 poly(3-hydroxybutyrate) depolymerase [EC:3.1.1.75]
Butyrate Metabolism	KEGG | ko00650 Butanoate metabolism | K07516 3-hydroxyacyl-CoA dehydrogenase [EC:1.1.1.35]
Butyrate Metabolism	KEGG | ko00650 Butanoate metabolism | K08318 4-hydroxybutyrate dehydrogenase / sulfolactaldehyde 3-reductase [EC:1.1.1.61 1.1.1.373]
Butyrate Metabolism	KEGG | ko00650 Butanoate metabolism | K08324 succinate-semialdehyde dehydrogenase [EC:1.2.1.16 1.2.1.24]
Butyrate Metabolism	KEGG | ko00650 Butanoate metabolism | K11258 acetolactate synthase II small subunit [EC:2.2.1.6]
Butyrate Metabolism	KEGG | ko00650 Butanoate metabolism | K14268 5-aminovalerate/4-aminobutyrate aminotransferase [EC:2.6.1.48 2.6.1.19]
Butyrate Metabolism	KEGG | ko00650 Butanoate metabolism | K14534 4-hydroxybutyryl-CoA dehydratase / vinylacetyl-CoA-Delta-isomerase [EC:4.2.1.120 5.3.3.3]
Butyrate Metabolism	KEGG | ko00650 Butanoate metabolism | K16871 4-aminobutyrate---pyruvate transaminase [EC:2.6.1.96]
Butyrate Metabolism	KEGG | ko00650 Butanoate metabolism | K18009 meso-butanediol dehydrogenase / (S,S)-butanediol dehydrogenase / diacetyl reductase [EC:1.1.1.- 1.1.1.76 1.1.1.304]
Butyrate Metabolism	KEGG | ko00650 Butanoate metabolism | K18119 succinate-semialdehyde dehydrogenase [EC:1.2.1.76]
Butyrate Metabolism	KEGG | ko00650 Butanoate metabolism | K18120 4-hydroxybutyrate dehydrogenase [EC:1.1.1.61]
Butyrate Metabolism	KEGG | ko00650 Butanoate metabolism | K18122 4-hydroxybutyrate CoA-transferase [EC:2.8.3.-]

METATRANSCRIPTOMICS	
**Metabolic Pathway**	**Functional Genes involved in Butyrate and Propionate metabolism based on KEGG database**
Glycolysis-associated Metabolism	KEGG | ko00010 Glycolysis / Gluconeogenesis | K00001 alcohol dehydrogenase [EC:1.1.1.1]
Glycolysis-associated Metabolism	KEGG | ko00010 Glycolysis / Gluconeogenesis | K00016 L-lactate dehydrogenase [EC:1.1.1.27]
Glycolysis-associated Metabolism	KEGG | ko00010 Glycolysis / Gluconeogenesis | K00121 S-(hydroxymethyl)glutathione dehydrogenase / alcohol dehydrogenase [EC:1.1.1.284 1.1.1.1]
Glycolysis-associated Metabolism	KEGG | ko00010 Glycolysis / Gluconeogenesis | K00128 aldehyde dehydrogenase (NAD+) [EC:1.2.1.3]
Glycolysis-associated Metabolism	KEGG | ko00010 Glycolysis / Gluconeogenesis | K00131 glyceraldehyde-3-phosphate dehydrogenase (NADP+) [EC:1.2.1.9]
Glycolysis-associated Metabolism	KEGG | ko00010 Glycolysis / Gluconeogenesis | K00134 glyceraldehyde 3-phosphate dehydrogenase [EC:1.2.1.12]
Glycolysis-associated Metabolism	KEGG | ko00010 Glycolysis / Gluconeogenesis | K00138 aldehyde dehydrogenase [EC:1.2.1.-]
Glycolysis-associated Metabolism	KEGG | ko00010 Glycolysis / Gluconeogenesis | K00161 pyruvate dehydrogenase E1 component alpha subunit [EC:1.2.4.1]
Glycolysis-associated Metabolism	KEGG | ko00010 Glycolysis / Gluconeogenesis | K00162 pyruvate dehydrogenase E1 component beta subunit [EC:1.2.4.1]
Glycolysis-associated Metabolism	KEGG | ko00010 Glycolysis / Gluconeogenesis | K00163 pyruvate dehydrogenase E1 component [EC:1.2.4.1]
Glycolysis-associated Metabolism	KEGG | ko00010 Glycolysis / Gluconeogenesis | K00169 pyruvate ferredoxin oxidoreductase alpha subunit [EC:1.2.7.1]
Glycolysis-associated Metabolism	KEGG | ko00010 Glycolysis / Gluconeogenesis | K00170 pyruvate ferredoxin oxidoreductase beta subunit [EC:1.2.7.1]
Glycolysis-associated Metabolism	KEGG | ko00010 Glycolysis / Gluconeogenesis | K00172 pyruvate ferredoxin oxidoreductase gamma subunit [EC:1.2.7.1]
Glycolysis-associated Metabolism	KEGG | ko00010 Glycolysis / Gluconeogenesis | K00174 2-oxoglutarate/2-oxoacid ferredoxin oxidoreductase subunit alpha [EC:1.2.7.3 1.2.7.11]
Glycolysis-associated Metabolism	KEGG | ko00010 Glycolysis / Gluconeogenesis | K00175 2-oxoglutarate/2-oxoacid ferredoxin oxidoreductase subunit beta [EC:1.2.7.3 1.2.7.11]
Glycolysis-associated Metabolism	KEGG | ko00010 Glycolysis / Gluconeogenesis | K00382 dihydrolipoamide dehydrogenase [EC:1.8.1.4]
Glycolysis-associated Metabolism	KEGG | ko00010 Glycolysis / Gluconeogenesis | K00627 pyruvate dehydrogenase E2 component (dihydrolipoamide acetyltransferase) [EC:2.3.1.12]
Glycolysis-associated Metabolism	KEGG | ko00010 Glycolysis / Gluconeogenesis | K00844 hexokinase [EC:2.7.1.1]
Glycolysis-associated Metabolism	KEGG | ko00010 Glycolysis / Gluconeogenesis | K00845 glucokinase [EC:2.7.1.2]
Glycolysis-associated Metabolism	KEGG | ko00010 Glycolysis / Gluconeogenesis | K00850 6-phosphofructokinase 1 [EC:2.7.1.11]
Glycolysis-associated Metabolism	KEGG | ko00010 Glycolysis / Gluconeogenesis | K00873 pyruvate kinase [EC:2.7.1.40]
Glycolysis-associated Metabolism	KEGG | ko00010 Glycolysis / Gluconeogenesis | K00886 polyphosphate glucokinase [EC:2.7.1.63]
Glycolysis-associated Metabolism	KEGG | ko00010 Glycolysis / Gluconeogenesis | K00895 diphosphate-dependent phosphofructokinase [EC:2.7.1.90]
Glycolysis-associated Metabolism	KEGG | ko00010 Glycolysis / Gluconeogenesis | K00927 phosphoglycerate kinase [EC:2.7.2.3]
Glycolysis-associated Metabolism	KEGG | ko00010 Glycolysis / Gluconeogenesis | K01085 glucose-1-phosphatase [EC:3.1.3.10]
Glycolysis-associated Metabolism	KEGG | ko00010 Glycolysis / Gluconeogenesis | K01222 6-phospho-beta-glucosidase [EC:3.2.1.86]
Glycolysis-associated Metabolism	KEGG | ko00010 Glycolysis / Gluconeogenesis | K01223 6-phospho-beta-glucosidase [EC:3.2.1.86]
Glycolysis-associated Metabolism	KEGG | ko00010 Glycolysis / Gluconeogenesis | K01568 pyruvate decarboxylase [EC:4.1.1.1]
Glycolysis-associated Metabolism	KEGG | ko00010 Glycolysis / Gluconeogenesis | K01596 phosphoenolpyruvate carboxykinase (GTP) [EC:4.1.1.32]
Glycolysis-associated Metabolism	KEGG | ko00010 Glycolysis / Gluconeogenesis | K01610 phosphoenolpyruvate carboxykinase (ATP) [EC:4.1.1.49]
Glycolysis-associated Metabolism	KEGG | ko00010 Glycolysis / Gluconeogenesis | K01623 fructose-bisphosphate aldolase, class I [EC:4.1.2.13]
Glycolysis-associated Metabolism	KEGG | ko00010 Glycolysis / Gluconeogenesis | K01624 fructose-bisphosphate aldolase, class II [EC:4.1.2.13]
Glycolysis-associated Metabolism	KEGG | ko00010 Glycolysis / Gluconeogenesis | K01689 enolase [EC:4.2.1.11]
Glycolysis-associated Metabolism	KEGG | ko00010 Glycolysis / Gluconeogenesis | K01785 aldose 1-epimerase [EC:5.1.3.3]
Glycolysis-associated Metabolism	KEGG | ko00010 Glycolysis / Gluconeogenesis | K01792 glucose-6-phosphate 1-epimerase [EC:5.1.3.15]
Glycolysis-associated Metabolism	KEGG | ko00010 Glycolysis / Gluconeogenesis | K01803 triosephosphate isomerase (TIM) [EC:5.3.1.1]
Glycolysis-associated Metabolism	KEGG | ko00010 Glycolysis / Gluconeogenesis | K01810 glucose-6-phosphate isomerase [EC:5.3.1.9]
Glycolysis-associated Metabolism	KEGG | ko00010 Glycolysis / Gluconeogenesis | K01834 2,3-bisphosphoglycerate-dependent phosphoglycerate mutase [EC:5.4.2.11]
Glycolysis-associated Metabolism	KEGG | ko00010 Glycolysis / Gluconeogenesis | K01835 phosphoglucomutase [EC:5.4.2.2]
Glycolysis-associated Metabolism	KEGG | ko00010 Glycolysis / Gluconeogenesis | K01895 acetyl-CoA synthetase [EC:6.2.1.1]
Glycolysis-associated Metabolism	KEGG | ko00010 Glycolysis / Gluconeogenesis | K01905 acetate---CoA ligase (ADP-forming) subunit alpha [EC:6.2.1.13]
Glycolysis-associated Metabolism	KEGG | ko00010 Glycolysis / Gluconeogenesis | K02446 fructose-1,6-bisphosphatase II [EC:3.1.3.11]
Glycolysis-associated Metabolism	KEGG | ko00010 Glycolysis / Gluconeogenesis | K02753 PTS system, beta-glucoside (arbutin/salicin/cellobiose)-specific IIC component
Glycolysis-associated Metabolism	KEGG | ko00010 Glycolysis / Gluconeogenesis | K02777 PTS system, sugar-specific IIA component [EC:2.7.1.-]
Glycolysis-associated Metabolism	KEGG | ko00010 Glycolysis / Gluconeogenesis | K02779 PTS system, glucose-specific IIC component
Glycolysis-associated Metabolism	KEGG | ko00010 Glycolysis / Gluconeogenesis | K02791 PTS system, maltose/glucose-specific IIC component
Glycolysis-associated Metabolism	KEGG | ko00010 Glycolysis / Gluconeogenesis | K03737 pyruvate-ferredoxin/flavodoxin oxidoreductase [EC:1.2.7.1 1.2.7.-]
Glycolysis-associated Metabolism	KEGG | ko00010 Glycolysis / Gluconeogenesis | K03841 fructose-1,6-bisphosphatase I [EC:3.1.3.11]
Glycolysis-associated Metabolism	KEGG | ko00010 Glycolysis / Gluconeogenesis | K04022 alcohol dehydrogenase
Glycolysis-associated Metabolism	KEGG | ko00010 Glycolysis / Gluconeogenesis | K04041 fructose-1,6-bisphosphatase III [EC:3.1.3.11]
Glycolysis-associated Metabolism	KEGG | ko00010 Glycolysis / Gluconeogenesis | K04072 acetaldehyde dehydrogenase / alcohol dehydrogenase [EC:1.2.1.10 1.1.1.1]
Glycolysis-associated Metabolism	KEGG | ko00010 Glycolysis / Gluconeogenesis | K11645 fructose-bisphosphate aldolase, class I [EC:4.1.2.13]
Glycolysis-associated Metabolism	KEGG | ko00010 Glycolysis / Gluconeogenesis | K13953 alcohol dehydrogenase, propanol-preferring [EC:1.1.1.1]
Glycolysis-associated Metabolism	KEGG | ko00010 Glycolysis / Gluconeogenesis | K13954 alcohol dehydrogenase [EC:1.1.1.1]
Glycolysis-associated Metabolism	KEGG | ko00010 Glycolysis / Gluconeogenesis | K15633 2,3-bisphosphoglycerate-independent phosphoglycerate mutase [EC:5.4.2.12]
Glycolysis-associated Metabolism	KEGG | ko00010 Glycolysis / Gluconeogenesis | K15634 probable phosphoglycerate mutase [EC:5.4.2.12]
Glycolysis-associated Metabolism	KEGG | ko00010 Glycolysis / Gluconeogenesis | K15635 2,3-bisphosphoglycerate-independent phosphoglycerate mutase [EC:5.4.2.12]
Glycolysis-associated Metabolism	KEGG | ko00010 Glycolysis / Gluconeogenesis | K15778 phosphomannomutase / phosphoglucomutase [EC:5.4.2.8 5.4.2.2]
Glycolysis-associated Metabolism	KEGG | ko00010 Glycolysis / Gluconeogenesis | K16370 6-phosphofructokinase 2 [EC:2.7.1.11]
Glycolysis-associated Metabolism	KEGG | ko00020 Citrate cycle (TCA cycle) | K00024 malate dehydrogenase [EC:1.1.1.37]
Glycolysis-associated Metabolism	KEGG | ko00020 Citrate cycle (TCA cycle) | K00030 isocitrate dehydrogenase (NAD+) [EC:1.1.1.41]
Glycolysis-associated Metabolism	KEGG | ko00020 Citrate cycle (TCA cycle) | K00031 isocitrate dehydrogenase [EC:1.1.1.42]
Glycolysis-associated Metabolism	KEGG | ko00020 Citrate cycle (TCA cycle) | K00116 malate dehydrogenase (quinone) [EC:1.1.5.4]
Glycolysis-associated Metabolism	KEGG | ko00020 Citrate cycle (TCA cycle) | K00164 2-oxoglutarate dehydrogenase E1 component [EC:1.2.4.2]
Glycolysis-associated Metabolism	KEGG | ko00020 Citrate cycle (TCA cycle) | K00176 2-oxoglutarate ferredoxin oxidoreductase subunit delta [EC:1.2.7.3]
Glycolysis-associated Metabolism	KEGG | ko00020 Citrate cycle (TCA cycle) | K00177 2-oxoglutarate ferredoxin oxidoreductase subunit gamma [EC:1.2.7.3]
Glycolysis-associated Metabolism	KEGG | ko00020 Citrate cycle (TCA cycle) | K00239 succinate dehydrogenase / fumarate reductase, flavoprotein subunit [EC:1.3.5.1 1.3.5.4]
Glycolysis-associated Metabolism	KEGG | ko00020 Citrate cycle (TCA cycle) | K00240 succinate dehydrogenase / fumarate reductase, iron-sulfur subunit [EC:1.3.5.1 1.3.5.4]
Glycolysis-associated Metabolism	KEGG | ko00020 Citrate cycle (TCA cycle) | K00241 succinate dehydrogenase / fumarate reductase, cytochrome b subunit
Glycolysis-associated Metabolism	KEGG | ko00020 Citrate cycle (TCA cycle) | K00244 fumarate reductase flavoprotein subunit [EC:1.3.5.4]
Glycolysis-associated Metabolism	KEGG | ko00020 Citrate cycle (TCA cycle) | K00245 fumarate reductase iron-sulfur subunit [EC:1.3.5.4]
Glycolysis-associated Metabolism	KEGG | ko00020 Citrate cycle (TCA cycle) | K00246 fumarate reductase subunit C
Glycolysis-associated Metabolism	KEGG | ko00020 Citrate cycle (TCA cycle) | K00247 fumarate reductase subunit D
Glycolysis-associated Metabolism	KEGG | ko00020 Citrate cycle (TCA cycle) | K00658 2-oxoglutarate dehydrogenase E2 component (dihydrolipoamide succinyltransferase) [EC:2.3.1.61]
Glycolysis-associated Metabolism	KEGG | ko00020 Citrate cycle (TCA cycle) | K01647 citrate synthase [EC:2.3.3.1]
Glycolysis-associated Metabolism	KEGG | ko00020 Citrate cycle (TCA cycle) | K01676 fumarate hydratase, class I [EC:4.2.1.2]
Glycolysis-associated Metabolism	KEGG | ko00020 Citrate cycle (TCA cycle) | K01677 fumarate hydratase subunit alpha [EC:4.2.1.2]
Glycolysis-associated Metabolism	KEGG | ko00020 Citrate cycle (TCA cycle) | K01678 fumarate hydratase subunit beta [EC:4.2.1.2]
Glycolysis-associated Metabolism	KEGG | ko00020 Citrate cycle (TCA cycle) | K01679 fumarate hydratase, class II [EC:4.2.1.2]
Glycolysis-associated Metabolism	KEGG | ko00020 Citrate cycle (TCA cycle) | K01681 aconitate hydratase [EC:4.2.1.3]
Glycolysis-associated Metabolism	KEGG | ko00020 Citrate cycle (TCA cycle) | K01682 aconitate hydratase 2 / 2-methylisocitrate dehydratase [EC:4.2.1.3 4.2.1.99]
Glycolysis-associated Metabolism	KEGG | ko00020 Citrate cycle (TCA cycle) | K01900 succinyl-CoA synthetase beta subunit [EC:6.2.1.4 6.2.1.5]
Glycolysis-associated Metabolism	KEGG | ko00020 Citrate cycle (TCA cycle) | K01902 succinyl-CoA synthetase alpha subunit [EC:6.2.1.5]
Glycolysis-associated Metabolism	KEGG | ko00020 Citrate cycle (TCA cycle) | K01903 succinyl-CoA synthetase beta subunit [EC:6.2.1.5]
Glycolysis-associated Metabolism	KEGG | ko00020 Citrate cycle (TCA cycle) | K01958 pyruvate carboxylase [EC:6.4.1.1]
Glycolysis-associated Metabolism	KEGG | ko00020 Citrate cycle (TCA cycle) | K01960 pyruvate carboxylase subunit B [EC:6.4.1.1]
Glycolysis-associated Metabolism	KEGG | ko00020 Citrate cycle (TCA cycle) | K18118 succinyl-CoA:acetate CoA-transferase [EC:2.8.3.18]
Glycolysis-associated Metabolism	KEGG | ko00030 Pentose phosphate pathway | K00033 6-phosphogluconate dehydrogenase [EC:1.1.1.44 1.1.1.343]
Glycolysis-associated Metabolism	KEGG | ko00030 Pentose phosphate pathway | K00034 glucose 1-dehydrogenase [EC:1.1.1.47]
Glycolysis-associated Metabolism	KEGG | ko00030 Pentose phosphate pathway | K00036 glucose-6-phosphate 1-dehydrogenase [EC:1.1.1.49 1.1.1.363]
Glycolysis-associated Metabolism	KEGG | ko00030 Pentose phosphate pathway | K00090 glyoxylate/hydroxypyruvate/2-ketogluconate reductase [EC:1.1.1.79 1.1.1.81 1.1.1.215]
Glycolysis-associated Metabolism	KEGG | ko00030 Pentose phosphate pathway | K00117 quinoprotein glucose dehydrogenase [EC:1.1.5.2]
Glycolysis-associated Metabolism	KEGG | ko00030 Pentose phosphate pathway | K00615 transketolase [EC:2.2.1.1]
Glycolysis-associated Metabolism	KEGG | ko00030 Pentose phosphate pathway | K00616 transaldolase [EC:2.2.1.2]
Glycolysis-associated Metabolism	KEGG | ko00030 Pentose phosphate pathway | K00851 gluconokinase [EC:2.7.1.12]
Glycolysis-associated Metabolism	KEGG | ko00030 Pentose phosphate pathway | K00852 ribokinase [EC:2.7.1.15]
Glycolysis-associated Metabolism	KEGG | ko00030 Pentose phosphate pathway | K00874 2-dehydro-3-deoxygluconokinase [EC:2.7.1.45]
Glycolysis-associated Metabolism	KEGG | ko00030 Pentose phosphate pathway | K00948 ribose-phosphate pyrophosphokinase [EC:2.7.6.1]
Glycolysis-associated Metabolism	KEGG | ko00030 Pentose phosphate pathway | K01057 6-phosphogluconolactonase [EC:3.1.1.31]
Glycolysis-associated Metabolism	KEGG | ko00030 Pentose phosphate pathway | K01619 deoxyribose-phosphate aldolase [EC:4.1.2.4]

Glycolysis-associated Metabolism	KEGG | ko00030 Pentose phosphate pathway | K01621 xylulose-5-phosphate/fructose-6-phosphate phosphoketolase [EC:4.1.2.9 4.1.2.22]
Glycolysis-associated Metabolism	KEGG | ko00030 Pentose phosphate pathway | K01625 2-dehydro-3-deoxyphosphogluconate aldolase / (4S)-4-hydroxy-2-oxoglutarate aldolase [EC:4.1.2.14 4.1.3.42]
Glycolysis-associated Metabolism	KEGG | ko00030 Pentose phosphate pathway | K01783 ribulose-phosphate 3-epimerase [EC:5.1.3.1]
Glycolysis-associated Metabolism	KEGG | ko00030 Pentose phosphate pathway | K01807 ribose 5-phosphate isomerase A [EC:5.3.1.6]
Glycolysis-associated Metabolism	KEGG | ko00030 Pentose phosphate pathway | K01808 ribose 5-phosphate isomerase B [EC:5.3.1.6]
Glycolysis-associated Metabolism	KEGG | ko00030 Pentose phosphate pathway | K01839 phosphopentomutase [EC:5.4.2.7]
Glycolysis-associated Metabolism	KEGG | ko00030 Pentose phosphate pathway | K03738 aldehyde:ferredoxin oxidoreductase [EC:1.2.7.5]
Glycolysis-associated Metabolism	KEGG | ko00030 Pentose phosphate pathway | K05774 ribose 1,5-bisphosphokinase [EC:2.7.4.23]
Glycolysis-associated Metabolism	KEGG | ko00030 Pentose phosphate pathway | K06151 gluconate 2-dehydrogenase alpha chain [EC:1.1.99.3]
Glycolysis-associated Metabolism	KEGG | ko00030 Pentose phosphate pathway | K06152 gluconate 2-dehydrogenase gamma chain [EC:1.1.99.3]
Glycolysis-associated Metabolism	KEGG | ko00030 Pentose phosphate pathway | K07404 6-phosphogluconolactonase [EC:3.1.1.31]
Glycolysis-associated Metabolism	KEGG | ko00030 Pentose phosphate pathway | K08093 3-hexulose-6-phosphate synthase [EC:4.1.2.43]
Glycolysis-associated Metabolism	KEGG | ko00030 Pentose phosphate pathway | K08094 6-phospho-3-hexuloisomerase [EC:5.3.1.27]
Glycolysis-associated Metabolism	KEGG | ko00030 Pentose phosphate pathway | K17465 PTS system, D-glucosaminate-specific IIB component [EC:2.7.1.203]
Glycolysis-associated Metabolism	KEGG | ko00030 Pentose phosphate pathway | K17466 PTS system, D-glucosaminate-specific IIC component
Glycolysis-associated Metabolism	KEGG | ko00030 Pentose phosphate pathway | K17467 PTS system, D-glucosaminate-specific IID component
Glycolysis-associated Metabolism	KEGG | ko00500 Starch and sucrose metabolism | K00688 glycogen phosphorylase [EC:2.4.1.1]
Glycolysis-associated Metabolism	KEGG | ko00500 Starch and sucrose metabolism | K00690 sucrose phosphorylase [EC:2.4.1.7]
Glycolysis-associated Metabolism	KEGG | ko00500 Starch and sucrose metabolism | K00691 maltose phosphorylase [EC:2.4.1.8]
Glycolysis-associated Metabolism	KEGG | ko00500 Starch and sucrose metabolism | K00692 levansucrase [EC:2.4.1.10]
Glycolysis-associated Metabolism	KEGG | ko00500 Starch and sucrose metabolism | K00694 cellulose synthase (UDP-forming) [EC:2.4.1.12]
Glycolysis-associated Metabolism	KEGG | ko00500 Starch and sucrose metabolism | K00697 trehalose 6-phosphate synthase [EC:2.4.1.15]
Glycolysis-associated Metabolism	KEGG | ko00500 Starch and sucrose metabolism | K00700 1,4-alpha-glucan branching enzyme [EC:2.4.1.18]
Glycolysis-associated Metabolism	KEGG | ko00500 Starch and sucrose metabolism | K00702 cellobiose phosphorylase [EC:2.4.1.20]
Glycolysis-associated Metabolism	KEGG | ko00500 Starch and sucrose metabolism | K00703 starch synthase [EC:2.4.1.21]
Glycolysis-associated Metabolism	KEGG | ko00500 Starch and sucrose metabolism | K00705 4-alpha-glucanotransferase [EC:2.4.1.25]
Glycolysis-associated Metabolism	KEGG | ko00500 Starch and sucrose metabolism | K00975 glucose-1-phosphate adenylyltransferase [EC:2.7.7.27]
Glycolysis-associated Metabolism	KEGG | ko00500 Starch and sucrose metabolism | K00978 glucose-1-phosphate cytidylyltransferase [EC:2.7.7.33]
Glycolysis-associated Metabolism	KEGG | ko00500 Starch and sucrose metabolism | K01087 trehalose 6-phosphate phosphatase [EC:3.1.3.12]
Glycolysis-associated Metabolism	KEGG | ko00500 Starch and sucrose metabolism | K01176 alpha-amylase [EC:3.2.1.1]
Glycolysis-associated Metabolism	KEGG | ko00500 Starch and sucrose metabolism | K01179 endoglucanase [EC:3.2.1.4]
Glycolysis-associated Metabolism	KEGG | ko00500 Starch and sucrose metabolism | K01194 alpha,alpha-trehalase [EC:3.2.1.28]
Glycolysis-associated Metabolism	KEGG | ko00500 Starch and sucrose metabolism | K01210 glucan 1,3-beta-glucosidase [EC:3.2.1.58]
Glycolysis-associated Metabolism	KEGG | ko00500 Starch and sucrose metabolism | K01226 trehalose-6-phosphate hydrolase [EC:3.2.1.93]
Glycolysis-associated Metabolism	KEGG | ko00500 Starch and sucrose metabolism | K01232 maltose-6'-phosphate glucosidase [EC:3.2.1.122]
Glycolysis-associated Metabolism	KEGG | ko00500 Starch and sucrose metabolism | K01236 maltooligosyltrehalose trehalohydrolase [EC:3.2.1.141]
Glycolysis-associated Metabolism	KEGG | ko00500 Starch and sucrose metabolism | K02438 glycogen debranching enzyme [EC:3.2.1.196]
Glycolysis-associated Metabolism	KEGG | ko00500 Starch and sucrose metabolism | K02750 PTS system, alpha-glucoside-specific IIC component
Glycolysis-associated Metabolism	KEGG | ko00500 Starch and sucrose metabolism | K02759 PTS system, cellobiose-specific IIA component [EC:2.7.1.196 2.7.1.205]
Glycolysis-associated Metabolism	KEGG | ko00500 Starch and sucrose metabolism | K02760 PTS system, cellobiose-specific IIB component [EC:2.7.1.196 2.7.1.205]
Glycolysis-associated Metabolism	KEGG | ko00500 Starch and sucrose metabolism | K02761 PTS system, cellobiose-specific IIC component
Glycolysis-associated Metabolism	KEGG | ko00500 Starch and sucrose metabolism | K02810 PTS system, sucrose-specific IIC component
Glycolysis-associated Metabolism	KEGG | ko00500 Starch and sucrose metabolism | K02819 PTS system, trehalose-specific IIC component
Glycolysis-associated Metabolism	KEGG | ko00500 Starch and sucrose metabolism | K05341 amylosucrase [EC:2.4.1.4]
Glycolysis-associated Metabolism	KEGG | ko00500 Starch and sucrose metabolism | K05342 alpha,alpha-trehalose phosphorylase [EC:2.4.1.64]
Glycolysis-associated Metabolism	KEGG | ko00500 Starch and sucrose metabolism | K05343 maltose alpha-D-glucosyltransferase / alpha-amylase [EC:5.4.99.16 3.2.1.1]
Glycolysis-associated Metabolism	KEGG | ko00500 Starch and sucrose metabolism | K05349 beta-glucosidase [EC:3.2.1.21]
Glycolysis-associated Metabolism	KEGG | ko00500 Starch and sucrose metabolism | K05350 beta-glucosidase [EC:3.2.1.21]
Glycolysis-associated Metabolism	KEGG | ko00500 Starch and sucrose metabolism | K06896 maltose 6'-phosphate phosphatase [EC:3.1.3.90]
Glycolysis-associated Metabolism	KEGG | ko00500 Starch and sucrose metabolism | K07405 alpha-amylase [EC:3.2.1.1]
Glycolysis-associated Metabolism	KEGG | ko00500 Starch and sucrose metabolism | K16055 trehalose 6-phosphate synthase/phosphatase [EC:2.4.1.15 3.1.3.12]
Glycolysis-associated Metabolism	KEGG | ko00500 Starch and sucrose metabolism | K16147 starch synthase (maltosyl-transferring) [EC:2.4.99.16]
Glycolysis-associated Metabolism	KEGG | ko00500 Starch and sucrose metabolism | K16148 alpha-maltose-1-phosphate synthase [EC:2.4.1.342]
Glycolysis-associated Metabolism	KEGG | ko00500 Starch and sucrose metabolism | K18775 levanbiose-producing levanase [EC:3.2.1.64]
Glycolysis-associated Metabolism	KEGG | ko00620 Pyruvate metabolism | K00027 malate dehydrogenase (oxaloacetate-decarboxylating) [EC:1.1.1.38]
Glycolysis-associated Metabolism	KEGG | ko00620 Pyruvate metabolism | K00029 malate dehydrogenase (oxaloacetate-decarboxylating)(NADP+) [EC:1.1.1.40]
Glycolysis-associated Metabolism	KEGG | ko00620 Pyruvate metabolism | K00101 L-lactate dehydrogenase (cytochrome) [EC:1.1.2.3]
Glycolysis-associated Metabolism	KEGG | ko00620 Pyruvate metabolism | K00132 acetaldehyde dehydrogenase (acetylating) [EC:1.2.1.10]
Glycolysis-associated Metabolism	KEGG | ko00620 Pyruvate metabolism | K00156 pyruvate dehydrogenase (quinone) [EC:1.2.5.1]
Glycolysis-associated Metabolism	KEGG | ko00620 Pyruvate metabolism | K00158 pyruvate oxidase [EC:1.2.3.3]
Glycolysis-associated Metabolism	KEGG | ko00620 Pyruvate metabolism | K00625 phosphate acetyltransferase [EC:2.3.1.8]
Glycolysis-associated Metabolism	KEGG | ko00620 Pyruvate metabolism | K00626 acetyl-CoA C-acetyltransferase [EC:2.3.1.9]
Glycolysis-associated Metabolism	KEGG | ko00620 Pyruvate metabolism | K00656 formate C-acetyltransferase [EC:2.3.1.54]
Glycolysis-associated Metabolism	KEGG | ko00620 Pyruvate metabolism | K00925 acetate kinase [EC:2.7.2.1]
Glycolysis-associated Metabolism	KEGG | ko00620 Pyruvate metabolism | K01006 pyruvate, orthophosphate dikinase [EC:2.7.9.1]
Glycolysis-associated Metabolism	KEGG | ko00620 Pyruvate metabolism | K01007 pyruvate, water dikinase [EC:2.7.9.2]
Glycolysis-associated Metabolism	KEGG | ko00620 Pyruvate metabolism | K01026 propionate CoA-transferase [EC:2.8.3.1]
Glycolysis-associated Metabolism	KEGG | ko00620 Pyruvate metabolism | K01069 hydroxyacylglutathione hydrolase [EC:3.1.2.6]
Glycolysis-associated Metabolism	KEGG | ko00620 Pyruvate metabolism | K01512 acylphosphatase [EC:3.6.1.7]
Glycolysis-associated Metabolism	KEGG | ko00620 Pyruvate metabolism | K01571 oxaloacetate decarboxylase, alpha subunit [EC:4.1.1.3]
Glycolysis-associated Metabolism	KEGG | ko00620 Pyruvate metabolism | K01572 oxaloacetate decarboxylase, beta subunit [EC:4.1.1.3]
Glycolysis-associated Metabolism	KEGG | ko00620 Pyruvate metabolism | K01573 oxaloacetate decarboxylase, gamma subunit [EC:4.1.1.3]
Glycolysis-associated Metabolism	KEGG | ko00620 Pyruvate metabolism | K01595 phosphoenolpyruvate carboxylase [EC:4.1.1.31]
Glycolysis-associated Metabolism	KEGG | ko00620 Pyruvate metabolism | K01638 malate synthase [EC:2.3.3.9]
Glycolysis-associated Metabolism	KEGG | ko00620 Pyruvate metabolism | K01649 2-isopropylmalate synthase [EC:2.3.3.13]
Glycolysis-associated Metabolism	KEGG | ko00620 Pyruvate metabolism | K01759 lactoylglutathione lyase [EC:4.4.1.5]
Glycolysis-associated Metabolism	KEGG | ko00620 Pyruvate metabolism | K01961 acetyl-CoA carboxylase, biotin carboxylase subunit [EC:6.4.1.2 6.3.4.14]
Glycolysis-associated Metabolism	KEGG | ko00620 Pyruvate metabolism | K01962 acetyl-CoA carboxylase carboxyl transferase subunit alpha [EC:6.4.1.2]
Glycolysis-associated Metabolism	KEGG | ko00620 Pyruvate metabolism | K01963 acetyl-CoA carboxylase carboxyl transferase subunit beta [EC:6.4.1.2]
Glycolysis-associated Metabolism	KEGG | ko00620 Pyruvate metabolism | K02160 acetyl-CoA carboxylase biotin carboxyl carrier protein
Glycolysis-associated Metabolism	KEGG | ko00620 Pyruvate metabolism | K02594 homocitrate synthase NifV [EC:2.3.3.14]
Glycolysis-associated Metabolism	KEGG | ko00620 Pyruvate metabolism | K03777 D-lactate dehydrogenase (quinone) [EC:1.1.5.12]
Glycolysis-associated Metabolism	KEGG | ko00620 Pyruvate metabolism | K03778 D-lactate dehydrogenase [EC:1.1.1.28]
Glycolysis-associated Metabolism	KEGG | ko00620 Pyruvate metabolism | K04020 phosphotransacetylase
Glycolysis-associated Metabolism	KEGG | ko00620 Pyruvate metabolism | K04021 aldehyde dehydrogenase
Glycolysis-associated Metabolism	KEGG | ko00620 Pyruvate metabolism | K05523 D-lactate dehydratase / protein deglycase [EC:4.2.1.130 3.5.1.124]
Glycolysis-associated Metabolism	KEGG | ko00620 Pyruvate metabolism | K07248 lactaldehyde dehydrogenase / glycolaldehyde dehydrogenase [EC:1.2.1.22 1.2.1.21]
Glycolysis-associated Metabolism	KEGG | ko00620 Pyruvate metabolism | K11263 acetyl-CoA/propionyl-CoA carboxylase, biotin carboxylase, biotin carboxyl carrier protein [EC:6.4.1.2 6.4.1.3 6.3.4.14]
Glycolysis-associated Metabolism	KEGG | ko00620 Pyruvate metabolism | K12972 glyoxylate/hydroxypyruvatereductase [EC:1.1.1.79 1.1.1.81]
Glycolysis-associated Metabolism	KEGG | ko00620 Pyruvate metabolism | K13788 phosphate acetyltransferase [EC:2.3.1.8]
Glycolysis-associated Metabolism	KEGG | ko00620 Pyruvate metabolism | K15024 putative phosphotransacetylase [EC:2.3.1.8]
Propionate Metabolism	KEGG | ko00640 Propanoate metabolism | K00005 glycerol dehydrogenase [EC:1.1.1.6]
Propionate Metabolism	KEGG | ko00640 Propanoate metabolism | K00086 1,3-propanediol dehydrogenase [EC:1.1.1.202]
Propionate Metabolism	KEGG | ko00640 Propanoate metabolism | K00140 malonate-semialdehyde dehydrogenase (acetylating) / methylmalonate-semialdehyde dehydrogenase [EC:1.2.1.18 1.2.1.27]
Propionate Metabolism	KEGG | ko00640 Propanoate metabolism | K00166 2-oxoisovalerate dehydrogenase E1 component alpha subunit [EC:1.2.4.4]
Propionate Metabolism	KEGG | ko00640 Propanoate metabolism | K00167 2-oxoisovalerate dehydrogenase E1 component beta subunit [EC:1.2.4.4]
Propionate Metabolism	KEGG | ko00640 Propanoate metabolism | K00823 4-aminobutyrate aminotransferase [EC:2.6.1.19]
Propionate Metabolism	KEGG | ko00640 Propanoate metabolism | K00932 propionate kinase [EC:2.7.2.15]
Propionate Metabolism	KEGG | ko00640 Propanoate metabolism | K01034 acetate CoA/acetoacetate CoA-transferase alpha subunit [EC:2.8.3.8 2.8.3.9]
Propionate Metabolism	KEGG | ko00640 Propanoate metabolism | K01035 acetate CoA/acetoacetate CoA-transferase beta subunit [EC:2.8.3.8 2.8.3.9]
Propionate Metabolism	KEGG | ko00640 Propanoate metabolism | K01574 acetoacetate decarboxylase [EC:4.1.1.4]
Propionate Metabolism	KEGG | ko00640 Propanoate metabolism | K01699 propanediol dehydratase large subunit [EC:4.2.1.28]
Propionate Metabolism	KEGG | ko00640 Propanoate metabolism | K01734 methylglyoxal synthase [EC:4.2.3.3]
Propionate Metabolism	KEGG | ko00640 Propanoate metabolism | K01782 3-hydroxyacyl-CoA dehydrogenase / enoyl-CoA hydratase / 3-hydroxybutyryl-CoA epimerase [EC:1.1.1.35 4.2.1.17 5.1.2.3]
Propionate Metabolism	KEGG | ko00640 Propanoate metabolism | K01825 3-hydroxyacyl-CoA dehydrogenase / enoyl-CoA hydratase / 3-hydroxybutyryl-CoA epimerase / enoyl-CoA isomerase [EC:1.1.1.35 4.2.1.17 5.1.2.3 5.3.3.8]

Propionate Metabolism	KEGG | ko00640 Propanoate metabolism | K03416 methylmalonyl-CoA carboxyltransferase 5S subunit [EC:2.1.3.1]
Propionate Metabolism	KEGG | ko00640 Propanoate metabolism | K07250 4-aminobutyrate aminotransferase / (S)-3-amino-2-methylpropionate transaminase [EC:2.6.1.19 2.6.1.22]
Propionate Metabolism	KEGG | ko00640 Propanoate metabolism | K08325 NADP-dependent alcohol dehydrogenase [EC:1.1.-.-]
Propionate Metabolism	KEGG | ko00640 Propanoate metabolism | K09699 2-oxoisovalerate dehydrogenase E2 component (dihydrolipoyl transacylase) [EC:2.3.1.168]
Propionate Metabolism	KEGG | ko00640 Propanoate metabolism | K11381 2-oxoisovalerate dehydrogenase E1 component [EC:1.2.4.4]
Propionate Metabolism	KEGG | ko00640 Propanoate metabolism | K13919 propanediol dehydratase medium subunit [EC:4.2.1.28]
Propionate Metabolism	KEGG | ko00640 Propanoate metabolism | K13920 propanediol dehydratase small subunit [EC:4.2.1.28]
Propionate Metabolism	KEGG | ko00640 Propanoate metabolism | K13921 1-propanol dehydrogenase
Propionate Metabolism	KEGG | ko00640 Propanoate metabolism | K13922 propionaldehyde dehydrogenase [EC:1.2.1.87]
Propionate Metabolism	KEGG | ko00640 Propanoate metabolism | K13923 phosphate propanoyltransferase [EC:2.3.1.222]
Propionate Metabolism	KEGG | ko00640 Propanoate metabolism | K17489 methylmalonyl-CoA carboxyltransferase 12S subunit [EC:2.1.3.1]
Propionate Metabolism	KEGG | ko00640 Propanoate metabolism | K18369 alcohol dehydrogenase [EC:1.1.1.-]
Propionate Metabolism	KEGG | ko00640 Propanoate metabolism | K18471 methylglyoxal reductase [EC:1.1.1.-]
Propionate Metabolism	KEGG | ko00640 Propanoate metabolism | K19697 propionate kinase [EC:2.7.2.15]
Propionate Metabolism	KEGG | ko00640 Propanoate metabolism | K19709 acetate CoA-transferase [EC:2.8.3.8]
Propionate Metabolism	KEGG | ko00640 Propanoate metabolism | K19745 acrylyl-CoA reductase (NADPH) [EC:1.3.1.-]
Butyrate Metabolism	KEGG | ko00650 Butanoate metabolism | K00004 (R,R)-butanediol dehydrogenase / meso-butanediol dehydrogenase / diacetyl reductase [EC:1.1.1.4 1.1.1.- 1.1.1.303]
Butyrate Metabolism	KEGG | ko00650 Butanoate metabolism | K00019 3-hydroxybutyrate dehydrogenase [EC:1.1.1.30]
Butyrate Metabolism	KEGG | ko00650 Butanoate metabolism | K00074 3-hydroxybutyryl-CoA dehydrogenase [EC:1.1.1.157]
Butyrate Metabolism	KEGG | ko00650 Butanoate metabolism | K00100 butanol dehydrogenase [EC:1.1.1.-]
Butyrate Metabolism	KEGG | ko00650 Butanoate metabolism | K00135 succinate-semialdehyde dehydrogenase / glutarate-semialdehyde dehydrogenase [EC:1.2.1.16 1.2.1.79 1.2.1.20]
Butyrate Metabolism	KEGG | ko00650 Butanoate metabolism | K00209 enoyl-[acyl-carrier protein] reductase / trans-2-enoyl-CoA reductase (NAD+) [EC:1.3.1.9 1.3.1.44]
Butyrate Metabolism	KEGG | ko00650 Butanoate metabolism | K00248 butyryl-CoA dehydrogenase [EC:1.3.8.1]
Butyrate Metabolism	KEGG | ko00650 Butanoate metabolism | K00634 phosphate butyryltransferase [EC:2.3.1.19]
Butyrate Metabolism	KEGG | ko00650 Butanoate metabolism | K00929 butyrate kinase [EC:2.7.2.7]
Butyrate Metabolism	KEGG | ko00650 Butanoate metabolism | K01029 3-oxoacid CoA-transferase subunit B [EC:2.8.3.5]
Butyrate Metabolism	KEGG | ko00650 Butanoate metabolism | K01039 glutaconate CoA-transferase, subunit A [EC:2.8.3.12]
Butyrate Metabolism	KEGG | ko00650 Butanoate metabolism | K01040 glutaconate CoA-transferase, subunit B [EC:2.8.3.12]
Butyrate Metabolism	KEGG | ko00650 Butanoate metabolism | K01575 acetolactate decarboxylase [EC:4.1.1.5]
Butyrate Metabolism	KEGG | ko00650 Butanoate metabolism | K01580 glutamate decarboxylase [EC:4.1.1.15]
Butyrate Metabolism	KEGG | ko00650 Butanoate metabolism | K01615 glutaconyl-CoA decarboxylase [EC:4.1.1.70]
Butyrate Metabolism	KEGG | ko00650 Butanoate metabolism | K01641 hydroxymethylglutaryl-CoA synthase [EC:2.3.3.10]
Butyrate Metabolism	KEGG | ko00650 Butanoate metabolism | K01652 acetolactate synthase I/II/III large subunit [EC:2.2.1.6]
Butyrate Metabolism	KEGG | ko00650 Butanoate metabolism | K01653 acetolactate synthase I/III small subunit [EC:2.2.1.6]
Butyrate Metabolism	KEGG | ko00650 Butanoate metabolism | K01715 enoyl-CoA hydratase [EC:4.2.1.17]
Butyrate Metabolism	KEGG | ko00650 Butanoate metabolism | K03366 meso-butanediol dehydrogenase / (S,S)-butanediol dehydrogenase / diacetyl reductase [EC:1.1.1.- 1.1.1.76 1.1.1.304]
Butyrate Metabolism	KEGG | ko00650 Butanoate metabolism | K08318 4-hydroxybutyrate dehydrogenase / sulfolactaldehyde 3-reductase [EC:1.1.1.61 1.1.1.373]
Butyrate Metabolism	KEGG | ko00650 Butanoate metabolism | K08324 succinate-semialdehyde dehydrogenase [EC:1.2.1.16 1.2.1.24]
Butyrate Metabolism	KEGG | ko00650 Butanoate metabolism | K14534 4-hydroxybutyryl-CoA dehydratase / vinylacetyl-CoA-Delta-isomerase [EC:4.2.1.120 5.3.3.3]
Butyrate Metabolism	KEGG | ko00650 Butanoate metabolism | K18120 4-hydroxybutyrate dehydrogenase [EC:1.1.1.61]
Butyrate Metabolism	KEGG | ko00650 Butanoate metabolism | K18122 4-hydroxybutyrate CoA-transferase [EC:2.8.3.-]

Supplementary Table 4a: Carbohydrate functional gene expression patterns derived from 13 bacterial species at metagenomics level.

		3 Weeks																
		Control (n=13)			Non-Allergen Sensitized Atopic Eczema (NAE) (n=5)							Allergen Sensitized Atopic Eczema (AE) (n=5)						
		Geometric Mean (Reads)	Upper Range of Geometric SD	Lower Range of Geometric SD	Geometric Mean (Reads)	Upper Range of Geometric SD	Lower Range of Geometric SD	B Estimate$^{\psi}$	95% CI Lower Bound$^{\psi}$	95% CI Upper Bound$^{\psi}$	Adj p value$^{\psi}$	Geometric Mean (Reads)	Upper Range of Geometric SD	Lower Range of Geometric SD	B Estimate$^{\psi}$	95% CI Lower Bound$^{\psi}$	95% CI Upper Bound$^{\psi}$	Adj p value$^{\psi}$
**METAGENOMICS**	Glycolysis-associated Metabolism	100.63	165.03	61.36	92.23	178.93	47.54	-121.03	-1,098.29	856.24	0.427	7.69	12.15	4.87	-214.51	-1,080.70	651.68	0.048*
	Butyrate Metabolism	11.24	21.24	5.95	25.79	31.98	20.80	32.67	-447.71	513.05	0.886	0.81	1.28	0.51	-52.72	-471.79	366.35	0.036*
	Propionate Metabolism	83.33	149.16	46.55	58.45	106.96	31.94	-184.96	-323.78	-46.14	0.221	17.74	25.54	12.32	-193.34	-468.04	81.37	0.031*

		3 Months																
		Control (n=16)			Non-Allergen Sensitized Atopic Eczema (NAE) (n=11)							Allergen Sensitized Atopic Eczema (AE) (n=10)						
		Geometric Mean (Reads)	Upper Range of Geometric SD	Lower Range of Geometric SD	Geometric Mean (Reads)	Upper Range of Geometric SD	Lower Range of Geometric SD	B Estimate$^{\psi}$	95% CI Lower Bound$^{\psi}$	95% CI Upper Bound$^{\psi}$	Adj p value$^{\psi}$	Geometric Mean (Reads)	Upper Range of Geometric SD	Lower Range of Geometric SD	B Estimate$^{\psi}$	95% CI Lower Bound$^{\psi}$	95% CI Upper Bound$^{\psi}$	Adj p value$^{\psi}$
**METAGENOMICS**	Glycolysis-associated Metabolism	609.93	969.78	383.60	474.54	578.94	388.97	-152.51	-1,378.73	1,073.71	0.179	28.17	51.83	15.31	-588.78	-1,264.41	86.86	0.039*
	Butyrate Metabolism	103.05	177.25	59.92	59.02	96.79	35.99	-330.52	-835.68	174.63	0.191	2.65	5.01	1.40	-438.63	-916.86	39.60	0.029*
	Propionate Metabolism	88.28	126.24	61.73	68.61	111.83	42.09	-84.59	-715.93	546.75	0.187	39.79	65.26	24.26	-138.12	-446.47	170.24	0.031*

		6 Months																
		Control (n=27)			Non-Allergen Sensitized Atopic Eczema (NAE) (n=9)							Allergen Sensitized Atopic Eczema (AE) (n=14)						
		Geometric Mean (Reads)	Upper Range of Geometric SD	Lower Range of Geometric SD	Geometric Mean (Reads)	Upper Range of Geometric SD	Lower Range of Geometric SD	B Estimate$^{\psi}$	95% CI Lower Bound$^{\psi}$	95% CI Upper Bound$^{\psi}$	Adj p value$^{\psi}$	Geometric Mean (Reads)	Upper Range of Geometric SD	Lower Range of Geometric SD	B Estimate$^{\psi}$	95% CI Lower Bound$^{\psi}$	95% CI Upper Bound$^{\psi}$	Adj p value$^{\psi}$
**METAGENOMICS**	Glycolysis-associated Metabolism	2,650.98	3,207.68	2,190.89	1,351.19	1,824.10	1,000.88	-1,284.78	-2,499.33	-70.22	0.827	224.48	286.21	176.06	-2,910.71	-4,860.02	-961.39	0.021*
	Butyrate Metabolism	194.70	319.31	118.72	98.17	140.97	68.36	-93.68	-462.11	274.75	0.735	53.57	61.61	46.58	-385.12	-644.00	-126.25	0.041*
	Propionate Metabolism	156.11	215.43	113.12	72.64	132.94	39.70	-185.71	-333.06	-38.36	0.473	49.41	55.59	43.92	-273.86	-602.17	54.45	0.037*

		12 Months																
		Control (n=26)			Non-Allergen Sensitized Atopic Eczema (NAE) (n=8)							Allergen Sensitized Atopic Eczema (AE) (n=18)						
		Geometric Mean (Reads)	Upper Range of Geometric SD	Lower Range of Geometric SD	Geometric Mean (Reads)	Upper Range of Geometric SD	Lower Range of Geometric SD	B Estimate$^{\psi}$	95% CI Lower Bound$^{\psi}$	95% CI Upper Bound$^{\psi}$	Adj p value$^{\psi}$	Geometric Mean (Reads)	Upper Range of Geometric SD	Lower Range of Geometric SD	B Estimate$^{\psi}$	95% CI Lower Bound$^{\psi}$	95% CI Upper Bound$^{\psi}$	Adj p value$^{\psi}$
**METAGENOMICS**	Glycolysis-associated Metabolism	7,860.14	9,667.97	6,390.36	6,611.45	9,454.38	4,623.39	-1,251.78	-3,568.35	1,064.80	0.491	2,895.93	3,793.66	2,210.63	-5,795.65	-17,667.48	6,076.18	0.034*
	Butyrate Metabolism	512.03	603.17	434.66	326.39	479.79	222.03	-107.63	-694.58	479.32	0.713	236.70	435.29	128.71	-498.22	-960.08	-36.37	0.035*
	Propionate Metabolism	271.88	383.35	192.82	105.99	195.02	57.60	-89.68	-624.05	444.69	0.736	97.16	181.68	51.96	-412.82	-833.29	7.66	0.045*

		Trend Analysis										
		Non-Allergen Sensitized Atopic Eczema (NAE)					Allergen Sensitized Atopic Eczema (AE)					
		Longitudinal Mean Difference (Reads) - B Estimate$^{\varphi}$	95% CI Lower Bound$^{\varphi}$	95% CI Upper Bound$^{\varphi}$	Adj p value for the mean difference$^{\varphi}$	Adj p value for the slope difference$^{\varphi}$	Longitudinal Mean Difference (Reads) - B Estimate$^{\varphi}$	95% CI Lower Bound$^{\varphi}$	95% CI Upper Bound$^{\varphi}$	Adj p value for the mean difference$^{\varphi}$	Adj p value for the slope difference$^{\varphi}$	Adj p value for time trend$^{\varphi}$
**METAGENOMICS**	Glycolysis-associated Metabolism	-469.86	-7,041.45	6,101.73	0.885	0.946	-7,351.96	-13,365.27	-1,338.66	0.018*	0.464	0.624
	Butyrate Metabolism	-525.20	-2,530.03	1,479.63	0.681	0.528	-2,268.75	-4,445.22	-92.28	0.017*	0.344	0.195
	Propionate Metabolism	-721.71	-2,440.64	997.22	0.947	0.932	-2,166.74	-482.09	-482.09	0.037*	0.258	0.979

_ Data are presented as geometric mean and geometric standard deviation range for normalized read counts at metagenomics level & plotted in Figure 3a.

_ 13 bacterial species include *Anaerostipes caccae, Bacteroides fragilis, Blautia producta, Blautia wexlerae, Erysipelatoclostridium ramosum, Eubacterium hallii, Eubacterium limosum, Eubacterium ramulus, Faecalibacterium prausnitzii, Lachnospiraceae bacterium, Ruminococcus gnavus, Ruminococcus sp. JC304, Tyzzerella nexilis* .

$^{\psi}$ General Linear models performed to compare the eczema group (NAE or AE) and controls (reference) at each time point with Bonferroni correction for pair wise comparisons adjusting for baseline values and the 7 mentioned potential confounders (i.e., gender, birth order, mode of delivery, breastfeeding till 6 months, antibiotics during labour, maternal and paternal atopic history)
.

$^{\varphi}$ Linear mixed model was used to evaluate read counts longitudinally and assess the trend significance of the trajectories of eczema group (NAE/AE) compared to controls group (i.e., at the selected four timepoints of week 3, months 3, 6 and 12) among the three clinical groups, adjusted for seven potential confounders (i.e., gender, birth order, mode of delivery, breastfeeding till 6 months, antibiotics during labour, maternal and paternal atopic history).

*Significance at adj p <0.05 between eczema (AE/NAE) and control (reference).

Supplementary Table 4b: Carbohydrate functional gene expression patterns derived from 13 bacterial species at metatrasncriptomics level.

		3 Weeks																
		Control (n=6)			Non-Allergen Sensitized Atopic Eczema (NAE) (n=2)							Allergen Sensitized Atopic Eczema (AE) (n=2)						
		Geometric Mean (Reads)	Upper Range of Geometric SD	Lower Range of Geometric SD	Geometric Mean (Reads)	Upper Range of Geometric SD	Lower Range of Geometric SD	B Estimate[ψ]	95% CI Lower Bound[ψ]	95% CI Upper Bound[ψ]	Adj p value[ψ]	Geometric Mean (Reads)	Upper Range of Geometric SD	Lower Range of Geometric SD	B Estimate[ψ]	95% CI Lower Bound[ψ]	95% CI Upper Bound[ψ]	Adj p value[ψ]
**METATRANSCRIPTOMICS**	Glycolysis-associated Metabolism	73.81	84.88	64.18	3.00	3.60	2.50	-60.23	-152.78	32.32	0.189	1.28	1.63	1.01	-80.64	-185.36	24.08	0.073
	Butyrate Metabolism	28.97	31.58	26.58	6.00	7.20	5.00	-32.78	-103.64	38.08	0.173	5.00	7.50	3.33	-56.72	-213.72	100.28	0.062
	Propionate Metabolism	20.28	22.51	18.27	1.00	1.51	0.66	-25.21	-130.41	79.99	0.083	14.17	32.52	10.15	-45.85	-183.42	91.72	0.058

		3 Months																
		Control (n=11)			Non-Allergen Sensitized Atopic Eczema (NAE) (n=3)							Allergen Sensitized Atopic Eczema (AE) (n=7)						
		Geometric Mean (Reads)	Upper Range of Geometric SD	Lower Range of Geometric SD	Geometric Mean (Reads)	Upper Range of Geometric SD	Lower Range of Geometric SD	B Estimate[ψ]	95% CI Lower Bound[ψ]	95% CI Upper Bound[ψ]	Adj p value[ψ]	Geometric Mean (Reads)	Upper Range of Geometric SD	Lower Range of Geometric SD	B Estimate[ψ]	95% CI Lower Bound[ψ]	95% CI Upper Bound[ψ]	Adj p value[ψ]
**METATRANSCRIPTOMICS**	Glycolysis-associated Metabolism	34.47	61.71	19.26	11.92	18.83	7.54	-71.95	-131.28	-12.62	0.019	5.91	7.74	4.51	-23.88	-128.30	80.54	0.037*
	Butyrate Metabolism	11.80	18.16	7.66	7.34	9.47	5.69	-2.485	-7.184	2.21	0.145	5.48	8.98	3.34	-0.18	-0.68	0.31	0.045*
	Propionate Metabolism	33.87	47.42	24.19	21.74	41.52	11.38	-10.818	-25.183	3.55	0.031*	18.64	32.25	10.78	-20.18	-40.93	0.56	0.026*

		6 Months																
		Control (n=17)			Non-Allergen Sensitized Atopic Eczema (NAE) (n=6)							Allergen Sensitized Atopic Eczema (AE) (n=11)						
		Geometric Mean (Reads)	Upper Range of Geometric SD	Lower Range of Geometric SD	Geometric Mean (Reads)	Upper Range of Geometric SD	Lower Range of Geometric SD	B Estimate[ψ]	95% CI Lower Bound[ψ]	95% CI Upper Bound[ψ]	Adj p value[ψ]	Geometric Mean (Reads)	Upper Range of Geometric SD	Lower Range of Geometric SD	B Estimate[ψ]	95% CI Lower Bound[ψ]	95% CI Upper Bound[ψ]	Adj p value[ψ]
**METATRANSCRIPTOMICS**	Glycolysis-associated Metabolism	57.51	108.69	30.43	117.56	184.57	74.88	48.21	-14.08	110.50	0.454	34.77	43.11	28.04	-40.72	-102.54	21.10	0.189
	Butyrate Metabolism	27.61	47.22	16.15	20.96	38.20	11.50	-0.529	-1.758	0.70	0.386	19.69	24.81	15.63	-10.35	-21.35	0.65	0.044*
	Propionate Metabolism	33.60	38.64	29.22	19.41	30.28	12.44	-5.412	-10.989	0.17	0.0356*	15.14	16.32	14.05	-29.32	-40.79	-17.85	0.017*

		12 Months																
		Control (n=13)			Non-Allergen Sensitized Atopic Eczema (NAE) (n=3)							Allergen Sensitized Atopic Eczema (AE) (n=11)						
		Geometric Mean (Reads)	Upper Range of Geometric SD	Lower Range of Geometric SD	Geometric Mean (Reads)	Upper Range of Geometric SD	Lower Range of Geometric SD	B Estimate[ψ]	95% CI Lower Bound[ψ]	95% CI Upper Bound[ψ]	Adj p value[ψ]	Geometric Mean (Reads)	Upper Range of Geometric SD	Lower Range of Geometric SD	B Estimate[ψ]	95% CI Lower Bound[ψ]	95% CI Upper Bound[ψ]	Adj p value[ψ]
**METATRANSCRIPTOMICS**	Glycolysis-associated Metabolism	181.77	341.73	96.69	121.91	206.02	72.13	-91.54	-233.40	50.32	0.195	72.70	84.33	62.67	-158.86	-249.59	-68.12	0.019*
	Butyrate Metabolism	15.70	21.04	11.71	8.14	9.61	6.90	-1.231	-3.355	0.89	0.243	14.49	26.80	7.83	-1.05	-2.41	0.31	0.041
	Propionate Metabolism	46.07	70.02	30.31	17.44	25.46	11.94	-52.202	-83.261	-21.14	0.204*	16.00	16.64	15.38	-62.11	-125.35	1.13	0.039*

		Trend Analysis										
		Non-Allergen Sensitized Atopic Eczema (NAE)					Allergen Sensitized Atopic Eczema (AE)					
		Longitudinal Mean Difference (Reads) - B Estimate[φ]	95% CI Lower Bound[φ]	95% CI Upper Bound[φ]	Adj p value for the mean difference[φ]	Adj p value for the slope difference[φ]	Longitudinal Mean Difference (Reads) - B Estimate[φ]	95% CI Lower Bound[φ]	95% CI Upper Bound[φ]	Adj p value for the mean difference[φ]	Adj p value for the slope difference[φ]	Adj p value for time trend[φ]
**METATRANSCRIPTOMICS**	Glycolysis-associated Metabolism	-6.39	-65.29	52.50	0.827	0.270	-340.85	-792.39	110.70	0.012*	0.131	<0.001
	Butyrate Metabolism	-12.24	-530.01	505.54	0.563	0.662	-121.90	-692.59	448.78	0.038*	0.054	0.001
	Propionate Metabolism	0.10	-0.18	0.38	0.472	0.128	-92.16	531.19	531.19	0.045*	0.124	0.002

_ Data are presented as geometric mean and geometric standard deviation range for normalized read counts at metatranscriptomics level & plotted in Figure 3b.

_ 13 bacterial species include *Anaerostipes caccae, Bacteroides fragilis, Blautia producta, Blautia wexlerae, Erysipelatoclostridium ramosum, Eubacterium hallii, Eubacterium limosum, Eubacterium ramulus, Faecalibacterium prausnitzii, Lachnospiraceae bacterium, Ruminococcus gnavus, Ruminococcus sp. JC304, Tyzzerella nexilis* .

[ψ] General Linear models performed to compare the eczema group (NAE or AE) and controls (reference) at each time point with Bonferroni correction for pair wise comparisons adjusting for baseline values and the 7 mentioned potential confounders (i.e., gender, birth order, mode of delivery, breastfeeding till 6 months, antibiotics during labour, maternal and paternal atopic history)

[φ] Linear mixed model was used to evaluate read counts longitudinally and assess the trend significance of the trajectories of eczema group (NAE/AE) compared to controls group (i.e., at the selected four timepoints of week 3, months 3, 6 and 12) among the three clinical groups, adjusted for seven potential confounders (i.e., gender, birth order, mode of delivery, breastfeeding till 6 months, antibiotics during labour, maternal and paternal atopic history).

*Significance at adj p <0.05 between eczema (AE/NAE) and control (reference).

Supplementary Table 5: List of metatranscriptomics functional annotation that are significantly different between allergen-sensitized eczema (AE) and controls.

METATRANSCRIPTOMICS	Metabolic Pathway	KEGG Functional Gene ID	Control			Allergen Sensitized Atopic Eczema (AE)						
			Geometric Mean (Reads)	Lower Range of Geometric SD	Upper Range of Geometric SD	Geometric Mean (Reads)	Lower Range of Geometric SD	Upper Range of Geometric SD	B Estimate$^{\psi}$	95% CI Lower Bound$^{\psi}$	95% CI Upper Bound$^{\psi}$	Adj p value$^{\psi}$
**(A)** *Bacteroides fragilis, Eubacterium ramulus, Eubacterium limosum, Eubacterium hallii, Erysipelatoclostridium ramosum, Anaerostipes caccae, Blautia wexlerae, Blautia producta, Lachnospiraceae bacterium, Tyzzerella nexilis, Faecalibacterium prausnitzii, Ruminococcus gnavus, Ruminococcus sp. JC304*	Glycolysis-associated Metabolism	KEGG | ko00010 Glycolysis / Gluconeogenesis | K00382 dihydrolipoamide dehydrogenase [EC:1.8.1.4]	2.91	2.04	4.15	1.15	0.84	1.57	6.77	-10.80	24.34	0.020*
	Glycolysis-associated Metabolism	KEGG | ko00010 Glycolysis / Gluconeogenesis | K00845 glucokinase [EC:2.7.1.2]	3.34	1.43	7.82	1.71	0.94	3.11	4.86	-9.12	18.83	0.013*
	Glycolysis-associated Metabolism	KEGG | ko00010 Glycolysis / Gluconeogenesis | K00927 phosphoglycerate kinase [EC:2.7.2.3]	12.56	2.68	58.96	6.95	1.63	29.65	13.39	-22.49	49.26	0.025*
	Glycolysis-associated Metabolism	KEGG | ko00010 Glycolysis / Gluconeogenesis | K01689 enolase [EC:4.2.1.11]	4.52	1.95	10.48	2.56	0.90	7.26	6.40	-11.37	24.17	0.003*
	Glycolysis-associated Metabolism	KEGG | ko00010 Glycolysis / Gluconeogenesis | K01785 aldose 1-epimerase [EC:5.1.3.3]	5.30	2.30	12.24	2.11	0.79	5.65	7.67	-11.54	26.88	0.034*
	Glycolysis-associated Metabolism	KEGG | ko00010 Glycolysis / Gluconeogenesis | K01835 phosphoglucomutase [EC:5.4.2.2]	6.82	1.63	28.54	3.58	1.09	11.74	3.59	-4.10	11.27	0.027*
	Glycolysis-associated Metabolism	KEGG | ko00010 Glycolysis / Gluconeogenesis | K15633 2,3-bisphosphoglycerate-independent phosphoglycerate mutase [EC:5.4.2.12]	9.10	3.22	25.71	1.98	1.18	3.32	5.90	-8.07	19.86	0.003*
	Glycolysis-associated Metabolism	KEGG | ko00020 Citrate cycle (TCA cycle) | K01681 aconitate hydratase [EC:4.2.1.3]	7.72	1.95	30.50	2.73	0.61	12.24	4.17	-4.72	13.06	0.011*
	Glycolysis-associated Metabolism	KEGG | ko00020 Citrate cycle (TCA cycle) | K01903 succinyl-CoA synthetase beta subunit [EC:6.2.1.5]	8.94	1.85	43.30	2.47	0.85	7.21	8.45	-10.95	27.85	0.017*
	Glycolysis-associated Metabolism	KEGG | ko00020 Citrate cycle (TCA cycle) | K01960 pyruvate carboxylase subunit B [EC:6.4.1.1]	3.54	1.12	11.18	1.49	1.45	1.53	4.12	-5.64	13.89	0.049*
	Glycolysis-associated Metabolism	KEGG | ko00620 Pyruvate metabolism | K00625 phosphate acetyltransferase [EC:2.3.1.8]	12.44	2.55	60.77	4.55	1.16	17.83	12.07	-16.79	40.94	0.012*
	Glycolysis-associated Metabolism	KEGG | ko00620 Pyruvate metabolism | K00656 formate C-acetyltransferase [EC:2.3.1.54]	4.19	1.03	17.07	1.69	1.06	2.69	7.49	-10.99	25.97	0.004*
	Glycolysis-associated Metabolism	KEGG | ko00620 Pyruvate metabolism | K01571 oxaloacetate decarboxylase, alpha subunit [EC:4.1.1.3]	3.26	1.31	8.12	1.59	0.71	3.53	4.42	-6.69	15.52	0.047*
	Glycolysis-associated Metabolism	KEGG | ko00620 Pyruvate metabolism | K04020 phosphotransacetylase	5.59	3.74	8.34	1.82	1.04	3.17	5.31	-7.38	18.01	0.012*
	Propionate Metabolism	KEGG | ko00640 Propanoate metabolism | K11381 2-oxoisovalerate dehydrogenase E1 component [EC:1.2.4.4]	25.64	3.27	201.27	12.53	1.77	88.58	23.48	-26.60	73.56	0.001*
	Butyrate Metabolism	KEGG | ko00650 Butanoate metabolism | K00634 phosphate butyryltransferase [EC:2.3.1.19]	4.50	1.02	19.82	1.83	1.03	3.25	7.61	-9.72	24.93	0.024*
	Butyrate Metabolism	KEGG | ko00650 Butanoate metabolism | K00929 butyrate kinase [EC:2.7.2.7]	8.20	1.75	38.37	2.68	1.16	6.23	4.44	-5.66	14.54	0.011*

METATRANSCRIPTOMICS	Metabolic Pathway	KEGG Functional Gene ID	Control			Allergen Sensitized Atopic Eczema (AE)						
			Geometric Mean (Reads)	Lower Range of Geometric SD	Upper Range of Geometric SD	Geometric Mean (Reads)	Lower Range of Geometric SD	Upper Range of Geometric SD	B Estimate$^{\psi}$	95% CI Lower Bound$^{\psi}$	95% CI Upper Bound$^{\psi}$	Adj p value$^{\psi}$
**(B)** *Escherichia coli, Klebsiella pneumoniae*	Signal transduction	KEGG | ko02020 Two-component system | K02406 flagellin	1.39	0.75	2.59	3.86	1.87	7.99	9.61	-17.33	36.54	0.028*
	Infectious diseases	KEGG | ko05100 Bacterial invasion of epithelial cells | K13735 adhesin/invasin	0.59	0.69	0.50	7.68	2.46	23.96	5.04	-6.28	16.36	0.030*
	Glycolysis-associated metabolism	KEGG | ko00500 Starch and sucrose metabolism | K01194 alpha,alpha-trehalase [EC:3.2.1.28]	1.26	0.84	1.88	4.12	0.56	30.57	2.80	-4.65	10.25	0.022*
	Glycolysis-associated metabolism	KEGG | ko00500 Starch and sucrose metabolism | K05342 alpha,alpha-trehalose phosphorylase [EC:2.4.1.64]	1.57	0.80	3.09	4.64	1.25	17.19	6.64	-8.51	21.79	0.022*
	Glycolysis-associated metabolism	KEGG | ko00500 Starch and sucrose metabolism | K05343 maltose alpha-D-glucosyltransferase / alpha-amylase [EC:5.4.99.16 3.2.1.1]	1.41	0.87	2.31	4.59	2.06	10.21	8.54	-10.94	28.01	0.011*
	Glycan biosynthesis & metabolism	KEGG | ko00540 Lipopolysaccharide biosynthesis | K00912 tetraacyldisaccharide 4'-kinase [EC:2.7.1.130]	1.59	1.06	2.37	3.82	2.13	6.84	5.91	-9.14	20.96	0.043*
	Glycan biosynthesis & metabolism	KEGG | ko00540 Lipopolysaccharide biosynthesis | K00979 3-deoxy-manno-octulosonate cytidylyltransferase (CMP-KDO synthetase) [EC:2.7.7.38]	1.22	0.87	1.71	2.41	1.62	3.60	9.21	-15.69	34.11	0.024*
	Glycan biosynthesis & metabolism	KEGG | ko00540 Lipopolysaccharide biosynthesis | K02847 O-antigen ligase [EC:2.4.1.-]	0.84	1.01	0.70	1.82	1.04	3.17	8.26	-14.03	30.56	0.031*
	Glycan biosynthesis & metabolism	KEGG | ko00540 Lipopolysaccharide biosynthesis | K12975 KDO II ethanolaminephosphotransferase [EC:2.7.8.42]	0.80	1.21	0.53	1.41	0.87	2.31	2.26	-4.09	8.61	0.043*
	Oxidative Phosphorylation	KEGG | ko00190 Oxidative phosphorylation | K00937 polyphosphate kinase [EC:2.7.4.1]	1.12	0.85	1.49	3.59	2.04	6.32	1.35	-1.81	4.50	0.043*
	Oxidative Phosphorylation	KEGG | ko00190 Oxidative phosphorylation | K00336 NADH-quinone oxidoreductase subunit G [EC:1.6.5.3]	0.26	0.18	0.37	1.82	0.65	5.11	8.26	-12.14	28.65	0.036*
	Oxidative Phosphorylation	KEGG | ko00190 Oxidative phosphorylation | K00341 NADH-quinone oxidoreductase subunit L [EC:1.6.5.3]	0.57	0.32	1.01	2.00	0.96	4.15	9.56	-12.93	32.05	0.025*

METATRANSCRIPTOMICS	Metabolic Pathway	KEGG Functional Gene ID	Control			Allergen Sensitized Atopic Eczema (AE)						
			Geometric Mean	Lower Range of Geometric SD	Upper Range of Geometric SD	Geometric Mean	Lower Range of Geometric SD	Upper Range of Geometric SD	B Estimate$^{\psi}$	95% CI Lower Bound$^{\psi}$	95% CI Upper Bound$^{\psi}$	Adj p value$^{\psi}$
**(C)** *Bacteroides fragilis*	Glycan biosynthesis & metabolism	KEGG | ko00511 Other glycan degradation | K01191 alpha-mannosidase [EC:3.2.1.24]	5.22	2.10	13.01	2.68	1.65	4.36	5.08	-8.94	19.10	0.010*
	Glycan biosynthesis & metabolism	KEGG | ko00511 Other glycan degradation | K01206 alpha-L-fucosidase [EC:3.2.1.51]	3.45	1.48	8.04	1.74	1.36	2.22	9.39	-16.76	35.55	0.048*
	Glycan biosynthesis & metabolism	KEGG | ko00531 Glycosaminoglycan degradation | K01205 alpha-N-acetylglucosaminidase [EC:3.2.1.50]	4.78	2.46	9.31	1.46	0.42	5.14	2.79	-4.64	10.21	0.016*
	Glycan biosynthesis & metabolism	KEGG | ko00536 Glycosaminoglycan binding proteins | K01197 hyaluronoglucosaminidase [EC:3.2.1.35]	13.89	3.27	59.03	5.41	1.16	25.26	6.69	-8.59	21.96	0.006*
	Glycan biosynthesis & metabolism	KEGG | ko00540 Lipopolysaccharide biosynthesis | K00677 UDP-N-acetylglucosamine acyltransferase [EC:2.3.1.129]	9.61	2.97	31.16	4.38	0.61	31.16	9.03	-14.23	32.28	0.045*
	Membrane Transport	KEGG | ko02000 Transporters | K02016 iron complex transport system substrate-binding protein	8.54	2.09	34.86	5.53	1.86	16.43	4.94	-7.28	17.15	0.006*
	Membrane Transport	KEGG | ko02000 Transporters | K05776 molybdate transport system ATP-binding protein	3.90	0.91	16.67	2.00	0.60	6.64	6.86	-10.19	23.91	0.009*
	Metabolism of cofactors and vitamins	KEGG | ko00790 Folate biosynthesis | K01633 7,8-dihydroneopterin aldolase/epimerase/oxygenase [EC:4.1.2.25 5.1.99.8 1.13.11.81]	5.39	0.86	33.73	2.77	0.34	22.77	8.52	-13.73	30.76	0.038*
	Metabolism of cofactors and vitamins	KEGG | ko00790 Folate biosynthesis | K01665 para-aminobenzoate synthetase component I [EC:2.6.1.85]	7.14	1.98	25.74	1.33	0.63	2.80	1.26	-1.80	4.31	0.023*
	Metabolism of cofactors and vitamins	KEGG | ko00790 Folate biosynthesis | K06920 7-cyano-7-deazaguanine synthase [EC:6.3.4.20]	5.67	1.76	18.23	1.15	0.84	1.57	4.39	-5.91	14.68	0.048*
	Galactose metabolism	KEGG | ko00052 Galactose metabolism | K01854 UDP-galactopyranose mutase [EC:5.4.99.9]	7.55	1.59	35.87	2.12	0.35	12.77	4.99	-6.01	15.99	0.017*
	Galactose metabolism	KEGG | ko00052 Galactose metabolism | K00965 UDP glucose--hexose-1-phosphate uridylyltransferase [EC:2.7.7.12]	10.44	2.30	47.41	4.05	0.76	21.43	8.77	-13.76	31.30	0.034*

_ The cumulative genes reads contributed by (A) butyrate- and propionate-producing bacteria, which are depleted in AE, (B) potentially pathogenic bacteria, which are enriched in AE and (C) Bacteroidetes fragilis were pooled together for comparsion between groups regardless of timepoints.

$^{\psi}$ General Linear models performed to compare the eczema group (NAE or AE) and controls (reference) at each time point with Bonferroni correction for pair wise comparisons adjusting for baseline values and the 7 mentioned potential confounders (i.e., gender, birth order, mode of delivery, breastfeeding till 6 months, antibiotics during labour, maternal and paternal atopic history). Comparsion of those genes beween NAE and controls were not significant (data not shown).

Supplementary Table 6a: Longitudinal production of 9 detected short chain fatty acids (SCFAs).

Short Chain Fatty Acid (SCFAs)	3 Weeks																
	Control - Reference (n=13)			Non-Allergen Sensitized Atopic Eczema (NAE) (n=5)							Allergen Sensitized Atopic Eczema (AE) (n=5)						
	Geometric Mean (nM)	Upper Range of geometric SD	Lower Range of geometric SD	Geometric Mean (nM)	Upper Range of geometric SD	Lower Range of geometric SD	B Estimate$^{\psi}$	95% CI Lower Bound$^{\psi}$	95% CI Upper Bound$^{\psi}$	Adj p value$^{\psi}$	Geometric Mean (nM)	Upper Range of geometric SD	Lower Range of geometric SD	B Estimate$^{\psi}$	95% CI Lower Bound$^{\psi}$	95% CI Upper Bound$^{\psi}$	Adj p value$^{\psi}$
Acetate	242,837.40	267,121.10	220,761.20	196,877.10	369,331.70	104,947.90	-89,378.40	-427,532.37	248,775.57	0.575	219,995.70	285,994.40	169,227.50	-146,095.97	-603,427.89	311,235.95	0.015*
Butyrate	2,101.24	3,361.98	1,313.28	402.98	721.33	225.13	-2,099.43	-15,377.87	11,179.02	0.042*	597.60	1,057.75	337.62	-2,315.71	-8,810.85	4,179.43	0.013*
Propionate	16,972.94	22,913.47	12,572.55	5,564.98	7,790.98	3,974.99	-2,268.27	-42,976.97	38,440.44	0.041*	8,883.67	10,926.91	7,222.49	-13,020.04	-74,211.36	48,171.29	0.025*
Isobutyrate	732.37	4,485.80	119.57	447.79	7,348.05	27.29	6,415.83	47.50	12,784.16	0.049	99.02	2,592.73	3.78	-1,251.29	-9,864.06	7,361.48	0.757
Isovalerate	353.58	2,195.58	56.94	153.85	1,889.20	12.53	299.44	-3,908.90	4,507.77	0.879	65.65	1,026.55	4.20	-1,700.51	-7,392.02	3,990.99	0.527
Valerate	161.82	1,095.75	23.90	326.25	9,416.85	11.30	1,798.97	-2,204.29	5,802.23	0.347	52.30	270.39	10.11	-1,380.47	-6,794.62	4,033.69	0.589
2-Methylbutyrate	181.16	1,370.77	23.94	48.44	934.49	2.51	1,255.90	-878.69	3,390.48	0.224	51.93	967.08	2.79	-539.76	-3,426.65	2,347.13	0.691
4-Methylvalerate	51.50	1,403.13	1.89	37.94	7,762.24	0.19	5,014.71	1,519.51	8,509.92	0.009	40.58	160.00	10.00	715.39	-4,011.65	5,442.43	0.747
Caproate	35.69	174.57	7.30	47.05	710.97	3.11	253.54	-104.32	611.40	0.149	20.70	135.21	3.17	-203.52	-687.50	280.46	0.378

Short Chain Fatty Acid (SCFAs)	3 Months																
	Control - Reference (n=16)			Non-Allergen Sensitized Atopic Eczema (NAE) (n=11)							Allergen Sensitized Atopic Eczema (AE) (n=10)						
	Geometric Mean (nM)	Upper Range of geometric SD	Lower Range of geometric SD	Geometric Mean (nM)	Upper Range of geometric SD	Lower Range of geometric SD	B Estimate$^{\psi}$	95% CI Lower Bound$^{\psi}$	95% CI Upper Bound$^{\psi}$	Adj p value$^{\psi}$	Geometric Mean (nM)	Upper Range of geometric SD	Lower Range of geometric SD	B Estimate$^{\psi}$	95% CI Lower Bound$^{\psi}$	95% CI Upper Bound$^{\psi}$	Adj p value$^{\psi}$
Acetate	303,348.20	382,218.70	240,752.50	501,598.40	759,808.50	331,137.40	-67,507.82	-223,568.00	88,552.36	0.034*	257,336.90	492,824.50	134,372.90	-130,305.88	-315,416.81	54,805.05	0.016*
Butyrate	14,571.45	23,168.60	9,164.43	9,601.70	9,985.76	9,232.40	-2,566.47	-29,724.87	24,591.93	0.815	5,926.17	10,430.05	3,367.14	-17,197.56	-38,793.02	4,397.89	0.011*
Propionate	38,003.28	60,805.25	23,752.05	45,267.67	89,177.32	22,978.52	-1,417.61	-47,924.42	45,089.20	0.951	29,597.08	30,485.00	28,735.03	-23,468.87	-68,523.86	21,586.12	0.012*
Isobutyrate	5,818.34	40,041.74	845.45	6,711.86	39,200.15	1,149.21	2,829.15	-7,440.79	13,099.09	0.576	2,968.06	17,398.74	506.32	-9,877.19	-19,826.53	72.15	0.052
Isovalerate	1,978.14	26,629.84	146.94	3,140.59	17,236.83	572.22	1,986.75	-3,864.44	7,837.94	0.491	1,554.02	8,933.62	270.32	-4,155.57	-9,824.10	1,512.96	0.144
Valerate	485.68	5,314.61	44.38	312.19	2,320.38	42.00	-1,955.00	-7,568.82	3,658.81	0.48	95.55	989.33	9.23	-3,512.84	-8,951.41	1,925.72	0.196
2-Methylbutyrate	975.33	13,699.66	69.44	1,167.73	11,657.74	116.97	1,220.71	-1,549.34	3,990.76	0.373	923.15	3,084.39	276.30	-2,004.47	-4,688.04	679.11	0.137
4-Methylvalerate	205.13	7,281.77	5.78	51.92	1,864.86	1.45	-1,381.32	-5,602.22	2,839.58	0.507	34.26	786.58	1.49	-2,898.66	-6,987.79	1,190.48	0.157
Caproate	42.80	329.97	5.55	33.62	125.14	9.03	-504.68	-1,716.28	706.92	0.4	25.79	107.28	6.20	-549.73	-1,723.51	624.04	0.345

Short Chain Fatty Acid (SCFAs)	6 Months																
	Control - Reference (n=27)			Non-Allergen Sensitized Atopic Eczema (NAE) (n=9)							Allergen Sensitized Atopic Eczema (AE) (n=14)						
	Geometric Mean (nM)	Upper Range of geometric SD	Lower Range of geometric SD	Geometric Mean (nM)	Upper Range of geometric SD	Lower Range of geometric SD	B Estimate$^{\psi}$	95% CI Lower Bound$^{\psi}$	95% CI Upper Bound$^{\psi}$	Adj p value$^{\psi}$	Geometric Mean (nM)	Upper Range of geometric SD	Lower Range of geometric SD	B Estimate$^{\psi}$	95% CI Lower Bound$^{\psi}$	95% CI Upper Bound$^{\psi}$	Adj p value$^{\psi}$
Acetate	342,293.40	533,977.60	219,418.80	411,304.20	740,530.80	228,445.80	-58,211.51	-334,311.48	217,888.47	0.672	270,385.40	308,239.40	237,180.20	-170,646.88	-395,706.53	54,412.78	0.013*
Butyrate	22,445.78	32,546.38	15,479.85	16,228.97	25,804.06	10,206.90	-9,380.77	-49,873.02	31,111.47	0.642	11,250.27	11,700.29	10,817.57	-15,149.32	-38,156.10	7,857.46	0.034*
Propionate	41,687.72	63,782.22	27,246.88	40,490.03	53,446.85	30,674.27	-5,792.45	-38,625.38	27,040.48	0.723	25,730.87	44,437.22	14,899.17	-17,364.56	-44,127.94	9,398.81	0.019*
Isobutyrate	8,920.87	54,003.80	1,473.64	5,921.47	32,895.83	1,065.91	-4,521.99	-18,934.43	9,890.44	0.529	6,029.90	47,975.43	757.88	-2,429.36	-14,177.49	9,318.77	0.678
Isovalerate	4,869.85	21,547.95	1,100.59	2,480.06	21,825.67	281.81	-3,987.30	-13,859.62	5,885.02	0.419	1,987.51	14,757.16	267.68	-4,786.48	-12,833.79	3,260.82	0.236
Valerate	1,114.35	7,777.65	159.66	599.17	6,403.40	56.07	1,539.91	-10,315.37	13,395.20	0.794	664.11	7,903.04	55.81	-1,441.05	-11,104.75	8,222.64	0.764
2-Methylbutyrate	2,218.58	13,528.02	363.85	1,250.53	8,865.42	176.40	-1,871.47	-6,430.36	2,687.41	0.411	1,301.29	9,501.94	178.21	-1,558.07	-5,274.19	2,158.05	0.401
4-Methylvalerate	371.49	9,538.32	14.47	270.16	5,212.90	14.00	-102.98	-5,205.09	4,999.14	0.968	387.07	7,415.48	20.20	1,399.89	-2,759.04	5,558.82	0.5
Caproate	61.93	432.18	8.87	44.79	393.41	5.10	-0.99	-720.88	718.91	0.998	34.84	267.46	4.54	-111.40	-698.22	475.41	0.703

Short Chain Fatty Acid (SCFAs)	12 Months																
	Control - Reference (n=26)			Non-Allergen Sensitized Atopic Eczema (NAE) (n=8)							Allergen Sensitized Atopic Eczema (AE) (n=18)						
	Geometric Mean (nM)	Upper Range of geometric SD	Lower Range of geometric SD	Geometric Mean (nM)	Upper Range of geometric SD	Lower Range of geometric SD	B Estimate$^{\psi}$	95% CI Lower Bound$^{\psi}$	95% CI Upper Bound$^{\psi}$	Adj p value$^{\psi}$	Geometric Mean (nM)	Upper Range of geometric SD	Lower Range of geometric SD	B Estimate$^{\psi}$	95% CI Lower Bound$^{\psi}$	95% CI Upper Bound$^{\psi}$	Adj p value$^{\psi}$
Acetate	506,144.40	951,551.40	269,225.70	369,872.80	473,437.20	288,963.10	-106,900.55	-362,596.30	148,795.20	0.024*	450,877.10	649,263.00	313,109.10	-113,166.05	-313,044.84	86,712.75	0.029*
Butyrate	58,327.30	83,991.32	40,505.07	45,582.68	72,476.46	28,668.35	-10,740.23	-87,787.19	66,306.72	0.78	44,649.45	53,132.85	37,520.55	-14,875.07	-65,103.11	35,352.96	0.038*
Propionate	63,829.28	114,892.70	35,460.71	51,796.07	65,781.01	40,784.31	-4,456.37	-36,082.96	27,170.22	0.777	57,426.16	108,535.50	30,384.21	-14,593.36	-39,316.04	10,129.32	0.024*
Isobutyrate	18,729.73	34,813.62	10,076.59	13,460.94	39,584.11	4,577.52	-1,714.94	-16,304.48	12,874.61	0.813	16,295.03	61,339.72	4,328.81	1,041.48	-10,363.25	12,446.21	0.855
Isovalerate	8,950.00	20,403.76	3,925.87	4,138.04	29,101.45	588.40	-1,601.62	-13,697.09	10,493.85	0.79	9,150.90	40,458.29	2,069.76	1,570.13	-7,884.96	11,025.22	0.739
Valerate	3,102.59	10,554.12	912.06	3,152.53	17,110.65	580.83	9,400.67	-5,133.43	23,934.77	0.199	2,252.27	18,533.28	273.71	373.60	-10,987.79	11,734.99	0.947
2-Methylbutyrate	4,404.19	10,163.02	1,908.57	2,409.77	17,251.20	336.61	-30.05	-5,298.52	5,238.43	0.991	4,217.12	18,293.98	972.13	541.58	-3,576.82	4,659.98	0.792
4-Methylvalerate	1,276.37	8,425.40	193.36	276.32	4,997.33	15.28	-1,702.38	-7,692.71	4,287.95	0.569	405.76	4,517.53	36.45	-1,469.09	-6,151.77	3,213.58	0.53
Caproate	79.74	351.11	18.11	150.36	717.12	31.53	-73.11	-1,714.49	1,568.28	0.929	92.79	887.50	9.70	535.47	-747.61	1,818.55	0.404

Short Chain Fatty Acid (SCFAs)	Trend Analysis										
	Non-Allergen Sensitized Atopic Eczema (NAE)					Allergen Sensitized Atopic Eczema (AE)					
	Longitudinal Mean Difference (nM) - B Estimate$^{\varphi}$	95% CI Lower Bound$^{\varphi}$	95% CI Upper Bound$^{\varphi}$	Adj p value for the mean difference$^{\varphi}$	Adj p value for the slope difference$^{\varphi}$	Longitudinal Mean Difference (nM) - B Estimate$^{\varphi}$	95% CI Lower Bound$^{\varphi}$	95% CI Upper Bound$^{\varphi}$	Adj p value for the mean difference$^{\varphi}$	Adj p value for the slope difference$^{\varphi}$	Adj p value for time trend$^{\varphi}$
Acetate	-24,090.03	-145,601.18	97,421.12	0.684	0.421	-78,805.09	-187,893.95	30,283.77	0.061	0.791	0.005
Butyrate	-11,039.53	-53,124.94	31,045.89	0.873	0.565	-28,694.71	-81,791.36	24,401.95	0.038*	0.683	<0.001
Propionate	-9,147.67	-29,477.37	11,182.03	0.371	0.759	-17,222.24	-34,141.53	-302.95	0.046*	0.311	<0.001
Isobutyrate	-3,235.48	-13,150.40	6,679.44	0.291	0.107	-761.25	-6,925.36	5,402.85	0.801	0.570	<0.001
Isovalerate	-2,233.12	-6,641.49	2,175.25	0.214	0.609	-851.77	-2,371.03	667.49	0.139	0.825	<0.001
Valerate	-1,425.81	-3,035.40	183.79	0.240	0.866	-1,993.85	-4,525.04	537.34	0.114	0.373	0.066
2-Methylbutyrate	-1,872.34	-3,496.45	-248.23	0.121	0.146	-777.79	-2,291.37	735.80	0.287	0.446	<0.001
4-Methylvalerate	-298.75	-1,240.20	642.70	0.624	0.085	-774.04	-2,242.59	694.51	0.274	0.700	0.030
Caproate	-132.60	-1,621.06	1,355.86	0.359	0.536	-212.84	-524.47	98.78	0.174	0.447	0.027

_ Data are presented as geometric mean and geometric standard deviation range for SCFAs in absolute concentration(nM) & plotted in Figure 1a and Supplementary Figure 1.

_ Glycolysis-associated intermediates [1], butyrate intermediates [2] and propionate intermediates [3] consists of intermediate metabolites belonging to the glycolysis-associated pathway, butyrate and propionate biosynthesis pathways respectively (list of metabolites are listed in Supplementary Table 10).

_ Only predominant intermediates of each pathway are listed.

$^{\psi}$ General Linear models performed to compare the eczema group (NAE or AE) and controls (reference) at each time point with Bonferroni correction for pair wise comparisons adjusting for baseline values and the 7 mentioned potential confounders (i.e., gender, birth order, mode of delivery, breastfeeding till 6 months, antibiotics during labour, maternal and paternal atopic history)
.

$^{\varphi}$ Linear mixed model was used to evaluate concentration in nM (SCFAs) and relative abundance in % (global metabolimics) longitudinally and assess the trend significance of the trajectories of eczema group (NAE/AE) compared to controls group (i.e., at the selected four timepoints of week 3, months 3, 6 and 12) among the three clinical groups, adjusted for seven potential confounders (i.e., gender, birth order, mode of delivery, breastfeeding till 6 months, antibiotics during labour, maternal and paternal atopic history).

*Significance at adj p <0.05 between eczema (AE/NAE) and control (reference).

Supplementary Table 6b: Longitudinal production of predominant metabolites which are significant different in concentration/abundance between groups

Metabolic Pathway	Metabolites	3 Weeks: Control - Reference (n=13) Geometric Mean (%)	Upper Range of geometric SD	Lower Range of geometric SD	Non-Allergen Sensitized Atopic Eczema (NAE) (n=5) Geometric Mean (%)	Upper Range of geometric SD	Lower Range of geometric SD	B Estimate[ψ]	95% CI Lower Bound[ψ]	95% CI Upper Bound[ψ]	Adj p value[ψ]	Allergen Sensitized Atopic Eczema (AE) (n=5) Geometric Mean (%)	Upper Range of geometric SD	Lower Range of geometric SD	B Estimate[ψ]	95% CI Lower Bound[ψ]	95% CI Upper Bound[ψ]	Adj p value[ψ]
Glycolysis-associated Metabolism	Glycolysis-associated Intermediates[1]	20.145	22.563	17.987	28.768	42.179	19.622	3.307	-16.160	22.774	0.758	25.746	46.723	14.187	4.883	-25.360	35.126	0.037*
Glycolysis-associated Metabolism	d_Galactose_2_3_4_5_6_pentakis_O_trimethylsilyl_o_methyloxyme_1E	0.644	0.578	0.718	1.112	0.597	2.072	-1.297	-3.239	0.645	0.811	1.985	0.948	4.158	-0.541	-3.215	2.133	0.712
Glycolysis-associated Metabolism	d_Galactose_2_3_4_5_6_pentakis_O_trimethylsilyl_o_methyloxyme_1Z	0.322	0.093	1.111	0.292	0.027	3.108	-0.013	-0.130	0.104	0.517	3.342	0.340	32.865	2.274	-0.136	4.684	0.026*
Glycolysis-associated Metabolism	439746_6_deoxy_D_glucose2_15.751	0.317	0.033	3.020	1.333	0.412	4.313	-0.662	-10.796	9.472	0.871	2.261	0.659	7.760	2.074	-7.033	11.181	0.032*
Glycolysis-associated Metabolism	d_Glucose_2_3_4_5_6_pentakis_O_trimethylsilyl_o_methyloxyme_1Z	0.202	0.020	2.049	1.357	0.118	15.585	-0.086	-0.267	0.095	0.307	2.441	0.280	21.263	2.011	-0.360	4.382	0.046*
Glycolysis-associated Metabolism	d_Glucose_2_3_4_5_6_pentakis_O_trimethylsilyl_o_methyloxyme_1E	0.375	0.099	1.413	0.526	0.078	3.560	-0.450	-2.302	1.402	0.838	3.113	0.381	25.425	1.478	-1.072	4.028	0.048*
Glycolysis-associated Metabolism	d_Glucose_2_3_4_5_6_pentakis_O_trimethylsilyl_o_methyloxyme_1Z_2	0.155	0.032	0.759	0.890	0.167	4.752	-0.018	-0.047	0.011	0.198	4.908	1.439	16.741	3.016	2.057	3.975	0.045*
Glycolysis-associated Metabolism	107689_L_lacticacid_6.851	0.209	0.131	0.336	0.810	0.083	7.935	-0.435	-4.184	3.314	0.527	1.444	0.337	6.195	0.823	-0.741	2.387	0.011*
Glycolysis-associated Metabolism	84571_lactose2_24.534_3	0.319	0.030	3.413	1.250	0.707	2.208	0.998	-1.365	3.361	0.018*	2.270	0.306	16.855	1.244	-1.809	4.297	0.048*
Glycolysis-associated Metabolism	84571_lactose2_24.534	0.225	0.012	4.306	0.230	0.024	2.167	-0.219	-0.597	0.159	0.582	1.126	0.040	31.442	0.581	-0.601	1.763	0.035*
Butyrate Metabolism	Butyrate Intermediates[2]	0.315	0.372	0.267	0.288	0.319	0.260	0.610	-0.610	1.830	0.297	0.226	0.264	0.193	-0.348	-0.832	0.136	0.031*
Butyrate Metabolism	(R)-3-Hydroxybutyric acid, trimethylsilyl ether, trimethylsilyl ester	0.491	0.110	2.190	0.186	0.022	1.601	-0.607	-1.590	0.376	0.031*	0.147	0.030	0.709	-0.146	-1.502	1.210	0.017*
Propionate Metabolism	Propionate Intermediates[3]	2.331	3.403	1.596	1.632	2.203	1.209	-0.819	-2.084	0.446	0.032*	1.552	2.018	1.194	-1.848	-5.236	1.540	0.044*
Propionate Metabolism	Propanoic acid, 2-oxo-3-(trimethylsilyl)-, trimethylsilyl ester	1.165	0.413	3.288	0.339	0.047	2.472	-0.514	-2.056	1.028	0.048*	0.412	0.142	1.193	-0.515	-1.974	0.944	0.045*
Propionate Metabolism	3-Phenyl-3-trimethylsilyloxypropanoic acid, trimethylsilyl ester	0.820	0.108	6.249	0.387	0.041	3.634	-0.096	-0.360	0.168	0.267	0.836	0.104	6.718	0.034	-0.330	0.398	0.811

Metabolic Pathway	Metabolites	3 Months: Control - Reference (n=16) Geometric Mean (%)	Upper Range of geometric SD	Lower Range of geometric SD	Non-Allergen Sensitized Atopic Eczema (NAE) (n=11) Geometric Mean (%)	Upper Range of geometric SD	Lower Range of geometric SD	B Estimate[ψ]	95% CI Lower Bound[ψ]	95% CI Upper Bound[ψ]	Adj p value[ψ]	Allergen Sensitized Atopic Eczema (AE) (n=10) Geometric Mean (%)	Upper Range of geometric SD	Lower Range of geometric SD	B Estimate[ψ]	95% CI Lower Bound[ψ]	95% CI Upper Bound[ψ]	Adj p value[ψ]
Glycolysis-associated Metabolism	Glycolysis-associated Intermediates[1]	13.649	17.607	10.581	14.162	20.534	9.767	4.508	-10.464	19.480	0.566	19.094	34.698	10.507	5.609	-11.625	22.843	0.036*
Glycolysis-associated Metabolism	d_Galactose_2_3_4_5_6_pentakis_O_trimethylsilyl_o_methyloxyme_1E	0.480	0.163	1.413	0.236	0.069	0.801	0.072	-0.542	0.686	0.068	1.174	0.300	4.600	0.584	-0.202	1.370	0.047*
Glycolysis-associated Metabolism	d_Galactose_2_3_4_5_6_pentakis_O_trimethylsilyl_o_methyloxyme_1Z	0.354	0.142	0.882	0.929	0.439	1.969	0.015	-0.042	0.072	0.069	2.185	1.189	4.014	1.030	-0.024	2.084	0.034*
Glycolysis-associated Metabolism	439746_6_deoxy_D_glucose2_15.751	0.188	0.033	1.052	0.545	0.271	1.099	-0.986	-3.892	1.920	0.936	1.611	0.648	4.005	1.509	-3.548	6.566	0.018*
Glycolysis-associated Metabolism	d_Glucose_2_3_4_5_6_pentakis_O_trimethylsilyl_o_methyloxyme_1Z	0.137	0.027	0.699	0.855	0.134	5.458	-0.018	-0.126	0.090	0.233	1.718	1.390	2.124	1.302	-1.072	3.676	0.028*
Glycolysis-associated Metabolism	d_Glucose_2_3_4_5_6_pentakis_O_trimethylsilyl_o_methyloxyme_1E	0.435	0.186	1.016	0.450	0.291	0.695	0.034	-0.037	0.105	0.739	1.701	1.079	2.681	1.303	-0.037	2.643	0.044*
Glycolysis-associated Metabolism	d_Glucose_2_3_4_5_6_pentakis_O_trimethylsilyl_o_methyloxyme_1Z_2	0.360	0.059	2.184	0.657	0.413	1.046	-0.008	-0.028	0.012	0.877	2.660	1.267	5.585	1.803	-0.021	3.627	0.036*
Glycolysis-associated Metabolism	107689_L_lacticacid_6.851	0.393	0.070	2.214	0.315	0.187	0.530	1.363	-0.904	3.630	0.651	1.081	0.246	4.759	1.711	-0.454	3.876	0.032*
Glycolysis-associated Metabolism	84571_lactose2_24.534_3	0.135	0.050	0.365	0.459	0.082	2.564	-0.009	-0.235	0.217	0.720	0.700	0.196	2.506	0.209	-0.008	0.426	0.419
Glycolysis-associated Metabolism	84571_lactose2_24.534	0.526	0.124	2.234	0.259	0.070	0.949	0.009	-0.164	0.182	0.372	0.310	0.042	2.274	0.113	-0.052	0.278	0.439
Butyrate Metabolism	Butyrate Intermediates[2]	0.394	0.603	0.257	0.366	0.622	0.215	0.023	-0.815	0.861	0.956	0.310	0.353	0.272	-0.195	-0.995	0.605	0.036*
Butyrate Metabolism	(R)-3-Hydroxybutyric acid, trimethylsilyl ether, trimethylsilyl ester	0.444	0.175	1.125	0.159	0.045	0.560	-0.336	-0.784	0.112	0.035*	0.152	0.048	0.476	-0.319	-0.973	0.335	0.028*
Propionate Metabolism	Propionate Intermediates[3]	2.299	3.702	1.428	1.508	1.885	1.206	-0.704	-1.540	0.132	0.025*	1.483	1.928	1.141	-1.021	-2.167	0.125	0.039
Propionate Metabolism	Propanoic acid, 2-oxo-3-(trimethylsilyl)-, trimethylsilyl ester	1.397	0.609	3.202	0.323	0.089	1.169	-0.524	-1.521	0.473	0.038*	0.290	0.059	1.435	-0.308	-1.038	0.422	0.023*
Propionate Metabolism	3-Phenyl-3-trimethylsilyloxypropanoic acid, trimethylsilyl ester	1.540	0.200	11.850	0.405	0.075	2.190	-0.427	-1.251	0.397	0.022*	0.426	0.190	0.957	-0.303	-1.078	0.472	0.044*

Metabolic Pathway	Metabolites	6 Months: Control - Reference (n=27) Geometric Mean (%)	Upper Range of geometric SD	Lower Range of geometric SD	Non-Allergen Sensitized Atopic Eczema (NAE) (n=9) Geometric Mean (%)	Upper Range of geometric SD	Lower Range of geometric SD	B Estimate[ψ]	95% CI Lower Bound[ψ]	95% CI Upper Bound[ψ]	Adj p value[ψ]	Allergen Sensitized Atopic Eczema (AE) (n=14) Geometric Mean (%)	Upper Range of geometric SD	Lower Range of geometric SD	B Estimate[ψ]	95% CI Lower Bound[ψ]	95% CI Upper Bound[ψ]	Adj p value[ψ]
Glycolysis-associated Metabolism	Glycolysis-associated Intermediates[1]	13.385	22.831	7.847	13.049	24.479	6.956	2.020	-13.248	17.288	0.790	14.622	18.862	11.335	7.731	-4.939	20.401	0.022*
Glycolysis-associated Metabolism	d_Galactose_2_3_4_5_6_pentakis_O_trimethylsilyl_o_methyloxyme_1E	0.365	0.151	0.882	0.723	0.108	4.858	-0.306	-1.504	0.892	0.552	0.543	0.212	1.391	0.171	-0.823	1.165	0.127
Glycolysis-associated Metabolism	d_Galactose_2_3_4_5_6_pentakis_O_trimethylsilyl_o_methyloxyme_1Z	0.424	0.174	1.033	0.773	0.118	5.045	-0.020	-0.139	0.099	0.713	0.797	0.489	1.300	0.638	-0.061	1.337	0.037*
Glycolysis-associated Metabolism	439746_6_deoxy_D_glucose2_15.751	0.465	0.143	1.507	0.929	0.658	1.313	0.650	-5.156	6.456	0.143	0.929	0.844	1.022	2.617	-2.200	7.434	0.025
Glycolysis-associated Metabolism	d_Glucose_2_3_4_5_6_pentakis_O_trimethylsilyl_o_methyloxyme_1Z	0.298	0.042	2.090	0.545	0.390	0.763	0.041	-0.097	0.179	0.456	0.626	0.147	2.660	0.806	-0.109	1.721	0.045*
Glycolysis-associated Metabolism	d_Glucose_2_3_4_5_6_pentakis_O_trimethylsilyl_o_methyloxyme_1E	0.310	0.070	1.380	1.092	0.193	6.185	-0.035	-0.473	0.403	0.222	0.933	0.825	1.055	0.917	-0.347	2.181	0.036*
Glycolysis-associated Metabolism	d_Glucose_2_3_4_5_6_pentakis_O_trimethylsilyl_o_methyloxyme_1Z_2	0.296	0.168	0.523	0.839	0.151	4.675	-0.004	-0.030	0.022	0.522	0.413	0.130	1.311	0.004	-0.018	0.026	0.828
Glycolysis-associated Metabolism	107689_L_lacticacid_6.851	0.268	0.052	1.389	0.615	0.096	3.959	0.681	-1.239	2.601	0.815	0.636	0.483	0.837	0.104	-1.489	1.697	0.206
Glycolysis-associated Metabolism	84571_lactose2_24.534_3	0.169	0.038	0.761	0.464	0.253	0.850	-0.083	-0.807	0.641	0.455	0.951	0.136	6.648	0.909	-0.391	2.209	0.014*
Glycolysis-associated Metabolism	84571_lactose2_24.534	0.761	0.102	5.652	0.957	0.131	7.009	-0.011	-0.054	0.032	0.699	0.895	0.261	3.072	0.012	-0.024	0.048	0.405
Butyrate Metabolism	Butyrate Intermediates[2]	0.788	0.969	0.641	0.515	0.619	0.430	-0.574	-1.739	0.591	0.032*	0.534	0.748	0.382	-0.594	-1.561	0.373	0.022*
Butyrate Metabolism	(R)-3-Hydroxybutyric acid, trimethylsilyl ether, trimethylsilyl ester	1.264	0.753	2.121	0.249	0.121	0.512	-0.542	-1.714	0.630	0.782	0.108	0.048	0.243	-0.568	-1.540	0.404	0.038*
Propionate Metabolism	Propionate Intermediates[3]	1.655	2.863	0.957	1.396	2.219	0.878	0.949	-0.259	2.157	0.12	0.985	1.478	0.657	-0.578	-1.281	0.125	0.048*
Propionate Metabolism	Propanoic acid, 2-oxo-3-(trimethylsilyl)-, trimethylsilyl ester	1.062	0.485	2.324	0.536	0.220	1.309	0.159	-0.059	0.377	0.353	0.299	0.123	0.727	-0.451	-1.231	0.329	0.025*
Propionate Metabolism	3-Phenyl-3-trimethylsilyloxypropanoic acid, trimethylsilyl ester	0.491	0.094	2.548	0.465	0.071	3.055	0.157	0.011	0.303	0.240	0.202	0.034	1.182	-0.065	-0.186	0.056	0.670

Metabolic Pathway	Metabolites	12 Months: Control - Reference (n=26) Geometric Mean (%)	Upper Range of geometric SD	Lower Range of geometric SD	Non-Allergen Sensitized Atopic Eczema (NAE) (n=8) Geometric Mean (%)	Upper Range of geometric SD	Lower Range of geometric SD	B Estimate[ψ]	95% CI Lower Bound[ψ]	95% CI Upper Bound[ψ]	Adj p value[ψ]	Allergen Sensitized Atopic Eczema (AE) (n=18) Geometric Mean (%)	Upper Range of geometric SD	Lower Range of geometric SD	B Estimate[ψ]	95% CI Lower Bound[ψ]	95% CI Upper Bound[ψ]	Adj p value[ψ]
Glycolysis-associated Metabolism	Glycolysis-associated Intermediates[1]	10.683	18.955	6.021	10.695	14.603	7.834	-0.601	-11.417	10.215	0.911	11.381	23.173	5.589	3.219	-5.348	11.786	0.452
Glycolysis-associated Metabolism	d_Galactose_2_3_4_5_6_pentakis_O_trimethylsilyl_o_methyloxyme_1E	0.356	0.054	2.364	0.283	0.075	1.069	-0.244	-0.738	0.250	0.670	0.732	0.114	4.721	0.258	-0.133	0.649	0.424
Glycolysis-associated Metabolism	d_Galactose_2_3_4_5_6_pentakis_O_trimethylsilyl_o_methyloxyme_1Z	0.409	0.115	1.455	0.691	0.416	1.148	-0.006	-0.060	0.048	0.957	0.710	0.125	4.030	0.023	-0.019	0.065	0.764
Glycolysis-associated Metabolism	439746_6_deoxy_D_glucose2_15.751	0.345	0.235	0.507	0.588	0.162	2.139	0.301	-2.275	2.877	0.544	0.512	0.282	0.930	0.107	-1.934	2.148	0.309
Glycolysis-associated Metabolism	d_Glucose_2_3_4_5_6_pentakis_O_trimethylsilyl_o_methyloxyme_1Z	6.979	0.897	54.303	0.269	0.075	0.965	-0.069	-0.175	0.037	0.646	0.644	0.460	0.901	0.021	-0.063	0.105	0.699
Glycolysis-associated Metabolism	d_Glucose_2_3_4_5_6_pentakis_O_trimethylsilyl_o_methyloxyme_1E	0.446	0.277	0.721	0.738	0.077	7.113	0.019	-0.037	0.075	0.079	0.491	0.355	0.680	-0.006	-0.051	0.039	0.855
Glycolysis-associated Metabolism	d_Glucose_2_3_4_5_6_pentakis_O_trimethylsilyl_o_methyloxyme_1Z_2	0.298	0.033	2.672	0.799	0.095	6.685	-0.008	-0.025	0.009	0.159	0.527	0.382	0.727	0.001	-0.012	0.014	0.718
Glycolysis-associated Metabolism	107689_L_lacticacid_6.851	0.367	0.329	0.409	0.206	0.184	0.231	-0.271	-2.253	1.711	0.679	0.857	0.478	1.536	0.013	-1.556	1.582	0.065
Glycolysis-associated Metabolism	84571_lactose2_24.534_3	0.437	0.291	0.658	0.568	0.130	2.480	0.033	-0.366	0.432	0.831	0.465	0.121	1.788	0.528	0.212	0.844	0.045*
Glycolysis-associated Metabolism	84571_lactose2_24.534	0.458	0.275	0.763	0.424	0.213	0.847	0.010	-0.032	0.052	0.578	0.640	0.407	1.007	0.035	0.002	0.068	0.912
Butyrate Metabolism	Butyrate Intermediates[2]	0.699	0.860	0.568	0.398	0.477	0.332	-0.433	-1.050	0.184	0.164	0.572	0.824	0.397	0.092	-0.396	0.580	0.704
Butyrate Metabolism	(R)-3-Hydroxybutyric acid, trimethylsilyl ether, trimethylsilyl ester	1.170	0.489	2.796	0.290	0.149	0.564	-0.440	-1.050	0.170	0.510	0.213	0.087	0.519	-0.292	-0.591	0.007	0.035*
Propionate Metabolism	Propionate Intermediates[3]	1.423	2.562	0.791	1.165	1.886	0.720	0.175	-1.834	2.184	0.861	1.277	1.665	0.979	2.015	0.423	3.607	0.014
Propionate Metabolism	Propanoic acid, 2-oxo-3-(trimethylsilyl)-, trimethylsilyl ester	0.929	0.275	3.143	0.429	0.080	2.292	0.055	-1.128	1.238	0.670	0.397	0.084	1.892	0.571	-0.366	1.508	0.802
Propionate Metabolism	3-Phenyl-3-trimethylsilyloxypropanoic acid, trimethylsilyl ester	0.692	0.140	3.419	0.476	0.108	2.097	0.070	-0.674	0.814	0.448	0.411	0.375	0.451	0.321	-0.268	0.910	0.805

Metabolic Pathway	Metabolites	Trend Analysis: Non-Allergen Sensitized Atopic Eczema (NAE) Longitudinal Mean Difference (%) - B Estimate[φ]	95% CI Lower Bound[φ]	95% CI Upper Bound[φ]	Adj p value for the mean difference[φ]	Adj p value for the slope difference[φ]	Allergen Sensitized Atopic Eczema (AE) Longitudinal Mean Difference (%) - B Estimate[φ]	95% CI Lower Bound[φ]	95% CI Upper Bound[φ]	Adj p value for the mean difference[φ]	Adj p value for the slope difference[φ]	Adj p value for time trend[φ]
Glycolysis-associated Metabolism	Glycolysis-associated Intermediates[1]	0.62	-6.72	7.95	0.867	0.568	13.33	-6.87	33.53	0.028*	0.898	0.003
Butyrate Metabolism	Butyrate Intermediates[2]	-0.23	-0.68	0.23	0.326	0.036	8.13	-0.53	16.79	0.035*	0.288	<0.001
Propionate Metabolism	Propionate Intermediates[3]	0.63	-0.17	1.43	0.122	0.797	4.53	-0.17	9.23	0.037*	0.857	0.800

_ Data are presented as geometric mean and geometric standard deviation range for SCFAs in relative abundance (%) & plotted in Figure 1b.

_ Glycolysis-associated intermediates[1], butyrate intermediates[2] and propionate intermediates[3] consists of intermediate metabolites belonging to the glycolysis-associated pathway, butyrate and propionate biosynthesis pathways respectively (list of metabolites are listed in Supplementary Table 10).

_ Only predominant intermediates of each pathway are listed.

[ψ] General Linear models performed to compare the eczema group (NAE or AE) and controls (reference) at each time point with Bonferroni correction for pair wise comparisons adjusting for baseline values and the 7 mentioned potential confounders (i.e., gender, birth order, mode of delivery, breastfeeding till 6 months, antibiotics during labour, maternal and paternal atopic history).

[φ] Linear mixed model was used to evaluate concentration in nM (SCFAs) and relative abundance in % (global metabolimics) longitudinally and assess the trend significance of the trajectories of eczema group (NAE/AE) compared to controls group (i.e., at the selected four timepoints of week 3, months 3, 6 and 12) among the three clinical groups, adjusted for seven potential confounders (i.e., gender, birth order, mode of delivery, breastfeeding till 6 months, antibiotics during labour, maternal and paternal atopic history).

*Significance at adj p <0.05 between eczema (AE/NAE) and control (reference).

Supplementary Table 7: Summary of metagenomics/metatranscriptomics, targeted SCFAs profiling and untargeted global metabolomic profiling

	Metagenomics Sequencing	Metatranscriptomics Sequencing	Targeted SCFAs Profiling	Untargeted Global Metabolic Profiling
Number of Subjects	63	46	63	63
Number of Stool Samples	162	91	162	162
Average Reads ± SD	22,974,600 ± 3,059,722	7,451,917 ± 763,368	-----	-----
Range	10,940,363 – 36,989,702	6,155,374 – 9,608,519	-----	-----

Supplementary Table 8: List of untargetted global metabolites quantitated by GC/TOFMS

#	Metabolites
1	(Methoxymethyl)trimethylsilane
2	(R)-3-Hydroxybutyric acid, trimethylsilyl ether, trimethylsilyl ester
3	(R*,S*)-3,4-Dihydroxybutanoic acid triTMS
4	[107689] L-(+) lactic acid [6.851]
5	[12290] 4-hydroxypyridine [8.204]
6	[12647] L-homoserine 2 [12.359]
7	[138] 5-aminovaleric acid 1 [14.458]
8	[169019] D-threitol [12.954]
9	[17106] L-(-)-fucose 1 [15.614]
10	[239] Beta- alanine 1 [12.044]
11	[304] cholesterol [27.555]
12	[439215] galacturonic acid 1 [17.917]
13	[439746] 6-deoxy-D-glucose 2 [15.751]
14	[439766] citramalic acid [12.63]
15	[440473] L-mimosine 1 [8.863]
16	[440658] melibiose 2 [25.784]
17	[500] 4-guanidinobutyric acid 2 [13.348]
18	[5610] tyramine [17.562]
19	[6508] quinic acid [17.076]
20	[750] glycine [10.456]
21	[750] glycine [10.456]:2
22	[751] DL-glyceraldehyde 1 [8.974]
23	[751] DL-glyceraldehyde 2 [9.186]
24	[84571] lactose 2 [24.534]
25	[84571] lactose 2 [24.534]:3
26	[84571] lactose 2 [24.534]:4
27	[84571] lactose 2 [24.534]:5
28	[8871] 2-hydroxypyridine [6.519]
29	[899] N-acetyl-D-mannosamine 1 [19.177]:2
30	[899] N-acetyl-D-mannosamine 1 [19.177]:3
31	[899] N-acetyl-D-mannosamine 1 [19.177]:4
32	[899] N-acetyl-D-mannosamine 2 [19.339]
33	[899] N-acetyl-D-mannosamine 2 [19.339]:2
34	[92904] 3-indolelactic acid 2 [20.077]
35	1,3-Bis(trimethylsiloxy)benzene
36	1,4-Butanediamine, N,N,N',N'-tetrakis(trimethylsilyl)-
37	10,12-Tricosadiynoic acid, trimethylsilyl ester
38	11-cis-Octadecenoic acid, trimethylsilyl ester
39	1H-Indole-3-ethanamine, N,N,1-tris(trimethylsilyl)-
40	1H-Indole-3-propanoic acid, 1-(trimethylsilyl)-, trimethylsilyl ester
41	1-Monooleoylglycerol trimethylsilyl ether
42	1-O-hexadecylglycerol - bis-trimethylsilyl ether derivative
43	2-Butenedioic acid (E)-, bis(trimethylsilyl) ester (Manual_DP_070809)
44	2-Butenedioic acid (E)-, bis(trimethylsilyl) ester (Manual_DP_070809):2
45	2-Butenedioic acid (E)-, bis(trimethylsilyl) ester (Manual_DP_070809):3
46	2-Butenedioic acid (E)-, bis(trimethylsilyl) ester:2
47	2-Butenoic acid, 3-methyl-2-[(trimethylsilyl)oxy]-, trimethylsilyl ester (Manual_DP_070809)
48	2-Deoxy-galactopyranose, tetrakis(trimethylsilyl)
49	2-Deoxy-galactopyranose, tetrakis(trimethylsilyl):2
50	2-Desoxy-pentos-3-ulose, bis(methoxime),O,O'-bis(trimethylsilyl)-:2
51	2-Ethyl-3-trimethylsilyloxy(trimethylsilyl)butyrate
52	2-Furanacetaldehyde, tetrahydro-à,3,4,5-tetrakis[(trimethylsilyl)oxy]-
53	2-Hydroxyisocaproic acid, trimethylsilyl ether, trimethylsilyl ester
54	2-Piperidinecarboxylic acid, trimethylsilyl ester
55	2-Piperidinone
56	2-Pyrrolidone-5-carboxylic acid, trimethylsilyl ester
57	3,3-Dichloropropyne
58	3,4,5-Trihydroxypentanoic acid, tetrakis(trimethylsilyl)-
59	3,6-Dioxa-2,7-disilaoctane, 2,2,4,7,7-pentamethyl-
60	3,8-Dioxa-2,9-disiladecane, 2,2,9,9-tetramethyl-5,6-bis[[(trimethylsilyl)oxy]methyl]-
61	3-Phenyl-3-trimethylsilyloxypropanoic acid, trimethylsilyl ester
62	3-Pyridinecarboxylic acid, trimethylsilyl ester
63	4-Ketoglucose, bis(O-methyloxime), tetrakis(trimethylsilyl)
64	4-N,N-Dimethylamino(trimethylsilyl)butyrate

65	4-Trimethylsiloxy(trimethylsilyl)valerate
66	5H-Cyclopenta[b]pyridine-3-carbonitrile, 6,7-dihydro-2-mercapto-
67	5-Trimethylsilyloxy-n-valeric acid, trimethylsilyl ester
68	9,12-Octadecadienoic acid (Z,Z)-, trimethylsilyl ester
69	9,12-Octadecadienoic acid, methyl ester, (E,E)-
70	9-Octadecenoic acid (Z)-, methyl ester
71	Acetic acid, [(trimethylsilyl)oxy]-, trimethylsilyl ester
72	à-D-Galactopyranoside, methyl 2,3,4,6-tetrakis-O-(trimethylsilyl)-
73	á-Eudesmol, trimethylsilyl ether
74	á-Hydroxypyruvic acid, trimethylsilyl ether, trimethylsilyl ester
75	Arabinofuranose, 1,2,3,5-tetrakis-O-(trimethylsilyl)-
76	Arabitol, TMS
77	Arabitol, TMS:2
78	Arabitol, TMS:3
79	à-Tocopherol (vitamin E), trimethysilyl derivative
80	Benzeneacetic acid, trimethylsilyl ester
81	Benzenepropanoic acid, 3-[(trimethylsilyl)oxy]-, trimethylsilyl ester
82	Benzenepropanoic acid, à,4-bis[(trimethylsilyl)oxy]-, trimethylsilyl ester
83	Benzenepropanoic acid, à-[(trimethylsilyl)oxy]-, trimethylsilyl ester
84	Benzoic acid trimethylsilyl ester
85	Butanal, 2,3,4-tris[(trimethylsilyl)oxy]-, O-methyloxime, [R-(R*,R*)]-
86	Butane, 1,2,3-tris(trimethylsiloxy)-
87	Butane, 1,2,4-tris(trimethylsiloxy)-
88	Butane, 2,3-bis(trimethylsiloxy)-:3
89	Butane, 2,3-bis(trimethylsiloxy)-:4
90	Butane, 2,3-bis(trimethylsiloxy)-:5
91	Butanedioic acid, bis(trimethylsilyl) ester
92	Butanoic acid, 2-(methoxyimino)-3-methyl-, trimethylsilyl ester
93	Butanoic acid, 2-(methoxyimino)-3-methyl-, trimethylsilyl ester:2
94	Butanoic acid, 2,4-bis[(trimethylsilyl)oxy]-, trimethylsilyl ester
95	Butanoic acid, 2-[(trimethylsilyl)oxy]-, trimethylsilyl ester
96	Butanoic acid, 3-methyl-2-[(trimethylsilyl)oxy]-, trimethylsilyl ester
97	Butanoic acid, 4-[(trimethylsilyl)oxy]-, trimethylsilyl ester
98	Butanoic acid, 4-[bis(trimethylsilyl)amino]-, trimethylsilyl ester:2
99	Cadaverine tri-TMS
100	Cadaverine tri-TMS:2
101	Cadaverine, N,N,N',N'-tetrakis(trimethylsilyl):2
102	cis-4-Trimethylsilyloxy-cyclohexyl(trimethylsilyl)carboxylate
103	Cyclohexanecarboxylic acid, trimethylsilyl ester
104	Decanoic acid, trimethylsilyl ester
105	d-Erythrotetrofuranose, tris-O-(trimethylsilyl)-
106	d-Galactose, 2,3,4,5,6-pentakis-O-(trimethylsilyl)-, o-methyloxyme, (1E)-
107	d-Galactose, 2,3,4,5,6-pentakis-O-(trimethylsilyl)-, o-methyloxyme, (1E)-:2
108	d-Galactose, 2,3,4,5,6-pentakis-O-(trimethylsilyl)-, o-methyloxyme, (1Z)-
109	d-Galactose, 2,3,4,5,6-pentakis-O-(trimethylsilyl)-, o-methyloxyme, (1Z)-:2
110	D-Galactose, 6-deoxy-2,3,4,5-tetrakis-O-(trimethylsilyl)-, O-methyloxime
111	D-Gluconic acid, 2,3,4,5,6-pentakis-O-(trimethylsilyl)-, trimethylsilyl ester (Manual_DP_070809)
112	d-Glucose, 2,3,4,5,6-pentakis-O-(trimethylsilyl)-, o-methyloxyme, (1E)-
113	d-Glucose, 2,3,4,5,6-pentakis-O-(trimethylsilyl)-, o-methyloxyme, (1Z)-
114	d-Glucose, 2,3,4,5,6-pentakis-O-(trimethylsilyl)-, o-methyloxyme, (1Z)-:2
115	DL-Ornithine, N,N,N'-tris(trimethylsilyl)-, trimethylsilyl ester
116	d-Mannose, 2,3,4,5,6-pentakis-O-(trimethylsilyl)-, o-methyloxyme, (1Z)-
117	Dodecanoic acid, trimethylsilyl ester
118	D-Ribofuranose, 1,2,3,5-tetrakis-O-(trimethylsilyl)-
119	D-Ribo-Hexitol, 3-deoxy-1,2,4,5,6-pentakis-O-(trimethylsilyl)-
120	D-Ribose, 2,3,4,5-tetrakis-O-(trimethylsilyl)-
121	D-Xylopyranose, 1,2,3,4-tetrakis-O-(trimethylsilyl)-
122	D-Xylopyranose, 1,2,3,4-tetrakis-O-(trimethylsilyl)-:2
123	Fructose-(D)-O-methyloxime-peak1 (Manual_DP_070809):10
124	Fructose-(D)-O-methyloxime-peak1 (Manual_DP_070809):2
125	Fructose-(D)-O-methyloxime-peak1 (Manual_DP_070809):3
126	Fructose-(D)-O-methyloxime-peak1 (Manual_DP_070809):4
127	Fructose-(D)-O-methyloxime-peak1 (Manual_DP_070809):5
128	Fructose-(D)-O-methyloxime-peak1 (Manual_DP_070809):6
129	Fructose-(D)-O-methyloxime-peak1 (Manual_DP_070809):7
130	Fructose-(D)-O-methyloxime-peak1 (Manual_DP_070809):8

131	Fructose-(D)-O-methyloxime-peak1 (Manual_DP_070809):9
132	Galactose, 2-(acetylamino)-2-deoxy-3,4,5,6-tetrakis-O-(trimethylsilyl)-
133	Glycoside, à-methyl-trtrakis-O-(trimethylsilyl)-:2
134	Hexadecanoic acid, 2,3-bis[(trimethylsilyl)oxy]propyl ester
135	Hexadecanoic acid, trimethylsilyl ester
136	Hexanedioic acid, 2-trimethylsilyloxy-, bis(trimethylsilyl) ester
137	Inosose, 2-desoxy-, O-methyloxime, tetrakis-O-(trimethylsilyl)-
138	l-Alanine, N-(trimethylsilyl)-, trimethylsilyl ester
139	L-Asparagine, Nç-[2-(acetylamino)-4-O-[2-(acetylamino)-2-deoxy-3,4,6-tris-O-(trimethylsilyl)-á-D-glucopyranosyl]-2-deoxy-3,6-bis-O-(trimethylsilyl)-á-D-glucopyranosyl]-:2
140	Lauric acid, 2,3-bis(trimethylsiloxy)propyl ester
141	L-Proline, 5-oxo-1-(trimethylsilyl)-, trimethylsilyl ester
142	L-Threonic acid, tris(trimethylsilyl) ether, trimethylsilyl ester
143	L-Threonic acid, tris(trimethylsilyl) ether, trimethylsilyl ester:2
144	L-Tyrosine, N,O-bis(trimethylsilyl)-, trimethylsilyl ester
145	M000000_A193012-101-xxx_NA_1930,4_PRED_VAR5_ALK_NA
146	M000014_A192003-101-xxx_NA_1913,61_TRUE_VAR5_ALK_Lysine (4TMS)
147	M000233_A183002-101-xxx_NA_1817,76_PRED_VAR5_ALK_Pinitol, D- (5TMS)
148	M000425_A200006-101-xxx_NA_1987,66_TRUE_VAR5_ALK_Pantothenic acid, D- (3TMS)
149	M000429_A139011-101-xxx_NA_1552,41_TRUE_VAR5_ALK_Propane-1,2-diol, 3-amino (4TMS)
150	M000463_A164003-101-xxx_NA_1633,29_TRUE_VAR5_ALK_Benzoic acid, 4-hydroxy- (2TMS)
151	M000677_A153005-101-xxx_NA_1524,61_TRUE_VAR5_ALK_Cytosine (2TMS)
152	M000717_A252001-101-xxx_NA_2504,62_PRED_VAR5_ALK_Tryptamine, 5-hydroxy- (3TMS)
153	M000980_A175013-101-xxx_NA_1758,1_TRUE_VAR5_ALK_Putrescine, N-acetyl- (2TMS)
154	M001033_A198011-101-xxx_NA_1987,02_TRUE_VAR5_ALK_Glycyl-proline (3TMS)
155	Melibiose, octakis(trimethylsilyl)- (Manual_DP_070809)
156	Melibiose, octakis(trimethylsilyl)- (Manual_DP_070809):10
157	Melibiose, octakis(trimethylsilyl)- (Manual_DP_070809):12
158	Melibiose, octakis(trimethylsilyl)- (Manual_DP_070809):2
159	Melibiose, octakis(trimethylsilyl)- (Manual_DP_070809):3
160	Melibiose, octakis(trimethylsilyl)- (Manual_DP_070809):4
161	Melibiose, octakis(trimethylsilyl)- (Manual_DP_070809):5
162	Melibiose, octakis(trimethylsilyl)- (Manual_DP_070809):6
163	Melibiose, octakis(trimethylsilyl)- (Manual_DP_070809):7
164	Melibiose, octakis(trimethylsilyl)- (Manual_DP_070809):8
165	Melibiose, octakis(trimethylsilyl)- (Manual_DP_070809):9
166	meso-Erythritol
167	meso-Erythritol:2
168	Myo-Inositol, 1,2,3,4,5,6-hexakis-O-(trimethylsilyl)-
169	Myo-Inositol, 1,2,3,4,5,6-hexakis-O-(trimethylsilyl)-:2
170	Myo-Inositol, 1,2,3,4,5,6-hexakis-O-(trimethylsilyl)-:3
171	Myo-Inositol, 1,2,3,4,5,6-hexakis-O-(trimethylsilyl)-:5
172	Myo-Inositol, 1,2,3,4,5,6-hexakis-O-(trimethylsilyl)-:7
173	N,O,O-Tris(trimethylsilyl)-L-threonine
174	n-Pentadecanoic acid, trimethylsilyl ester
175	Octadecanoic acid, 9,10-bis[(trimethylsilyl)oxy]-, ethyl ester
176	Octadecanoic acid, trimethylsilyl ester
177	Octanoic acid, trimethylsilyl ester
178	Oleic acid, trimethylsilyl ester:2
179	Palmitelaidic acid, trimethylsilyl ester
180	Palmitelaidic acid, trimethylsilyl ester:2
181	Pentanedioic acid, 2-[(trimethylsilyl)oxy]-, bis(trimethylsilyl) ester
182	Pentanedioic acid, bis(trimethylsilyl) ester
183	Pentanoic acid, 2-(methoxyimino)-3-methyl-, trimethylsilyl ester
184	Pentanoic acid, 3-methyl-2-[(trimethylsilyl)oxy]-, trimethylsilyl ester
185	Pentanoic acid, 3-methyl-2-[(trimethylsilyl)oxy]-, trimethylsilyl ester:2
186	Pentitol, 1-desoxytetrakis-O-(trimethylsilyl)-
187	Propanedioic acid, bis(trimethylsilyl) ester
188	Propanetriol, 2-methyl-, tris-O-(trimethylsilyl)-
189	Propanetriol, 2-methyl-, tris-O-(trimethylsilyl)-:2
190	Propanetriol, 2-methyl-, tris-O-(trimethylsilyl)-:3
191	Propanoic acid, 2,3-bis[(trimethylsilyl)oxy]-, trimethylsilyl ester
192	Propanoic acid, 2-[(trimethylsilyl)oxy]-, trimethylsilyl ester
193	Propanoic acid, 2-oxo-3-(trimethylsilyl)-, trimethylsilyl ester
194	Propanoic acid, 3-[(trimethylsilyl)oxy]-, trimethylsilyl ester
195	Pyrimidine, 2,4-bis[(trimethylsilyl)oxy]-
196	Pyrimidine, 5-methyl-2,4-bis[(trimethylsilyl)oxy]-

197	Pyrimidine, 5-methyl-2,4-bis[(trimethylsilyl)oxy]-:2
198	Ribitol, 1,2,3,4,5-pentakis-O-(trimethylsilyl)- (Manual_DP_070809)
199	Ribitol, 1,2,3,4,5-pentakis-O-(trimethylsilyl)- (Manual_DP_070809):3
200	Ribonic acid gamma lactone, MOX TMS:2
201	Ribonic acid, 2,3,4,5-tetrakis-O-(trimethylsilyl)-, trimethylsilyl ester
202	Ribonic acid, 2,3,4,5-tetrakis-O-(trimethylsilyl)-, trimethylsilyl ester:2
203	Ribonic acid, 2,3,4,5-tetrakis-O-(trimethylsilyl)-, trimethylsilyl ester:3
204	Ribose
205	Ribose:3
206	Ribose:4
207	Sebacic acid, bis(trimethylsilyl) ester:2
208	Sedoheptulose, o-methyloxime, hexakis-O-(trimethylsilyl)-
209	Silanamine, 1,1,1-trimethyl-N-(trimethylsilyl)-N-[2-[(trimethylsilyl)oxy]ethyl]-:4
210	Silanamine, 1,1,1-trimethyl-N-(trimethylsilyl)-N-[2-[(trimethylsilyl)oxy]ethyl]-:5
211	Silanamine, 1,1,1-trimethyl-N-(trimethylsilyl)-N-[2-[(trimethylsilyl)oxy]ethyl]-:6
212	Silanamine, N-[2-[3,4-bis[(trimethylsilyl)oxy]phenyl]ethyl]-1,1,1-trimethyl-N-(trimethylsilyl)-:2
213	Silanamine, N-[2-[3,4-bis[(trimethylsilyl)oxy]phenyl]ethyl]-1,1,1-trimethyl-N-(trimethylsilyl)-:3
214	Spiro[5,5]undecane, 3-oxa-4,4-dimethyl-8-aza-8-ethoxycarbonylmethyl-
215	Succinic anhydride, O,O'-bis(trimethylsilyl)-
216	Tetradecanoic acid, trimethylsilyl ester
217	trans-9-Octadecenoic acid, trimethylsilyl ester
218	Trimethylsilyl ether of glycerol
219	Trimethylsilyl ether of glycerol (Manual_DP_070809):2
220	Xylose-MOX-TMS-peak1
221	Xylose-MOX-TMS-peak2
222	Xylose-MOX-TMS-peak2:2

Supplementary Table 9a: List of butyrate-producing and propionate-producing bacteria at family level

Butyrate-Producing Bacterial Families					
ID	Phylum	Families	Spearman Correlation Coefficient (r)	P-Value	Shortlisted Butyrate Producing Families
1	Firmicutes	Ruminococcaceae	0.787930411	< 0.01	Ruminococcaceae
2	Bacteroidetes	Bacteroidaceae	0.766123709	< 0.01	Bacteroidaceae
3	Firmicutes	Eubacteriaceae	0.74549566	< 0.01	Eubacteriaceae
4	Firmicutes	Lachnospiraceae	0.725531182	< 0.01	Lachnospiraceae
5	Firmicutes	Erysipelotrichaceae	0.710063834	< 0.01	Erysipelotrichaceae
6	Proteobacteria	Rhodospirillaceae	0.685488466	< 0.01	Rhodospirillaceae
7	Proteobacteria	Heliobacteriaceae	0.676935644	< 0.01	Heliobacteriaceae
8	Proteobacteria	Succinivibrionaceae	0.673222218	< 0.01	Succinivibrionaceae
9	Firmicutes	Thermoanaerobacteraceae	0.661367427	< 0.01	Thermoanaerobacteraceae
10	Firmicutes	Alicyclobacillaceae	0.659773694	< 0.01	Alicyclobacillaceae
11	Actinobacteria	Eggerthellaceae	0.657864743	< 0.01	Eggerthellaceae
12	Firmicutes	Haloplasmataceae	0.65670561	< 0.01	Haloplasmataceae
13	Firmicutes	Peptococcaceae	0.636480081	< 0.01	
14	Firmicutes/Synergistetes	Synergistaceae	0.614677786	< 0.01	
15	Firmicutes	Paenibacillaceae	0.61416166	< 0.01	
16	Firmicutes	Oscillospiraceae	0.606699029	< 0.01	
17	Firmicutes	Catabacteriaceae	0.565055073	< 0.01	
18	Spirochaetes	Spirochaetaceae	0.556421087	< 0.01	
19	Firmicutes	Clostridiaceae	0.494022392	< 0.01	
20	Firmicutes	Peptostreptococcaceae	0.490750973	< 0.01	
21	Firmicutes	Acholeplasmataceae	0.481940545	< 0.01	
22	Fusobacteria	Leptotrichiaceae	0.466366734	< 0.01	
23	Fusobacteria	Fusobacteriaceae	0.454430239	< 0.01	
24	Firmicutes	Aerococcaceae	0.45363929	< 0.01	
25	Firmicutes/Synergistetes	Syntrophomonadaceae	0.453321504	< 0.01	
26	Firmicutes	Clostridiales Family XIII. Incertae Sedis	0.444403705	< 0.01	
27	Tenericutes	Entomoplasmataceae	0.438938525	< 0.01	
28	Firmicutes	Thermoanaerobacterales Family IV. Incertae Sedis	0.432356908	< 0.01	
29	Firmicutes	Caldicoprobacteraceae	0.423335809	< 0.01	
30	Firmicutes	Thermoanaerobacterales Family III. Incertae Sedis	0.411844952	< 0.01	
31	Tenericutes	Helicobacteraceae	0.400557239	< 0.01	
32	Firmicutes	Halobacteroidaceae	0.399567416	< 0.01	
33	Thermotogae	Thermotogaceae	0.397661381	< 0.01	
34	Firmicutes	Bacillaceae	0.393878579	< 0.01	
35	Spirochaetes	Brachyspiraceae	0.392520149	< 0.01	
36	Firmicutes	Carnobacteriaceae	0.388200418	< 0.01	
37	Actinobacteria	Coriobacteriaceae	0.382678556	< 0.01	
38	Firmicutes	Defluviitaleaceae	0.38184146	< 0.01	
39	Firmicutes	Sporomusaceae	0.376372595	< 0.01	
40	Firmicutes	Planococcaceae	0.375788954	< 0.01	
41	Proteobacteria	Desulfovibrionaceae	0.370116357	< 0.01	
42	Proteobacteria	Desulfobacteraceae	0.369126375	< 0.01	
43	Cyanobacteria	Microcoleaceae	0.354411791	< 0.01	
44	Actinobacteria	Promicromonosporaceae	0.353405231	< 0.01	
45	Actinobacteria	Atopobiaceae	0.350591701	< 0.01	
46	Proteobacteria	Legionellaceae	0.349393303	< 0.01	
47	Actinobacteria	Sanguibacteraceae	0.349222566	< 0.01	
48	Proteobacteria	Sutterellaceae	0.347157751	< 0.01	
49	Proteobacteria	Cardiobacteriaceae	0.336635256	< 0.01	
50	Firmicutes	Acidaminococcaceae	0.335740854	< 0.01	
51	Firmicutes	Selenomonadaceae	0.332946634	< 0.01	
52	Actinobacteria	Dermacoccaceae	0.330525203	< 0.01	
53	Planctomycetes	Candidatus Brocadiaceae	0.323468736	< 0.01	
54	Firmicutes	Halanaerobiaceae	0.321851755	< 0.01	
55	Chlorobacteria	Ktedonobacteraceae	0.321845567	< 0.01	
56	Actinobacteria	Actinomycetaceae	0.321297813	< 0.01	
57	Firmicutes	Sporolactobacillaceae	0.314694749	< 0.01	
58	Proteobacteria	Campylobacteraceae	0.313859536	< 0.01	
59	Actinobacteria	Gordoniaceae	0.311908172	< 0.01	
60	Fibrobacteres	Fibrobacteraceae	0.308892271	< 0.01	
61	Deinococcus–Thermus	Trueperaceae	0.303538414	< 0.01	
62	Cyanobacteria	Nitrospinaceae	0.298171803	0.011	
63	Proteobacteria	Saccharospirillaceae	0.297079269	0.012	
64	Firmicutes	Mycoplasmataceae	0.293197556	0.014	
65	Proteobacteria	Moritellaceae	0.285371769	0.018	
66	Bacteroidetes	Balneolaceae	0.283412207	0.019	
67	Actinobacteria	Cellulomonadaceae	0.280873799	0.021	
68	Firmicutes	Hyellaceae	0.270185578	0.030	
69	Firmicutes	Thermodesulfobiaceae	0.268524488	0.032	
70	Firmicutes	Symbiobacteriaceae	0.265408544	0.035	
71	Actinobacteria	Tsukamurellaceae	0.262360405	0.039	
72	Cyanobacteria	Chroococcaceae	0.254026935	0.050	

_ Correlation between bacterial read counts and concentration (nM) of SCFAs was determined by Spearman's rank correlation coefficient.
_ Shortlisted major butyrate and propionate bacteria families were based on threshold of correlation coefficient = 0.65 and p value <0.05.

Propionate-Producing Bacterial Families					
**ID**	**Phylum**	**Families**	**Spearman Correlation Coefficient (r)**	**P-Value**	**Shortlisted Propionate Producing Families**
1	*Firmicutes*	*Eubacteriaceae*	0.745829082	< 0.01	*Eubacteriaceae*
2	*Firmicutes*	*Lachnospiraceae*	0.73079979	< 0.01	*Lachnospiraceae*
3	*Bacteroidetes*	*Bacteroidaceae*	0.7277526	< 0.01	*Bacteroidaceae*
4	*Firmicutes*	*Selenomonadaceae*	0.71972279	< 0.01	*Selenomonadaceae*
5	*Actinobacteria*	*Coriobacteriaceae*	0.690121189	< 0.01	*Coriobacteriaceae*
6	*Firmicutes*	*Ruminococcaceae*	0.688876074	< 0.01	*Ruminococcaceae*
7	*Firmicutes*	*Veillonellaceae*	0.683267933	< 0.01	*Veillonellaceae*
8	*Proteobacteria*	*Desulfovibrionaceae*	0.681528451	< 0.01	*Desulfovibrionaceae*
9	*Firmicutes*	*Peptostreptococcaceae*	0.679585103	< 0.01	*Peptostreptococcaceae*
10	*Firmicutes*	*Erysipelotrichaceae*	0.675128041	< 0.01	*Erysipelotrichaceae*
11	*Fusobacteria*	*Fusobacteriaceae*	0.662556965	< 0.01	*Fusobacteriaceae*
12	*Firmicutes*	*Catabacteriaceae*	0.605381319	< 0.01	
13	*Proteobacteria*	*Succinivibrionaceae*	0.595850513	< 0.01	
14	*Firmicutes*	*Ruminococcaceae*	0.560803092	< 0.01	
15	*Firmicutes*	*Heliobacteriaceae*	0.553852657	< 0.01	
16	*Actinobacteria*	*Atopobiaceae*	0.547200131	< 0.01	
17	*Firmicutes*	*Oscillospiraceae*	0.538264907	< 0.01	
18	*Spirochaetes*	*Spirochaetaceae*	0.515202748	< 0.01	
19	*Firmicutes*	*Clostridiales Family XIII. Incertae Sedis*	0.49962967	< 0.01	
20	*Firmicutes*	*Peptococcaceae*	0.49691749	< 0.01	
21	*Firmicutes/Synergistetes*	*Synergistaceae*	0.49386305	< 0.01	
22	*Firmicutes*	*Aerococcaceae*	0.488004101	< 0.01	
23	*Actinobacteria*	*Dermacoccaceae*	0.483884306	< 0.01	
24	*Firmicutes/Synergistetes*	*Syntrophomonadaceae*	0.477387236	< 0.01	
25	*Actinobacteria*	*Eggerthellaceae*	0.472240499	< 0.01	
26	*Firmicutes*	*Paenibacillaceae*	0.467625511	< 0.01	
27	*Firmicutes*	*Thermoanaerobacteraceae*	0.451769747	< 0.01	
28	*Actinobacteria*	*Actinomycetaceae*	0.440446482	< 0.01	
29	*Tenericutes*	*Haloplasmataceae*	0.437165001	< 0.01	
30	*Firmicutes*	*Halobacteroidaceae*	0.43401652	< 0.01	
31	*Firmicutes*	*Caldicoprobacteraceae*	0.423241745	< 0.01	
32	*Firmicutes*	*Sporolactobacillaceae*	0.420436997	< 0.01	
33	*Proteobacteria*	*Syntrophaceae*	0.416105884	< 0.01	
34	*Firmicutes*	*Thermoanaerobacterales Family III. Incertae Sedis*	0.414363315	< 0.01	
35	*Fibrobacteres*	*Fibrobacteraceae*	0.412580967	< 0.01	
36	*Bacteroidetes*	*Rikenellaceae*	0.410052267	< 0.01	
37	*Firmicutes*	*Acidaminococcaceae*	0.408283613	< 0.01	
38	*Firmicutes*	*Sporomusaceae*	0.396865494	0.012	
39	*Tenericutes*	*Entomoplasmataceae*	0.396551083	0.012	
40	*Fusobacteria*	*Leptotrichiaceae*	0.392978997	0.014	
41	*Firmicutes*	*Carnobacteriaceae*	0.391607191	0.015	
42	*Firmicutes*	*Halanaerobiaceae*	0.388822574	0.016	
43	*Firmicutes*	*Bacillaceae*	0.388120712	0.017	
44	*Spirochaetes*	*Brachyspiraceae*	0.38731463	0.017	
45	*Proteobacteria*	*Rhodospirillaceae*	0.386284728	0.018	
46	*Bacteroidetes*	*Porphyromonadaceae*	0.384854938	0.019	
47	*Proteobacteria*	*Sutterellaceae*	0.384031587	0.019	
48	*Firmicutes*	*Thermoanaerobacterales Family IV. Incertae Sedis*	0.379881423	0.022	
49	*Bacteroidetes*	*Prevotellaceae*	0.376006018	0.025	
50	*Firmicutes*	*Mycoplasmataceae*	0.369057566	0.032	
51	*Firmicutes*	*Alicyclobacillaceae*	0.368368322	0.032	
52	*Firmicutes*	*Defluviitaleaceae*	0.364613007	0.036	
53	*Actinobacteria*	*Sanguibacteraceae*	0.362453421	0.039	
54	*Proteobacteria*	*Helicobacteraceae*	0.361826545	0.040	
55	*Cyanobacteria*	*Hapalosiphonaceae*	0.359441656	0.043	
56	*Bacteroidetes*	*Balneolaceae*	0.354084719	0.050	

Supplementary Table 9b: List of dominant butyrate-producing and propionate-producing bacteria at species level

Butyrate-Producing Bacterial Species				
**ID**	**Species**	**Spearman Correlation Coefficient (r)**	**P-Value**	**Shortlisted Butyrate Producing Species**
1	*Ruminococcus gnavus*	0.744532229	< 0.01	*Ruminococcus gnavus*
2	*Blautia wexlerae*	0.729076051	< 0.01	*Blautia wexlerae*
3	*Lachnospiraceae bacterium*	0.718675217	< 0.01	*Lachnospiraceae bacterium*
4	*Eubacterium ramulus*	0.711229788	< 0.01	*Eubacterium ramulus*
5	*Faecalibacterium prausnitzii*	0.707269434	< 0.01	*Faecalibacterium prausnitzii*
6	*Blautia producta*	0.69381914	< 0.01	*Blautia producta*
7	*Tyzzerella nexilis*	0.673244024	< 0.01	*Tyzzerella nexilis*
8	*Erysipelatoclostridium ramosum*	0.669114127	< 0.01	*Erysipelatoclostridium ramosum*
9	*Bacteroides fragilis*	0.660869386	< 0.01	*Bacteroides fragilis*
10	*Erysipelotrichaceae bacterium*	0.639843193	< 0.01	
11	*Firmicutes bacterium CAG:424*	0.633603798	< 0.01	
12	*Clostridium innocuum*	0.583384862	< 0.01	
14	*Clostridiaceae bacterium MS3*	0.575192585	< 0.01	
13	*Ruminococcus torques*	0.554478676	< 0.01	
15	*Eubacterium rectale*	0.530230483	< 0.01	
16	*Ruminococcus sp. CAG:60*	0.517433884	< 0.01	
17	*Eubacterium sp. 3_1_31*	0.51355323	< 0.01	
18	*Blautia sp. CAG:257*	0.508339505	< 0.01	
19	*Clostridium clostridioforme*	0.507968097	< 0.01	
20	*Clostridium bolteae*	0.506826072	< 0.01	
21	*Lachnospiraceae bacterium 2_1_58FAA*	0.506619847	< 0.01	
22	*Clostridium hylemonae*	0.504541183	< 0.01	
23	*Clostridioides difficile*	0.495506379	< 0.01	
24	*Hungatella hathewayi*	0.482586493	< 0.01	
25	*Ruminococcus sp. 5_1_39BFAA*	0.473114389	< 0.01	
26	*Clostridiales bacterium VE202-14*	0.467874424	< 0.01	
27	*Ruminococcus gnavus CAG:126*	0.454185904	< 0.01	
28	*Blautia sp. CAG:37*	0.452972905	< 0.01	
29	*Megamonas hypermegale*	0.445144851	< 0.05	
30	*Clostridium perfringens*	0.42509033	< 0.05	
31	*Clostridium spiroforme*	0.41772701	< 0.05	
32	*Subdoligranulum sp. 4_3_54A2FAA*	0.416314756	< 0.05	
33	*Megasphaera micronuciformis*	0.408712379	< 0.05	
34	*Coprobacillus sp. 8_2_54BFAA*	0.402201805	< 0.05	
35	*Coprobacillus sp. D7*	0.401195579	< 0.05	
36	*Coprobacillus sp. 3_3_56FAA*	0.393801701	< 0.05	
37	*Clostridium neonatale*	0.385887163	< 0.05	
38	*Coprobacillus sp. CAG:183*	0.380849115	< 0.05	
39	*Enterococcus gallinarum*	0.369176239	< 0.05	
40	*Enterococcus faecium*	0.33124955	< 0.05	
41	*Enterococcus casseliflavus*	0.317332335	< 0.05	
42	*Eggerthella lenta*	0.315898184	< 0.05	
43	*Enterococcus raffinosus*	0.314644543	< 0.05	

_ Correlation between bacterial read counts and concentration (nM) of SCFAs was determined by Spearman's rank correlation coefficient.
_ Shortlisted major butyrate and propionate bacteria species were based on threshold of correlation coefficient = 0.65 and p value <0.05.

Propionate-Producing Bacterial Species				
ID	Species	Spearman Correlation Coefficient (r)	P-Value	Shortlisted Propionate Producing Species
1	*Eubacterium limosum*	0.714366492	< 0.01	*Eubacterium limosum*
2	*Blautia wexlerae*	0.712905987	< 0.01	*Blautia wexlerae*
3	*Ruminococcus sp_ JC304*	0.689636013	< 0.01	*Ruminococcus sp_ JC304*
4	*Eubacterium hallii*	0.680017424	< 0.01	*Eubacterium hallii*
5	*Anaerostipes caccae*	0.670513248	< 0.01	*Anaerostipes caccae*
6	*Bacteroides fragilis*	0.661971226	< 0.01	*Bacteroides fragilis*
7	*Eubacterium sp. 3_1_31*	0.620039082	< 0.01	
8	*Ruminococcus torques*	0.576619512	< 0.01	
9	*Erysipelotrichaceae bacterium 3_1_53*	0.447750478	< 0.01	
10	*Parabacteroides distasonis*	0.384976052	< 0.01	
11	*Ruminococcus sp. CAG:60*	0.339471223	< 0.01	
12	*Bacteroides vulgatus*	0.335408184	< 0.01	
14	*Firmicutes bacterium CAG:424*	0.333968786	< 0.01	
13	*Bacteroides uniformis*	0.33257772	< 0.01	
15	*Bacteroides ovatus*	0.329615808	< 0.01	
16	*Blautia sp. CAG:257*	0.317647659	< 0.01	
17	*Bacteroides caccae*	0.313023515	< 0.01	
18	*Megamonas hypermegale*	0.31196808	< 0.01	
19	*Tyzzerella nexilis*	0.30900875	< 0.01	
20	*Subdoligranulum sp. 4_3_54A2FAA*	0.303692922	< 0.01	
21	*Bacteroides thetaiotaomicron*	0.30341392	< 0.01	
22	*Clostridiales bacterium VE202-14*	0.302216695	< 0.01	
23	*Holdemanella biformis*	0.301460504	< 0.05	
24	*Clostridium hylemonae*	0.28871409	< 0.05	
25	*Bacteroides dorei*	0.223998073	< 0.05	
26	*Collinsella sp. 4_8_47FAA*	0.192266059	< 0.05	
27	*Ruminococcus sp. 5_1_39BFAA*	0.186629057	< 0.05	
28	*Enterococcus gallinarum*	0.181482133	< 0.05	
29	*Coprobacillus sp. 8_2_54BFAA*	0.178496138	< 0.05	
30	*Enterococcus casseliflavus*	0.178162962	< 0.05	
31	*Clostridium clostridioforme*	0.177994461	< 0.05	
32	*Coprobacillus sp. 3_3_56FAA*	0.174524684	< 0.05	
33	*Clostridium bolteae*	0.16973771	< 0.05	
34	*Clostridiaceae bacterium MS3*	0.169523636	< 0.05	
35	*Eubacterium rectale*	0.168435303	< 0.05	
36	*Collinsella sp. CAG:166*	0.165024281	< 0.05	
37	*Clostridioides difficile*	0.159815683	< 0.05	
38	*Lactobacillus salivarius*	0.159223625	< 0.05	
39	*Coprobacillus sp. D7*	0.156843649	< 0.05	
40	*Coprobacillus sp. CAG:183*	0.15670081	< 0.05	
41	*Bifidobacterium catenulatum*	0.156549957	< 0.05	

Supplementary Table 10: List of metabolites grouped according to three main carbohydrate metabolic pathways based on KEGG database

Glycolysis-associated Intermediate Metabolites	Butyrate Intermediate Metabolites	Propionate Intermediate Metabolites
[107689] L-(+) lactic acid [6.851]	(R)-3-Hydroxybutyric acid, trimethylsilyl ether, trimethylsilyl ester	1H-Indole-3-propanoic acid, 1-(trimethylsilyl)-, trimethylsilyl ester
[17106] L-(-)-fucose 1 [15.614]	(R*,S*)-3,4-Dihydroxybutanoic acid triTMS	3-Phenyl-3-trimethylsilyloxypropanoic acid, trimethylsilyl ester
[439215] galacturonic acid 1 [17.917]		Propanoic acid, 2,3-bis[(trimethylsilyl)oxy]-, trimethylsilyl ester
[439746] 6-deoxy-D-glucose 2 [15.751]		Propanoic acid, 2-[(trimethylsilyl)oxy]-, trimethylsilyl ester
[440658] melibiose 2 [25.784]		Propanoic acid, 2-oxo-3-(trimethylsilyl)-, trimethylsilyl ester
[751] DL-glyceraldehyde 1 [8.974]		Propanoic acid, 3-[(trimethylsilyl)oxy]-, trimethylsilyl ester
[751] DL-glyceraldehyde 2 [9.186]		
[84571] lactose 2 [24.534]		
[84571] lactose 2 [24.534]:3		
[84571] lactose 2 [24.534]:4		
[84571] lactose 2 [24.534]:5		
[92904] 3-indolelactic acid 2 [20.077]		
2-Deoxy-galactopyranose, tetrakis(trimethylsilyl)		
2-Deoxy-galactopyranose, tetrakis(trimethylsilyl):2		
2-Desoxy-pentos-3-ulose, bis(methoxime),O,O'-bis(trimethylsilyl)-:2		
4-Ketoglucose, bis(O-methyloxime), tetrakis(trimethylsilyl)		
à-D-Galactopyranoside, methyl 2,3,4,6-tetrakis-O-(trimethylsilyl)-		
Arabinofuranose, 1,2,3,5-tetrakis-O-(trimethylsilyl)-		
d-Erythrotetrofuranose, tris-O-(trimethylsilyl)-		
d-Galactose, 2,3,4,5,6-pentakis-O-(trimethylsilyl)-, o-methyloxyme, (1E)-		
d-Galactose, 2,3,4,5,6-pentakis-O-(trimethylsilyl)-, o-methyloxyme, (1E)-:2		
d-Galactose, 2,3,4,5,6-pentakis-O-(trimethylsilyl)-, o-methyloxyme, (1Z)-		
d-Galactose, 2,3,4,5,6-pentakis-O-(trimethylsilyl)-, o-methyloxyme, (1Z)-:2		
D-Galactose, 6-deoxy-2,3,4,5-tetrakis-O-(trimethylsilyl)-, O-methyloxime		
d-Glucose, 2,3,4,5,6-pentakis-O-(trimethylsilyl)-, o-methyloxyme, (1E)-		
d-Glucose, 2,3,4,5,6-pentakis-O-(trimethylsilyl)-, o-methyloxyme, (1Z)-		
d-Glucose, 2,3,4,5,6-pentakis-O-(trimethylsilyl)-, o-methyloxyme, (1Z)-:2		
d-Mannose, 2,3,4,5,6-pentakis-O-(trimethylsilyl)-, o-methyloxyme, (1Z)-		
D-Ribofuranose, 1,2,3,5-tetrakis-O-(trimethylsilyl)-		
D-Ribose, 2,3,4,5-tetrakis-O-(trimethylsilyl)-		
D-Xylopyranose, 1,2,3,4-tetrakis-O-(trimethylsilyl)-		
D-Xylopyranose, 1,2,3,4-tetrakis-O-(trimethylsilyl)-:2		
Fructose-(D)-O-methyloxime-peak1 (Manual_DP_070809):10		
Fructose-(D)-O-methyloxime-peak1 (Manual_DP_070809):2		
Fructose-(D)-O-methyloxime-peak1 (Manual_DP_070809):3		
Fructose-(D)-O-methyloxime-peak1 (Manual_DP_070809):4		
Fructose-(D)-O-methyloxime-peak1 (Manual_DP_070809):5		
Fructose-(D)-O-methyloxime-peak1 (Manual_DP_070809):6		
Fructose-(D)-O-methyloxime-peak1 (Manual_DP_070809):7		
Fructose-(D)-O-methyloxime-peak1 (Manual_DP_070809):8		
Fructose-(D)-O-methyloxime-peak1 (Manual_DP_070809):9		
Galactose, 2-(acetylamino)-2-deoxy-3,4,5,6-tetrakis-O-(trimethylsilyl)-		
Melibiose, octakis(trimethylsilyl)- (Manual_DP_070809)		
Melibiose, octakis(trimethylsilyl)- (Manual_DP_070809):10		
Melibiose, octakis(trimethylsilyl)- (Manual_DP_070809):12		
Melibiose, octakis(trimethylsilyl)- (Manual_DP_070809):2		
Melibiose, octakis(trimethylsilyl)- (Manual_DP_070809):3		
Melibiose, octakis(trimethylsilyl)- (Manual_DP_070809):4		
Melibiose, octakis(trimethylsilyl)- (Manual_DP_070809):5		
Melibiose, octakis(trimethylsilyl)- (Manual_DP_070809):6		
Melibiose, octakis(trimethylsilyl)- (Manual_DP_070809):7		
Melibiose, octakis(trimethylsilyl)- (Manual_DP_070809):8		
Melibiose, octakis(trimethylsilyl)- (Manual_DP_070809):9		
Ribose		
Ribose:3		
Ribose:4		
Sedoheptulose, o-methyloxime, hexakis-O-(trimethylsilyl)-		
Trimethylsilyl ether of glycerol		
Trimethylsilyl ether of glycerol (Manual_DP_070809):2		
Xylose-MOX-TMS-peak1		
Xylose-MOX-TMS-peak2		
Xylose-MOX-TMS-peak2:2		
